# Effectiveness and safety of commercial Chinese polyherbal preparations combined with menopausal hormone therapy for perimenopausal symptoms: a network meta-analysis of randomized controlled trials

**DOI:** 10.3389/fphar.2026.1861652

**Published:** 2026-07-08

**Authors:** Yiming Chen, Siya Gao, Yiyu Wang, Zilong Zhong, Xiaoxu Fu

**Affiliations:** 1 Chengdu University of Traditional Chinese Medicine, Chengdu, China; 2 TCM Prevention and Treatment of Metabolic and Chronic Diseases Key Laboratory of Sichuan Province, Hospital of Chengdu University of Traditional Chinese Medicine, Chengdu, China; 3 Department of Endocrinology, Hospital of Chengdu University of Traditional Chinese Medicine, Chengdu, China

**Keywords:** commercial Chinese polyherbal preparations, hormone therapy, network meta-analysis, perimenopausal symptoms, systematic review

## Abstract

**Background:**

Perimenopause is a natural physiological transition associated with declining ovarian function and bothersome systemic symptoms. Menopausal hormone therapy (HT) remains the primary treatment, but safety concerns persist. Commercial Chinese polyherbal preparations (CCPPs) combined with HT are increasingly used, yet comparative evidence remains limited. This network meta-analysis (NMA) evaluated the effectiveness and safety of CCPPs plus HT for perimenopausal symptoms.

**Methods:**

Chinese and English databases were searched for randomized controlled trials (RCTs) evaluating CCPPs plus HT. The study followed the reporting guideline for network meta-analyses and was registered in PROSPERO (CRD420261335785). Risk of bias was assessed using the revised Cochrane risk-of-bias tool. Data were synthesized in Stata 18.0, and surface under the cumulative ranking curve values were estimated as exploratory summaries.

**Results:**

Seventy-three RCTs (n = 8,314) involving 11 CCPPs were included. Most studies had some concerns or high risk of bias, mainly owing to inadequate blinding and subjective-outcome vulnerability. Five CCPP + HT regimens significantly reduced the Kupperman Index (KI) compared with HT alone. Linglianhua Granule plus HT showed the largest KI estimate, but was supported by only two trials and low-certainty evidence. Given the very large standardized mean differences and the subjective nature of KI assessment in mostly unblinded trials, these results should be interpreted cautiously. For the Menopause-Specific Quality of Life questionnaire, only Kuntai Capsule plus HT showed significant improvement. The overall effective rate was treated as auxiliary because definitions varied. Dingkun Pill plus HT and Kuntai Capsule plus HT showed favorable estimates versus HT alone for estradiol elevation and follicle-stimulating hormone/luteinizing hormone reduction, respectively. CCPP + HT did not significantly increase reported short-term adverse reactions, but monitoring and follow-up were insufficient to establish safety superiority, equivalence, or long-term safety.

**Conclusion:**

CCPP + HT may be associated with improvements in selected symptom-related and endocrine outcomes compared with HT alone, but the evidence was generally of low to moderate certainty and derived mostly from unblinded trials. Ranking results, particularly for KI, should be considered exploratory summaries and not clinically actionable treatment hierarchies. Further high-quality blinded RCTs with standardized outcome assessment, adverse-event monitoring, and longer follow-up are needed.

**Systematic Review Registration:**

https://www.crd.york.ac.uk/PROSPERO/view/CRD420261335785, identifier CRD420261335785.

## 1 Introduction

Perimenopause is a natural physiological transition from the reproductive to the non-reproductive stage in women, characterized primarily by declining ovarian function and marked fluctuations in sex hormone levels. Perimenopausal symptoms refer to a constellation of physical and psychological symptoms associated with ovarian ageing and the subsequent oscillation or decline of estrogen levels around the time of menopause. Clinical manifestations commonly include hot flashes, sweating, sleep disturbances, emotional volatility, musculoskeletal pain, and urogenital symptoms.

With global population aging, the number of women experiencing the menopausal transition and living after menopause is increasing. These symptoms may substantially affect the Menopause-Specific Quality of Life of middle-aged and older women and may also create a socioeconomic and public-health burden. A recent global health commentary reported that, by 2030, more than 1.2 billion women worldwide will be menopausal or postmenopausal (Delanerolle et al., 2025). In China, this cohort is approximately 167 million, accounting for 23% of the global total (Wang et al., 2025). Epidemiological studies indicate that the overall prevalence of perimenopausal symptoms among Chinese women aged 40–60 ranges from 46.3% to 61.0% (Li et al., 2025; Yong et al., 2025).

These clinical manifestations occur in the context of a multidimensional physiological transition involving the hypothalamic-pituitary-ovarian (HPO) axis. As the primordial follicle reserve declines, ovarian sensitivity to gonadotropins declines significantly, leading to reduced inhibin secretion. This relieves the negative feedback inhibition on the pituitary gland, causing a compensatory rise in follicle-stimulating hormone (FSH) and luteinizing hormone (LH). During this process, estrogen levels transition from cyclic fluctuations to a sustained lower-estrogen state. This change in estrogenic signaling may affect estrogen receptors (ERs) widely distributed throughout bodily tissues. In the central nervous system, declining estrogen levels may contribute to an imbalance of neurotransmitters such as 5-hydroxytryptamine (5-HT) and norepinephrine (NE) in the hypothalamus, narrowing the thermoregulatory threshold and manifesting as classic vasomotor symptoms (VMS) and autonomic dysfunction (Khan et al., 2023). At the same time, lower estrogen levels are associated with systemic physiological changes. In the skeletal system, it leads to bone turnover imbalance and mineral loss due to enhanced osteoclast activity. Furthermore, it may contribute to impaired vascular endothelial vasodilation and pro-atherogenic lipid profile shifts in the cardiovascular system, as well as mucosal atrophy in the urogenital system. In summary, perimenopausal symptoms arise during a multidimensional physiological transition involving neuroendocrine, metabolic, and multi-organ changes, rather than representing menopause itself as a pathological condition. Parallel to these modern endocrine insights, Traditional Chinese Medicine (TCM) describes this stage as the exhaustion of Tiangui and depletion of essence and blood, with the core pathogenesis identified as Kidney deficiency (Chen X., 2020). Kidney deficiency not only disrupts the reproductive axis but is often accompanied by patterns such as Liver Qi stagnation, causing emotional depression and the disharmony between the Heart and Kidney, leading to severe insomnia.

To address bothersome symptoms associated with these neuroendocrine changes, Menopausal Hormone Therapy (HT), including estrogen-only therapy, combined estrogen-progestogen therapy, or Tibolone, remains the gold standard and first-line regimen for alleviating VMS and genitourinary syndrome of menopause (Khan et al., 2023). HT effectively restores negative feedback to the hypothalamus and stabilizes the neurotransmitter system, providing significant relief for over 80% of symptomatic women (Atwell et al., 2024). However, HT alone has recognized limitations. Long-term or irregular use of HT may increase the risk of endometrial thickening, breast cancer, venous thromboembolism, and cardiovascular disease (Chlebowski and Aragaki, 2023). These potential adverse effects and contraindications significantly restrict its widespread clinical application and contribute to poor treatment adherence. Consequently, there is an urgent need to identify adjunctive therapeutic strategies that may improve symptom control and treatment acceptability while maintaining an acceptable short-term safety profile. Whether such adjunctive strategies allow reduction of hormone dosage remains uncertain and was not directly evaluated in the present NMA.

In response to this need, commercial Chinese polyherbal preparations (CCPPs) combined with HT have been increasingly explored as adjunctive treatments for perimenopausal symptoms. From a pharmacological perspective, botanical drugs and related bioactive compounds contained in CCPPs have been investigated in preclinical or pharmacological studies in relation to neuroendocrine regulation, ovarian function, neurotransmitter balance, oxidative stress, inflammation, metabolic regulation, and endometrial cellular processes (Wang Y. et al., 2022; Shang et al., 2023; Chen et al., 2026; Li et al., 2026; Chen et al., 2018; Su et al., 2020; Liu X.-L. et al., 2023; Bi et al., 2025; Tan et al., 2025; Zhang et al., 2025b; Wu et al., 2023; Liu et al., 2025; Zhang X. et al., 2025; Yan et al., 2020; Liang et al., 2023; Yong et al., 2024; Zhong et al., 2024; Ma et al., 2025; Liu S. et al., 2023). These findings provide biological plausibility for studying CCPPs as adjunctive therapies to HT. However, these mechanisms are mainly derived from preclinical evidence and were not directly assessed in the included RCTs. Therefore, they should not be interpreted as direct mechanistic evidence for the clinical effects observed in this NMA.

While numerous randomized controlled trials (RCTs) have evaluated CCPPs combined with HT, the variety of CCPPs used in clinical practice is extensive. Existing meta-analyses are largely limited to pairwise comparisons between a single CCPP and conventional hormone therapy, lacking comprehensive direct or indirect comparisons between various CCPP combination regimens (Zhou et al., 2016; Tang et al., 2025). Consequently, clinicians lack systematically summarized comparative evidence regarding different CCPP + HT combinations. Network meta-analysis (NMA) allows the integration of direct and indirect evidence to compare multiple interventions within a connected evidence network. Therefore, this study employed a frequentist NMA approach to compare the efficacy and safety of 11 CCPPs combined with HT for perimenopausal symptoms by integrating direct and indirect evidence across the treatment network. The objective was to provide preliminary comparative evidence on different CCPP + HT regimens and to summarize outcome-specific relative effect patterns, while recognizing that ranking metrics should be interpreted as exploratory summaries rather than definitive clinical recommendations.

## 2 Methods

This systematic review and NMA was conducted and reported in strict accordance with the Preferred Reporting Items for Systematic Reviews and Meta-Analyses for Network Meta-Analyses (PRISMA-NMA) extension statement, as detailed in Supplementary Appendix 1; (Hutton et al., 2015; Page et al., 2021). The study protocol was registered on the PROSPERO platform (CRD420261335785).

### 2.1 Eligibility criteria

The inclusion criteria for this study were developed based on the PICOS principle. In line with the clinical question specified in the registered protocol, timing-related factors, including treatment duration and outcome assessment time point, were extracted and considered when assessing clinical comparability and interpreting subgroup analyses. Regarding participants (P), we included adult participants/women (aged 18 years or older) with perimenopausal symptoms diagnosed or staged according to established criteria. Diagnostic criteria were required to refer to the “Chinese Guidelines for Menopause Management and Hormone Supplement Therapy (2023 Edition)” or the international reproductive aging staging standard (STRAW+10). Patients with severe hepatic or renal dysfunction, malignant tumors, or psychiatric disorders were excluded. Regarding interventions (I), the experimental group must receive CCPPs combined with HT. Based on preliminary searches and clinical application status, 11 specific CCPPs with available clinical evidence were selected for inclusion: Kuntai Capsule (KT), Dingkun Pill (DK), Liuwei Dihuang Pill (LWDH), Dizhen Granule (DZ), Xiangshao Granule (XS), Shugan Granule (SG), Gengnianshu Tablet (GNS), Linglianhua Granule (LLH), Guanhuangmu Granule (GHM), Zhibai Dihuang Pill (ZBDH), and Wuji Baifeng Pill (WJBF). HT included estrogen-only therapy, progestogen-only therapy, combined estrogen-progestogen therapy, and tibolone. For the primary NMA, background HT regimens were grouped as the common comparator node because the clinical question specified in the protocol was whether adding a CCPP to the corresponding HT regimen improved outcomes compared with the same HT regimen alone. However, estrogen-only therapy, progestogen-only therapy, combined estrogen-progestogen therapy, and tibolone were not assumed to be fully interchangeable. Background HT type was therefore treated as a key potential effect modifier, extracted at the trial level, considered in the transitivity assessment, and further explored in subgroup or sensitivity analyses where data permitted. Regarding comparisons (C), eligible studies included CCPP + HT versus HT alone and the limited direct active-comparator trials between different CCPP + HT regimens. The primary outcome was the Kupperman Index (KI). Key secondary outcomes included the Menopause-Specific Quality of Life (MENQOL), sex hormone levels—estradiol (E2), follicle-stimulating hormone (FSH), and luteinizing hormone (LH)—endometrial thickness, and adverse reactions. The overall effective rate was analyzed as an auxiliary outcome because its definition varied across trials and often incorporated composite or subjective clinical judgment. Regarding study design (S), only RCTs were included, regardless of blinding or allocation concealment. Exclusion criteria consisted of non-RCT studies such as retrospective studies, case reports, reviews, and animal experiments; interventions combined with other TCM external therapies like acupuncture or massage; and studies with duplicate publications, logical data errors, or inaccessible full texts/key data.

### 2.2 Search strategy

Two researchers independently conducted electronic searches across English and Chinese databases, including PubMed, Embase, Cochrane Library, Web of Science, China National Knowledge Infrastructure (CNKI), Wanfang Data, VIP Database, and China Biology Medicine (CBM). The search period spanned from database inception to January 2026. Furthermore, the reference lists of included studies, relevant systematic reviews, and academic conference proceedings were manually searched to minimize the risk of missing relevant literature. The search strategy utilized a combination of Medical Subject Headings (MeSH) and free-text terms, including “perimenopausal,” “menopause,” “Chinese patent medicine,” “Kuntai capsule,” “Tibolone,” “estrogen and progestogen,” and “randomized controlled trial,” along with their synonyms. The full search strategy for each electronic database is provided in Supplementary Appendix 2. The term “Chinese patent medicine” was retained as a bibliographic search term because it is commonly used in Chinese and English databases, whereas the manuscript uses “commercial Chinese polyherbal preparations (CCPPs)” as the preferred scientific terminology.

### 2.3 Data extraction

EndNote software was utilized to remove duplicates from the search results. Two researchers independently screened the titles and abstracts, followed by a full-text review to determine final inclusion. Any disagreements were resolved through discussion with a third senior researcher. Extracted data included the first author, publication year, sample size, mean age, symptom duration, specific interventions, names and treatment courses of CCPPs and HT regimens, control measures, and the mean and standard deviation or event counts for each outcome indicator. For studies reporting multiple follow-up time points, only data from the primary observation endpoint or the end of the treatment course were extracted. For adverse reactions, total event counts were extracted for quantitative synthesis when available, and specific event categories were extracted descriptively. When a specific adverse-event category was not reported in an original RCT, it was recorded as NR, indicating non-reporting rather than absence of that event. In addition to the main outcome data, we extracted available trial-level characteristics relevant to clinical comparability, including background HT regimen, treatment duration, mean age, symptom duration, intervention details, and control measures. Baseline symptom severity, baseline KI/MENQOL scores, and TCM syndrome differentiation criteria were not consistently summarized across the included reports and, therefore, could not be incorporated into quantitative effect-modifier analyses. These unmeasured or incompletely reported factors were considered qualitatively when assessing transitivity and interpreting indirect comparisons.

We also summarized the botanical and other drugs included in each CCPP using accessible product instructions, official drug information, pharmacopeial information, or publicly available regulatory information. For plant-derived drugs, accepted scientific names, author citations, families, and taxonomic validation sources were recorded using POWO, MPNS, and/or WFO where applicable; fungal-derived medicinal materials were checked using fungal taxonomic resources where applicable. We also extracted product-level information for each CCPP where available, including manufacturer identity, batch number, production or expiry date, regulatory approval information, quality-control specifications, marker-metabolite or assay information, and whether and how the preparation composition was reported in the original RCT publications. When such information was not reported in the original RCTs, it was recorded as not reported (NR) rather than assumed to be absent. Complete composition and taxonomic validation information are summarized in Supplementary Appendix 3, Supplementary Table S3.2; product-level reporting and composition-reporting status are summarized in Supplementary Table S3.3; and shared botanical and other drugs across CCPPs are summarized in Supplementary Table S3.4. Product-level reporting was checked against the review-relevant items of the ConPhyMP best-practice tool, and the completed ConPhyMP Supplementary File 1 were provided as separate PDF files (Heinrich et al., 2022).

### 2.4 Risk of bias assessment

Two researchers independently evaluated the quality of the RCTs using the Cochrane RoB 2.0 tool, covering five domains: randomization process, deviations from intended interventions, missing outcome data, measurement of the outcome, and selection of the reported result (Sterne et al., 2019). Each domain was rated as “Low risk,” “Some concerns,” or “High risk.” For measurement of the outcome (D4), particular attention was paid to whether the outcome was subjective or objective and whether participants, personnel, or outcome assessors were blinded. Trials reporting subjective or patient-reported outcomes without adequate blinding or blinded outcome assessment were generally rated as having some concerns for outcome measurement. Objective laboratory or imaging-based outcomes, such as E2, FSH, LH, and endometrial thickness, were considered less vulnerable to outcome-measurement bias. The overall risk of bias profile for all included studies was visualized using risk of bias summary plots and bar charts. Disagreements were resolved through consultation or third-party arbitration. Sensitivity analyses were conducted for KI to assess the impact of studies with a high risk of bias, small sample sizes, and progestogen-only controls.

### 2.5 Statistical analysis

All statistical analyses were performed using Stata 18.0 software with the “network” package and “mvmeta” command within a frequentist random-effects framework. Binary variables, such as the overall effective rate and adverse reactions, were evaluated using the Odds Ratio (OR) or Risk Ratio (RR) with 95% Confidence Intervals (CI). Because the definition of overall effective rate was not standardized across trials and often included subjective or composite clinical judgment, this outcome was analyzed as an auxiliary endpoint and was not used as a primary basis for clinical interpretation or treatment ranking. Continuous variables were evaluated using Mean Difference (MD) when outcome measures were consistent across all included trials, such as sex hormone levels and endometrial thickness. Standardized Mean Difference (SMD) was employed for symptom-scale outcomes such as KI and MENQOL because these patient-reported scales may involve different versions, score ranges, or reporting conventions across trials. However, SMD cannot fully eliminate measurement heterogeneity, particularly when the exact version or modification of the KI was not clearly specified in the original reports. Therefore, very large SMDs for KI were interpreted with particular caution and were not treated as directly proportional indicators of absolute clinical improvement. All effect sizes are reported with 95% Confidence Intervals (CI). Parameter estimation was conducted using the Restricted Maximum Likelihood (REML) method. Heterogeneity was assessed via τ^2^, where τ^2^ < 0.04 indicates low heterogeneity, 0.04–0.16 low-to-moderate, 0.16–0.36 moderate-to-high, and >0.36 high heterogeneity; the analysis assumes a constant τ^2^ across comparisons and a correlation coefficient of 0.5 between studies. Statistical inconsistency was evaluated using the node-splitting approach and the design-by-treatment interaction model. However, because statistical tests may have limited power in sparse networks, the absence of statistically significant inconsistency was not interpreted as proof that the transitivity assumption was fully satisfied. Treatment rankings were summarized using Surface under the cumulative ranking curve (SUCRA) values and P-scores as exploratory probabilistic summaries of the current evidence network. The interpretation of comparative findings prioritized effect estimates, 95% CIs, certainty of evidence, and the structure of direct and indirect evidence rather than rank order alone. SUCRA rankings were not used as standalone evidence of clinical superiority or treatment recommendation. Because KI was the most frequently reported core symptom outcome and had the most connected evidence network, subgroup and sensitivity analyses were primarily conducted for KI. Pre-specified subgroup analyses were performed according to treatment duration (short course <3 months, standard course = 3 months, and long course >3 months) and background HT type (combined estrogen-progestogen therapy, tibolone, and estrogen-only therapy). Pre-specified sensitivity analyses were conducted by sequentially excluding studies with a high risk of bias, small sample sizes (N < 30 per group), and progestogen-only controls.

These analyses were interpreted as exploratory assessments of potential effect modifiers and partial robustness checks rather than as definitive subgroup-specific comparisons. Extensive subgroup analyses for other outcomes were not performed when the network became disconnected or when the number of studies within subgroups was insufficient. Subgroup analyses by TCM syndrome pattern were considered but were not feasible because syndrome differentiation criteria and syndrome-stratified outcomes were inconsistently reported across the included trials. Publication bias was assessed using funnel plots for outcomes involving 10 or more studies. Furthermore, the certainty of the evidence for primary outcomes was evaluated using the confidence in Network Meta-Analysis (CINeMA) framework, implemented through the CINeMA web application (Nikolakopoulou et al., 2020; Papakonstantinou et al., 2020).

### 2.6 Assessment of transitivity and clinical comparability

The transitivity assumption was assessed before interpreting indirect comparisons by examining whether the distribution of potential effect modifiers was sufficiently comparable across intervention nodes. The main variables considered were background HT regimen, treatment duration, age, symptom duration, intervention characteristics, and available baseline symptom information. Background HT regimen was regarded as a key potential effect modifier because the common HT node included estrogen-only therapy, progestogen-only therapy, combined estrogen-progestogen therapy, and tibolone, which may differ in mechanisms, eligible patient populations, indications, and expected effects on symptoms and endocrine outcomes. Treatment duration was also considered clinically relevant because the included trials ranged from 1 to 6 months.

Several potentially important effect modifiers could not be fully assessed. Baseline symptom severity, baseline KI/MENQOL scores, TCM syndrome pattern, and explicit syndrome differentiation criteria were inconsistently reported across the included trials and were not sufficiently standardized for quantitative meta-regression. Therefore, transitivity could not be fully verified. To partially address this limitation, we conducted exploratory KI subgroup analyses according to treatment duration and background HT type, including a restricted analysis within the combined estrogen-progestogen subgroup where data permitted. We also performed a sensitivity analysis excluding progestogen-only studies. These analyses were interpreted as partial assessments of clinical heterogeneity rather than as definitive confirmation of transitivity.

## 3 Results

### 3.1 Literature search results

**FIGURE 1:**
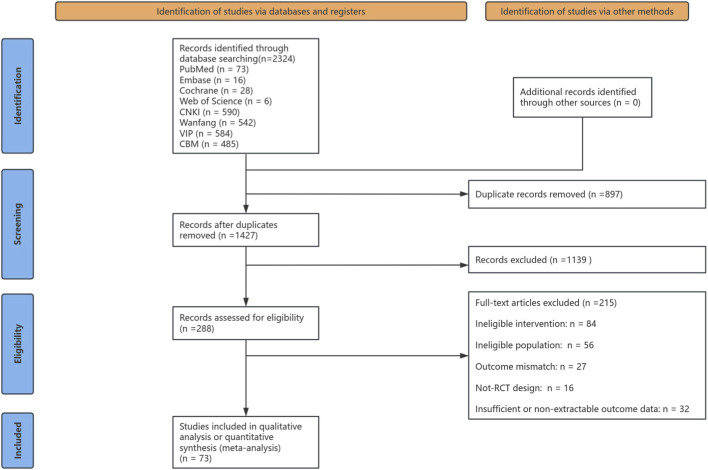
Flowchart of literature search and selection process.

A systematic search of eight electronic databases identified 2,324 records in total (PubMed, n = 73; Embase, n = 16; Cochrane Library, n = 28; Web of Science, n = 6; CNKI, n = 590; VIP, n = 584; Wanfang, n = 542; CBM, n = 485). After the removal of 897 duplicates, 1,427 unique records underwent title and abstract screening, of which 1,139 were excluded. The remaining 288 records were retrieved for full-text assessment, and 215 were subsequently excluded for the following reasons: ineligible intervention (n = 84), ineligible population (n = 56), outcome mismatch (n = 27), not-RCT design (n = 16), and insufficient or non-extractable outcome data (n = 32). Finally, 73 RCTs (An, 2024; Cai, 2016; Chen, 2013; Chen, 2019; Chen M. M., 2020; Chen, 2022; Chen, 2023; Chen and Chen, 2024; Chen and Dong, 2020; Chen et al., 2022; Chen L. M. et al., 2024; Cuan et al., 2020; Ding et al., 2018; Ding and He, 2025; Fan, 2018; Fang and Sun, 2024; Fei et al., 2024; Fu, 2021; Geng et al., 2021; Gong, 2016; Hou and Li, 2017; Ji et al., 2016; Ju et al., 2023; Kong et al., 2025; Li, 2019; Li and Zhu, 2022; Li X. H. et al., 2021; Li et al., 2022; Li et al., 2023; Liao, 2021; Liu, 2020; Liu and Hu, 2025; Liu and Li, 2022; Liu and Sun, 2016; Liu et al., 2020; Lu et al., 2018; Ouyang et al., 2022; Qi et al., 2025; Ren et al., 2019; Shan, 2019; Shi, 2019; Shi et al., 2022; Tang, 2003; Wang, 2025; Wang and Cao, 2023; Wang and Zhao, 2024; Wang et al., 2017; Wang et al., 2020; Wang Y. H. et al., 2022; Wang et al., 2023; Wei, 2020; Wu, 2022; Wu and Dai, 2025; Wu and Guo, 2023; Wu et al., 2014; Xi et al., 2021; Xiang et al., 2025; Yan, 2015; Yan et al., 2019; Yang and Tang, 2019; Yang et al., 2017; Yin et al., 2025; Yu and Jin, 2025; Yuan, 2015; Zeng, 2023; Zhang, 2019; Zhang et al., 2025b; Zhao and Zhai, 2013; Zhao et al., 2024; Zhao et al., 2026; Zhou, 2015; Zhou, 2023; Zhu et al., 2023) fulfilled all pre-specified eligibility criteria and were included in the quantitative NMA (Figure 1).

### 3.2 Characteristics of included studies

A total of 73 RCTs published between 2003 and 2026 were included, encompassing 8,314 patients (4,166 in the treatment group and 4,148 in the control group). All trials compared a CCPP combined with HT versus the corresponding HT regimen alone. In the primary NMA, HT regimens were grouped as the common comparator node, including estrogen-only therapy, progestogen-only therapy, combined estrogen-progestogen therapy, and tibolone. Because these HT regimens may differ in mechanisms, eligible patient populations, and expected clinical effects, background HT type was considered a potential source of clinical heterogeneity and a possible threat to transitivity.

Research frequency varied significantly across the 11 identified CCPPs. While KT (n = 22) and DK (n = 13) constituted the core nodes of the evidence network, LLH (n = 2) and WJBF (n = 2) were classified as sparse-evidence nodes, defined as intervention nodes supported by ≤ 2 trials. Estimates and ranking metrics involving these nodes were considered potentially unstable and were therefore interpreted as exploratory signals rather than as evidence of definitive clinical superiority. The majority of enrolled participants were aged 45–55 years, with mean ages across the different intervention nodes ranging from 47.54 ± 3.57 to 52.33 ± 2.97 years. Symptom duration typically spanned 1.01–4.41 years. Regarding background hormone regimens, combined estrogen and progestogen was the most frequently utilized, followed by tibolone, while some trials specifically employed progesterone or estrogen monotherapy. Treatment duration ranged from 1 to 6 months, with most studies adopting a 3-month (approximately three cycles) regimen. The variation in treatment duration and background HT regimen was therefore considered when interpreting indirect comparisons and ranking estimates. Detailed baseline characteristics of the 73 included RCTs are summarized in Supplementary Appendix 3, Supplementary Table S3.1.

Concerning pharmacological characteristics, the 11 included CCPPs were categorized into three primary functional groups based on TCM functional characteristics. The first group focuses on nourishing Yin and clearing internal heat, targeting Kidney Yin deficiency; this includes KT, LWDH, DZ, GHM, GNS, LLH, and ZBDH. Preclinical studies have reported that botanical drugs and related bioactive compounds contained in these formulations may be related to neuroendocrine or inflammatory pathways. The second group focuses on tonifying Qi and nourishing Blood, addressing patterns of Qi–Blood deficiency; this includes DK and WJBF. From a TCM perspective, these formulations are commonly used to tonify Qi and nourish Blood; however, the clinical effects of these functional categories were not directly evaluated in the present NMA. The third group focuses on soothing the Liver and relieving Qi stagnation, targeting patterns of Liver Qi stagnation; this includes XS and SG. These formulations are traditionally used to soothe Liver Qi and regulate emotional symptoms, but the underlying neuroendocrine mechanisms were not directly assessed in the included trials. This pharmacological and TCM functional diversity provides a rationale for comparing different CCPP + HT combinations. However, the present NMA compared clinical outcomes rather than directly testing pharmacological mechanisms. The botanical and other drugs included in the 11 CCPPs, together with taxonomic validation information for plant-derived drugs, are listed in Supplementary Appendix 3, Supplementary Table S3.2.

At the product-composition and reporting level, the 11 included CCPPs contained botanical drugs together with fungal-derived, animal-derived, shell-derived, chemical or vitamin ingredients, compound processed materials, and excipients. Among 75 product-level records from 73 RCTs, 38 reported full or near-complete formula-style composition, 28 reported only partial or representative composition information, and 9 did not report any drug-composition information in the trial publication itself (Supplementary Appendix 3, Supplementary Table S3.3). Shared recurrent botanical or other drugs across preparations included Rehmannia-derived drugs, Paeoniae Radix Alba, Poria, Glycyrrhizae Radix et Rhizoma, Angelicae Sinensis Radix, Cyperi Rhizoma, Moutan Cortex, and several animal-derived materials. These overlaps are summarized descriptively in Supplementary Appendix 3, Supplementary Table S3.4 and do not imply equivalent dose, extraction process, quality-control specification, pharmacological activity, or clinical effect.

### 3.3 Risk of bias assessment

**FIGURE 2:**
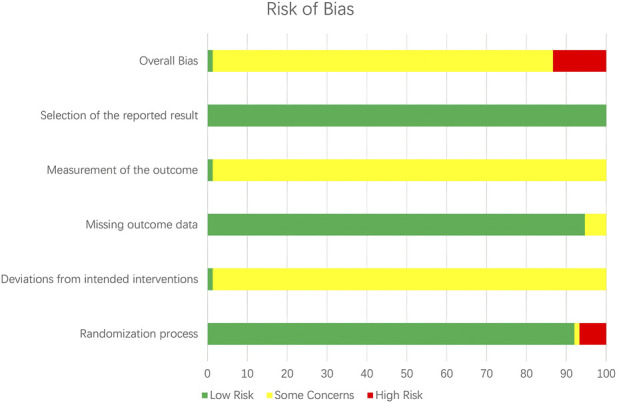
Risk-of-bias summary of the included RCTs assessed using RoB 2.0.

Risk of bias was assessed for all 73 RCTs using the Cochrane RoB 2.0 tool (Figure 2). For the randomization process (D1), 67 trials (91.8%) were rated as low risk, 1 (1.4%) as some concerns, and 5 (6.8%) as high risk owing to inadequate description of sequence generation or allocation concealment. Regarding deviations from intended interventions (D2), 72 trials (98.6%) were rated as some concerns, predominantly reflecting the open-label nature of oral CCPP administration, where blinding of participants and personnel was not implemented. Missing outcome data (D3) was rated as low risk in the vast majority of trials (n = 71, 97.3%). For measurement of the outcome (D4), 1 trial (1.4%) was rated as low risk, and 72 trials (98.6%) were rated as having some concerns. This judgment primarily reflected the lack of adequate blinding or unclear reporting of blinded outcome assessment in most trials, which may have affected subjective or patient-reported outcomes such as KI, MENQOL, and the overall effective rate. By contrast, objective laboratory or imaging-based outcomes, including E2, FSH, LH, and endometrial thickness, were considered less vulnerable to outcome-measurement bias. Selection of the reported result (D5) was generally judged as low risk when the reported outcomes were consistent with the stated methods. Overall, the vast majority of trials were judged as having some concerns, and a small proportion were judged as high risk. These limitations were mainly attributable to inadequate blinding, incomplete reporting of allocation concealment, and the vulnerability of subjective outcomes to awareness of treatment allocation. The detailed item-level and overall judgments are presented in Supplementary Appendix 4 and Supplementary Table S4.

### 3.4 Consistency, heterogeneity, and publication bias

No statistically significant global inconsistency was detected across the evidence network using the design-by-treatment interaction model (all P > 0.05). However, node-splitting analysis identified a large divergence on the SMD scale between direct and indirect estimates for the KT + HT versus LLH + HT loop, with P = 0.108. Although this did not reach the conventional P < 0.05 threshold, the high standard error in the indirect chain (SE > 25) reduced the power to detect inconsistency. Consequently, rankings for sparse-evidence nodes, particularly LLH + HT and WJBF + HT, should be interpreted with particular caution. Because these nodes were supported by only two trials, their SUCRA values and rank positions may be unstable, and the addition of a small number of future trials could substantially change their relative order.

The results of global inconsistency testing for all clinical outcomes are presented in Supplementary Appendix 5, Supplementary Table S5.1. No statistically significant global inconsistency was detected for most outcomes. However, the absence of statistically significant inconsistency should not be interpreted as definitive evidence that the transitivity assumption was fully satisfied, particularly because several networks were sparse and had limited direct active-comparator evidence.

Regarding heterogeneity and clinical comparability, τ^2^ values varied across outcomes. Although several available characteristics, including age, symptom duration, and treatment duration, appeared broadly comparable across some intervention nodes, transitivity could not be fully verified. The common HT comparator included clinically heterogeneous background regimens, including estrogen-only therapy, progestogen-only therapy, combined estrogen-progestogen therapy, and tibolone. These regimens may differ in mechanisms, eligible patient populations, and expected effects on symptoms and endocrine outcomes. In addition, treatment duration ranged from 1 to 6 months. Baseline symptom severity, baseline KI/MENQOL scores, and TCM syndrome differentiation criteria were not consistently reported, limiting our ability to assess whether these factors were balanced across treatment nodes. Therefore, indirect comparisons and SUCRA rankings should be interpreted cautiously. The qualitative assessment of transitivity across intervention nodes is presented in Supplementary Appendix 5, Supplementary Table S5.7.

Visual inspection of the comparison-adjusted funnel plots revealed a generally symmetrical distribution of studies. Visual inspection did not suggest obvious small-study effects; however, publication bias could not be completely excluded, particularly because most included studies were single-center Chinese RCTs. Comparison-adjusted funnel plots for evaluating potential publication bias are provided in Supplementary Appendix 9.

For the auxiliary outcome of overall effective rate, the global inconsistency test approached but did not reach statistical significance (P = 0.06). However, node-splitting analysis identified statistically significant local inconsistency in selected comparisons, including XS + HT versus KT + HT and KT + HT versus HT. This finding suggests that direct and indirect evidence were not fully compatible for parts of the overall-effective-rate network. Because this endpoint was non-standardized across trials and often incorporated composite or subjective clinical judgment, the consistency-model estimates and SUCRA rankings for this outcome were interpreted only as descriptive supplementary evidence and were not used as a primary basis for clinical recommendations.

### 3.5 KI

**FIGURE 3:**
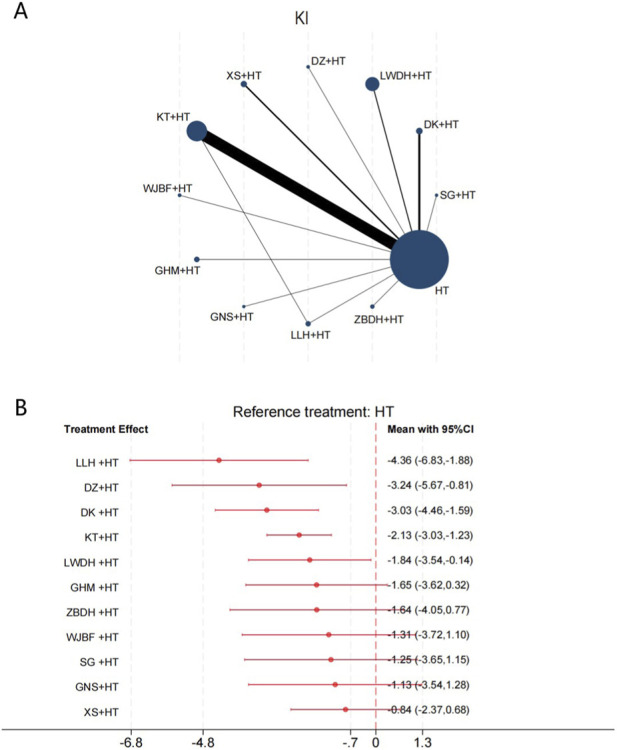
Network meta-analysis of the KI. **(A)** Evidence network plot for KI. Nodes represent individual interventions (CCPPs combined with HT or HT alone), with node size proportional to the total number of randomized patients. Edges represent direct trial comparisons, with edge thickness proportional to the number of contributing trials. The presence of closed loops indicates that both direct and indirect evidence contribute to the analysis. **(B)** Forest plot of pooled SMDs with 95% CIs for each CCPP-based intervention combined with HT versus HT alone (reference treatment). An SMD <0 indicates a greater reduction in KI score compared with HT alone; the vertical dashed line represents the null effect (SMD = 0). Because KI is a subjective symptom score and SMDs may be influenced by within-study standard deviations and differences in KI versions or reporting conventions, very large SMDs should not be interpreted as directly proportional to absolute clinical improvements. Sparse-evidence nodes were defined as intervention nodes supported by ≤ 2 trials. Estimates involving sparse nodes, particularly LLH + HT and WJBF + HT, should be interpreted cautiously.

Regarding the improvement of the KI, this NMA included 44 RCTs involving 5,185 participants across 12 intervention nodes. The evidence network (Figure 3A) was primarily centered on HT and only partially departed from a star-shaped structure because limited direct active-comparator evidence was available between selected intervention nodes, such as KT + HT versus LLH + HT. As shown in the forest plot (Figure 3B), several CCPP + HT combinations showed favorable estimates for reducing KI scores compared with HT alone, and five regimens reached statistical significance.

For KI, five regimens showed statistically significant reductions compared with HT alone. LLH + HT showed the largest effect estimate (SMD -4.36, 95% CI -6.83 to −1.88; low certainty), followed by DZ + HT (SMD -3.24, 95% CI -5.67 to −0.81; low certainty), DK + HT (SMD -3.03, 95% CI -4.46 to −1.59; low certainty), KT + HT (SMD -2.13, 95% CI -3.03 to −1.23; moderate certainty), and LWDH + HT (SMD -1.84, 95% CI -3.54 to −0.14; low certainty). However, several KI effects were very large on the SMD scale, particularly for LLH + HT, DZ + HT, and DK + HT. These large SMDs should not be interpreted as proportional clinical effects or reliable evidence of large absolute symptom improvement. Their interpretation is limited by the subjective nature of KI, inadequate blinding in most included trials, inconsistent reporting of KI versions or modifications, and sparse or low-certainty evidence for several intervention nodes. LLH + HT was supported by only two trials and should therefore be regarded as a sparse-evidence signal rather than as evidence of a definitive optimal regimen. Overall, KI findings should be interpreted primarily as low- to moderate-certainty estimates versus HT alone, rather than as reliable evidence of comparative superiority among active CCPP + HT regimens. Given the limitations of KI and the sparse evidence for several nodes, detailed KI SUCRA rankings and cumulative probability plots are presented only in Supplementary Appendix 7, Supplementary Figure S7.1, and Supplementary Table S7.1. These rankings should be regarded only as exploratory statistical summaries and are not suitable for clinical interpretation or treatment selection. Based on the league table (Supplementary Appendix 7, Supplementary Table S8.1), most active-regimen comparisons between CCPP+HT combinations did not reach statistical significance, except for the comparison involving LLH + HT and XS + HT; however, this finding should be interpreted cautiously because LLH + HT was a sparse-evidence node. Overall, CINeMA assessment results indicated that the certainty of evidence for KI was predominantly of low to moderate levels. The certainty of evidence for the KI, evaluated using the CINeMA framework, is detailed in Supplementary Appendix 12. Because KI is a subjective symptom score with recognized measurement limitations, the very large KI estimates and the corresponding exploratory ranking results should be interpreted as symptom-score signals within the available evidence network, rather than as direct evidence of large absolute clinical effects or definitive clinical superiority.

### 3.6 MENQOL

**FIGURE 4:**
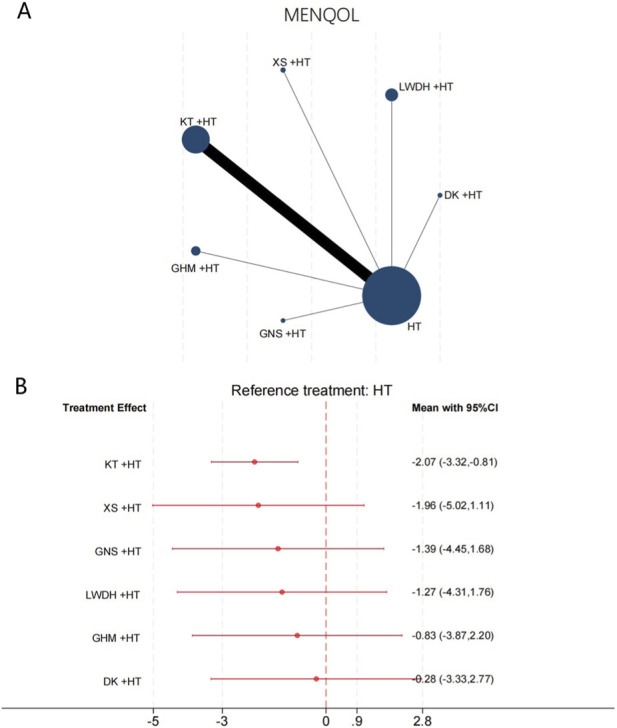
Network meta-analysis of the MENQOL. **(A)** Evidence network plot for MENQOL. Nodes represent individual interventions (CCPPs combined with HT or HT alone), with node size proportional to the total number of randomized patients. Edges represent direct trial comparisons, with edge thickness proportional to the number of contributing trials. The presence of closed loops indicates that both direct and indirect evidence contribute to the analysis. **(B)** Forest plot of pooled SMDs with 95% CIs for each CCPP-based intervention combined with HT versus HT alone (reference treatment). An SMD <0 indicates a greater improvement in quality of life compared to the control; the vertical dashed line represents the null effect (SMD = 0).

For the MENQOL, the NMA incorporated 11 RCTs encompassing 1,299 patients across 7 intervention nodes. The evidence network (Figure 4A) was star-shaped, with HT alone as the central hub directly connected to all 6 CCPP + HT combination nodes. As shown in the forest plot (Figure 4B), several CCPP + HT interventions showed favorable estimates for improving quality of life compared with HT alone; however, only KT + HT showed a statistically significant improvement in the pooled estimate (SMD -2.07, 95% CI -3.32 to −0.81). Most active-regimen comparisons between CCPP + HT combinations did not reach statistical significance. Therefore, the MENQOL results should be interpreted primarily as evidence of a favorable estimate for KT + HT versus HT alone, rather than as reliable comparative evidence among active regimens. Detailed exploratory ranking results, including SUCRA values and cumulative probability plots, are provided in Supplementary Appendix 7, Supplementary Figure S7.2 and Supplementary Table S7.2. According to the league table (Supplementary Appendix 8, Supplementary Table S8.2), most active-regimen comparisons between CCPP + HT combinations did not reach statistical significance.

### 3.7 Auxiliary outcome: overall effective rate

**FIGURE 5:**
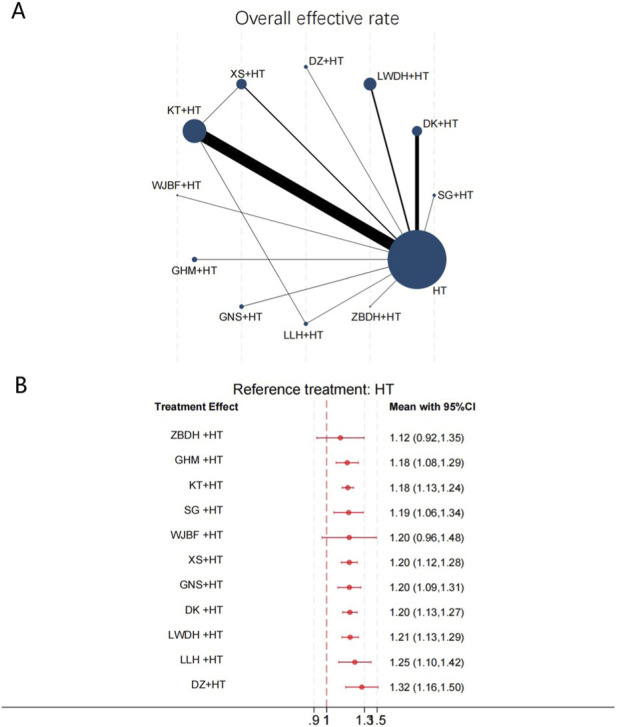
Network meta-analysis of the auxiliary outcome: overall effective rate. **(A)** Evidence network plot for the overall effective rate. Nodes represent individual interventions (CCPPs combined with HT or HT alone), with node size proportional to the total number of randomized patients. Edges represent direct trial comparisons, with edge thickness proportional to the number of contributing trials. The presence of closed loops indicates that both direct and indirect evidence contribute to the analysis. **(B)** Forest plot of pooled RR with 95% CI for each CCPP-based intervention combined with HT versus HT alone (reference treatment). An RR > 1 indicates a higher overall effective rate compared with HT alone; statistical significance should be judged by whether the 95% CI crosses the null value (RR = 1). Because the definition of this endpoint varied across trials and local inconsistency was identified in node-splitting analysis, the results should be interpreted as auxiliary and descriptive evidence.

The overall effective rate was analyzed as an auxiliary outcome because its definition varied across trials and often incorporated composite or subjective clinical judgment. This NMA included 12 intervention nodes based on data from 7,237 participants across 62 studies. The evidence network (Figure 5A) identified HT alone, KT + HT, LWDH + HT, and DK + HT as the core nodes. The network was not a pure star-shaped structure because limited direct active-comparator evidence was available, such as XS + HT versus KT + HT.

As shown in the forest plot (Figure 5B), several CCPP + HT combinations showed higher overall effective rates than HT alone. In the consistency-model analysis, DZ + HT, LLH + HT, and LWDH + HT showed favorable estimates compared with HT alone. However, because this endpoint was non-standardized, partly subjective, and showed statistically significant local inconsistency in node-splitting analysis, these estimates and SUCRA rankings should be regarded as descriptive supplementary findings rather than confirmatory comparative evidence. They were therefore not used to support primary clinical interpretation or treatment recommendations. Detailed ranking results are provided in Supplementary Appendix 7, Supplementary Figure S7.3, and Supplementary Table S7.3, and the full league table is provided in Supplementary Appendix 8, Supplementary Table S8.3.

### 3.8 Sex hormone levels (E2, FSH, and LH)

**FIGURE 6:**
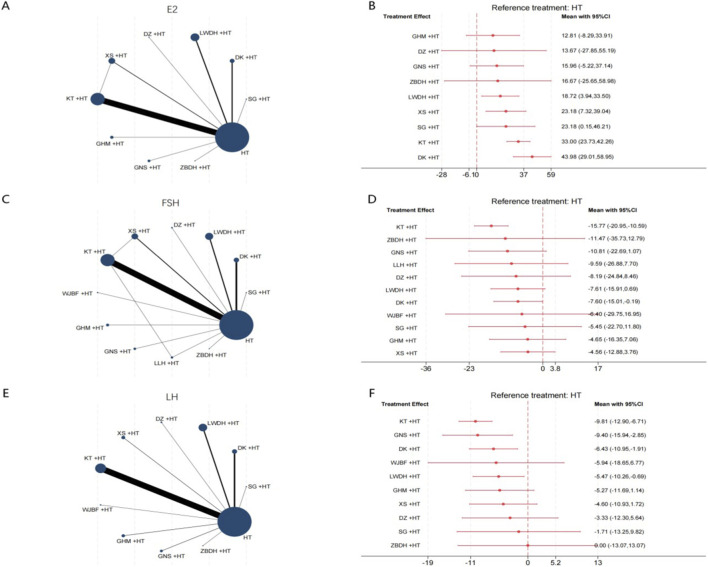
Network meta-analysis of sex hormone levels (E2, FSH, and LH). **(A,C,E)** Evidence network plots for E2, FSH, and LH, respectively. Nodes represent individual interventions (CCPPs combined with HT or HT alone), with size proportional to the total number of randomized patients. Edges represent direct trial comparisons, with thickness proportional to the number of contributing trials. The presence of closed loops indicates that both direct and indirect evidence contribute to the analysis. **(B,D,F)** Forest plots of pooled MDs with 95% CIs for E2, FSH, and LH, respectively, for each CCPP-based intervention combined with HT versus HT alone (reference treatment). For E2 (B), MD > 0 indicates a greater increase in hormone levels compared with HT alone. For FSH (D) and LH (F), MD < 0 indicates a greater reduction in hormone levels compared with HT alone. The vertical dashed line represents the null effect (MD = 0).

The systematic evaluation of sex hormone levels integrated data from 49 to 61 RCTs, encompassing between 5,673 and 7,095 participants across the three endocrine indicators. Within the evidence networks (Figures 6A,C,E), HT alone, KT + HT, LWDH + HT, and DK + HT were identified as the most frequently represented nodes. The network architecture for E2 and FSH displayed a non-star-shaped distribution because limited direct active-comparator evidence was available, such as XS + HT versus KT + HT, whereas the LH network presented a star-shaped distribution centered on HT alone. Across the endocrine outcomes, no statistically significant global inconsistency was detected (P > 0.05). Several CCPP + HT combinations showed favorable estimates for E2 elevation or FSH/LH reduction compared with HT alone, but these findings should be interpreted as outcome-specific endocrine signals rather than direct evidence of restored hormonal balance.

Regarding E2 elevation (Figure 6B), five CCPP+HT regimens showed statistically significant, favorable estimates compared with HT alone. DK+HT showed a favorable estimate and a high exploratory ranking in the current network (MD 43.98, 95% CI 29.01–58.95; SUCRA 95.1%). Other statistically significant regimens included KT+HT (MD 33.00, 95% CI 23.73–42.26; SUCRA 79.7%), SG+HT (MD 23.18, 95% CI 0.15–46.21; SUCRA 56.6%), XS+HT (MD 23.18, 95% CI 7.32–39.04; SUCRA 56.5%), and LWDH+HT (MD 18.72, 95% CI 3.94–33.50; SUCRA 45.3%). However, these rankings should be interpreted as exploratory summaries of the current evidence network and not as definitive evidence of comparative superiority among active CCPP+HT regimens. The detailed ranking results are provided in Supplementary Appendix 7, Supplementary Figure S7.4 and Supplementary Table S7.4.

For gonadotropin reductions (Figure 6D,F), KT+HT showed statistically significant favorable estimates versus HT alone and high exploratory rankings for both FSH reduction (MD -15.77, 95% CI -20.95 to -10.59; SUCRA 85.9%) and LH reduction (MD -9.81, 95% CI -12.90 to -6.71; SUCRA 86.4%). DK+HT also showed favorable estimates for FSH reduction, while GNS+HT and DK+HT showed favorable estimates for LH reduction. However, most active-regimen comparisons did not reach statistical significance, and these endocrine findings should be interpreted as outcome-specific signals rather than reliable evidence of comparative superiority among active regimens. The detailed ranking results are provided in Supplementary Appendix 7, Supplementary Figure S7.5, S7.6 and Supplementary Tables S7.5, S7.6.

League table analyses (Supplementary Appendix 8, Supplementary Tables S8.5, S8.6) suggested statistically significant differences in selected comparisons between CCPP+HT regimens. DK+HT showed a higher E2 estimate than LWDH+HT (MD 25.26, 95% CI 4.24–46.28) and GHM+HT (MD 28.02, 95% CI 2.10–53.95). For FSH reduction, KT+HT showed statistically significant differences compared with XS+HT (MD -11.21, 95% CI -20.65 to -1.76) and HT alone (MD -15.77, 95% CI -20.95 to -10.59). However, most other active-regimen comparisons did not reach statistical significance. These endocrine outcomes provide supportive objective indicators for interpreting symptom-related findings. However, they should not be interpreted as direct mechanistic evidence that CCPP+HT stabilizes the neuroendocrine network, because mechanistic endpoints were not directly assessed in the included trials.

### 3.9 Adverse reactions

**FIGURE 7:**
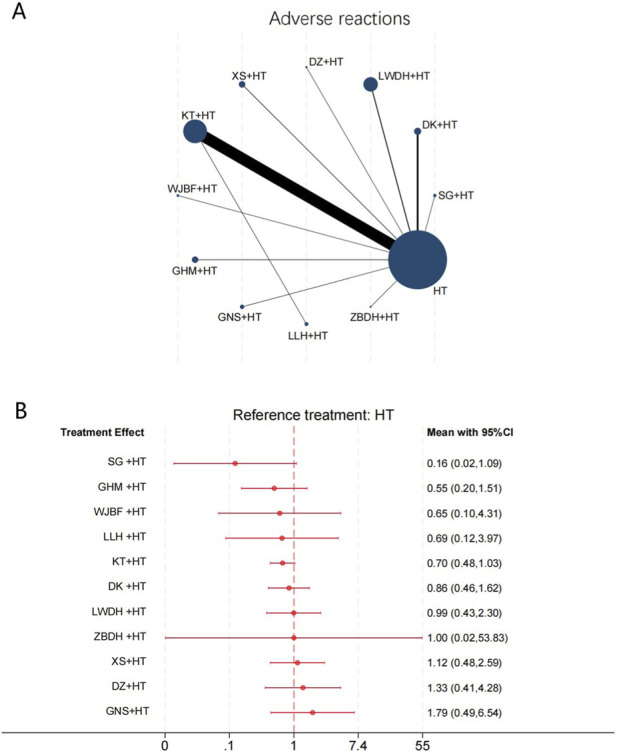
Network meta-analysis of the adverse reactions. **(A)** Evidence network plot for adverse reactions. Nodes represent individual interventions (CCPPs combined with HT or HT alone), with node size proportional to the total number of randomized patients. Edges represent direct trial comparisons, with edge thickness proportional to the number of contributing trials. The presence of closed loops indicates that both direct and indirect evidence contribute to the analysis. **(B)** Forest plot of pooled OR with 95% CI for each CCPP-based intervention combined with HT versus HT alone (reference treatment). An OR < 1 indicates a lower point estimate for adverse reactions compared to the control; the vertical dashed line represents the null effect (OR = 1).Sparse-evidence nodes supported by ≤ 2 trials should be interpreted cautiously. Lower OR point estimates should not be interpreted as safety superiority when confidence intervals are wide, evidence is sparse, or adverse-event reporting is incomplete. NR for specific adverse-event categories indicates non-reporting rather than absence of events.

For adverse reactions, this NMA included 47 RCTs involving 5,434 participants across 12 intervention nodes. The evidence network (Figure 7A) identified HT alone, KT + HT, LWDH + HT, and DK + HT as the core nodes, with limited direct active-comparator evidence such as KT + HT versus LLH + HT. As shown in the forest plot (Figure 7B), CCPP + HT did not show a statistically significant increase in short-term adverse reactions compared with HT alone. Some regimens had lower point estimates for reported adverse reactions, but these estimates were imprecise, and no combination demonstrated definitive safety superiority. SG + HT had a high exploratory ranking (SUCRA 91.8%; OR 0.16, 95% CI 0.02–1.09), but the confidence interval crossed the null value. This ranking should be interpreted cautiously because adverse-event reporting was incomplete, confidence intervals were wide in several comparisons, and sparse-evidence nodes such as WJBF + HT may yield unstable estimates. The detailed ranking results, including SUCRA values and cumulative probability plots for adverse reactions, are provided in Supplementary Appendix 7, Supplementary Figure S7.8 and Supplementary Table S7.8. The league table (Supplementary Appendix 8, Supplementary Table S8.8) suggested a lower adverse-reaction estimate for SG + HT compared with GNS + HT (OR 0.09, 95% CI 0.01–0.90). However, this active-regimen comparison should be interpreted cautiously because of sparse evidence and wide uncertainty across the adverse-event network. Reported adverse reactions mainly included breast tenderness, gastrointestinal discomfort, vaginal spotting, headache, dizziness, and skin rash. However, the reporting of adverse-event categories and monitoring methods was incomplete in many trials, with several studies reporting only total adverse-event counts or not reporting specific event categories. In Supplementary Appendix 13, NR for a specific adverse-event category indicates non-reporting rather than absence of that event. Therefore, the absence of explicitly reported serious adverse events or specific event categories should not be interpreted as evidence that these events did not occur or were systematically excluded. Differential reporting of adverse-event categories across CCPP nodes may also have introduced reporting bias, because nodes with less detailed event reporting could appear to have more favorable safety profiles. Overall, the available evidence suggests only that CCPP + HT did not significantly increase reported short-term adverse reactions compared with HT alone. It does not establish safety superiority, clinical equivalence, or medium- to long-term safety compared with standard HT. A study-by-study breakdown of reported event categories is provided in Supplementary Appendix 13. Potential botanical drug–hormone interactions were not systematically assessed in the included trials. Most studies did not report predefined monitoring of pharmacokinetic interactions, endocrine-related adverse effects, abnormal bleeding patterns, liver function abnormalities, or withdrawals attributable to suspected interactions. Therefore, the present NMA cannot determine whether CCPPs alter the safety profile, metabolism, or clinical effects of background HT.

### 3.10 Endometrial thickness

**FIGURE 8:**
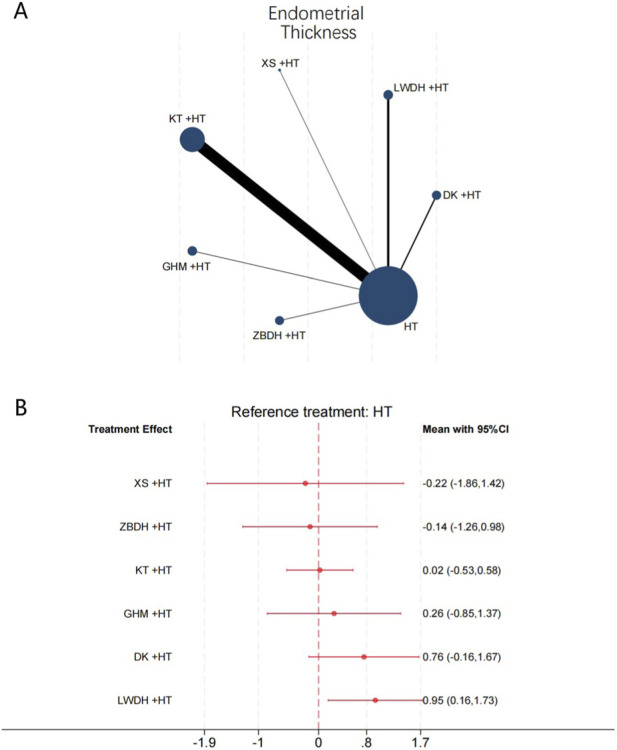
Network meta-analysis of the endometrial thickness. **(A)** Evidence network plot for endometrial thickness. Nodes represent individual interventions (CCPPs combined with HT or HT alone), with node size proportional to the total number of randomized patients. Edges represent direct trial comparisons, with edge thickness proportional to the number of contributing trials. The presence of closed loops indicates that both direct and indirect evidence contribute to the analysis. **(B)** Forest plot of pooled MD with 95% CI for each CCPP-based intervention combined with HT versus HT alone (reference treatment). An MD > 0 indicates an increase in endometrial thickness compared to the control; the vertical dashed line represents the null effect (MD = 0).

For endometrial thickness, this NMA included 21 RCTs involving 2,126 participants across 7 intervention nodes. The evidence network (Figure 8A) was star-shaped, where HT alone, KT + HT, and LWDH + HT served as the core nodes of the evidence chain with the most significant research weight. As shown in the forest plot (Figure 8B), most combination regimens showed no significant difference compared to HT alone, except LWDH + HT, which significantly increased endometrial thickness (MD 0.95, 95% CI 0.16–1.73). Detailed exploratory ranking results are provided in Supplementary Appendix 7, Supplementary Figure S7.7 and Supplementary Table S7.7. Because endometrial thickness is a safety-related or physiological outcome rather than a unidirectional efficacy endpoint, these ranking results should not be interpreted as clinically meaningful superiority among regimens. According to the league table (Supplementary Appendix 8, Supplementary Table S8.7), LWDH + HT resulted in significantly greater thickness than DK + HT (MD 1.17, 95% CI 0.65–2.98).

### 3.11 Subgroup analyses

Exploratory subgroup analyses for KI were conducted according to treatment duration and background HT type to examine potential effect modification. Regarding treatment duration, no analyzed CCPP + HT regimen reached statistical significance in the short-course subgroup (<3 months), whereas several regimens, including LLH + HT, DK + HT, DZ + HT, and KT + HT, showed statistically significant effects in the 3-month or >3-month subgroups. These findings may suggest that treatment duration influences the estimated effects of CCPP + HT combinations; however, they should not be interpreted as establishing a minimum effective treatment duration because the subgroup networks were sparse and the distribution of CCPPs differed across duration categories.

In analyses stratified by background HT type, effect estimates varied across estrogen-only, estrogen-progestogen, and tibolone subgroups. KT + HT showed relatively consistent estimates across available HT-type subgroups, while DK + HT, DZ + HT, LLH + HT, ZBDH + HT, and GHM + HT showed favorable estimates in selected subgroups. These findings suggest that background HT type may act as a potential effect modifier and may influence indirect comparisons in the full network. Because several subgroup networks contained few studies and limited active-comparator evidence, these analyses were interpreted as exploratory and were used primarily to inform the interpretation of clinical heterogeneity and transitivity. The full subgroup results for KI are provided in Supplementary Appendix 10.

### 3.12 Sensitivity analyses

Three pre-specified sensitivity analyses were conducted for KI by sequentially excluding: (1) studies rated as high risk of bias; (2) small-sample studies (N < 30 per arm); and (3) progestogen-only studies. The direction of effect for several major regimens, including DK + HT, DZ + HT, KT + HT, and LLH + HT, was generally maintained across these analyses. However, the interpretation of LLH + HT remained limited by sparse evidence.

Changes in statistical significance were observed for LWDH + HT, which lost statistical significance after excluding high-risk studies and progestogen-only studies. This suggests that the estimate for LWDH + HT may be less stable than those for several other regimens. Overall, these analyses provide partial support for the stability of the main KI findings but do not eliminate concerns related to sparse nodes, heterogeneous HT backgrounds, limited blinding, and incomplete reporting of baseline symptom severity or TCM syndrome differentiation. The full sensitivity analysis results are provided in Supplementary Appendix 11.

Across all outcomes, SUCRA values were used as exploratory probabilistic summaries of the current evidence network. They were interpreted alongside effect estimates, 95% CIs, certainty of evidence, and the availability of direct and indirect evidence, rather than as standalone evidence of clinical superiority or treatment recommendation.

## 4 Discussion

### 4.1 Primary findings

This NMA synthesized 73 RCTs involving 8,314 participants and summarized the comparative evidence for 11 CCPP + HT combinations in perimenopausal symptoms. Overall, several CCPP + HT regimens showed favorable estimates for specific outcomes compared with HT alone. However, the certainty of evidence was generally low to moderate, most trials were unblinded, and most comparisons between active CCPP + HT regimens relied on indirect evidence. Therefore, exploratory ranking results should not be interpreted as definitive evidence of optimal treatment choices. For KI, several CCPP + HT regimens showed statistically significant, favorable estimates versus HT alone. However, some estimates, particularly those for LLH + HT, DZ + HT, and DK + HT, were very large on the SMD scale and should be interpreted cautiously because KI was a subjective outcome, most trials were unblinded, and the certainty of evidence was low for several nodes. These findings should therefore be regarded as preliminary symptom-score signals rather than evidence of reliable comparative superiority among active regimens. For MENQOL, only KT + HT showed statistically significant improvement. For endocrine outcomes, DK + HT and KT + HT showed favorable estimates and high exploratory rankings for E2 elevation and FSH/LH reduction, respectively; however, these findings should be regarded as outcome-specific endocrine signals rather than evidence of reliable comparative superiority. Safety-related rankings should be interpreted conservatively because no regimen demonstrated definitive safety superiority over HT alone. Therefore, the present findings provide preliminary outcome-specific comparative signals rather than direct clinical recommendations.

### 4.2 Interpretation of efficacy findings

This NMA suggests that some CCPP + HT combinations may show favorable estimates across selected efficacy outcomes. However, comparative interpretation should prioritize effect estimates, 95% CIs, certainty of evidence, and the structure of direct and indirect evidence rather than ranking metrics alone. Because most active-regimen comparisons were indirect and several nodes were supported by limited evidence, the ranking results should be regarded as exploratory summaries.

#### 4.2.1 Interpretation of KI and MENQOL findings

KI and MENQOL represent two clinically important but conceptually different patient-reported outcomes. KI mainly reflects the severity of menopausal symptoms, including vasomotor, sleep-related, psychological, and somatic symptoms, whereas MENQOL evaluates broader quality-of-life domains (Lewis et al., 2005). Therefore, the different outcome-specific findings observed for KI and MENQOL should be interpreted as distinct clinical signals rather than as evidence of distinct confirmed pharmacological mechanisms.

For KI, several regimens showed statistically significant favorable estimates versus HT alone, but the magnitude of some SMDs was unusually large. This was particularly evident for LLH + HT, DZ + HT, and DK + HT. Such large SMDs may partly reflect small within-study standard deviations, differences in KI scoring versions or reporting conventions, and subjective-outcome measurement in mostly unblinded trials. Therefore, these estimates should not be interpreted as direct evidence of very large absolute clinical effects. For MENQOL, KT + HT was the only regimen showing statistically significant improvement. Overall, these findings should be interpreted cautiously because KI and MENQOL are patient-reported outcomes, most trials lacked blinding, and direct evidence between active CCPP + HT regimens was limited.

The interpretation of KI-based findings requires additional caution because the Kupperman Index has recognized psychometric limitations (Alder, 1998; Tao et al., 2013). Although KI remains widely used in Chinese RCTs on perimenopausal symptoms and was the most frequently reported symptom outcome in this evidence base, previous methodological critiques have questioned its construct validity, item weighting, and ability to capture the multidimensional nature of menopausal symptoms. In addition, the exact KI version or modification was not consistently specified across the included trials. Although SMD was used to partly account for differences in score ranges or reporting conventions, it cannot fully remove measurement heterogeneity. Therefore, KI-based effect estimates and SUCRA rankings should be interpreted as exploratory symptom-score response patterns rather than as precise evidence of clinical superiority based on a fully homogeneous and psychometrically robust instrument.

Several preclinical and pharmacological studies have suggested that botanical drugs and related bioactive compounds contained in CCPPs may be associated with neuroendocrine regulation, neurotransmitter balance, oxidative stress, inflammatory pathways, sleep-related regulation, ovarian function, lipid metabolism, or bone homeostasis. These include studies on geniposide and *Xylaria nigripes*-related pharmacological constituents in relation to BDNF-related or sleep-related biological activities (Wang et al., 2016; Deshmukh et al., 2021; Li J. et al., 2021), Dingkun Pill-related or ovarian-function-related pathways and ginsenoside pharmacokinetics (Ma et al., 2020; Peng et al., 2012; Wu et al., 2023), catalpol, rehmannioside D, and berberine in relation to oxidative stress and ovarian aging (Yan et al., 2020; Liang et al., 2023; Liu et al., 2024), and berberine, baicalin, or related bioactive compounds in relation to neurotransmitter, thermoregulatory, GABAergic, metabolic, or BDNF-related pathways (Kulkarni and Dhir, 2008; Jiang et al., 2013; Singh et al., 2020; Gao et al., 2024; Wang et al., 2008; Shi et al., 2014; Shen et al., 2016; Ren et al., 2020; Chen M. M. et al., 2024; Tang et al., 2024). Saikosaponins and related bioactive compounds have also been investigated in relation to inflammatory, neuroregulatory, or mood-related pathways (Chen et al., 2018; Liu X.-L. et al., 2023; Tan et al., 2025; Zhang et al., 2025c).

These findings provide biological plausibility for interpreting the observed clinical trends, but they do not establish causal mechanisms for the treatment effects observed in this NMA. The included RCTs did not directly measure mechanistic endpoints such as receptor sensitivity, inflammatory biomarkers, gut–brain–ovarian signaling, neuroendocrine pathway activity, thermoregulatory biomarkers, product-specific chemical profiles, or marker-compound exposure. Therefore, the associations between specific pharmacological constituents and observed clinical effect patterns should be regarded as hypothesis-generating rather than as mechanistic proof.

From a TCM perspective, the included CCPPs can be broadly categorized according to functions such as nourishing Yin and clearing deficiency heat, tonifying Qi and Blood, or soothing Liver Qi. These categories may help contextualize clinical use, but because syndrome differentiation criteria and syndrome-stratified outcomes were inconsistently reported, the present NMA could not evaluate syndrome-specific treatment effects. Thus, the findings should not be interpreted as a validated individualized TCM prescription framework.

#### 4.2.2 Endocrine outcomes and auxiliary overall effective rate

Changes in sex hormone levels provide objective biological indicators that may support the interpretation of symptom-related outcomes. DK + HT showed favorable estimates for E2 elevation, whereas KT + HT showed favorable estimates for FSH and LH reduction.

The overall effective rate should be interpreted with particular caution. Although several CCPP + HT regimens showed favorable estimates for this outcome, the definition of “effectiveness” was not standardized across trials and often incorporated composite criteria, symptom score reductions, or clinician judgment. In addition, node-splitting analysis identified statistically significant local inconsistency for selected comparisons in this network, indicating that direct and indirect evidence were not fully compatible for this endpoint. Therefore, rankings based on the overall effective rate should be regarded as descriptive supplementary information and should not be used as a primary basis for clinical decision-making or comparative treatment recommendations.

#### 4.2.3 Outcome-specific interpretation of findings

Differences in outcome-specific effect patterns were observed between KI, MENQOL, and endocrine outcomes. This may reflect the multidimensional nature of perimenopausal symptoms and the fact that different outcomes capture different clinical constructs. KI mainly evaluates symptom severity, whereas MENQOL reflects broader quality-of-life domains, and endocrine indicators provide objective but indirect biological information. Therefore, differences across outcomes should be interpreted as outcome-specific findings rather than as evidence of a unified pharmacological mechanism or a consistent treatment hierarchy.

Although preclinical studies suggest that botanical drugs and related bioactive compounds contained in CCPPs may influence neuroendocrine regulation, inflammatory pathways, oxidative stress, or neurotransmitter balance, these mechanisms were not directly assessed in the included RCTs. The present NMA also did not evaluate hormone dose reduction, receptor sensitivity, autophagy, cellular repair, product-specific chemical profiles, or marker-compound exposure. Therefore, mechanistic interpretations should be regarded as biological plausibility and hypothesis-generating explanations rather than causal conclusions.

### 4.3 Safety outcomes

The safety findings should be interpreted conservatively. CCPP + HT was not associated with a statistically significant increase in short-term adverse reactions compared with HT alone. However, adverse-event monitoring was insufficiently described in many trials, specific event categories were often incompletely reported, and follow-up durations were generally short. Therefore, the current evidence cannot establish safety superiority, clinical equivalence, or medium- to long-term safety compared with standard HT.

Preclinical studies have explored endocrine, inflammatory, gut-related, and endometrial processes related to botanical drugs and bioactive compounds contained in CCPPs, including saikosaponins, paeoniflorin, geniposide, mangiferin, berberine, and paeonol (Wang et al., 2010; Zhang et al., 2016; Zhang et al., 2022; Wu et al., 2023; Imran et al., 2017; Zhang et al., 2021; Liu S. et al., 2023; Fu et al., 2023). However, these mechanisms were not directly assessed in the included RCTs. The present NMA did not measure estrogen receptor activity, inflammatory biomarkers, gut–brain-axis markers, endometrial molecular endpoints, pharmacokinetic interactions, product-specific chemical profiles, marker-compound exposure, or clinically predefined botanical drug–hormone interaction outcomes. Therefore, these preclinical findings should be regarded as biological plausibility rather than evidence of safety superiority, improved tolerability, or reduced interaction risk.

Future RCTs should prospectively define adverse-event monitoring procedures and report event type, severity, causality, withdrawals due to adverse events, and longer follow-up outcomes.

### 4.4 Interpretation of absence of significant differences between active regimens

In this NMA, several CCPP + HT regimens showed statistically significant estimates compared with HT alone for selected outcomes. However, most comparisons between active CCPP + HT regimens did not reach statistical significance. Although several direct active-comparator paths were available for outcomes such as KI and the auxiliary overall effective rate, the overall volume of direct evidence between active regimens remained limited.

The absence of statistically significant differences between most active CCPP + HT regimens should not be interpreted as evidence of clinical equivalence or a class effect. Rather, it likely reflects limited direct active-comparator evidence, sparse comparisons, wide confidence intervals, and imprecision within the current network. Therefore, the present results cannot establish that different CCPPs have similar pharmacological potency or interchangeable clinical effects.

From a statistical and epidemiological perspective, a rigorous distinction must be made between “absence of significant difference” and “clinical equivalence.” Although our network transcends the limitations of a purely star-shaped topology, the volume of direct evidence between CCPPs remains sparse compared to the evidence for CCPP versus HT comparisons. A distinct dissociation between clinical magnitude and statistical significance was observed in the comparison of LLH and KT regimens. Despite a large divergence on the SMD scale between direct and indirect estimates, the node-splitting test yielded non-significant results. This lack of statistical significance may be partly attributable to the very large standard errors in the indirect evidence, indicating that the current network may have had limited power to detect potential inconsistency. Therefore, the pooled estimates for these specific nodes should be interpreted with analytical caution. Consequently, SUCRA rankings should be interpreted only as exploratory probabilistic summaries of the current evidence network. Sparse nodes, such as LLH + HT, should be interpreted in light of their limited sample size and small evidence contribution, regardless of their ranking position. The inclusion of even a small number of future high-quality trials could substantially change both the effect estimates and the relative ordering. This underscores the need for caution when interpreting evidence-sparse regimens. Future research should include adequately powered, direct active-comparator trials among promising regimens to generate more conclusive comparative evidence.

### 4.5 Subgroup and sensitivity analyses

Subgroup analyses were conducted to explore whether treatment duration and background HT type may act as potential effect modifiers. Some regimens showed statistically significant effects in the 3-month and >3-month subgroups, whereas no regimen reached statistical significance in the short-course subgroup (<3 months). However, these findings should not be interpreted as definitive evidence of a duration-dependent treatment effect because subgroup networks were sparse and unevenly distributed. These subgroup findings may reflect differences in treatment duration and study characteristics, but they should not be interpreted as direct evidence of duration-dependent pharmacological mechanisms.

Subgroup analysis by background HT type was conducted to explore whether the heterogeneous HT comparator influenced the KI findings. The combined estrogen-progestogen subgroup was considered the most clinically relevant restricted network because it represented the most common background HT category. KT + HT showed relatively consistent estimates across the available E + P and E-only subgroups, whereas DK + HT, DZ + HT, and LLH + HT showed favorable estimates in selected HT-type subgroups. However, these findings should be interpreted cautiously because several subgroup networks contained limited numbers of studies, and the analyses cannot establish subgroup-specific superiority or synergistic effects with specific HT backgrounds.

Furthermore, three pre-specified sensitivity analyses for KI—sequentially excluding studies with high risk of bias, small samples, and progestogen-only controls—showed that the direction of effect for several major regimens was generally maintained. The exclusion of progestogen-only controls was particularly relevant because progestogen-only therapy may not be clinically comparable with estrogen-containing HT regimens in all patient contexts. These analyses provide partial support for the stability of the main KI findings but do not eliminate concerns related to sparse nodes, heterogeneous HT backgrounds, limited blinding, and incomplete reporting of baseline symptom severity or TCM syndrome differentiation. Therefore, the results should remain exploratory.

### 4.6 Limitations

Several limitations should be acknowledged. First, the methodological quality of the included trials was limited. Most trials were open-label and lacked placebo or double-dummy designs. For outcome measurement under RoB 2.0, D4 was judged as having some concerns in most trials because adequate blinding or blinded outcome assessment was generally not reported. Subjective outcomes such as KI, MENQOL, and the overall effective rate may therefore be influenced by participants’ or assessors’ awareness of treatment allocation, introducing potential measurement bias. In addition, the psychometric limitations and inconsistently reported versions of KI may reduce the clinical interpretability of both KI-based effect estimates and SUCRA rankings, particularly when very large SMDs were observed.

Second, transitivity and clinical comparability represent major limitations of this NMA. Although HT was used as the common comparator, the background HT regimens were clinically heterogeneous and included estrogen-only therapy, progestogen-only therapy, combined estrogen-progestogen therapy, and tibolone. These regimens may differ in mechanisms, indications, eligible patient populations, and expected effects on symptoms and endocrine outcomes. In addition, treatment duration ranged from 1 to 6 months. Baseline symptom severity, baseline KI/MENQOL scores, TCM syndrome pattern, and explicit syndrome differentiation criteria were inconsistently reported across the included trials, limiting our ability to assess whether these potential effect modifiers were balanced across intervention nodes. Therefore, the transitivity assumption could not be fully verified, and indirect comparisons should be interpreted cautiously. Although KI subgroup analyses by treatment duration and background HT type, including an exploratory E + P-restricted subgroup, and sensitivity analyses excluding progestogen-only controls were performed, these analyses had limited ability to fully address clinical heterogeneity. Several subgroup networks were sparse, and subgroup analyses for MENQOL, endocrine outcomes, adverse reactions, and endometrial thickness were limited by network connectivity and the number of studies per subgroup. Moreover, subgroup analysis by TCM syndrome pattern was not feasible because syndrome differentiation criteria and syndrome-stratified outcomes were inconsistently reported in the original trials.

Third, the evidence network architecture had inherent limitations. Although partial closed loops were formed for several outcomes, the overall network remained predominantly centered on HT. Direct active-comparator evidence between different CCPP + HT regimens was limited, meaning that many inter-regimen comparisons relied heavily on indirect evidence. This structure limits the power of inconsistency testing and increases dependence on the transitivity assumption.

Fourth, the interpretation of sparse-evidence nodes remains uncertain. Nodes such as LLH + HT and WJBF + HT were supported by only two trials. Estimates and exploratory rankings involving these nodes may therefore be susceptible to sparse evidence, small-study effects, and imprecision. In particular, the KI findings for LLH + HT should not be interpreted as a first-choice clinical recommendation. The addition of only a small number of future high-quality trials could substantially change the effect estimates and relative ordering. Therefore, findings for sparse nodes should be regarded as exploratory signals rather than definitive evidence of clinical superiority.

Another limitation concerns product-composition reporting, product-level reporting, and safety assessment. Although complete preparation composition was summarized using accessible product or official information sources, direct composition reporting within the original RCT publications was inconsistent (Supplementary Table S3.3). Product-level information on CCPPs was incompletely reported: manufacturer identity, batch numbers, and regulatory approval information were inconsistently reported, whereas production or expiry dates, quality-control specifications, and marker-metabolite/assay information were not reported (Supplementary Table S3.3). Therefore, potential inter-trial variability in CCPP product quality, processing, storage, excipients, or chemical composition could not be quantitatively assessed. Such variability may have contributed to clinical heterogeneity and could affect the interpretation of both efficacy rankings and safety findings. In addition, although the NMA did not show a significant increase in short-term adverse reactions, adverse-event monitoring was not standardized across trials. Many studies did not clearly report event categories, severity, causality assessment, withdrawals due to adverse events, or follow-up after treatment. In Supplementary Appendix 13, NR for a specific adverse-event category indicates non-reporting rather than absence of the event. Differential reporting of adverse-event categories across CCPP nodes may therefore have introduced reporting bias into the safety analysis. Follow-up durations were also generally short, preventing reliable assessment of rare, delayed, or medium- to long-term safety outcomes. Therefore, the present study cannot establish safety superiority, clinical equivalence, or long-term safety of CCPP + HT compared with standard HT.

Finally, the generalizability of the findings is geographically restricted, as all included trials were conducted in China. Differences in ethnicity, healthcare systems, dietary patterns, CCPP availability, and HT prescribing practices may limit extrapolation to non-Chinese or non-Asian populations. Future multicenter trials with standardized outcome assessment, explicit syndrome differentiation criteria, and longer follow-up are needed to confirm these findings.

### 4.7 Implications for future research

Future research should address the methodological and reporting limitations identified in this NMA. First, future RCTs should follow CONSORT guidance and adopt adequate randomization, allocation concealment, placebo or double-dummy designs where feasible, and intention-to-treat analysis. Product-level reporting and safety monitoring should also be improved by clearly documenting preparation composition, manufacturer identity, batch numbers, quality-control specifications, marker-metabolite/assay information, predefined adverse-event categories, event severity, causality assessment, withdrawals due to adverse events, and follow-up after treatment. Where applicable, plant-derived drugs should be reported with taxonomically validated scientific names, author citations, and family information. Second, outcome reporting should be standardized, with greater emphasis on validated symptom and quality-of-life measures, including KI, MENQOL, and their domain-specific scores where available. Third, future trials should prospectively define TCM syndrome differentiation criteria and report stratified outcomes by syndrome pattern. Where feasible, clinically relevant biomarkers may be incorporated to explore potential effect modifiers, but such analyses should be considered exploratory. Finally, adequately powered direct active-comparator trials and multicenter studies with longer follow-up are needed to generate more reliable comparative and safety evidence.

## 5 Conclusion

This NMA of 73 RCTs suggests that some CCPP + HT combinations may be associated with additional symptom-related and endocrine benefits compared with HT alone in women experiencing perimenopausal symptoms. However, these findings should be interpreted in light of generally low-to-moderate certainty of evidence, the predominance of unblinded trials, limited direct active-comparator evidence, and sparse evidence for several intervention nodes. Several KI effects were very large on the SMD scale and should not be interpreted as direct evidence of large absolute clinical improvement. The present findings, therefore, do not establish reliable comparative superiority among CCPP + HT regimens and should be regarded as preliminary, outcome-specific comparative evidence rather than definitive clinical recommendations. Safety findings were limited to reported short-term adverse-event data and did not establish safety superiority or long-term safety. Further adequately powered, blinded, and direct active-comparator RCTs with standardized outcome assessment and longer follow-up are needed to confirm these findings.

## Data Availability

The original contributions presented in the study are included in the article/Supplementary Material, further inquiries can be directed to the corresponding author.
