# Supplementary material for: Effectiveness and safety of commercial Chinese polyherbal preparations combined with menopausal hormone therapy for perimenopausal symptoms: a network meta-analysis of randomized controlled trials
Source: Front Pharmacol. 2026 Jul 8;17:1861652. doi: 10.3389/fphar.2026.1861652 (PMC13388411; doi:10.3389/fphar.2026.1861652)
Supplement: Supplementary file 2 [file Supplementaryfile1.docx]

**Supplementary Appendix**

**Effectiveness and Safety of Commercial Chinese Polyherbal Preparations Combined with Menopausal Hormone Therapy for Perimenopausal Symptoms: A Network Meta-Analysis of Randomized Controlled Trials**

**Table of Contents**

Appendix 1. PRISMA-NMA checklist

Appendix 2. Search strategy

Appendix 3. Study characteristics, CCPP composition, and product-level reporting

Appendix 4. Risk of bias assessment of randomized clinical trials (RCTs)

Appendix 5. Evaluation of inconsistency, heterogeneity, and transitivity

Appendix 6. Network meta-analysis of various commercial Chinese polyherbal preparations (CCPPs) on secondary endpoints of perimenopausal symptoms

Appendix 7. SUCRA and cumulative probability plots

Appendix 8. League tables of pooled estimates derived from network meta-analysis of 73 RCTs

Appendix 9. Funnel plots for publication bias

Appendix 10. Subgroup analyses for Kupperman Index (KI)

Appendix 11. Sensitivity analyses for KI

Appendix 12. Certainty of evidence assessment (CINeMA)

Appendix 13. Reported adverse reactions and limitations of safety reporting

Appendix 1. PRISMA-NMA checklist

| **Section/Topic** | **Item #** | **Checklist Item** | **Reported in section / appendix** |
| --- | --- | --- | --- |
| **TITLE** |  |  |  |
| Title | 1 | Identify the report as a systematic review *incorporating a network meta-analysis (or related form of meta-analysis).* | Title |
|  |  |  |  |
| **ABSTRACT** |  |  |  |
| Structured summary | 2 | Provide a structured summary including, as applicable:  **Background:** main objectives  **Methods:** data sources; study eligibility criteria, participants, and interventions; study appraisal; and *synthesis methods, such as network meta-analysis.*  **Results:** number of studies and participants identified; summary estimates with corresponding confidence/credible intervals; *treatment rankings may also be discussed. Authors may choose to summarize pairwise comparisons against a chosen treatment included in their analyses for brevity.*  **Discussion/Conclusions:** limitations; conclusions and implications of findings.  **Other:** primary source of funding; systematic review registration number with registry name. | Abstract |
|  |  |  |  |
| **INTRODUCTION** |  |  |  |
| Rationale | 3 | Describe the rationale for the review in the context of what is already known*, including mention of why a network meta-analysis has been conducted.* | Introduction |
| Objectives | 4 | Provide an explicit statement of questions being addressed, with reference to participants, interventions, comparisons, outcomes, and study design (PICOS). | Introduction; Methods: Eligibility Criteria |
|  |  |  |  |
| **METHODS** |  |  |  |
| Protocol and registration | 5 | Indicate whether a review protocol exists and if and where it can be accessed (e.g., Web address); and, if available, provide registration information, including registration number. | Methods |
| Eligibility criteria | 6 | Specify study characteristics (e.g., PICOS, length of follow-up) and report characteristics (e.g., years considered, language, publication status) used as criteria for eligibility, giving rationale. *Clearly describe eligible treatments included in the treatment network, and note whether any have been clustered or merged into the same node (with justification).* | Methods: Eligibility Criteria |
| Information sources | 7 | Describe all information sources (e.g., databases with dates of coverage, contact with study authors to identify additional studies) in the search and date last searched. | Methods: Search Strategy |
| Search | 8 | Present full electronic search strategy for at least one database, including any limits used, such that it could be repeated. | Methods: Search Strategy; Appendix 2 |
| Study selection | 9 | State the process for selecting studies (i.e., screening, eligibility, included in systematic review, and, if applicable, included in the meta-analysis). | Methods: Data Extraction; Results: Literature Search Results |
| Data collection process | 10 | Describe method of data extraction from reports (e.g., piloted forms, independently, in duplicate) and any processes for obtaining and confirming data from investigators. | Methods: Data Extraction |
| Data items | 11 | List and define all variables for which data were sought (e.g., PICOS, funding sources) and any assumptions and simplifications made. | Methods: Data Extraction; Appendix 3 |
| **Geometry of the network** | **S1** | Describe methods used to explore the geometry of the treatment network under study and potential biases related to it. This should include how the evidence base has been graphically summarized for presentation, and what characteristics were compiled and used to describe the evidence base to readers. | Methods: Statistical Analysis; Results: network plots |
| Risk of bias within individual studies | 12 | Describe methods used for assessing risk of bias of individual studies (including specification of whether this was done at the study or outcome level), and how this information is to be used in any data synthesis. | Methods: Risk of Bias Assessment; Appendix 4 |
| Summary measures | 13 | State the principal summary measures (e.g., risk ratio, difference in means). *Also describe the use of additional summary measures assessed, such as treatment rankings and surface under the cumulative ranking curve (SUCRA) values, as well as modified approaches used to present summary findings from meta-analyses.* | Methods: Statistical Analysis |
| Planned methods of analysis | 14 | Describe the methods of handling data and combining results of studies for each network meta-analysis. This should include, but not be limited to:   - *Handling of multi-arm trials;* - *Selection of variance structure;* - *Selection of prior distributions in Bayesian analyses; and* - *Assessment of model fit.* | Methods: Statistical Analysis |
| **Assessment of Inconsistency** | **S2** | Describe the statistical methods used to evaluate the agreement of direct and indirect evidence in the treatment network(s) studied. Describe efforts taken to address its presence when found. | Methods: Statistical Analysis; Appendix 5 |
| Risk of bias across studies | 15 | Specify any assessment of risk of bias that may affect the cumulative evidence (e.g., publication bias, selective reporting within studies). | Methods: Statistical Analysis; Appendices 9, 12 |
| Additional analyses | 16 | Describe methods of additional analyses if done, indicating which were pre-specified. This may include, but not be limited to, the following:   - Sensitivity or subgroup analyses; - Meta-regression analyses; - *Alternative formulations of the treatment network; and* - *Use of alternative prior distributions for Bayesian analyses (if applicable).* | Methods: Statistical Analysis; Methods: Transitivity; Appendices 10, 11 |
| **RESULTS†** |  |  |  |
| Study selection | 17 | Give numbers of studies screened, assessed for eligibility, and included in the review, with reasons for exclusions at each stage, ideally with a flow diagram. | Results: Literature Search Results; Figure 1 |
| **Presentation of network structure** | **S3** | Provide a network graph of the included studies to enable visualization of the geometry of the treatment network. | Results: outcome NMA sections; Figures 3-8; Appendix 6 |
| **Summary of network geometry** | **S4** | Provide a brief overview of characteristics of the treatment network. This may include commentary on the abundance of trials and randomized patients for the different interventions and pairwise comparisons in the network, gaps of evidence in the treatment network, and potential biases reflected by the network structure. | Results: Study Characteristics; Results: Consistency/Heterogeneity |
| Study characteristics | 18 | For each study, present characteristics for which data were extracted (e.g., study size, PICOS, follow-up period) and provide the citations. | Results: Study Characteristics; Appendix 3, Table S3.1 |
| Risk of bias within studies | 19 | Present data on risk of bias of each study and, if available, any outcome level assessment. | Results: Risk of Bias; Appendix 4 |
| Results of individual studies | 20 | For all outcomes considered (benefits or harms), present, for each study: 1) simple summary data for each intervention group, and 2) effect estimates and confidence intervals. *Modified approaches may be needed to deal with information from larger networks.* | Appendices 3, 13 |
| Synthesis of results | 21 | Present results of each meta-analysis done, including confidence/credible intervals. *In larger networks, authors may focus on comparisons versus a particular comparator (e.g. placebo or standard care), with full findings presented in an appendix. League tables and forest plots may be considered to summarize pairwise comparisons.* If additional summary measures were explored (such as treatment rankings), these should also be presented. | Results: outcome NMA sections; Appendices 6-8 |
| **Exploration for inconsistency** | **S5** | Describe results from investigations of inconsistency. This may include such information as measures of model fit to compare consistency and inconsistency models, *P* values from statistical tests, or summary of inconsistency estimates from different parts of the treatment network. | Results: Consistency/Heterogeneity; Appendix 5 |
| Risk of bias across studies | 22 | Present results of any assessment of risk of bias across studies for the evidence base being studied. | Results: Consistency/Heterogeneity; Appendices 9, 12 |
| Results of additional analyses | 23 | Give results of additional analyses, if done (e.g., sensitivity or subgroup analyses, meta-regression analyses*, alternative network geometries studied, alternative choice of prior distributions for Bayesian analyses,* and so forth). | Results: Subgroup and Sensitivity Analyses; Appendices 10, 11 |
| **DISCUSSION** |  |  |  |
| Summary of evidence | 24 | Summarize the main findings, including the strength of evidence for each main outcome; consider their relevance to key groups (e.g., healthcare providers, users, and policy-makers). | Discussion: Primary Findings; Conclusion |
| Limitations | 25 | Discuss limitations at study and outcome level (e.g., risk of bias), and at review level (e.g., incomplete retrieval of identified research, reporting bias). *Comment on the validity of the assumptions, such as transitivity and consistency. Comment on any concerns regarding network geometry (e.g., avoidance of certain comparisons).* | Discussion: Limitations |
| Conclusions | 26 | Provide a general interpretation of the results in the context of other evidence, and implications for future research. | Conclusion |
| **FUNDING** |  |  |  |
| Funding | 27 | Describe sources of funding for the systematic review and other support (e.g., supply of data); role of funders for the systematic review. This should also include information regarding whether funding has been received from manufacturers of treatments in the network and/or whether some of the authors are content experts with professional conflicts of interest that could affect use of treatments in the network. | Funding statement |

Appendix 2. Search strategy

**PubMed**

((("Perimenopause"[MeSH Terms]) OR ("Menopause"[MeSH Terms]) OR ("Climacteric"[MeSH Terms]) OR ("perimenopausal syndrome"[Title/Abstract]) OR ("menopausal syndrome"[Title/Abstract]) OR ("climacteric syndrome"[Title/Abstract]) OR ("menopausal symptoms"[Title/Abstract])) AND (("Drugs, Chinese Herbal"[MeSH Terms]) OR ("Medicine, Chinese Traditional"[MeSH Terms]) OR ("Chinese patent medicine"[Title/Abstract]) OR ("Zhongchengyao"[Title/Abstract]) OR ("Kuntai"[Title/Abstract]) OR ("Dingkun"[Title/Abstract]) OR ("Liuwei Dihuang"[Title/Abstract]) OR ("Dizhen"[Title/Abstract]) OR ("Xiangshao"[Title/Abstract]) OR ("Shugan"[Title/Abstract]) OR ("Wuji Baifeng"[Title/Abstract]) OR ("Guanhuangmu"[Title/Abstract]) OR ("Gengnianshu"[Title/Abstract]) OR ("Linglianhua"[Title/Abstract]) OR ("Zhibai Dihuang"[Title/Abstract]))AND (("Hormone Replacement Therapy"[MeSH Terms]) OR ("Estrogen Replacement Therapy"[MeSH Terms]) OR ("hormone therapy"[Title/Abstract]) OR ("hormone replacement therapy"[Title/Abstract]) OR ("HRT"[Title/Abstract]) OR ("MHT"[Title/Abstract]) OR ("Tibolone"[Title/Abstract]) OR ("Estrogen"[Title/Abstract]) OR ("Estradiol"[Title/Abstract])) AND (("randomized controlled trial"[Publication Type]) OR ("randomized"[Title/Abstract]) OR ("placebo"[Title/Abstract]) OR ("randomly"[Title/Abstract]) OR ("RCT"[Title/Abstract])))

**Embase**

('perimenopause'/exp OR 'menopause'/exp OR 'climacteric'/exp OR 'perimenopausal syndrome':ti,ab OR 'menopausal syndrome':ti,ab OR 'climacteric syndrome':ti,ab OR 'menopausal symptoms':ti,ab) AND ('chinese herbal drug'/exp OR 'chinese patent medicine':ti,ab OR 'zhongchengyao':ti,ab OR 'kuntai':ti,ab OR 'dingkun':ti,ab OR 'liuwei dihuang':ti,ab OR 'dizhen':ti,ab OR 'xiangshao':ti,ab OR 'shugan':ti,ab OR 'wuji baifeng':ti,ab OR 'guanhuangmu':ti,ab OR 'gengnianshu':ti,ab OR 'linglianhua':ti,ab OR 'zhibai dihuang':ti,ab) AND ('hormone substitution'/exp OR 'hormone therapy':ti,ab OR 'hormone replacement therapy':ti,ab OR 'hrt':ti,ab OR 'mht':ti,ab OR 'tibolone':ti,ab OR 'estrogen':ti,ab) AND ('randomized controlled trial'/exp OR 'randomization'/exp OR 'randomly':ti,ab OR 'rct':ti,ab)

**Web of Science (WOS)**

TS=("perimenopause" OR "menopause" OR "climacteric" OR "perimenopausal syndrome" OR "menopausal syndrome" OR "climacteric syndrome" OR "menopausal symptoms") AND TS=("Chinese patent medicine" OR "Zhongchengyao" OR "Kuntai" OR "Dingkun" OR "Liuwei Dihuang" OR "Dizhen" OR "Xiangshao" OR "Shugan" OR "Wuji Baifeng" OR "Guanhuangmu" OR "Gengnianshu" OR "Linglianhua" OR "Zhibai Dihuang") AND TS=("hormone therapy" OR "hormone replacement therapy" OR "HRT" OR "MHT" OR "Tibolone" OR "Estrogen" OR "Estradiol") AND TS=("randomized controlled trial" OR "RCT" OR "random allocation" OR "randomly assigned")

**Cochrane Library**

#1 MeSH descriptor: [Perimenopause] explode all trees

#2 MeSH descriptor: [Menopause] explode all trees

#3 MeSH descriptor: [Climacteric] explode all trees

#4 "perimenopausal syndrome" OR "menopausal syndrome" OR "climacteric syndrome" OR "menopausal symptoms":ti,ab,kw

#5 #1 OR #2 OR #3 OR #4

#6 MeSH descriptor: [Drugs, Chinese Herbal] explode all trees

#7 "Chinese patent medicine" OR "Zhongchengyao":ti,ab,kw

#8 "Kuntai" OR "Dingkun" OR "Liuwei Dihuang" OR "Dizhen" OR "Xiangshao" OR "Shugan" OR "Wuji Baifeng" OR "Guanhuangmu" OR "Gengnianshu" OR "Linglianhua" OR "Zhibai Dihuang"

#9 #6 OR #7 OR #8

#10 MeSH descriptor: [Hormone Replacement Therapy] explode all trees

#11 "hormone therapy" OR "hormone replacement therapy" OR "HRT" OR "MHT" OR "Tibolone" OR "Estrogen":ti,ab,kw

#12 #10 OR #11

#13 #5 AND #9 AND #12

**Chinese Databases (CNKI / Wanfang / VIP)**

（围绝经期综合征 OR 更年期综合征 OR 绝经综合征 OR 围绝经期 OR 更年期）AND（中成药 OR 坤泰 OR 定坤 OR 六味地黄 OR 地贞 OR 香芍 OR 舒肝 OR 乌鸡白凤 OR 关黄母 OR 更年舒 OR 灵莲花 OR 知柏地黄）AND（随机对照试验 OR 随机对照研究 OR RCT OR 随机分组 OR 随机）

Appendix 3. Study characteristics, CCPP composition, and product-level reporting

## Table S3.1. Baseline characteristics of included studies

| **Study** | **Intervention** | **Comparator** | **Course of disease (T, years)** | **Course of disease (C, years)** | **Sample Size (T/C)** | **Age (years) (T/C)** | **Treatment Cycles** | **Outcomes** |
| --- | --- | --- | --- | --- | --- | --- | --- | --- |
| Zhang Z 2019 | SG + Estrogen | Estrogen | 1.8±0.7 | 1.6±0.5 | 43/43 | 46.1±3.2 / 46.8±3.5 | 3 months | ①, ②, ④, ⑥ |
| Liu Y 2020 | SG + Estrogen | Estrogen | — | — | 32/32 | 48.7±3.8 / 46.2±3.3 | 3 months | ①, ② |
| Zhou CQ 2015 | SG+ Estrogen | Estrogen | — | — | 50/50 | — | 2 months | ①, ②, ⑥ |
| An W 2024 | DK + Progestogen | Progestogen | 0.50±0.04 | 0.50±0.05 | 50/50 | 47.79±1.1 / 47.95±1.09 | 2 months | ②, ④, ⑤ |
| Wang YH 2022 | DK + Progestogen | Progestogen | 0.55±0.06 | 0.55±0.06 | 40/40 | 48.48±4.59 / 48.52±4.65 | 6 months | ①, ②, ④, ⑥ |
| Xi YN 2021 | DK + Estrogen + Progestogen | Estrogen + Progestogen | 2.39±0.15 | 2.37±0.18 | 48/48 | 63.85±4.02 / 63.81±4.09 | 3 cycles | ①, ②, ④, ⑤ |
| Zeng F 2023 | DK + Estrogen + Progestogen | Estrogen + Progestogen | — | — | 34/34 | 53.39±5.87 / 53.21±5.82 | 3 months | ①, ④, ⑥ |
| Wang M 2017 | DK + Estrogen | Estrogen | 2.0±0.8 | 2.2±0.4 | 41/41 | 51.5±4.1 / 50.5±4.7 | 3 months | ②, ③, ④, ⑥ |
| Chen SM 2020 | DK + Tibolone | Tibolone | 1.35±0.43 | 1.51±0.48 | 41/41 | 47.37±2.20 / 47.85±2.79 | 3 months | ①, ②, ④, ⑥ |
| Ju LJ 2023 | DK + Estrogen + Progestogen | Estrogen + Progestogen | 2.41±0.78 | 2.34±0.51 | 51/51 | 51.52±4.35 / 50.47±4.62 | 3 months | ①, ②, ④ |
| Wang Y 2024 | DK + Estrogen + Progestogen | Estrogen + Progestogen | 1.35±0.27 | 1.33±0.18 | 50/50 | 49.23±2.52 / 49.35±2.61 | 3 months | ② |
| Li LJ 2022 | DK + Estrogen + Progestogen | Estrogen + Progestogen | 3.50±0.79 | 3.22±0.89 | 15/15 | 51.34±4.54 / 51.72±4.90 | 3 months | ①, ⑥ |
| Shi YR 2019 | DK + Estrogen + Progestogen | Estrogen + Progestogen | 1.65±0.75 | 1.63±0.71 | 44/44 | 48.15±2.21 / 48.33±2.91 | 3 months | ②, ④, ⑥ |
| Zhao HX 2024 | DK + Tibolone | Tibolone | 2.56±0.64 | 2.68±0.92 | 49/49 | 51.41±2.57 / 51.23±2.48 | 3 months | ②, ④, ⑤, ⑥ |
| Wu J 2025 | DK + Estrogen + Progestogen | Estrogen + Progestogen | 1.93±0.47 | 1.87±0.55 | 49/49 | 49.58±4.31 / 49.31±4.58 | 3 months | ②, ④ |
| Zhu XQ 2023 | DK + Estrogen + Progestogen | Estrogen + Progestogen | 1.86±0.35 | 1.87±0.34 | 50/50 | 48.01±2.08 / 48.14±2.04 | 3 months | ② |
| Ren W 2019 | LWDH + Estrogen + Progestogen | Estrogen + Progestogen | 2.4±0.7 | 2.3±0.5 | 131/131 | 49.3±4.4 / 48.8±4.1 | 6 months | ①, ②, ③, ④, ⑥ |
| Wu XJ 2022 | LWDH + Estrogen + Progestogen | Estrogen + Progestogen | 1.38±0.23 | 1.32±0.28 | 30/30 | 47.19±3.52 / 47.14±3.56 | 3 cycles | ②, ④, ⑥ |
| Fu XN 2021 | LWDH + Estrogen + Progestogen | Estrogen + Progestogen | 0.98±0.09 | 0.99±0.09 | 39/39 | 47.4±3.0 / 47.1±2.6 | 3 months | ①, ②, ④, ⑤, ⑥ |
| Chen L 2013 | LWDH + Progestogen | Progestogen | 3.75±3.02 | 3.75±3.02 | 340/343 | 49.67±5.21 / 49.67±5.21 | 3 cycles | ①, ②, ④, ⑥ |
| Wang XH 2023 | LWDH + Estrogen | Estrogen | 1.2±0.3 | 1.3±0.4 | 56/56 | 48.3±2.6 / 48.4±2.8 | 3 months | ②, ④, ⑥ |
| Chen Y 2024 | LWDH + Estrogen + Progestogen | Estrogen + Progestogen | 2.74±0.36 | 2.79±0.41 | 35/35 | 49.27±3.44 / 49.13±3.25 | 6 months | ②, ④, ⑤ |
| Chen Y 2022 | LWDH + Estrogen + Progestogen | Estrogen + Progestogen | — | — | 40/40 | 50.32±3.18 / 50.29±3.21 | 3 months | ①, ②, ④, ⑤ |
| Wu YL 2023 | LWDH + Estrogen + Progestogen | Estrogen + Progestogen | 0.86±0.10 | 0.85±0.12 | 40/40 | 48.32±2.57 / 48.15±2.36 | 3 cycles | ④, ⑤ |
| Yuan Y 2015 | DZ + Tibolone | Tibolone | 2.17±0.37 | 2.14±0.36 | 60/60 | 48.55±2.71 / 45.58±2.35 | 3 cycles | ①, ② |
| Wang Q 2020 | DZ + Tibolone | Tibolone | 1.28±0.14 | 1.27±0.12 | 43/43 | 49.53±6.28 / 49.61±5.32 | 3 months | ①, ②, ④, ⑥ |
| Wei CK 2020 | DZ + Tibolone | Tibolone | Mean 3.9 | Mean 3.9 | 50/50 | Mean 49.5 / Mean 49.5 | 3 months | ②, ④ |
| Kong XJ 2025 | XS + Progestogen | Progestogen | 0.43±0.07 | 0.44±0.06 | 103/102 | 47.48±1.3 / 47.55±1.23 | 3 months | ②, ④, ⑥ |
| Fang Q 2024 | XS + Estrogen + Progestogen | Estrogen + Progestogen | 3.62±1.08 | 3.57±1.19 | 45/45 | 50.27±4.89 / 50.45±4.04 | 3 cycles | ①, ②, ③, ④, ⑥ |
| Geng YN 2021 | XS + Estrogen + Progestogen | Estrogen + Progestogen | 1.12±0.29 | 1.06±0.27 | 59/58 | 49.91±2.71 / 49.83±2.86 | 2 cycles | ①, ②, ④, ⑥ |
| Chen YL 2019 | XS + Estrogen + Progestogen | Estrogen + Progestogen | 0.51±0.17 | 0.53±0.18 | 40/40 | 49.01±2.82 / 48.11±2.87 | 2 cycles | ①, ②, ④ |
| Wu YQ 2014 | XS+ Estrogen + Progestogen | Estrogen + Progestogen | 0.53±0.18 | 0.48±0.29 | 30/30 | 48.73±3.23 / 49.02±2.95 | 2 months | ①, ②, ④, ⑤, ⑥ |
| Li WL 2022 | XS+ Estrogen | KT+ Estrogen | 1.7±0.7 | 1.6±0.8 | 198/198 | 50.5±2.4 / 50.4±2.3 | 4 months | ②, ④ |
| Cai ZM 2016 | XS+ Estrogen + Progestogen | Estrogen + Progestogen | 1.11±0.48 | 1.08±0.43 | 42/42 | 50.9±5.2 / 51.3±4.8 | 3 months | ①, ④ |
| Zhang Y 2025 | XS + Tibolone | Tibolone | 4.17±0.36 | 4.23±0.32 | 60/60 | 51.09±1.13 / 51.03±1.12 | 1 month | ②, ④ |
| Cuan MR 2020 | KT + Progestogen | Progestogen | 1.72±0.85 | 1.8±0.89 | 40/40 | 54.63±5.27 / 54.75±5.35 | 3 months | ①, ②, ④, ⑤, ⑥ |
| Zhou YY 2023 | KT + Estrogen + Progestogen | Estrogen + Progestogen | 1.23±0.55 | 1.21±0.58 | 30/30 | 52.36±3.31 / 52.15±3.47 | 3 cycles | ②, ③, ④ |
| Li XH 2021 | KT + Estrogen + Progestogen | Estrogen + Progestogen | 1.24±0.62 | 1.27±0.65 | 73/73 | 47.98±4.92 / 48.41±4.77 | 3 cycles | ①, ②, ③, ④, ⑥ |
| Wang HW 2025 | KT + Estrogen + Progestogen | Estrogen + Progestogen | 1.13±0.17 | 1.14±0.17 | 60/60 | 51.29±1.01 / 51.05±1.09 | 3 cycles | ①, ②, ④, ⑤, ⑥ |
| Ding KQ 2018 | KT + Estrogen | Estrogen | 1.5±0.3 | 1.4±0.7 | 80/80 | 48.9±3.8 / 48.0±2.9 | 3 cycles | ①, ②, ④, ⑥ |
| Ding YL 2025 | KT + Estrogen | Estrogen | 0.56±0.14 | 0.56±0.15 | 55/54 | 51.07±2.39 / 50.83±2.46 | 3 months | ①, ②, ④ |
| Hou JX 2017 | KT + Estrogen | Estrogen | 1.3±0.6 | 1.5±0.5 | 42/40 | 46.37±7.81 / 48.13±5.18 | 3 months | ①, ②, ④, ⑤, ⑥ |
| Liu CF 2020 | KT + Estrogen | Estrogen | 0.49±0.17 | 0.45±0.18 | 50/50 | 52.68±3.01 / 53.21±2.36 | 3 months | ①, ③, ④, ⑥ |
| Li SS 2023 | KT + Estrogen + Progestogen | Estrogen + Progestogen | 1.07±0.27 | 1.10±0.26 | 45/45 | 46.86±1.99 / 47.93±2.01 | 3 cycles | ①, ②, ④, ⑥ |
| Shan D 2019 | KT + Estrogen | Estrogen | 1.48±0.52 | 1.56±0.46 | 43/43 | 54.63±1.37 / 51.46±2.44 | 1 month | ③, ⑤, ⑥ |
| Ji Y 2016 | KT + Estrogen | Estrogen | 1.7±0.4 | 1.6±0.8 | 42/42 | 50.4±4.2 / 50.1±4.1 | 3 months | ①, ②, ④, ⑤, ⑥ |
| Gong CL 2016 | KT + Tibolone | Tibolone | 2.3±0.68 | 2.5±0.53 | 59/59 | 49.2±3.2 / 49.7±2.8 | 3 months | ①, ②, ④ |
| Yin H 2025 | KT + Estrogen + Progestogen | Estrogen + Progestogen | 1.97±0.41 | 1.74±0.41 | 51/51 | 51.56±1.35 / 50.13±2.09 | 3 cycles | ①, ②, ④, ⑥ |
| Liu WW 2025 | KT + Estrogen + Progestogen | Estrogen + Progestogen | 1.42±0.20 | 1.41±0.20 | 32/33 | 50.09±1.95 / 50.06±1.99 | 3 months | ①, ④, ⑥ |
| Yang B 2017 | KT + Estrogen + Progestogen | Estrogen + Progestogen | 1.27±0.56 | 1.21±0.61 | 53/53 | 50.4±1.0 / 50.2±1.3 | 3 months | ①, ②, ④ |
| OuYang H 2022 | KT + Estrogen + Progestogen | Estrogen + Progestogen | 1.92±0.41 | 1.96±0.35 | 45/45 | 49.86±1.41 / 49.34±1.28 | 3 cycles | ②, ③, ④, ⑥ |
| Shi LX 2022 | KT + Estrogen + Progestogen | Estrogen + Progestogen | 1.20±0.11 | 1.21±0.11 | 41/41 | 51.65±1.21 / 51.73±1.25 | 3 months | ②, ④, ⑤, ⑥ |
| Qi BB 2025 | KT + Estrogen | Estrogen | 0.58±0.18 | 0.57±0.17 | 41/41 | 49.83±2.25 / 49.52±2.34 | 3 cycles | ②, ④, ⑤, ⑥ |
| Yu JX 2025 | KT + Estrogen + Progestogen | Estrogen + Progestogen | 1.15±0.09 | 1.20±0.10 | 30/30 | 46.98±5.03 / 47.61±5.12 | 3 months | ①, ④, ⑤, ⑥ |
| Yan XH 2019 | KT + Tibolone | Tibolone | 1.6±0.3 | 1.6±0.2 | 60/60 | 47.2±2.1 / 46.6±3.1 | 2 months | ①, ③, ④ |
| Chen LM 2024 | KT + Estrogen + Progestogen | Estrogen + Progestogen | 2.17±0.82 | 2.19±0.84 | 35/35 | 48.96±3.44 / 49.02±3.48 | 3 cycles | ①, ②, ④, ⑥ |
| Chen XF 2022 | KT + Estrogen + Progestogen | Estrogen + Progestogen | 1.49±0.44 | 1.42±0.42 | 100/100 | 46.88±6.52 / 47.06±6.44 | 3 months | ①, ②, ④, ⑤, ⑥ |
| Zhao AM 2013 | SG + Estrogen | Estrogen | 2.0±0.5 | 2.0±0.5 | 53/42 | 48±3.4 / 48±3.4 | 2 months | ②, ⑥ |
| Fan X 2018 | WJBF + Estrogen | Estrogen | 1.69±0.39 | 1.62±0.32 | 41/40 | 49.27±4.34 / 49.53±4.92 | 3 months | ①, ② |
| Li SM 2019 | WJBF + Estrogen + Progestogen | Estrogen + Progestogen | 0.58±0.19 | 0.57±0.18 | 66/65 | 46.88±1.62 / 46.74±1.75 | 3 cycles | ①, ④, ⑥ |
| Wang Y 2023 | GHM + Tibolone | Tibolone | 1.52±0.18 | 1.49±0.19 | 60/60 | 49.45±5.12 / 50.13±4.79 | 2 months | ①, ②, ④, ⑤, ⑥ |
| Zhao J 2026 | GHM + Tibolone | Tibolone | 2.13±0.26 | 2.08±0.24 | 32/31 | 51.00±2.91 / 50.92±2.69 | 3 months | ①, ④, ⑥ |
| Fei Y 2024 | GHM+ Estrogen + Progestogen | Estrogen + Progestogen | 2.33±0.55 | 2.19±0.52 | 93/92 | 49.23±3.75 / 48.65±3.24 | 2 months | ①, ②, ③, ④, ⑤, ⑥ |
| Chen JM 2023 | GHM + Tibolone | Tibolone | 1.44±0.27 | 1.37±0.22 | 50/50 | 48.91±2.16 / 49.25±2.31 | 2 months | ②, ④, ⑥ |
| Yang QX 2019 | GNS + Estrogen | Estrogen | 2.2±0.8 | 2.0±0.6 | 39/39 | 50.1±4.2 / 50.7±4.7 | 3 months | ①, ②, ③, ④, ⑥ |
| Liu QH 2016 | GNS + Tibolone | Tibolone | 2.7±2.4 | 2.5±2.2 | 50/51 | 50.9±6.2 / 51.5±6.0 | 3 months | ①, ②, ④ |
| Liao Y 2021 | GNS + Tibolone | Tibolone | 2.15±0.63 | 2.17±0.65 | 51/51 | 51.24±4.15 / 51.31±4.19 | 3 months | ②, ④, ⑥ |
| Chen MM 2020 | GNS + Tibolone | Tibolone | 2.05±0.57 | 2.03±0.52 | 50/50 | 52.25±2.27 / 52.22±2.26 | 3 months | ②, ④, ⑥ |
| Liu WY 2022 | LLH + Estrogen + Progestogen | Estrogen + Progestogen | 1.47±0.48 | 1.52±0.57 | 56/56 | 50.45±2.62 / 50.13±2.54 | 3 months | ①, ②, ④ |
| Lu Y 2018 | LLH+ Estrogen | KT+ Estrogen | 5.80±4.69 | 5.59±4.77 | 118/116 | 50.60±3.10 / 50.59±2.89 | 3 months | ①, ②, ④, ⑥ |
| Xiang D 2025 | ZBDH + Tibolone | Tibolone | 1.91±0.99 | 1.94±1.09 | 30/30 | 52.33±2.97 / 52.80±3.67 | 6 months | ①, ②, ④, ⑥ |
| Yan JH 2015 | ZBDH + Estrogen | Estrogen | — | — | 107/106 | — | 6 cycles | ②, ⑤ |
| Tang JJ 2003 | ZBDH + Estrogen + Progestogen | Estrogen + Progestogen | Mean 1.48 | Mean 1.29 | 35/35 | Mean 47.37 / Mean 46.35 | 2 months | ①, ④, ⑤ |

Abbreviations: SG (Shugan Granule); LWDH (Liuwei Dihuang Pill); DZ (Dizhen Granule); XS (Xiangshao Granule); KT (Kuntai Capsule); DK (Dingkun Pill); WJBF (Wuji Baifeng Pill); GHM (Guanhuangmu Granule); GNS (Gengnianshu Tablet); LLH (Linglianhua Granule); ZBDH (Zhibai Dihuang Pill); Tibolone (Tibolone therapy); Estrogen (Estrogen therapy); Progestogen (Progestogen therapy); T (treatment group); C (control group); — (not reported).

*Outcome measures:* *① KI; ② Overall effective rate; ③ MENQOL (Menopause-Specific Quality of Life); ④ Sex hormones: Estradiol(E2), Follicle-Stimulating Hormone(FSH), Luteinizing Hormone(LH); ⑤ Endometrial thickness; ⑥ Adverse reactions.*

## Table S3.2. Botanical and other drugs included in the 11 commercial Chinese polyherbal preparations, with taxonomic validation of plant-derived drugs

| **CCPP** | **Drug included in the preparation** | **Original Chinese drug/material name** | **Origin category** | **Accepted scientific name with author citation** | **Family** | **Medicinal material / plant part** | **Taxonomic validation source** | **Notes** |
| --- | --- | --- | --- | --- | --- | --- | --- | --- |
| Kuntai Capsule | Rehmanniae Radix | 地黄 | Botanical | *Rehmannia* *glutinosa* (Gaertn.) Libosch. ex DC. | Orobanchaceae | Root tuber / Radix | POWO / MPNS | Plant-derived drug |
| Kuntai Capsule | Coptidis Rhizoma | 黄连 | Botanical | *Coptis* *chinensis* Franch. | Ranunculaceae | Rhizome / Rhizoma | POWO / MPNS | Plant-derived drug |
| Kuntai Capsule | Paeoniae Radix Alba | 白芍 | Botanical | *Paeonia* *lactiflora* Pall. | Paeoniaceae | Root / Radix | POWO / MPNS | Plant-derived drug |
| Kuntai Capsule | Scutellariae Radix | 黄芩 | Botanical | *Scutellaria* *baicalensis* Georgi | Lamiaceae | Root / Radix | POWO / MPNS | Plant-derived drug |
| Kuntai Capsule | Poria | 茯苓 | Fungal-derived | *Wolfiporia* *cocos* (F.A.Wolf) Ryvarden & Gilb. | Polyporaceae | Sclerotium | Index Fungorum / MycoBank | Fungal-derived medicinal material; botanical validation not applicable |
| Kuntai Capsule | Asini Corii Colla | 阿胶 | Animal-derived | *Equus asinus* Linnaeus, 1758 | Equidae | Donkey-hide gelatin / Colla | GBIF / ITIS | Animal-derived medicinal material; valid zoological source name and family added where identifiable. |
| Dingkun Pill | Ginseng Radix Rubra | 红参 | Botanical | *Panax* *ginseng* C.A.Mey. | Araliaceae | Steamed and dried root / Radix Rubra | POWO / MPNS | Plant-derived drug |
| Dingkun Pill | Cervi Cornu Pantotrichum | 鹿茸 | Animal-derived | *Cervus nippon* Temminck, 1838 / *Cervus elaphus* Linnaeus, 1758 | Cervidae | Young pilose antler | GBIF / ITIS | Multi-source animal-derived medicinal material; recognized zoological source species and family information added where identifiable. |
| Dingkun Pill | Croci Stigma | 西红花 | Botanical | *Crocus* *sativus* L. | Iridaceae | Stigma / Stigma | POWO / MPNS | Plant-derived drug |
| Dingkun Pill | Notoginseng Radix et Rhizoma | 三七 | Botanical | *Panax* *notoginseng* (Burkill) F.H.Chen | Araliaceae | Root and rhizome / Radix et Rhizoma | POWO / MPNS | Plant-derived drug |
| Dingkun Pill | Paeoniae Radix Alba | 白芍 | Botanical | *Paeonia* *lactiflora* Pall. | Paeoniaceae | Root / Radix | POWO / MPNS | Plant-derived drug |
| Dingkun Pill | Rehmanniae Radix Praeparata | 熟地黄 | Botanical | *Rehmannia* *glutinosa* (Gaertn.) Libosch. ex DC. | Orobanchaceae | Prepared root tuber / Radix Praeparata | POWO / MPNS | Plant-derived drug |
| Dingkun Pill | Angelicae Sinensis Radix | 当归 | Botanical | *Angelica* *sinensis* (Oliv.) Diels | Apiaceae | Root / Radix | POWO / MPNS | Plant-derived drug |
| Dingkun Pill | Atractylodis Macrocephalae Rhizoma | 白术 | Botanical | *Atractylodes* *macrocephala* Koidz. | Asteraceae | Rhizome / Rhizoma | POWO / MPNS | Plant-derived drug |
| Dingkun Pill | Lycii Fructus | 枸杞子 | Botanical | *Lycium* *barbarum* L. | Solanaceae | Fruit / Fructus | POWO / MPNS | Plant-derived drug |
| Dingkun Pill | Scutellariae Radix | 黄芩 | Botanical | *Scutellaria* *baicalensis* Georgi | Lamiaceae | Root / Radix | POWO / MPNS | Plant-derived drug |
| Dingkun Pill | Cyperi Rhizoma | 香附 | Botanical | *Cyperus* *rotundus* L. | Cyperaceae | Rhizome / Rhizoma | POWO / MPNS | Plant-derived drug |
| Dingkun Pill | Leonuri Fructus | 茺蔚子 | Botanical | *Leonurus* *japonicus* Houtt. | Lamiaceae | Fruit / Fructus | POWO / MPNS | Plant-derived drug |
| Dingkun Pill | Chuanxiong Rhizoma | 川芎 | Botanical | *Conioselinum* *anthriscoides* (H.Boissieu) Pimenov & Kljuykov | Apiaceae | Rhizome / Rhizoma | POWO / MPNS | Plant-derived drug |
| Dingkun Pill | Cervi Cornu Degelatinatum | 鹿角霜 | Animal-derived | *Cervus nippon* Temminck, 1838 / *Cervus elaphus* Linnaeus, 1758 | Cervidae | Degelatinized deer antler / Cornu Degelatinatum | GBIF / ITIS | Multi-source animal-derived medicinal material; recognized zoological source species and family information added where identifiable. |
| Dingkun Pill | Asini Corii Colla | 阿胶 | Animal-derived | *Equus asinus* Linnaeus, 1758 | Equidae | Donkey-hide gelatin / Colla | GBIF / ITIS | Animal-derived medicinal material; valid zoological source name and family added where identifiable. |
| Dingkun Pill | Corydalis Rhizoma | 延胡索 | Botanical | *Corydalis* *yanhusuo* W.T.Wang ex Z.Y.Su & C.Y.Wu | Papaveraceae | Rhizome / Rhizoma | POWO / MPNS | Plant-derived drug |
| Dingkun Pill | Poria | 茯苓 | Fungal-derived | *Wolfiporia* *cocos* (F.A.Wolf) Ryvarden & Gilb. | Polyporaceae | Sclerotium | Index Fungorum / MycoBank | Fungal-derived medicinal material; botanical validation not applicable |
| Dingkun Pill | Leonuri Herba | 益母草 | Botanical | *Leonurus* *japonicus* Houtt. | Lamiaceae | Aerial part / Herba | POWO / MPNS | Plant-derived drug |
| Dingkun Pill | Trogopterori Faeces | 五灵脂 | Animal-derived | *Trogopterus xanthipes* Milne-Edwards, 1867 | Sciuridae | Processed faeces of flying squirrel-related medicinal material | GBIF / ITIS | Animal-derived medicinal material; valid zoological source name and family added where identifiable. |
| Dingkun Pill | Bupleuri Radix | 柴胡 | Botanical | *Bupleurum* *chinense* DC. | Apiaceae | Root / Radix | POWO / MPNS | Multi-source medicinal material in pharmacopeial use; species entry standardized to one common accepted source |
| Dingkun Pill | Amomi Fructus | 砂仁 | Botanical | *Amomum* *villosum* Lour. | Zingiberaceae | Fruit / Fructus | POWO / MPNS | Plant-derived drug |
| Dingkun Pill | Asari Radix et Rhizoma | 细辛 | Botanical | *Asarum* *heterotropoides* F.Schmidt | Aristolochiaceae | Root and rhizome / Radix et Rhizoma | POWO / MPNS | Multi-source medicinal material in pharmacopeial use; species entry standardized to one common accepted source |
| Dingkun Pill | Cinnamomi Cortex | 肉桂 | Botanical | *Cinnamomum* *cassia* (L.) J.Presl | Lauraceae | Bark / Cortex | POWO / MPNS | Plant-derived drug |
| Dingkun Pill | Linderae Radix | 乌药 | Botanical | *Lindera* *aggregata* (Sims) Kosterm. | Lauraceae | Root / Radix | POWO / MPNS | Plant-derived drug |
| Dingkun Pill | Compound Dian Jixueteng Extract | 复方滇鸡血藤膏 | Compound processed botanical material | Not fully identifiable from accessible product information | Not fully identifiable | Processed extract / Extractum | Not applicable / insufficiently specified | Reported as a compound extract; exact botanical subcomposition not consistently identifiable |
| Dingkun Pill | Glycyrrhizae Radix et Rhizoma | 甘草 | Botanical | *Glycyrrhiza* *uralensis* Fisch. ex DC. | Fabaceae | Root and rhizome / Radix et Rhizoma | POWO / MPNS | Multi-source medicinal material in pharmacopeial use; species entry standardized to one common accepted source |
| Dingkun Pill | Cyathulae Radix | 川牛膝 | Botanical | *Cyathula* *officinalis* K.C.Kuan | Amaranthaceae | Root / Radix | POWO / MPNS | Plant-derived drug |
| Dingkun Pill | Eucommiae Cortex | 杜仲 | Botanical | *Eucommia* *ulmoides* Oliv. | Eucommiaceae | Bark / Cortex | POWO / MPNS | Plant-derived drug |
| Dingkun Pill | Zingiberis Rhizoma | 干姜 | Botanical | *Zingiber* *officinale* Roscoe | Zingiberaceae | Rhizome / Rhizoma | POWO / MPNS | Plant-derived drug |
| Dingkun Pill | Fuligo Plantae | 百草霜 | Other non-botanical / processed soot material | Not applicable | Not applicable | Processed soot / Fuligo | Not applicable | Non-botanical material; botanical taxonomic validation not applicable |
| Dingkun Pill | Aquilariae Lignum Resinatum | 沉香 | Botanical | *Aquilaria* *sinensis* (Lour.) Spreng. | Thymelaeaceae | Resin-containing wood / Lignum Resinatum | POWO / MPNS | Multi-source medicinal material in pharmacopeial use; species entry standardized to one common accepted source |
| Dingkun Pill | Mel | 蜂蜜 | Excipient | Not applicable | Not applicable | Honey / excipient | Not applicable | Excipient; not counted as a botanical or active medicinal drug |
| Liuwei Dihuang Pill | Rehmanniae Radix Praeparata | 熟地黄 | Botanical | *Rehmannia* *glutinosa* (Gaertn.) Libosch. ex DC. | Orobanchaceae | Prepared root tuber / Radix Praeparata | POWO / MPNS | Plant-derived drug |
| Liuwei Dihuang Pill | Corni Fructus | 山茱萸 | Botanical | *Cornus* *officinalis* Siebold & Zucc. | Cornaceae | Fruit / Fructus | POWO / MPNS | Plant-derived drug |
| Liuwei Dihuang Pill | Dioscoreae Rhizoma | 山药 | Botanical | *Dioscorea* *polystachya* Turcz. | Dioscoreaceae | Rhizome / Rhizoma | POWO / MPNS | Plant-derived drug |
| Liuwei Dihuang Pill | Poria | 茯苓 | Fungal-derived | *Wolfiporia* *cocos* (F.A.Wolf) Ryvarden & Gilb. | Polyporaceae | Sclerotium | Index Fungorum / MycoBank | Fungal-derived medicinal material; botanical validation not applicable |
| Liuwei Dihuang Pill | Alismatis Rhizoma | 泽泻 | Botanical | *Alisma* *plantago-aquatica* subsp. *orientale* (Sam.) Sam. | Alismataceae | Rhizome / Rhizoma | POWO / WFO / MPNS | Plant-derived drug |
| Liuwei Dihuang Pill | Moutan Cortex | 牡丹皮 | Botanical | *Paeonia* × *suffruticosa* Andrews | Paeoniaceae | Root bark / Cortex | POWO / MPNS | Plant-derived drug |
| Dizhen Granule | Lycii Cortex | 地骨皮 | Botanical | *Lycium* *chinense* Mill. / *Lycium* *barbarum* L. | Solanaceae | Root bark / Cortex | POWO / MPNS | Multi-source medicinal material in pharmacopeial use; species entry standardized to one common accepted source |
| Dizhen Granule | Ligustri Lucidi Fructus | 女贞子 | Botanical | *Ligustrum* *lucidum* W.T.Aiton | Oleaceae | Fruit / Fructus | POWO / MPNS | Plant-derived drug |
| Dizhen Granule | Ecliptae Herba | 墨旱莲 | Botanical | *Eclipta* *prostrata* (L.) L. | Asteraceae | Aerial part / Herba | POWO / MPNS | Plant-derived drug |
| Dizhen Granule | Schisandrae Chinensis Fructus | 五味子 | Botanical | *Schisandra* *chinensis* (Turcz.) Baill. | Schisandraceae | Fruit / Fructus | POWO / MPNS | Plant-derived drug |
| Dizhen Granule | Astragali Complanati Semen | 沙苑子 | Botanical | *Astragalus* *complanatus* R.Br. | Fabaceae | Seed / Semen | POWO / MPNS | Plant-derived drug |
| Dizhen Granule | Albiziae Cortex | 合欢皮 | Botanical | *Albizia* *julibrissin* Durazz. | Fabaceae | Bark / Cortex | POWO / MPNS | Plant-derived drug |
| Dizhen Granule | Glycyrrhizae Radix et Rhizoma | 甘草 | Botanical | *Glycyrrhiza* *uralensis* Fisch. ex DC. | Fabaceae | Root and rhizome / Radix et Rhizoma | POWO / MPNS | Multi-source medicinal material in pharmacopeial use; species entry standardized to one common accepted source |
| Dizhen Granule | Curcumae Radix | 郁金 | Botanical | *Curcuma* *longa* L. | Zingiberaceae | Tuberous root / Radix | POWO / MPNS | Multi-source medicinal material in pharmacopeial use; species entry standardized to one common accepted source |
| Xiangshao Granule | Paeoniae Radix Alba | 白芍 | Botanical | *Paeonia* *lactiflora* Pall. | Paeoniaceae | Root / Radix | POWO / MPNS | Plant-derived drug |
| Xiangshao Granule | Cyperi Rhizoma | 香附 | Botanical | *Cyperus* *rotundus* L. | Cyperaceae | Rhizome / Rhizoma | POWO / MPNS | Plant-derived drug |
| Xiangshao Granule | Toosendan Fructus | 川楝子 | Botanical | *Melia* *toosendan* Siebold & Zucc. | Meliaceae | Fruit / Fructus | POWO / MPNS | Plant-derived drug |
| Xiangshao Granule | Bupleuri Radix | 柴胡 | Botanical | *Bupleurum* *chinense* DC. | Apiaceae | Root / Radix | POWO / MPNS | Multi-source medicinal material in pharmacopeial use; species entry standardized to one common accepted source |
| Xiangshao Granule | Chuanxiong Rhizoma | 川芎 | Botanical | *Conioselinum* *anthriscoides* (H.Boissieu) Pimenov & Kljuykov | Apiaceae | Rhizome / Rhizoma | POWO / MPNS | Plant-derived drug |
| Xiangshao Granule | Aurantii Fructus | 枳壳 | Botanical | *Citrus* × *aurantium* L. | Rutaceae | Mature fruit / Fructus | POWO / MPNS | Plant-derived drug |
| Xiangshao Granule | Pinelliae Rhizoma | 半夏 | Botanical | *Pinellia* *ternata* (Thunb.) Makino | Araceae | Tuber / Rhizoma | POWO / MPNS | Plant-derived drug |
| Xiangshao Granule | Amomi Fructus Rotundus | 豆蔻 | Botanical | *Amomum* *kravanh* Pierre ex Gagnep. | Zingiberaceae | Fruit / Fructus | POWO / MPNS | Plant-derived drug |
| Xiangshao Granule | Aucklandiae Radix | 木香 | Botanical | *Dolomiaea* *costus* (Falc.) Kasana & A.K.Pandey | Asteraceae | Root / Radix | POWO / MPNS | Plant-derived drug |
| Xiangshao Granule | Glycyrrhizae Radix et Rhizoma | 甘草 | Botanical | *Glycyrrhiza* *uralensis* Fisch. ex DC. | Fabaceae | Root and rhizome / Radix et Rhizoma | POWO / MPNS | Multi-source medicinal material in pharmacopeial use; species entry standardized to one common accepted source |
| Shugan Granule | Angelicae Sinensis Radix | 当归 | Botanical | *Angelica* *sinensis* (Oliv.) Diels | Apiaceae | Root / Radix | POWO / MPNS | Plant-derived drug |
| Shugan Granule | Paeoniae Radix Alba | 白芍 | Botanical | *Paeonia* *lactiflora* Pall. | Paeoniaceae | Root / Radix | POWO / MPNS | Plant-derived drug |
| Shugan Granule | Atractylodis Macrocephalae Rhizoma | 白术 | Botanical | *Atractylodes* *macrocephala* Koidz. | Asteraceae | Rhizome / Rhizoma | POWO / MPNS | Plant-derived drug |
| Shugan Granule | Cyperi Rhizoma | 香附 | Botanical | *Cyperus* *rotundus* L. | Cyperaceae | Rhizome / Rhizoma | POWO / MPNS | Plant-derived drug |
| Shugan Granule | Bupleuri Radix | 柴胡 | Botanical | *Bupleurum* *chinense* DC. | Apiaceae | Root / Radix | POWO / MPNS | Multi-source medicinal material in pharmacopeial use; species entry standardized to one common accepted source |
| Shugan Granule | Poria | 茯苓 | Fungal-derived | *Wolfiporia* *cocos* (F.A.Wolf) Ryvarden & Gilb. | Polyporaceae | Sclerotium | Index Fungorum / MycoBank | Fungal-derived medicinal material; botanical validation not applicable |
| Shugan Granule | Menthae Haplocalycis Herba | 薄荷 | Botanical | *Mentha* *canadensis* L. | Lamiaceae | Aerial part / Herba | POWO / MPNS | Plant-derived drug |
| Shugan Granule | Gardeniae Fructus | 栀子 | Botanical | *Gardenia* *jasminoides* J.Ellis | Rubiaceae | Fruit / Fructus | POWO / MPNS | Plant-derived drug |
| Shugan Granule | Moutan Cortex | 牡丹皮 | Botanical | *Paeonia* × *suffruticosa* Andrews | Paeoniaceae | Root bark / Cortex | POWO / MPNS | Plant-derived drug |
| Shugan Granule | Glycyrrhizae Radix et Rhizoma | 甘草 | Botanical | *Glycyrrhiza* *uralensis* Fisch. ex DC. | Fabaceae | Root and rhizome / Radix et Rhizoma | POWO / MPNS | Multi-source medicinal material in pharmacopeial use; species entry standardized to one common accepted source |
| Shugan Granule | Sucrose | 蔗糖 | Excipient | Not applicable | Not applicable | Excipient | Not applicable | Excipient; not counted as a botanical or active medicinal drug |
| Wuji Baifeng Pill | Gallus Domesticus | 乌鸡 | Animal-derived | *Gallus gallus domesticus* (Linnaeus, 1758) | Phasianidae | Processed black-bone chicken material | GBIF / ITIS | Animal-derived medicinal material; valid zoological source name and family added where identifiable. |
| Wuji Baifeng Pill | Cervi Cornus Colla | 鹿角胶 | Animal-derived | *Cervus nippon* Temminck, 1838 / *Cervus elaphus* Linnaeus, 1758 | Cervidae | Deer antler gelatin / Colla | GBIF / ITIS | Multi-source animal-derived medicinal material; recognized zoological source species and family information added where identifiable. |
| Wuji Baifeng Pill | Trionycis Carapax | 鳖甲 | Animal-derived | *Pelodiscus sinensis* (Wiegmann, 1835) | Trionychidae | Turtle shell / Carapax | GBIF / ITIS | Shell-derived medicinal material; recognized zoological source species and family information added where identifiable. |
| Wuji Baifeng Pill | Ostreae Concha | 牡蛎 | Shell-derived / animal-derived | *Magallana gigas* (Thunberg, 1793) / *Crassostrea talienwhanensis* (Crosse, 1862) / *Crassostrea rivularis* (Gould, 1861) | Ostreidae | Oyster shell / Concha | GBIF / WoRMS / ITIS | Multi-source shell-derived medicinal material; recognized zoological source species and family information added where identifiable. |
| Wuji Baifeng Pill | Mantidis Ootheca | 桑螵蛸 | Animal-derived | *Tenodera sinensis* Saussure, 1871 / *Statilia maculata* Thunberg, 1784 / *Hierodula patellifera* Serville, 1839 | Mantidae | Ootheca / egg case | GBIF / ITIS | Multi-source insect-derived medicinal material; recognized zoological source species and family information added where identifiable. |
| Wuji Baifeng Pill | Ginseng Radix et Rhizoma | 人参 | Botanical | *Panax* *ginseng* C.A.Mey. | Araliaceae | Root and rhizome / Radix et Rhizoma | POWO / MPNS | Plant-derived drug |
| Wuji Baifeng Pill | Astragali Radix | 黄芪 | Botanical | *Astragalus* *mongholicus* Bunge | Fabaceae | Root / Radix | POWO / MPNS | Multi-source medicinal material in pharmacopeial use; species entry standardized to one common accepted source |
| Wuji Baifeng Pill | Angelicae Sinensis Radix | 当归 | Botanical | *Angelica* *sinensis* (Oliv.) Diels | Apiaceae | Root / Radix | POWO / MPNS | Plant-derived drug |
| Wuji Baifeng Pill | Paeoniae Radix Alba | 白芍 | Botanical | *Paeonia* *lactiflora* Pall. | Paeoniaceae | Root / Radix | POWO / MPNS | Plant-derived drug |
| Wuji Baifeng Pill | Cyperi Rhizoma | 香附 | Botanical | *Cyperus* *rotundus* L. | Cyperaceae | Rhizome / Rhizoma | POWO / MPNS | Plant-derived drug |
| Wuji Baifeng Pill | Asparagi Radix | 天冬 | Botanical | *Asparagus* *cochinchinensis* (Lour.) Merr. | Asparagaceae | Tuberous root / Radix | POWO / MPNS | Plant-derived drug |
| Wuji Baifeng Pill | Glycyrrhizae Radix et Rhizoma | 甘草 | Botanical | *Glycyrrhiza* *uralensis* Fisch. ex DC. | Fabaceae | Root and rhizome / Radix et Rhizoma | POWO / MPNS | Multi-source medicinal material in pharmacopeial use; species entry standardized to one common accepted source |
| Wuji Baifeng Pill | Rehmanniae Radix | 地黄 | Botanical | *Rehmannia* *glutinosa* (Gaertn.) Libosch. ex DC. | Orobanchaceae | Root tuber / Radix | POWO / MPNS | Plant-derived drug |
| Wuji Baifeng Pill | Rehmanniae Radix Praeparata | 熟地黄 | Botanical | *Rehmannia* *glutinosa* (Gaertn.) Libosch. ex DC. | Orobanchaceae | Prepared root tuber / Radix Praeparata | POWO / MPNS | Plant-derived drug |
| Wuji Baifeng Pill | Chuanxiong Rhizoma | 川芎 | Botanical | *Conioselinum* *anthriscoides* (H.Boissieu) Pimenov & Kljuykov | Apiaceae | Rhizome / Rhizoma | POWO / MPNS | Plant-derived drug |
| Wuji Baifeng Pill | Stellariae Radix | 银柴胡 | Botanical | *Stellaria* *dichotoma* L. var. *lanceolata* Bunge | Caryophyllaceae | Root / Radix | POWO / MPNS | Plant-derived drug |
| Wuji Baifeng Pill | Salviae Miltiorrhizae Radix et Rhizoma | 丹参 | Botanical | *Salvia* *miltiorrhiza* Bunge | Lamiaceae | Root and rhizome / Radix et Rhizoma | POWO / MPNS | Plant-derived drug |
| Wuji Baifeng Pill | Dioscoreae Rhizoma | 山药 | Botanical | *Dioscorea* *polystachya* Turcz. | Dioscoreaceae | Rhizome / Rhizoma | POWO / MPNS | Plant-derived drug |
| Wuji Baifeng Pill | Euryales Semen | 芡实 | Botanical | *Euryale* *ferox* Salisb. | Nymphaeaceae | Seed / Semen | POWO / WFO / MPNS | Plant-derived drug |
| Wuji Baifeng Pill | Cervi Cornu Degelatinatum | 鹿角霜 | Animal-derived | *Cervus nippon* Temminck, 1838 / *Cervus elaphus* Linnaeus, 1758 | Cervidae | Degelatinized deer antler / Cornu Degelatinatum | GBIF / ITIS | Multi-source animal-derived medicinal material; recognized zoological source species and family information added where identifiable. |
| Guanhuangmu Granule | Rehmanniae Radix Praeparata | 熟地黄 | Botanical | *Rehmannia* *glutinosa* (Gaertn.) Libosch. ex DC. | Orobanchaceae | Prepared root tuber / Radix Praeparata | POWO / MPNS | Plant-derived drug |
| Guanhuangmu Granule | Testudinis Carapacis Colla | 龟甲胶 | Animal-derived | *Mauremys reevesii* (Gray, 1831) [syn. *Chinemys reevesii*] | Geoemydidae | Tortoise shell gelatin / Colla | GBIF / ITIS | Shell-derived animal medicinal material; valid zoological source name and family added where identifiable. |
| Guanhuangmu Granule | Phellodendri Chinensis Cortex | 关黄柏 | Botanical | *Phellodendron* *chinense* C.K.Schneid. | Rutaceae | Bark / Cortex | POWO / MPNS | Plant-derived drug |
| Guanhuangmu Granule | Anemarrhenae Rhizoma | 知母 | Botanical | *Anemarrhena* *asphodeloides* Bunge | Asparagaceae | Rhizome / Rhizoma | POWO / MPNS | Plant-derived drug |
| Guanhuangmu Granule | Paeoniae Radix Alba | 白芍 | Botanical | *Paeonia* *lactiflora* Pall. | Paeoniaceae | Root / Radix | POWO / MPNS | Plant-derived drug |
| Gengnianshu Tablet | Rehmanniae Radix Praeparata | 熟地黄 | Botanical | *Rehmannia* *glutinosa* (Gaertn.) Libosch. ex DC. | Orobanchaceae | Prepared root tuber / Radix Praeparata | POWO / MPNS | Plant-derived drug |
| Gengnianshu Tablet | Testudinis Carapax | 龟甲 | Animal-derived | *Mauremys reevesii* (Gray, 1831) [syn. *Chinemys reevesii*] | Geoemydidae | Tortoise shell / Carapax | GBIF / ITIS | Shell-derived animal medicinal material; valid zoological source name and family added where identifiable. |
| Gengnianshu Tablet | Cervi Cornu Degelatinatum | 鹿角霜 | Animal-derived | *Cervus nippon* Temminck, 1838 / *Cervus elaphus* Linnaeus, 1758 | Cervidae | Degelatinized deer antler / Cornu Degelatinatum | GBIF / ITIS | Multi-source animal-derived medicinal material; recognized zoological source species and family information added where identifiable. |
| Gengnianshu Tablet | Asini Corii Colla | 阿胶 | Animal-derived | *Equus asinus* Linnaeus, 1758 | Equidae | Donkey-hide gelatin / Colla | GBIF / ITIS | Animal-derived medicinal material; valid zoological source name and family added where identifiable. |
| Gengnianshu Tablet | Epimedii Folium | 淫羊藿 | Botanical | *Epimedium* *brevicornu* Maxim. | Berberidaceae | Leaf / Folium | POWO / MPNS | Multi-source medicinal material in pharmacopeial use; species entry standardized to one common accepted source |
| Gengnianshu Tablet | Schisandrae Chinensis Fructus | 五味子 | Botanical | *Schisandra* *chinensis* (Turcz.) Baill. | Schisandraceae | Fruit / Fructus | POWO / MPNS | Plant-derived drug |
| Gengnianshu Tablet | Angelicae Sinensis Radix | 当归 | Botanical | *Angelica* *sinensis* (Oliv.) Diels | Apiaceae | Root / Radix | POWO / MPNS | Plant-derived drug |
| Gengnianshu Tablet | Leonuri Herba | 益母草 | Botanical | *Leonurus* *japonicus* Houtt. | Lamiaceae | Aerial part / Herba | POWO / MPNS | Plant-derived drug |
| Gengnianshu Tablet | Moutan Cortex | 牡丹皮 | Botanical | *Paeonia* × *suffruticosa* Andrews | Paeoniaceae | Root bark / Cortex | POWO / MPNS | Plant-derived drug |
| Gengnianshu Tablet | Artemisiae Argyi Folium | 艾叶 | Botanical | *Artemisia* *argyi* H.Lév. & Vaniot | Asteraceae | Leaf / Folium | POWO / MPNS | Plant-derived drug |
| Gengnianshu Tablet | Poria | 茯苓 | Fungal-derived | *Wolfiporia* *cocos* (F.A.Wolf) Ryvarden & Gilb. | Polyporaceae | Sclerotium | Index Fungorum / MycoBank | Fungal-derived medicinal material; botanical validation not applicable |
| Gengnianshu Tablet | Alismatis Rhizoma | 泽泻 | Botanical | *Alisma* *plantago-aquatica* subsp. *orientale* (Sam.) Sam. | Alismataceae | Rhizome / Rhizoma | POWO / WFO / MPNS | Plant-derived drug |
| Gengnianshu Tablet | Dioscoreae Rhizoma | 山药 | Botanical | *Dioscorea* *polystachya* Turcz. | Dioscoreaceae | Rhizome / Rhizoma | POWO / MPNS | Plant-derived drug |
| Gengnianshu Tablet | Amomi Fructus | 砂仁 | Botanical | *Amomum* *villosum* Lour. | Zingiberaceae | Fruit / Fructus | POWO / MPNS | Plant-derived drug |
| Gengnianshu Tablet | Oryzanol | 谷维素 | Other non-botanical / chemical constituent | Not applicable | Not applicable | Chemical constituent / pharmaceutical ingredient | Not applicable | Non-botanical ingredient; botanical taxonomic validation not applicable |
| Gengnianshu Tablet | Vitamin B6 | 维生素 B6 | Other non-botanical / vitamin | Not applicable | Not applicable | Vitamin / pharmaceutical ingredient | Not applicable | Non-botanical ingredient; botanical taxonomic validation not applicable |
| Linglianhua Granule | Wuling Mycelia Powder | 乌灵菌粉 | Fungal-derived | *Xylaria* *nigripes* (Klotzsch) Cooke | Xylariaceae | Fungal mycelia powder | Index Fungorum / MycoBank | Fungal-derived medicinal material; botanical validation not applicable |
| Linglianhua Granule | Gardeniae Fructus | 栀子 | Botanical | *Gardenia* *jasminoides* J.Ellis | Rubiaceae | Fruit / Fructus | POWO / MPNS | Plant-derived drug |
| Linglianhua Granule | Ligustri Lucidi Fructus | 女贞子 | Botanical | *Ligustrum* *lucidum* W.T.Aiton | Oleaceae | Fruit / Fructus | POWO / MPNS | Plant-derived drug |
| Linglianhua Granule | Ecliptae Herba | 墨旱莲 | Botanical | *Eclipta* *prostrata* (L.) L. | Asteraceae | Aerial part / Herba | POWO / MPNS | Plant-derived drug |
| Linglianhua Granule | Lilii Bulbus | 百合 | Botanical | *Lilium* *brownii* F.E.Br. ex Miellez | Liliaceae | Bulb / Bulbus | POWO / MPNS | Multi-source medicinal material in pharmacopeial use; species entry standardized to one common accepted source |
| Linglianhua Granule | Rosae Rugosae Flos | 玫瑰花 | Botanical | *Rosa* *rugosa* Thunb. | Rosaceae | Flower / Flos | POWO / MPNS | Plant-derived drug |
| Linglianhua Granule | Leonuri Herba | 益母草 | Botanical | *Leonurus* *japonicus* Houtt. | Lamiaceae | Aerial part / Herba | POWO / MPNS | Plant-derived drug |
| Linglianhua Granule | Polygalae Radix | 远志 | Botanical | *Polygala* *tenuifolia* Willd. | Polygalaceae | Root / Radix | POWO / MPNS | Multi-source medicinal material in pharmacopeial use; species entry standardized to one common accepted source |
| Zhibai Dihuang Pill | Anemarrhenae Rhizoma | 知母 | Botanical | *Anemarrhena* *asphodeloides* Bunge | Asparagaceae | Rhizome / Rhizoma | POWO / MPNS | Plant-derived drug |
| Zhibai Dihuang Pill | Phellodendri Chinensis Cortex | 黄柏 | Botanical | *Phellodendron* *chinense* C.K.Schneid. | Rutaceae | Bark / Cortex | POWO / MPNS | Plant-derived drug |
| Zhibai Dihuang Pill | Rehmanniae Radix Praeparata | 熟地黄 | Botanical | *Rehmannia* *glutinosa* (Gaertn.) Libosch. ex DC. | Orobanchaceae | Prepared root tuber / Radix Praeparata | POWO / MPNS | Plant-derived drug |
| Zhibai Dihuang Pill | Corni Fructus | 山茱萸 | Botanical | *Cornus* *officinalis* Siebold & Zucc. | Cornaceae | Fruit / Fructus | POWO / MPNS | Plant-derived drug |
| Zhibai Dihuang Pill | Moutan Cortex | 牡丹皮 | Botanical | *Paeonia* × *suffruticosa* Andrews | Paeoniaceae | Root bark / Cortex | POWO / MPNS | Plant-derived drug |
| Zhibai Dihuang Pill | Dioscoreae Rhizoma | 山药 | Botanical | *Dioscorea* *polystachya* Turcz. | Dioscoreaceae | Rhizome / Rhizoma | POWO / MPNS | Plant-derived drug |
| Zhibai Dihuang Pill | Poria | 茯苓 | Fungal-derived | *Wolfiporia* *cocos* (F.A.Wolf) Ryvarden & Gilb. | Polyporaceae | Sclerotium | Index Fungorum / MycoBank | Fungal-derived medicinal material; botanical validation not applicable |
| Zhibai Dihuang Pill | Alismatis Rhizoma | 泽泻 | Botanical | *Alisma* *plantago-aquatica* subsp. *orientale* (Sam.) Sam. | Alismataceae | Rhizome / Rhizoma | POWO / WFO / MPNS | Plant-derived drug |

Abbreviations: CCPP, commercial Chinese polyherbal preparation; MPNS, Medicinal Plant Names Services; POWO, Plants of the World Online; WFO, World Flora Online.

Note: This table lists the botanical and other drugs included in each preparation. For plant-, fungal-, animal-, and shell-derived drugs or materials, valid scientific names, author citations where applicable, family information, and taxonomic validation sources were recorded where identifiable. Plant-derived drugs were checked using POWO, MPNS, and/or WFO where applicable. Fungal-derived medicinal materials were checked using Index Fungorum and/or MycoBank where applicable. Animal-derived and shell-derived materials were checked using recognized zoological or pharmacopeial source-name resources, including GBIF, ITIS, and/or WoRMS where applicable. Non-biological materials, including chemical constituents, vitamins, processed soot, compound processed materials, and excipients, were retained in the table but marked as not applicable for biological taxonomic validation. For complex preparations, the composition was summarized according to the most complete accessible product instruction, official drug information, pharmacopeial information, or publicly available regulatory information. When abbreviated ingredient descriptions using “等” (“and others”) were encountered, these were treated as incomplete descriptions rather than full formula lists.

## Table S3.3. Product-level reporting and composition-reporting status of CCPPs in the included RCTs

| **Study** | **CCPP** | **Full CCPP name** | **Composition reported in original RCT** | **How composition was reported in original RCT** | **Composition source used for composition summary** | **Manufacturer** | **Batch number** | **Production/expiry date** | **Regulatory approval** | **QC specification** | **Marker-metabolite/assay information** | **Product specification/dosage form/administration** | **Source location** | **Product-level reporting notes** | **Composition-reporting notes** |
| --- | --- | --- | --- | --- | --- | --- | --- | --- | --- | --- | --- | --- | --- | --- | --- |
| Chen JM 2023 | GHM | Guanhuangmu Granule (关黄母颗粒) | Yes | Product name + manufacturer + specification/dosage form/administration; full or near-complete formula-style composition reported | Product instruction / official drug information / pharmacopeial information / publicly available regulatory information | Tonghua Wantong Pharmaceutical Co., Ltd. (通化万通药业股份有限公司) | NR | NR | NR | NR | NR | Granule; one sachet per dose, three times daily | Section 1.4 Treatment methods | Reported: manufacturer. NR: batch number, production/expiry date, regulatory approval, QC specification, marker-metabolite/assay information. | Full or near-complete formula-style composition was reported. |
| Chen L 2013 | LWDH | Concentrated Liuwei Dihuang Pill (浓缩六味地黄丸) | Partial | Product name + manufacturer + batch number + approval number + specification/dosage form/administration; partial or representative composition reported | Product instruction / official drug information / pharmacopeial information / publicly available regulatory information | Jiuzhitang Co., Ltd. (九芝堂股份有限公司) | 20110611 | NR | NMPA approval No. Z43020145 | NR | NR | Pill; 12 g orally, once daily | Section 1.2 Methods | Reported: manufacturer, batch number, regulatory approval. NR: production/expiry date, QC specification, marker-metabolite/assay information. | Only partial composition was reported; selected drugs were mentioned. |
| Ding KQ 2018 | KT | Kuntai Capsule (坤泰胶囊) | Yes | Product name + manufacturer + specification/dosage form/administration; full or near-complete formula-style composition reported | Product instruction / official drug information / pharmacopeial information / publicly available regulatory information | Guiyang Xintian Pharmaceutical Co., Ltd. (贵阳新天药业有限责任公司) | NR | NR | NR | NR | NR | Capsule; four capsules per dose, three times daily | Section 1.2 Treatment methods | Reported: manufacturer. NR: batch number, production/expiry date, regulatory approval, QC specification, marker-metabolite/assay information. | Full or near-complete formula-style composition was reported. |
| Ding YL 2025 | KT | Kuntai Capsule (坤泰胶囊) | Yes | Product name + manufacturer + approval number + specification/dosage form/administration; full or near-complete formula-style composition reported | Product instruction / official drug information / pharmacopeial information / publicly available regulatory information | Guiyang Xintian Pharmaceutical Co., Ltd. (贵阳新天药业股份有限公司) | NR | NR | NMPA approval No. Z20000083 | NR | NR | Capsule; 2 g per dose, three times daily | Section 1.3 Methods | Reported: manufacturer, regulatory approval. NR: batch number, production/expiry date, QC specification, marker-metabolite/assay information. | Full or near-complete formula-style composition was reported. |
| Zhang Z 2019 | SG | Shugan Granule (舒肝颗粒) | Partial | Product name + manufacturer + approval number + specification/dosage form/administration; partial or representative composition reported | Product instruction / official drug information / pharmacopeial information / publicly available regulatory information | Kunming Traditional Chinese Medicine Factory Co., Ltd. (昆明中药厂有限公司) | NR | NR | NMPA approval No. Z53021150 | NR | NR | Granule; specification: 3 g × 12 sachets; 3 g per dose, three times daily | Section 1.2 Methods | Reported: manufacturer, regulatory approval. NR: batch number, production/expiry date, QC specification, marker-metabolite/assay information. | Partial composition was reported with “etc.”. |
| Liu Y 2020 | SG | Shugan Granule (舒肝颗粒) | Partial | Product name + manufacturer + approval number + specification/dosage form/administration; partial or representative composition reported | Product instruction / official drug information / pharmacopeial information / publicly available regulatory information | Kunming Traditional Chinese Medicine Factory Co., Ltd. (昆明中药厂有限公司) | NR | NR | NMPA approval No. z53021161 | NR | NR | Granule; 3 g × 16 sachets; one sachet per dose, twice daily | Section 1.4 Treatment methods | Reported: manufacturer, regulatory approval. NR: batch number, production/expiry date, QC specification, marker-metabolite/assay information. | Partial composition was reported with “etc.”. |
| Fan X 2018 | WJBF | Wuji Baifeng Pill (乌鸡白凤丸) | Yes | Product name + specification/dosage form/administration; full or near-complete formula-style composition reported | Product instruction / official drug information / pharmacopeial information / publicly available regulatory information | NR | NR | NR | NR | NR | NR | Pill; one pill per dose, twice or three times daily | Section 1.2 Treatment methods | Only product name and administration were reported; all other product-level information NR. | Formula-style composition was reported; minor differences may reflect product/manufacturer-version differences. |
| Ren W 2019 | LWDH | Liuwei Dihuang Pill (六味地黄丸) | Partial | Product name + manufacturer + batch number + specification/dosage form/administration; partial or representative composition reported | Product instruction / official drug information / pharmacopeial information / publicly available regulatory information | Beijing Tongrentang Technology Development Co., Ltd., Pharmaceutical Factory (北京同仁堂科技发展股份有限公司制药厂) | 14120576、17021435 | NR | NR | NR | NR | Pill; specification: 9 g per pill; one pill per dose, twice daily | Section 1.3 Medications | Reported: manufacturer, batch number. NR: production/expiry date, regulatory approval, QC specification, marker-metabolite/assay information. | Partial composition was reported with “etc.”. |
| Hou JX 2017 | KT | Kuntai Capsule (坤泰胶囊) | No | Product name + manufacturer + batch number + specification/dosage form/administration; full composition not reported | Product instruction / official drug information / pharmacopeial information / publicly available regulatory information | Guiyang Xintian Pharmaceutical Co., Ltd. (贵阳新天药业股份有限公司) | 130202 | NR | NR | NR | NR | Capsule; specification: 0.5 g per capsule; four capsules per dose, three times daily | Section 1.2 Medications | Reported: manufacturer, batch number. NR: production/expiry date, regulatory approval, QC specification, marker-metabolite/assay information. | Product traceability information was reported, but no drug composition was provided. |
| Xi YN 2021 | DK | Dingkun Pill (定坤丹) | Partial | Product name + manufacturer + approval number + specification/dosage form/administration; partial or representative composition reported | Product instruction / official drug information / pharmacopeial information / publicly available regulatory information | Inner Mongolia Jingxin Pharmaceutical Co., Ltd. (内蒙古京新药业有限公司) | NR | NR | NMPA approval No. Z15021765 | NR | NR | Pill; specification: 10.8 g × 1 pill per box; two pills daily | Section 1.2 Study methods | Reported: manufacturer, regulatory approval. NR: batch number, production/expiry date, QC specification, marker-metabolite/assay information. | Only representative drugs were mentioned for this complex preparation. |
| Fei Y 2024 | GHM | Guanhuangmu Granule (关黄母颗粒) | Yes | Product name + manufacturer + batch number + specification/dosage form/administration; full or near-complete formula-style composition reported | Product instruction / official drug information / pharmacopeial information / publicly available regulatory information | Tonghua Wantong Pharmaceutical Co., Ltd. (通化万通药业股份有限公司) | 20210207 | NR | NR | NR | NR | Granule; specification: 9 g × 10 sachets; one sachet per dose, three times daily | Section 1.2.1 Treatment methods | Reported: manufacturer, batch number. NR: production/expiry date, regulatory approval, QC specification, marker-metabolite/assay information. | Full or near-complete formula-style composition was reported. |
| Wang Y 2023 | GHM | Guanhuangmu Granule (关黄母颗粒) | Yes | Product name + manufacturer + specification/dosage form/administration; full or near-complete formula-style composition reported | Product instruction / official drug information / pharmacopeial information / publicly available regulatory information | Tonghua Wantong Pharmaceutical Co., Ltd. (通化万通药业股份有限公司) | NR | NR | NR | NR | NR | Granule; one sachet per dose, three times daily | Section 1.4 Treatment methods | Reported: manufacturer. NR: batch number, production/expiry date, regulatory approval, QC specification, marker-metabolite/assay information. | Full or near-complete formula-style composition was reported. |
| Liu WW 2025 | KT | Kuntai Capsule (坤泰胶囊) | Partial | Product name + manufacturer + approval number + specification/dosage form/administration; partial or representative composition reported | Product instruction / official drug information / pharmacopeial information / publicly available regulatory information | Guiyang Xintian Pharmaceutical Co., Ltd. (贵阳新天药业股份有限公司) | NR | NR | NMPA approval No. Z20000083 | NR | NR | Capsule; specification: 0.5 g per capsule; 0.5 mg per dose, once daily (as reported) | Section 1.3 Methods | Reported: manufacturer, regulatory approval. NR: batch number, production/expiry date, QC specification, marker-metabolite/assay information. | Only selected drugs were mentioned; the full formula was not reported. |
| Liu WY 2022 | LLH | Linglianhua Granule (灵莲花颗粒) | Partial | Product name + manufacturer + approval number + specification/dosage form/administration; partial or representative composition reported | Product instruction / official drug information / pharmacopeial information / publicly available regulatory information | Zhejiang ZuoLi Pharmaceutical Co., Ltd. (浙江佐力药业股份有限公司) | NR | NR | NMPA approval No. Z20090711 | NR | NR | Granule; one sachet per dose, twice daily | Section 1.3 Methods | Reported: manufacturer, regulatory approval. NR: batch number, production/expiry date, QC specification, marker-metabolite/assay information. | Partial composition was reported with “etc.”. |
| Liu CF 2020 | KT | Kuntai Capsule (坤泰胶囊) | Yes | Product name + manufacturer + approval number + specification/dosage form/administration; full or near-complete formula-style composition reported | Product instruction / official drug information / pharmacopeial information / publicly available regulatory information | Guiyang Xintian Pharmaceutical Co., Ltd. (贵阳新天药业股份有限公司) | NR | NR | NMPA approval No. Z20000083 | NR | NR | Capsule; four capsules, three times daily (tid) | Section 1.2 Treatment methods | Reported: manufacturer, regulatory approval. NR: batch number, production/expiry date, QC specification, marker-metabolite/assay information. | Full composition was inferable from the discussion; all six drugs were mentioned, although not listed as a formal composition statement in the intervention description. |
| Li SS 2023 | KT | Kuntai Capsule (坤泰胶囊) | Yes | Product name + manufacturer + approval number + specification/dosage form/administration; full or near-complete formula-style composition reported | Product instruction / official drug information / pharmacopeial information / publicly available regulatory information | Guiyang Xintian Pharmaceutical Co., Ltd. (贵阳新天药业股份有限公司) | NR | NR | NMPA approval No. Z20000083 | NR | NR | Capsule; specification: 0.5 g × 12 capsules × 2 blister plates; 2 g per dose, three times daily | Section 2.2 Study group | Reported: manufacturer, regulatory approval. NR: batch number, production/expiry date, QC specification, marker-metabolite/assay information. | Full or near-complete formula-style composition was reported in the discussion. |
| Xiang D 2025 | ZBDH | Zhibai Dihuang Pill (知柏地黄丸) | Yes | Product name + manufacturer + approval number + specification/dosage form/administration; full or near-complete formula-style composition reported | Product instruction / official drug information / pharmacopeial information / publicly available regulatory information | Henan Wanxi Pharmaceutical Co., Ltd. (河南省宛西制药股份有限公司) | NR | NR | NMPA approval No. Z41021904 | NR | NR | Concentrated pill; specification: 200 pills per box; every 8 pills equivalent to 3 g crude drug; four pills per dose, three times daily | Section 1.4 Methods | Reported: manufacturer, regulatory approval. NR: batch number, production/expiry date, QC specification, marker-metabolite/assay information. | Full or near-complete formula-style composition was reported. |
| Wu XJ 2022 | LWDH | Liuwei Dihuang Pill (六味地黄丸) | Partial | Product name + manufacturer + approval number + specification/dosage form/administration; partial or representative composition reported | Product instruction / official drug information / pharmacopeial information / publicly available regulatory information | Ningxia Qiyuan Chinese Medicine Co., Ltd. (宁夏启元国药有限公司) | NR | NR | NMPA approval No. Z20043842 | NR | NR | Pill; eight pills per dose, twice daily | Section 2 Treatment methods | Reported: manufacturer, regulatory approval. NR: batch number, production/expiry date, QC specification, marker-metabolite/assay information. | Partial composition was reported with “etc.”. |
| Wu YQ 2014 | XS | Xiangshao Granule (香芍颗粒) | Partial | Product name + manufacturer + batch number + specification/dosage form/administration; partial or representative composition reported | Product instruction / official drug information / pharmacopeial information / publicly available regulatory information | Yangtze River Pharmaceutical Group / Sichuan Hairong Pharmaceutical Co., Ltd. (扬子江药业集团/四川海蓉药业有限公司) | 13110101 | NR | NR | NR | NR | Granule; specification: 4 g per sachet; one sachet per dose, three times daily | Section 1.3 Methods | Reported: manufacturer, batch number. NR: production/expiry date, regulatory approval, QC specification, marker-metabolite/assay information. | Only representative drugs were mentioned in the discussion. |
| Zhou YY 2023 | KT | Kuntai Capsule (坤泰胶囊) | Partial | Product name + manufacturer + approval number + specification/dosage form/administration; partial or representative composition reported | Product instruction / official drug information / pharmacopeial information / publicly available regulatory information | Guiyang Xintian Pharmaceutical Co., Ltd. (贵阳新天药业股份有限公司) | NR | NR | NMPA approval No. Z20000083 | NR | NR | Capsule; 2.0 g per dose, three times daily | Section 1.2.2 Study group | Reported: manufacturer, regulatory approval. NR: batch number, production/expiry date, QC specification, marker-metabolite/assay information. | Partial composition was reported with “etc.”. |
| Tang JJ 2003 | ZBDH | Zhibai Dihuang Pill (知柏地黄丸) | No | Product name + specification/dosage form/administration; full composition not reported | Product instruction / official drug information / pharmacopeial information / publicly available regulatory information | NR | NR | NR | NR | NR | NR | Pill; 27 g daily | Section 1.2 Administration method | Only product name and administration were reported; all other product-level information NR. | Product name and administration were reported, but composition was not reported. |
| Wei CK 2020 | DZ | Dizhen Granule (地贞颗粒) | Partial | Product name + specification/dosage form/administration; partial or representative composition reported | Product instruction / official drug information / pharmacopeial information / publicly available regulatory information | NR | NR | NR | NR | NR | NR | Granule; one sachet per dose, three times daily | Section 1.2 Methods | Only product name and administration were reported; all other product-level information NR. | Only one representative drug was mentioned; the full formula was not reported. |
| Yuan Y 2015 | DZ | Dizhen Granule (地贞颗粒) | Yes | Product name + manufacturer + approval number + specification/dosage form/administration; full or near-complete formula-style composition reported | Product instruction / official drug information / pharmacopeial information / publicly available regulatory information | Tiansheng Pharmaceutical Group Co., Ltd. (天圣制药集团股份有限公司) | NR | NR | NMPA approval No. Z20103065 | NR | NR | Granule; specification: 5 g per sachet; one sachet per dose, three times daily | Section 1.2 Methods | Reported: manufacturer, regulatory approval. NR: batch number, production/expiry date, QC specification, marker-metabolite/assay information. | Full or near-complete formula-style composition was reported. |
| Wang Q 2020 | DZ | Dizhen Granule (地贞颗粒) | Yes | Product name + manufacturer + approval number + specification/dosage form/administration; full or near-complete formula-style composition reported | Product instruction / official drug information / pharmacopeial information / publicly available regulatory information | Tiansheng Pharmaceutical Group Co., Ltd. (天圣制药集团股份有限公司) | NR | NR | NMPA approval No. Z20103065 | NR | NR | Granule; specification: 5 g per sachet; one sachet per dose, three times daily | Section 1.2 Methods | Reported: manufacturer, regulatory approval. NR: batch number, production/expiry date, QC specification, marker-metabolite/assay information. | Full or near-complete formula-style composition was reported. |
| Shan D 2019 | KT | Kuntai Capsule (坤泰胶囊) | Yes | Product name + manufacturer + approval number + specification/dosage form/administration; full or near-complete formula-style composition reported | Product instruction / official drug information / pharmacopeial information / publicly available regulatory information | Guiyang Xintian Pharmaceutical Co., Ltd. (贵阳新天药业股份有限公司) | NR | NR | NMPA approval No. Z2000083 | NR | NR | Capsule; specification: 0.5 g × 36 capsules; 2 g per dose, three times daily | Section 1.2.2 Study group | Reported: manufacturer, regulatory approval. NR: batch number, production/expiry date, QC specification, marker-metabolite/assay information. | Formula-style composition was reported; the wording included “etc. six drugs,” but the six drugs were listed. |
| Kong XJ 2025 | XS | Xiangshao Granule (香芍颗粒) | Yes | Product name + manufacturer + approval number + specification/dosage form/administration; full or near-complete formula-style composition reported | Product instruction / official drug information / pharmacopeial information / publicly available regulatory information | Yangtze River Pharmaceutical Group Sichuan Hairong Pharmaceutical Co., Ltd. (扬子江药业集团四川海蓉药业有限公司) | NR | NR | NMPA approval No. Z20050425 | NR | NR | Granule; specification: 4 g; 4 g per dose, three times daily | Section 1.4 Treatment methods | Reported: manufacturer, regulatory approval. NR: batch number, production/expiry date, QC specification, marker-metabolite/assay information. | Full or near-complete formula-style composition was reported. |
| Ji Y 2016 | KT | Kuntai Capsule (坤泰胶囊) | Yes | Product name + manufacturer + approval number + specification/dosage form/administration; full or near-complete formula-style composition reported | Product instruction / official drug information / pharmacopeial information / publicly available regulatory information | Guiyang Xintian Pharmaceutical Co., Ltd. (贵阳新天药业股份有限公司) | NR | NR | NMPA approval No. Z20000083 | NR | NR | Capsule; specification: 0.5 g per capsule × 36 capsules per box; four capsules per dose, three times daily | Section 1.2.1 Medications and specifications | Reported: manufacturer, regulatory approval. NR: batch number, production/expiry date, QC specification, marker-metabolite/assay information. | Full or near-complete formula-style composition was reported. |
| An W 2024 | DK | Dingkun Pill (定坤丹) | Partial | Product name + manufacturer + approval number + specification/dosage form/administration; partial or representative composition reported | Product instruction / official drug information / pharmacopeial information / publicly available regulatory information | Shanxi Guangyuyuan Chinese Pharmaceutical Co., Ltd. (山西广誉远国药有限公司) | NR | NR | NMPA approval No. Z20059003 | NR | NR | 7 g per dose, twice daily | Materials and methods - Methods | Reported: manufacturer, regulatory approval. NR: batch number, production/expiry date, QC specification, marker-metabolite/assay information. | Only representative drugs were mentioned for this complex preparation. |
| Wang YH 2022 | DK | Dingkun Pill (定坤丹) | Partial | Product name + manufacturer + approval number + specification/dosage form/administration; partial or representative composition reported | Product instruction / official drug information / pharmacopeial information / publicly available regulatory information | Shanxi Guangyuyuan Chinese Pharmaceutical Co., Ltd. (山西广誉远国药有限公司) | NR | NR | NMPA approval No. Z20059003 | NR | NR | Specification: 7 g per bottle; 7 g orally per dose, twice daily | Section 1.4 Methods | Reported: manufacturer, regulatory approval. NR: batch number, production/expiry date, QC specification, marker-metabolite/assay information. | Only representative drugs were mentioned for this complex preparation. |
| Zeng F 2023 | DK | Dingkun Pill (定坤丹) | Partial | Product name + manufacturer + approval number + specification/dosage form/administration; partial or representative composition reported | Product instruction / official drug information / pharmacopeial information / publicly available regulatory information | Shanxi Guangyu Chinese Pharmaceutical Co., Ltd. (山西广誉国药有限公司) | NR | NR | NMPA approval No. Z20059003 | NR | NR | Specification: 7 g per bottle; 7 g per dose, twice daily | Section 1.2 Methods | Reported: manufacturer, regulatory approval. NR: batch number, production/expiry date, QC specification, marker-metabolite/assay information. | Partial composition was reported with “etc.” for this complex preparation. |
| Shi YR 2019 | DK | Dingkun Pill (定坤丹) | Partial | Product name + specification/dosage form/administration; partial or representative composition reported | Product instruction / official drug information / pharmacopeial information / publicly available regulatory information | NR | NR | NR | NR | NR | NR | Pill; one pill per dose, twice daily | Section 1.2 Methods | Only product name and administration were reported; all other product-level information NR. | Partial composition was reported with “etc.” for this complex preparation. |
| Wang M 2017 | DK | Dingkun Pill (定坤丹) | Partial | Product name + manufacturer + batch number + specification/dosage form/administration; partial or representative composition reported | Product instruction / official drug information / pharmacopeial information / publicly available regulatory information | Shanxi Guangyuyuan Chinese Pharmaceutical Co., Ltd. (山西广誉远国药有限公司) | 20130347、20150536 | NR | NR | NR | NR | Pill; specification: 10.8 g per pill; half a pill in the morning and evening daily | Section 1.4 Medications, 1.5 Grouping and treatment methods | Reported: manufacturer, batch number. NR: production/expiry date, regulatory approval, QC specification, marker-metabolite/assay information. | Only selected drugs were mentioned for this complex preparation. |
| Chen SM 2020 | DK | Dingkun Pill (定坤丹) | Partial | Product name + manufacturer + batch number + specification/dosage form/administration; partial or representative composition reported | Product instruction / official drug information / pharmacopeial information / publicly available regulatory information | Shanxi Guangyuyuan Chinese Pharmaceutical Co., Ltd. (山西广誉远国药有限公司) | 149170406 | NR | NR | NR | NR | Pill; specification: 6 g per pill; 6 g per dose, twice daily | Section 1.2 Medications, 1.3 Grouping and treatment methods | Reported: manufacturer, batch number. NR: production/expiry date, regulatory approval, QC specification, marker-metabolite/assay information. | Partial composition was reported with “etc.” for this complex preparation. |
| Zhao HX 2024 | DK | Dingkun Pill (定坤丹) | Partial | Product name + manufacturer + approval number + specification/dosage form/administration; partial or representative composition reported | Product instruction / official drug information / pharmacopeial information / publicly available regulatory information | Shanxi Guangyuyuan Chinese Pharmaceutical Co., Ltd. (山西广誉远国药有限公司) | NR | NR | NMPA approval No. Z14020656 | NR | NR | Pill; specification: 10.8 g per pill; one pill per dose, twice daily | Section 1.2 Methods | Reported: manufacturer, regulatory approval. NR: batch number, production/expiry date, QC specification, marker-metabolite/assay information. | Partial composition was reported with “etc.” for this complex preparation. |
| Ju LJ 2023 | DK | Dingkun Pill (定坤丹) | Partial | Product name + specification/dosage form/administration; partial or representative composition reported | Product instruction / official drug information / pharmacopeial information / publicly available regulatory information | NR | NR | NR | NR | NR | NR | Pill; half a pill per dose, twice daily | Section 1.3 Methods | Only product name and administration were reported; all other product-level information NR. | Partial composition was reported with “etc.” for this complex preparation. |
| Wang Y 2024 | DK | Dingkun Pill (定坤丹) | Partial | Product name + manufacturer + approval number + specification/dosage form/administration; partial or representative composition reported | Product instruction / official drug information / pharmacopeial information / publicly available regulatory information | Inner Mongolia Jingxin Pharmaceutical Co., Ltd. (内蒙古京新药业有限公司) | NR | NR | NMPA approval No. Z15021765 | NR | NR | Pill; specification: 10.8 g per pill; half a pill per dose, twice daily | Section 1.3 Methods | Reported: manufacturer, regulatory approval. NR: batch number, production/expiry date, QC specification, marker-metabolite/assay information. | Partial composition was reported with “etc.” for this complex preparation. |
| Wu J 2025 | DK | Dingkun Pill (定坤丹) | Partial | Product name + manufacturer + approval number + specification/dosage form/administration; partial or representative composition reported | Product instruction / official drug information / pharmacopeial information / publicly available regulatory information | Heilongjiang Shenge Pharmaceutical Co., Ltd. (黑龙江参鸽药业有限公司) | NR | NR | NMPA approval No. Z23021902 | NR | NR | 6 g per dose, twice daily | Section 1.3 Methods | Reported: manufacturer, regulatory approval. NR: batch number, production/expiry date, QC specification, marker-metabolite/assay information. | Partial composition was reported with “etc.” for this complex preparation. |
| Zhu XQ 2023 | DK | Dingkun Pill (定坤丹) | Partial | Product name + manufacturer + approval number + specification/dosage form/administration; partial or representative composition reported | Product instruction / official drug information / pharmacopeial information / publicly available regulatory information | Shanxi Guangyuyuan Chinese Pharmaceutical Co., Ltd. (山西广誉远国药有限公司) | NR | NR | NMPA approval No. Z14020656 | NR | NR | Pill; specification: 10.8 g per pill; 6 g per dose, twice daily | Section 1.3 Methods | Reported: manufacturer, regulatory approval. NR: batch number, production/expiry date, QC specification, marker-metabolite/assay information. | Only representative drugs were mentioned and the expression used “etc.”. |
| Gong CL 2016 | KT | Kuntai Capsule (坤泰胶囊) | Yes | Product name + manufacturer + batch number + approval number + specification/dosage form/administration; full or near-complete formula-style composition reported | Product instruction / official drug information / pharmacopeial information / publicly available regulatory information | Guiyang Xintian Pharmaceutical Co., Ltd. (贵阳新天药业股份有限公司) | 141009 | NR | NMPA approval No. Z20000083 | NR | NR | Capsule; specification: 0.5 g per capsule; four capsules per dose, three times daily | Section 1.2 Treatment methods | Reported: manufacturer, batch number, regulatory approval. NR: production/expiry date, QC specification, marker-metabolite/assay information. | Formula-style composition was reported; the wording included “etc. six drugs,” but the six drugs were listed. |
| Yin H 2025 | KT | Kuntai Capsule (坤泰胶囊) | Yes | Product name + manufacturer + approval number + specification/dosage form/administration; full or near-complete formula-style composition reported | Product instruction / official drug information / pharmacopeial information / publicly available regulatory information | Guiyang Xintian Pharmaceutical Co., Ltd. (贵阳新天药业股份有限公司) | NR | NR | NMPA approval No. Z20000083 | NR | NR | Capsule; specification: 0.5 g per capsule; 2 g per dose, three times daily (tid) | Section 1.2 Treatment methods | Reported: manufacturer, regulatory approval. NR: batch number, production/expiry date, QC specification, marker-metabolite/assay information. | Full or near-complete formula-style composition was reported. |
| Zhang Y 2025 | XS | Xiangshao Granule (香芍颗粒) | Partial | Product name + manufacturer + batch number + specification/dosage form/administration; partial or representative composition reported | Product instruction / official drug information / pharmacopeial information / publicly available regulatory information | Yangtze River Pharmaceutical Group Sichuan Hairong Pharmaceutical Co., Ltd. (扬子江药业集团四川海蓉药业有限公司) | 21080529 | NR | NR | NR | NR | Granule; one sachet per dose, three times daily | Section 1.2Methods | Reported: manufacturer, batch number. NR: production/expiry date, regulatory approval, QC specification, marker-metabolite/assay information. | Partial composition was reported with “etc.”. |
| Fang Q 2024 | XS | Xiangshao Granule (香芍颗粒) | Yes | Product name + manufacturer + approval number + specification/dosage form/administration; full or near-complete formula-style composition reported | Product instruction / official drug information / pharmacopeial information / publicly available regulatory information | Yangtze River Pharmaceutical Group Sichuan Hairong Pharmaceutical Co., Ltd. (扬子江药业集团四川海蓉药业有限公司) | NR | NR | NMPA approval No. Z20050425 | NR | NR | Granule; 4 g per dose, three times daily | Section 2 Treatment methods | Reported: manufacturer, regulatory approval. NR: batch number, production/expiry date, QC specification, marker-metabolite/assay information. | Full or near-complete formula-style composition was reported in the intervention description. |
| Zhou CQ 2015 | SG | Shugan Granule (舒肝颗粒) | Yes | Product name + approval number + specification/dosage form/administration; full or near-complete formula-style composition reported | Product instruction / official drug information / pharmacopeial information / publicly available regulatory information | NR | NR | NR | NMPA approval No. Z5321161 | NR | NR | Granule; 3 mg per dose, three times daily (as reported) | Section 1.2 Treatment methods | Reported: regulatory approval. NR: manufacturer, batch number, production/expiry date, QC specification, marker-metabolite/assay information. | Near-complete formula-style composition was reported; minor differences may reflect product/manufacturer-version differences. |
| Yang QX 2019 | GNS | Gengnianshu Tablet (更年舒片) | Partial | Product name + manufacturer + batch number + specification/dosage form/administration; partial or representative composition reported | Product instruction / official drug information / pharmacopeial information / publicly available regulatory information | Zhuzhou Qianjin Pharmaceutical Co., Ltd. (株洲千金药业股份有限公司) | 20120305、20130912、20150306、20160807、20170510 | NR | NR | NR | NR | Tablet; specification: 0.38 g per tablet; five tablets per dose, three times daily | Section 1.2 Medications, 1.3 Grouping and treatment methods | Reported: manufacturer, batch number. NR: production/expiry date, regulatory approval, QC specification, marker-metabolite/assay information. | Only representative drugs were mentioned with “etc.” for this complex preparation. |
| Liao Y 2021 | GNS | Gengnianshu Tablet (更年舒片) | Yes | Product name + manufacturer + approval number + specification/dosage form/administration; full or near-complete formula-style composition reported | Product instruction / official drug information / pharmacopeial information / publicly available regulatory information | Xi’an Qianhe Pharmaceutical Co., Ltd. (西安千禾药业股份有限公司) | NR | NR | NMPA approval No. Z20054491 | NR | NR | Tablet; specification: 0.48 g; five tablets per dose, three times daily | Section 1.2 Methods | Reported: manufacturer, regulatory approval. NR: batch number, production/expiry date, QC specification, marker-metabolite/assay information. | Full or near-complete formula-style composition was reported. |
| Chen MM 2020 | GNS | Gengnianshu Tablet (更年舒片) | No | Product name + specification/dosage form/administration; full composition not reported | Product instruction / official drug information / pharmacopeial information / publicly available regulatory information | NR | NR | NR | NR | NR | NR | Tablet; 2.4 mg per dose, three times daily (as reported) | Section 1.2 Methods | Only product name and administration were reported; all other product-level information NR. | Product name and administration were reported, but composition was not reported. |
| Liu QH 2016 | GNS | Gengnianshu Tablet (更年舒片) | Yes | Product name + manufacturer + approval number + specification/dosage form/administration; full or near-complete formula-style composition reported | Product instruction / official drug information / pharmacopeial information / publicly available regulatory information | Zhuzhou Qianjin Pharmaceutical Co., Ltd. (株洲千金药业股份有限公司) | NR | NR | NMPA approval No. Z20083325 | NR | NR | Tablet; five tablets per dose, three times daily | Section 1.5 Treatment methods | Reported: manufacturer, regulatory approval. NR: batch number, production/expiry date, QC specification, marker-metabolite/assay information. | Near-complete formula-style composition was reported; minor differences may reflect product/manufacturer-version differences. |
| Li WL 2022 | XS | Xiangshao Granule (香芍颗粒) | Partial | Product name + manufacturer + approval number + specification/dosage form/administration; partial or representative composition reported | Product instruction / official drug information / pharmacopeial information / publicly available regulatory information | Yangtze River Pharmaceutical Group Sichuan Hairong Pharmaceutical Co., Ltd. (扬子江药业集团四川海蓉药业有限公司) | NR | NR | NMPA approval No. Z20050425 | NR | NR | Granule; specification: 4 g × 9 sachets; one sachet per dose, three times daily | Section 1.2 Methods | Reported: manufacturer, regulatory approval. NR: batch number, production/expiry date, QC specification, marker-metabolite/assay information. | Only two representative drugs were mentioned. |
| Li WL 2022 | KT | Kuntai Capsule (坤泰胶囊) | No | Product name + manufacturer + approval number + specification/dosage form/administration; full composition not reported | Product instruction / official drug information / pharmacopeial information / publicly available regulatory information | Guiyang Xintian Pharmaceutical Co., Ltd. (贵阳新天药业股份有限公司) | NR | NR | NMPA approval No. Z20000083 | NR | NR | Capsule; specification: 0.5 g × 36 capsules; four capsules per dose, three times daily | Section 1.2 Methods | Reported: manufacturer, regulatory approval. NR: batch number, production/expiry date, QC specification, marker-metabolite/assay information. | Product traceability information was reported for the comparator, but no drug composition was provided. |
| Li XH 2021 | KT | Kuntai Capsule (坤泰胶囊) | Yes | Product name + manufacturer + approval number + specification/dosage form/administration; full or near-complete formula-style composition reported | Product instruction / official drug information / pharmacopeial information / publicly available regulatory information | Guiyang Xintian Pharmaceutical Co., Ltd. (贵阳新天药业股份有限公司) | NR | NR | NMPA approval No. Z20000083 | NR | NR | Capsule; 2.0 g per dose, three times daily | Section 1.2 Treatment methods | Reported: manufacturer, regulatory approval. NR: batch number, production/expiry date, QC specification, marker-metabolite/assay information. | Full formula-style composition was listed with “etc.” wording, but all six Kuntai drugs were mentioned. |
| Li SM 2019 | WJBF | Wuji Baifeng Pill (乌鸡白凤丸) | No | Product name + manufacturer + approval number + specification/dosage form/administration; full composition not reported | Product instruction / official drug information / pharmacopeial information / publicly available regulatory information | Beijing Tongrentang Co., Ltd., Tongrentang Pharmaceutical Factory (北京同仁堂股份有限公司同仁堂制药厂) | NR | NR | NMPA approval No. Z11020026 | NR | NR | Pill; 6 g per dose, twice daily | Section 1.3 Treatment methods | Reported: manufacturer, regulatory approval. NR: batch number, production/expiry date, QC specification, marker-metabolite/assay information. | Product traceability information was reported, but no drug composition was provided. |
| Yang B 2017 | KT | Kuntai Capsule (坤泰胶囊) | Yes | Product name + manufacturer + approval number + specification/dosage form/administration; full or near-complete formula-style composition reported | Product instruction / official drug information / pharmacopeial information / publicly available regulatory information | Guiyang Xintian Pharmaceutical Co., Ltd. (贵阳新天药业股份有限公司) | NR | NR | NMPA approval No. Z20000083 | NR | NR | Capsule; four capsules per dose, three times daily | Section 1.2 Treatment methods | Reported: manufacturer, regulatory approval. NR: batch number, production/expiry date, QC specification, marker-metabolite/assay information. | Full formula-style composition was listed with “etc.” wording, but all six Kuntai drugs were mentioned. |
| Ouyang H 2022 | KT | Kuntai Capsule (坤泰胶囊) | Yes | Product name + manufacturer + approval number + specification/dosage form/administration; full or near-complete formula-style composition reported | Product instruction / official drug information / pharmacopeial information / publicly available regulatory information | Guiyang Xintian Pharmaceutical Co., Ltd. (贵阳新天药业股份有限公司) | NR | NR | NMPA approval No. Z20000083 | NR | NR | Capsule; specification: 0.5 g per capsule; 2.0 g per dose, three times daily | Section 1.3 Treatment methods | Reported: manufacturer, regulatory approval. NR: batch number, production/expiry date, QC specification, marker-metabolite/assay information. | Full or near-complete formula-style composition was reported. |
| Cuan MR 2020 | KT | Kuntai Capsule (坤泰胶囊) | Partial | Product name + manufacturer + batch number + specification/dosage form/administration; partial or representative composition reported | Product instruction / official drug information / pharmacopeial information / publicly available regulatory information | Guiyang Xintian Pharmaceutical Co., Ltd. (贵阳新天药业股份有限公司) | 170716、180215 | NR | NR | NR | NR | Capsule; specification: 0.5 g per capsule; four capsules per dose, three times daily | Section 1.2 Medications, 1.3 Grouping and treatment methods | Reported: manufacturer, batch number. NR: production/expiry date, regulatory approval, QC specification, marker-metabolite/assay information. | Only four drugs were listed with “etc.”; full formula was not reported. |
| Wang HW 2025 | KT | Kuntai Capsule (坤泰胶囊) | Yes | Product name + specification/dosage form/administration; full or near-complete formula-style composition reported | Product instruction / official drug information / pharmacopeial information / publicly available regulatory information | NR | NR | NR | NR | NR | NR | Capsule; 2.0 g per dose, three times daily | Section 1.5 Treatment methods | Only product name and administration were reported; all other product-level information NR. | Full or near-complete formula-style composition was reported. |
| Wang XH 2023 | LWDH | Liuwei Dihuang Pill (六味地黄丸) | Yes | Product name + manufacturer + specification/dosage form/administration; full or near-complete formula-style composition reported | Product instruction / official drug information / pharmacopeial information / publicly available regulatory information | Henan Jishi Pharmaceutical Co., Ltd. (河南济世药业有限公司) | NR | NR | NR | NR | NR | Concentrated pill; eight pills orally per dose, twice daily | Section 1.3 Treatment methods | Reported: manufacturer. NR: batch number, production/expiry date, regulatory approval, QC specification, marker-metabolite/assay information. | Full or near-complete formula-style composition was reported. |
| Cai ZM 2016 | XS | Xiangshao Granule (香芍颗粒) | Yes | Product name + manufacturer + specification/dosage form/administration; full or near-complete formula-style composition reported | Product instruction / official drug information / pharmacopeial information / publicly available regulatory information | Yangtze River Pharmaceutical Group Sichuan Hairong Pharmaceutical Co., Ltd. (扬子江药业集团四川海蓉药业有限公司) | NR | NR | NR | NR | NR | Granule; one sachet per dose, three times daily | Section 1.2 Methods | Reported: manufacturer. NR: batch number, production/expiry date, regulatory approval, QC specification, marker-metabolite/assay information. | Near-complete formula-style composition was reported; minor differences may reflect product/manufacturer-version differences. |
| Shi LX 2022 | KT | Kuntai Capsule (坤泰胶囊) | Yes | Product name + manufacturer + approval number + specification/dosage form/administration; full or near-complete formula-style composition reported | Product instruction / official drug information / pharmacopeial information / publicly available regulatory information | Guiyang Xintian Pharmaceutical Co., Ltd. (贵阳新天药业股份有限公司) | NR | NR | Approval No. 20190705 (as reported) | NR | NR | Capsule; specification: 0.5 g per capsule; four capsules per dose, three times daily | Section 1.2.1 Treatment methods | Reported: manufacturer, regulatory approval. NR: batch number, production/expiry date, QC specification, marker-metabolite/assay information. | Full or near-complete formula-style composition was reported in the discussion. |
| Qi BB 2025 | KT | Kuntai Capsule (坤泰胶囊) | Yes | Product name + manufacturer + approval number + specification/dosage form/administration; full or near-complete formula-style composition reported | Product instruction / official drug information / pharmacopeial information / publicly available regulatory information | Guiyang Xintian Pharmaceutical Co., Ltd. (贵阳新天药业) | NR | NR | NMPA approval No. Z20000083 | NR | NR | Capsule; specification: 0.5 g per capsule; four capsules per dose, three times daily | Section 1.2 Treatment methods | Reported: manufacturer, regulatory approval. NR: batch number, production/expiry date, QC specification, marker-metabolite/assay information. | Full formula-style composition was reported in the discussion; one extracted drug name should be checked against the original PDF if needed. |
| Fu XN 2021 | LWDH | Liuwei Dihuang Pill (六味地黄丸) | Yes | Product name + manufacturer + approval number + specification/dosage form/administration; full or near-complete formula-style composition reported | Product instruction / official drug information / pharmacopeial information / publicly available regulatory information | Beijing Tongrentang Technology Development Co., Ltd., Pharmaceutical Factory (北京同仁堂科技发展股份有限公司制药厂) | NR | NR | NMPA approval No. Z11021283 | NR | NR | Pill; 6 g per dose, twice daily | Section 1.2 Methods | Reported: manufacturer, regulatory approval. NR: batch number, production/expiry date, QC specification, marker-metabolite/assay information. | Full or near-complete formula-style composition was reported in the discussion. |
| Li LJ 2022 | DK | Dingkun Pill (定坤丹) | Partial | Product name + manufacturer + batch number + approval number + specification/dosage form/administration; partial or representative composition reported | Product instruction / official drug information / pharmacopeial information / publicly available regulatory information | Shanxi Guangyuyuan Chinese Pharmaceutical Co., Ltd. (山西广誉远国药有限公司) | 3282008082 | NR | NMPA approval No. Z20059003 | NR | NR | Specification: 7 g × 6 bottles per box; 3.5 g per dose, twice daily | Section 1.2Methods | Reported: manufacturer, batch number, regulatory approval. NR: production/expiry date, QC specification, marker-metabolite/assay information. | Only selected drugs were mentioned for this complex preparation. |
| Geng YN 2021 | XS | Xiangshao Granule (香芍颗粒) | Yes | Product name + manufacturer + batch number + specification/dosage form/administration; full or near-complete formula-style composition reported | Product instruction / official drug information / pharmacopeial information / publicly available regulatory information | Yangtze River Pharmaceutical Group Sichuan Hairong Pharmaceutical Co., Ltd. (扬子江药业集团四川海蓉药业有限公司) | 170519、180416、190624 | NR | NR | NR | NR | Granule; specification: 4 g per sachet; one sachet per dose, three times daily | Section 1.2 Grouping and treatment methods | Reported: manufacturer, batch number. NR: production/expiry date, regulatory approval, QC specification, marker-metabolite/assay information. | Full or near-complete formula-style composition was reported. |
| Zhao AM 2013 | SG | Shugan Granule (舒肝颗粒) | Yes | Product name + manufacturer + specification/dosage form/administration; full or near-complete formula-style composition reported | Product instruction / official drug information / pharmacopeial information / publicly available regulatory information | Kunming Traditional Chinese Medicine Factory (昆明中药厂) | NR | NR | NR | NR | NR | Granule; specification: 3 g; 3 g orally per dose, three times daily | Section 1.2 Methods | Reported: manufacturer. NR: batch number, production/expiry date, regulatory approval, QC specification, marker-metabolite/assay information. | Full or near-complete formula-style composition was reported. |
| Zhao J 2026 | GHM | Guanhuangmu Granule (关黄母颗粒) | No | Product name + manufacturer + batch number + specification/dosage form/administration; full composition not reported | Product instruction / official drug information / pharmacopeial information / publicly available regulatory information | Tonghua Wantong Pharmaceutical Co., Ltd. (通化万通药业股份有限公司) | 231106、240217 | NR | NR | NR | NR | Granule; specification: 9 g per sachet; one sachet per dose, three times daily | Section 1.2 Medications, 1.3 Grouping and treatment methods | Reported: manufacturer, batch number. NR: production/expiry date, regulatory approval, QC specification, marker-metabolite/assay information. | Product traceability information was reported, but no drug composition was provided. |
| Lu Y 2018 | LLH | Linglianhua Granule (灵莲花颗粒) | Yes | Product name + manufacturer + batch number + specification/dosage form/administration; full or near-complete formula-style composition reported | Product instruction / official drug information / pharmacopeial information / publicly available regulatory information | Zhejiang ZuoLi Pharmaceutical Co., Ltd. (浙江佐力药业股份有限公司) | 140601 | NR | NR | NR | NR | Granule; 4 g per sachet, containing 13 g crude drug equivalent; one sachet per dose, twice daily | Section 5 Treatment methods | Reported: manufacturer, batch number. NR: production/expiry date, regulatory approval, QC specification, marker-metabolite/assay information. | Full formula-style composition was reported in the intervention description. |
| Lu Y 2018 | KT | Kuntai Capsule (坤泰胶囊) | Yes | Product name + manufacturer + batch number + specification/dosage form/administration; full or near-complete formula-style composition reported | Product instruction / official drug information / pharmacopeial information / publicly available regulatory information | Guiyang Xintian Pharmaceutical Co., Ltd. (贵阳新天药业股份有限公司) | 140504 | NR | NR | NR | NR | Capsule; 0.5 g per capsule; four capsules per dose, three times daily | Section 5 Treatment methods | Reported: manufacturer, batch number. NR: production/expiry date, regulatory approval, QC specification, marker-metabolite/assay information. | Full formula-style composition was reported in the intervention description for the comparator product. |
| Yu JX 2025 | KT | Kuntai Capsule (坤泰胶囊) | Yes | Product name + approval number + specification/dosage form/administration; full or near-complete formula-style composition reported | Product instruction / official drug information / pharmacopeial information / publicly available regulatory information | NR | NR | NR | NMPA approval No. Z20000083 | NR | NR | Capsule; 2 g per dose, three times daily | Section 1.3 Treatment methods | Reported: regulatory approval. NR: manufacturer, batch number, production/expiry date, QC specification, marker-metabolite/assay information. | Full formula-style composition was listed with “etc.” wording, but all six Kuntai drugs were mentioned. |
| Yan JH 2015 | ZBDH | Zhibai Dihuang Pill (知柏地黄丸) | No | Product name + manufacturer + approval number + specification/dosage form/administration; full composition not reported | Product instruction / official drug information / pharmacopeial information / publicly available regulatory information | Henan Wanxi Pharmaceutical Manufacturing Co., Ltd. (河南省宛西制药生产有限公司) | NR | NR | NMPA approval No. Z41021904 | NR | NR | Pill; specification: 200 pills per box; every 4 pills equivalent to 1.5 g crude drug; eight pills per dose, three times daily | Section 1.2 Methods | Reported: manufacturer, regulatory approval. NR: batch number, production/expiry date, QC specification, marker-metabolite/assay information. | Product traceability information was reported, but no drug composition was provided. |
| Yan XH 2019 | KT | Kuntai Capsule (坤泰胶囊) | No | Product name + manufacturer + approval number + specification/dosage form/administration; full composition not reported | Product instruction / official drug information / pharmacopeial information / publicly available regulatory information | Guiyang Xintian Pharmaceutical Co., Ltd. (贵阳新天药业) | NR | NR | NMPA approval No. Z20000083 | NR | NR | Capsule; four capsules per dose, three times daily | Section 1.3 Methods | Reported: manufacturer, regulatory approval. NR: batch number, production/expiry date, QC specification, marker-metabolite/assay information. | Product name, manufacturer, approval number, and administration were reported, but composition was not reported. |
| Chen YL 2019 | XS | Xiangshao Granule (香芍颗粒) | Partial | Product name + manufacturer + specification/dosage form/administration; partial or representative composition reported | Product instruction / official drug information / pharmacopeial information / publicly available regulatory information | Yangtze River Pharmaceutical Group Co., Ltd. (扬子江药业集团有限公司) | NR | NR | NR | NR | NR | Granule; one sachet per dose, three times daily | Section 1.2.2 Observation group | Reported: manufacturer. NR: batch number, production/expiry date, regulatory approval, QC specification, marker-metabolite/assay information. | Only two representative drugs were mentioned. |
| Chen LM 2024 | KT | Kuntai Capsule (坤泰胶囊) | Yes | Product name + manufacturer + approval number + specification/dosage form/administration; full or near-complete formula-style composition reported | Product instruction / official drug information / pharmacopeial information / publicly available regulatory information | Guiyang Xintian Pharmaceutical Co., Ltd. (贵阳新天药业股份有限公司) | NR | NR | NMPA approval No. Z20000083 | NR | NR | Capsule; four capsules per dose, three times daily | Section 1.3.2 Observation group | Reported: manufacturer, regulatory approval. NR: batch number, production/expiry date, QC specification, marker-metabolite/assay information. | Full formula-style composition was listed with “etc.” wording, but all six Kuntai drugs were mentioned. |
| Chen XF 2022 | KT | Kuntai Capsule (坤泰胶囊) | Yes | Product name + manufacturer + approval number + specification/dosage form/administration; full or near-complete formula-style composition reported | Product instruction / official drug information / pharmacopeial information / publicly available regulatory information | Guiyang Xintian Pharmaceutical Co., Ltd. (贵阳新天药业股份有限公司) | NR | NR | NMPA approval No. Z20000083 | NR | NR | Capsule; specification: 0.5 g per capsule; four capsules orally per dose, three times daily | Section 1.3 Treatment methods | Reported: manufacturer, regulatory approval. NR: batch number, production/expiry date, QC specification, marker-metabolite/assay information. | Full formula-style composition was listed with “etc.” wording, but all six Kuntai drugs were mentioned. |
| Chen Y 2024 | LWDH | Liuwei Dihuang Pill (六味地黄丸) | Yes | Product name + manufacturer + approval number + specification/dosage form/administration; full or near-complete formula-style composition reported | Product instruction / official drug information / pharmacopeial information / publicly available regulatory information | Shijiazhuang Wanhe Pharmaceutical Co., Ltd. (石家庄万和制药) | NR | NR | NMPA approval No. Z13020347 | NR | NR | Pill; specification: 9 g per pill; eight pills per dose, twice daily | Section 1.2 Study methods | Reported: manufacturer, regulatory approval. NR: batch number, production/expiry date, QC specification, marker-metabolite/assay information. | Full or near-complete formula-style composition was reported. |
| Chen Y 2022 | LWDH | Liuwei Dihuang Pill (六味地黄丸) | No | Product name + manufacturer + approval number + specification/dosage form/administration; full composition not reported | Product instruction / official drug information / pharmacopeial information / publicly available regulatory information | Jiangxi Huiren Pharmaceutical Co., Ltd. (江西汇仁药业有限公司) | NR | NR | NMPA approval No. Z20033154 | NR | NR | Pill; specification: 200 pills × 1 bottle; eight pills per dose, three times daily | Section 1.2 Methods | Reported: manufacturer, regulatory approval. NR: batch number, production/expiry date, QC specification, marker-metabolite/assay information. | Product traceability information was reported, but no drug composition was provided. |
| Wu YL 2023 | LWDH | Liuwei Dihuang Pill (六味地黄丸) | Yes | Product name + specification/dosage form/administration; full or near-complete formula-style composition reported | Product instruction / official drug information / pharmacopeial information / publicly available regulatory information | NR | NR | NR | NR | NR | NR | Pill; eight pills per dose, twice daily | Section 1.2 Study methods | Only product name and administration were reported; all other product-level information NR. | Full or near-complete formula-style composition was reported. |

Abbreviations: CCPP, commercial Chinese polyherbal preparation; NR, not reported; QC, quality control; RCT, randomized controlled trial.

Note: This table summarizes product-level reporting and composition-reporting status for the CCPPs used in the included RCTs. Complete product-level composition is provided in Table S3.2; the present table focuses on whether and how this composition information was reported in the original RCT publications. Because two RCTs reported two CCPP products within the same trial, this table includes 75 product-level records from 73 included RCTs. “Composition reported in original RCT” refers only to whether the original trial publication itself reported the preparation composition. “Yes” indicates that a full or near-complete formula-style composition list was reported. Minor differences in reported ingredients were not automatically treated as incomplete reporting because they may reflect product- or manufacturer-version differences. “Partial” indicates that only representative drugs, selected ingredients, or incomplete formula information was reported. “No” indicates that no drug-composition information was reported, even if product traceability information such as product name, manufacturer, batch number, approval number, specification, or administration was provided.

## Table S3.4. Shared botanical and other drugs across the included CCPPs

| **Shared drug/material** | **Origin category** | **CCPPs containing this drug/material** | **Notes** |
| --- | --- | --- | --- |
| Rehmannia-derived drugs, including Rehmanniae Radix and/or Rehmanniae Radix Praeparata | Botanical | Kuntai Capsule; Dingkun Pill; Liuwei Dihuang Pill; Wuji Baifeng Pill; Guanhuangmu Granule; Gengnianshu Tablet; Zhibai Dihuang Pill | Shared occurrence was summarized descriptively according to listed formula composition. Processed and unprocessed forms were grouped together where appropriate. |
| Paeoniae Radix Alba | Botanical | Kuntai Capsule; Dingkun Pill; Xiangshao Granule; Shugan Granule; Wuji Baifeng Pill; Guanhuangmu Granule | Shared botanical drug across multiple CCPPs; summarized descriptively according to listed formula composition. |
| Poria | Fungal-derived | Kuntai Capsule; Dingkun Pill; Liuwei Dihuang Pill; Shugan Granule; Gengnianshu Tablet; Zhibai Dihuang Pill | Fungal-derived medicinal material; shared occurrence was summarized descriptively and does not imply equivalent product quality or dose. |
| Glycyrrhizae Radix et Rhizoma | Botanical | Dizhen Granule; Dingkun Pill; Xiangshao Granule; Shugan Granule; Wuji Baifeng Pill | Shared botanical drug across several CCPPs; summarized descriptively according to listed formula composition. |
| Angelicae Sinensis Radix | Botanical | Dingkun Pill; Shugan Granule; Wuji Baifeng Pill; Gengnianshu Tablet | Shared botanical drug across several CCPPs; summarized descriptively according to listed formula composition. |
| Cyperi Rhizoma | Botanical | Dingkun Pill; Xiangshao Granule; Shugan Granule; Wuji Baifeng Pill | Shared botanical drug across several CCPPs; summarized descriptively according to listed formula composition. |
| Moutan Cortex | Botanical | Liuwei Dihuang Pill; Shugan Granule; Gengnianshu Tablet; Zhibai Dihuang Pill | Shared botanical drug across several CCPPs; summarized descriptively according to listed formula composition. |
| Dioscoreae Rhizoma | Botanical | Liuwei Dihuang Pill; Wuji Baifeng Pill; Gengnianshu Tablet; Zhibai Dihuang Pill | Shared botanical drug across several CCPPs; summarized descriptively according to listed formula composition. |
| Alismatis Rhizoma | Botanical | Liuwei Dihuang Pill; Gengnianshu Tablet; Zhibai Dihuang Pill | Shared botanical drug across several CCPPs; summarized descriptively according to listed formula composition. |
| Anemarrhenae Rhizoma | Botanical | Guanhuangmu Granule; Zhibai Dihuang Pill | Shared botanical drug across two CCPPs; summarized descriptively according to listed formula composition. |
| Phellodendri Chinensis Cortex | Botanical | Guanhuangmu Granule; Zhibai Dihuang Pill | Shared botanical drug across two CCPPs; summarized descriptively according to listed formula composition. |
| Ligustri Lucidi Fructus | Botanical | Dizhen Granule; Linglianhua Granule | Shared botanical drug in two granule preparations. |
| Ecliptae Herba | Botanical | Dizhen Granule; Linglianhua Granule | Shared botanical drug in two granule preparations. |
| Schisandrae Chinensis Fructus | Botanical | Dizhen Granule; Gengnianshu Tablet | Shared botanical drug in two CCPPs. |
| Gardeniae Fructus | Botanical | Shugan Granule; Linglianhua Granule | Shared botanical drug in two granule preparations. |
| Leonuri Herba / Leonurus-derived drugs | Botanical | Dingkun Pill; Wuji Baifeng Pill; Gengnianshu Tablet; Linglianhua Granule | Leonurus-derived drugs included Leonuri Herba and/or Leonuri Fructus and were grouped descriptively. |
| Chuanxiong Rhizoma | Botanical | Dingkun Pill; Xiangshao Granule; Wuji Baifeng Pill | Shared botanical drug across several CCPPs; summarized descriptively according to listed formula composition. |
| Ginseng Radix et Rhizoma / Ginseng Radix Rubra | Botanical | Dingkun Pill; Wuji Baifeng Pill | Ginseng-derived drugs were grouped descriptively; processed red ginseng was not treated as a separate botanical species. |
| Asini Corii Colla | Animal-derived | Kuntai Capsule; Dingkun Pill; Gengnianshu Tablet | Shared animal-derived material; botanical taxonomic validation is not applicable. |
| Cervi-derived materials, including Cervi Cornu Pantotrichum, Cervi Cornu Degelatinatum, and/or Cervi Cornus Colla | Animal-derived | Dingkun Pill; Wuji Baifeng Pill; Gengnianshu Tablet | Cervi-derived materials were grouped descriptively because different preparations used different processed forms. |
| Testudinis-derived materials, including Testudinis Carapacis Colla and/or Testudinis Carapax | Animal-derived | Guanhuangmu Granule; Gengnianshu Tablet | Shared animal-derived shell material or gelatin; botanical taxonomic validation is not applicable. |

Abbreviations: CCPP, commercial Chinese polyherbal preparation.

Note: This table summarizes recurring botanical and other drugs identified across the 11 CCPPs based on the composition information listed in Table S3.2. Processed and unprocessed forms of the same medicinal material were grouped descriptively where appropriate. Shared occurrence indicates overlap in listed formula composition only and does not imply equivalent dose, extraction process, quality-control specification, chemical profile, pharmacological activity, or clinical effect.

Appendix 4. Risk of bias assessment of RCTs

## Figure S4: Overall risk of bias presented as a percentage of each risk of bias item across all included studies. Green = Low risk, Red = High risk, Yellow = Some concerns.


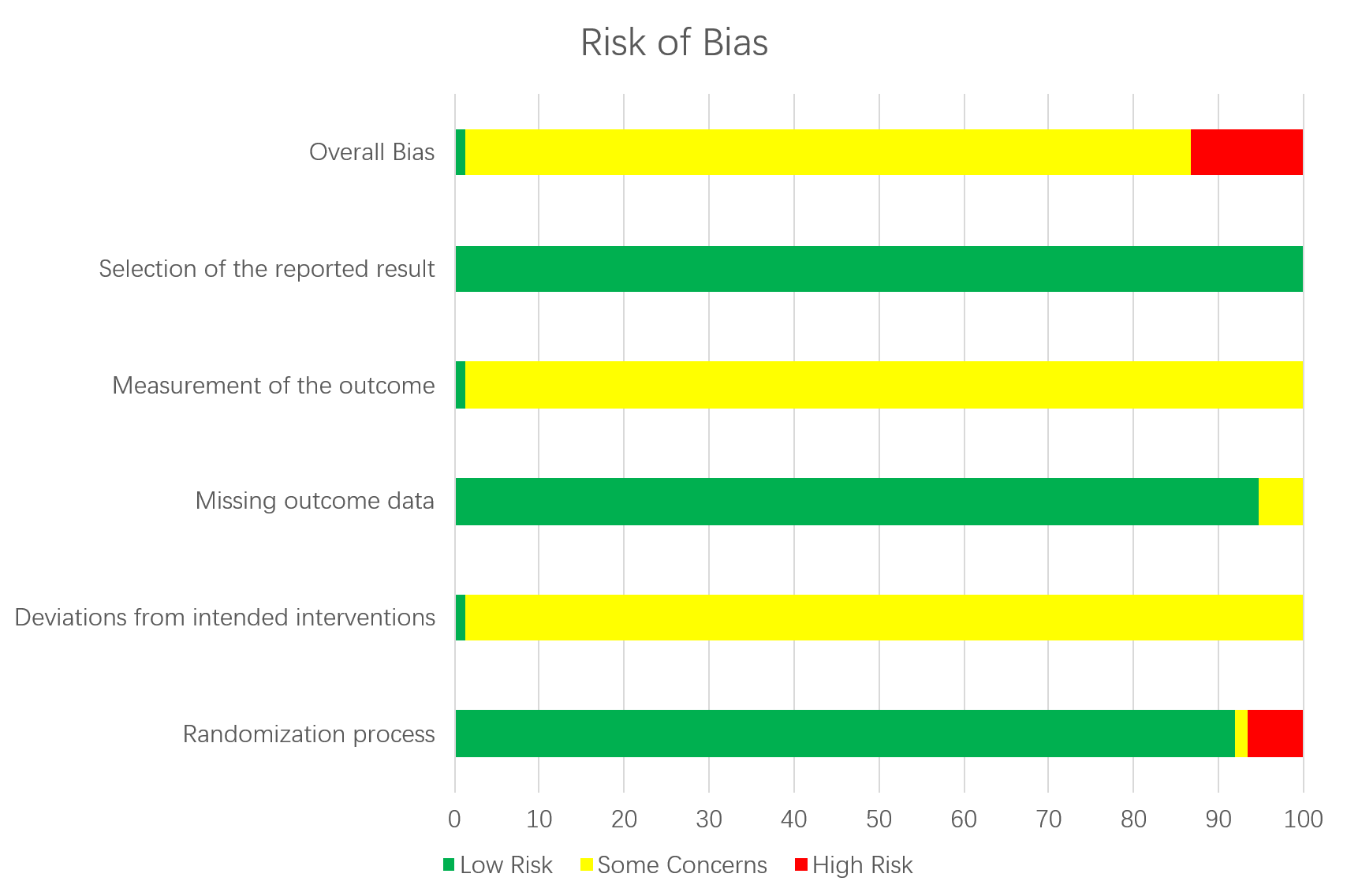


## Table S4: Risk of Bias Assessment of Included Studies (RoB 2.0)

| **Study** | **D1: Randomization process** | **D2: Deviations from intended interventions** | **D3: Missing outcome data** | **D4: Measurement of the outcome** | **D5: Selection of the reported result** | **Overall** |
| --- | --- | --- | --- | --- | --- | --- |
| Zhang Z 2019 | Low | Some concerns | Low | Some concerns | Low | Some concerns |
| Liu Y 2020 | Low | Some concerns | Low | Some concerns | Low | Some concerns |
| Zhou CQ 2015 | Low | Some concerns | Low | Some concerns | Low | Some concerns |
| An W 2024 | Low | Some concerns | Low | Some concerns | Low | Some concerns |
| Wang YH 2022 | Low | Some concerns | Low | Some concerns | Low | Some concerns |
| Xi YN 2021 | Low | Some concerns | Low | Some concerns | Low | Some concerns |
| Zeng F 2023 | Low | Some concerns | Low | Some concerns | Low | Some concerns |
| Wang M 2017 | Low | Some concerns | Low | Some concerns | Low | Some concerns |
| Chen SM 2020 | Low | Some concerns | Low | Some concerns | Low | Some concerns |
| Ju LJ 2023 | Low | Some concerns | Low | Some concerns | Low | Some concerns |
| Wang Y 2024 | Low | Some concerns | Low | Some concerns | Low | Some concerns |
| Li LJ 2022 | Low | Some concerns | Low | Some concerns | Low | Some concerns |
| Shi YR 2019 | Low | Some concerns | Low | Some concerns | Low | Some concerns |
| Zhao HX 2024 | Low | Some concerns | Low | Some concerns | Low | Some concerns |
| Wu J 2025 | Low | Some concerns | Low | Some concerns | Low | Some concerns |
| Zhu XQ 2023 | Low | Some concerns | Low | Some concerns | Low | Some concerns |
| Ren W 2019 | Low | Some concerns | Low | Some concerns | Low | Some concerns |
| Wu XJ 2022 | Low | Some concerns | Low | Some concerns | Low | Some concerns |
| Fu XN 2021 | Low | Some concerns | Low | Some concerns | Low | Some concerns |
| Chen L 2013 | Low | Some concerns | Low | Some concerns | Low | Some concerns |
| Wang XH 2023 | Low | Some concerns | Low | Some concerns | Low | Some concerns |
| Chen Y 2024 | Low | Some concerns | Low | Some concerns | Low | Some concerns |
| Chen Y 2022 | Low | Some concerns | Low | Some concerns | Low | Some concerns |
| Wu YL 2023 | Low | Some concerns | Low | Some concerns | Low | Some concerns |
| Yuan Y 2015 | Low | Some concerns | Low | Some concerns | Low | Some concerns |
| Wang Q 2020 | Low | Some concerns | Low | Some concerns | Low | Some concerns |
| Wei CK 2020 | Low | Some concerns | Low | Some concerns | Low | Some concerns |
| Kong XJ 2025 | Low | Some concerns | Low | Some concerns | Low | Some concerns |
| Fang Q 2024 | Low | Some concerns | Low | Some concerns | Low | Some concerns |
| Geng YN 2021 | High | Some concerns | Low | Some concerns | Low | High |
| Chen YL 2019 | High | Some concerns | Low | Some concerns | Low | High |
| Wu YQ 2014 | Low | Some concerns | Low | Some concerns | Low | Some concerns |
| Li WL 2022 | Low | Some concerns | Low | Some concerns | Low | Some concerns |
| Cai ZM 2016 | Low | Some concerns | Low | Some concerns | Low | Some concerns |
| Zhang Y 2025 | Low | Some concerns | Low | Some concerns | Low | Some concerns |
| Cuan MR 2020 | Low | Some concerns | Low | Some concerns | Low | Some concerns |
| Zhou YY 2023 | Low | Some concerns | Low | Some concerns | Low | Some concerns |
| Li XH 2021 | Low | Some concerns | Low | Some concerns | Low | Some concerns |
| Wang HW 2025 | Low | Some concerns | Low | Some concerns | Low | Some concerns |
| Ding KQ 2018 | Low | Some concerns | Low | Some concerns | Low | Some concerns |
| Ding YL 2025 | Low | Some concerns | Low | Some concerns | Low | Some concerns |
| Hou JX 2017 | Low | Some concerns | Low | Some concerns | Low | Some concerns |
| Liu CF 2020 | Low | Some concerns | Low | Some concerns | Low | Some concerns |
| Li SS 2023 | Low | Some concerns | Low | Some concerns | Low | Some concerns |
| Shan D 2019 | High | Some concerns | Low | Some concerns | Low | High |
| Ji Y 2016 | Low | Some concerns | Low | Some concerns | Low | Some concerns |
| Gong CL 2016 | Low | Some concerns | Low | Some concerns | Low | Some concerns |
| Yin H 2025 | Low | Some concerns | Low | Some concerns | Low | Some concerns |
| Liu WW 2025 | Low | Some concerns | Low | Some concerns | Low | Some concerns |
| Yang B 2017 | Low | Some concerns | Low | Some concerns | Low | Some concerns |
| OuYang H 2022 | Low | Some concerns | Low | Some concerns | Low | Some concerns |
| Shi LX 2022 | Low | Some concerns | Low | Some concerns | Low | Some concerns |
| Qi BB 2025 | Low | Some concerns | Low | Some concerns | Low | Some concerns |
| Yu JX 2025 | High | Some concerns | Low | Some concerns | Low | High |
| Yan XH 2019 | Low | Some concerns | Low | Some concerns | Low | Some concerns |
| Chen LM 2024 | Low | Some concerns | Low | Some concerns | Low | Some concerns |
| Chen XF 2022 | Low | Some concerns | Low | Some concerns | Low | Some concerns |
| Zhao AM 2013 | Low | Some concerns | Low | Some concerns | Low | Some concerns |
| Fan X 2018 | Low | Some concerns | Low | Some concerns | Low | Some concerns |
| Li SM 2019 | High | Some concerns | Low | Some concerns | Low | High |
| Wang Y 2023 | Low | Some concerns | Low | Some concerns | Low | Some concerns |
| Zhao J 2026 | Low | Some concerns | Low | Some concerns | Low | Some concerns |
| Fei Y 2024 | Low | Some concerns | Some concerns | Some concerns | Low | High |
| Chen JM 2023 | Low | Some concerns | Low | Some concerns | Low | Some concerns |
| Yang QX 2019 | Low | Some concerns | Low | Some concerns | Low | Some concerns |
| Liu QH 2016 | Low | Some concerns | Some concerns | Some concerns | Low | High |
| Liao Y 2021 | Low | Some concerns | Low | Some concerns | Low | Some concerns |
| Chen MM 2020 | Low | Some concerns | Low | Some concerns | Low | Some concerns |
| Liu WY 2022 | Low | Some concerns | Low | Some concerns | Low | Some concerns |
| Lu Y 2018 | Low | Low | Low | Low | Low | Low |
| Xiang D 2025 | Low | Some concerns | Low | Some concerns | Low | Some concerns |
| Yan JH 2015 | Some concerns | Some concerns | Low | Some concerns | Low | High |
| Tang JJ 2003 | Low | Some concerns | Low | Some concerns | Low | Some concerns |

Color coding: Green = Low risk; Yellow = Some concerns; Red = High risk. D1, randomization process; D2, deviations from intended interventions; D3, missing outcome data; D4, measurement of the outcome; D5, selection of the reported result.

Appendix 5. Evaluation of inconsistency, heterogeneity, and transitivity

## Table S5.1: Global consistency and Heterogeneity score

| **Clinical outcome** | **Chi^2^** | **P value** | **τ^2^** |
| --- | --- | --- | --- |
| KI | 2.57 | 0.1086 | 0.965019851 |
| MENQOL | 0.79 | 0.3745 | 0.378272887 |
| Overall Effective Rate | 23.78 | 0.06 | 0.002938019 |
| E2 | 1.16 | 0.2824 | 0.4523697229 |
| FSH | 4.64 | 0.0983 | 0.419377012 |
| LH | 0 | 0.9995 | 0.4142122071 |
| Endometrial Thickness | 0.06 | 0.8045 | 0.636401568 |
| Adverse Reactions | 0 | 0.9997 | 0.073370551 |

## Table S5.2. Node-splitting of KI. Inconsistency test between direct and indirect treatment comparisons in mixed treatment comparison.

| **Comparison** | **Direct** | | **Indirect** | | **Difference** | | | **τ^2^** |
| --- | --- | --- | --- | --- | --- | --- | --- | --- |
|  | Coef. | Std. Err. | Coef. | Std. Err. | Coef. | Std. Err. | P>\|z\| |  |
| SG +HT v.s. HT | 1.243431 | 1.226227 | 1.072326 | 4.886955 | 0.1711056 | 5.038448 | 0.973 | 0.9546825 |
| DK +HT v.s. HT | 3.02679 | 0.7296951 | 2.461854 | 25.95854 | 0.5649356 | 25.96882 | 0.983 | 0.9484187 |
| LWDH +HT v.s. HT | 1.837701 | 0.8661496 | 2.471536 | 31.74752 | -0.6338346 | 31.75934 | 0.984 | 0.9480171 |
| DZ +HT v.s. HT | 3.240055 | 1.234108 | 2.464446 | 44.83458 | 0.7756085 | 44.85156 | 0.986 | 0.9476917 |
| XS +HT v.s. HT | 0.8423368 | 0.7750162 | 2.474191 | 28.41614 | -1.631854 | 28.42671 | 0.954 | 0.9481817 |
| KT +HT v.s. LLH +HT | -0.3917679 | 1.666633 | -4.314781 | 1.783322 | 3.923013 | 2.440882 | 0.108 | 0.7602367 |
| KT +HT v.s. HT | 1.997267 | 0.4496869 | 5.837781 | 2.371664 | -3.840514 | 2.413957 | 0.112 | 0.7579544 |
| WJBF +HT v.s. HT | 1.30955 | 1.22398 | 2.457105 | 44.83418 | -1.147555 | 44.85089 | 0.98 | 0.9476882 |
| GHM +HT v.s. HT | 1.647908 | 1.00092 | 2.45477 | 36.63299 | -0.8068625 | 36.64667 | 0.982 | 0.9478523 |
| GNS +HT v.s. HT | 1.130135 | 1.224972 | 2.475406 | 44.83426 | -1.345272 | 44.85099 | 0.976 | 0.9476882 |
| LLH +HT v.s. HT | 6.319304 | 1.726875 | 2.387894 | 1.724869 | 3.931409 | 2.440753 | 0.107 | 0.7602167 |
| ZBDH +HT v.s. HT | 1.64369 | 1.226569 | 2.465724 | 44.83415 | -0.8220336 | 44.85093 | 0.985 | 0.9476882 |

Abbreviations: SG (Shugan Granule); LWDH (Liuwei Dihuang Pill); DZ (Dizhen Granule); XS (Xiangshao Granule); KT (Kuntai Capsule); DK (Dingkun Pill); WJBF (Wuji Baifeng Pill); GHM (Guanhuangmu Granule); GNS (Gengnianshu Tablet); LLH (Linglianhua Granule); ZBDH (Zhibai Dihuang Pill)

## Table S5.3. Node-splitting of E2. Inconsistency test between direct and indirect treatment comparisons in mixed treatment comparison.

| **Comparison** | **Direct** | | **Indirect** | | **Difference** | | | **τ^2^** |
| --- | --- | --- | --- | --- | --- | --- | --- | --- |
|  | Coef. | Std. Err. | Coef. | Std. Err. | Coef. | Std. Err. | P>\|z\| |  |
| SG +HT v.s. HT | -12.06 | 21.74382 | -18.26257 | 32.48507 | 6.202573 | 39.09057 | 0.874 | 0.720768694 |
| DK +HT v.s. HT | -44.47497 | 7.813079 | -27.35272 | 195.6348 | -17.12224 | 195.7906 | 0.93 | 0.690572497 |
| LWDH +HT v.s. HT | -18.61974 | 7.712362 | -28.24105 | 207.8332 | 9.621309 | 207.9763 | 0.963 | 0.468891152 |
| DZ +HT v.s. HT | -13.46 | 21.68092 | -28.11797 | 329.5572 | 14.65797 | 330.2698 | 0.965 | 0.4688303204 |
| XS +HT v.s. KT +HT | -10.78 | 21.6122 | 14.70186 | 10.16189 | -25.48187 | 23.88203 | 0.286 | 0.4670461266 |
| XS +HT v.s. HT | -19.69058 | 8.904175 | -43.6299 | 21.81145 | 23.93933 | 23.55941 | 0.31 | 0.46703143 |
| KT +HT v.s. HT | -34.33512 | 4.924534 | -9.422006 | 22.52402 | -24.91312 | 23.0559 | 0.28 | 0.4664187467 |
| GHM +HT v.s. HT | -12.56295 | 11.01349 | -27.95769 | 420.7411 | 15.39473 | 420.885 | 0.971 | 0.4686562534 |
| GNS +HT v.s. HT | -15.78848 | 11.05438 | -28.02051 | 673.5662 | 12.23203 | 673.6561 | 0.986 | 0.4685839502 |
| ZBDH +HT v.s. HT | -16.50999 | 22.0891 | -27.95297 | 1059.693 | 11.44298 | 1059.917 | 0.991 | 0.4685770212 |

Abbreviations: SG (Shugan Granule); LWDH (Liuwei Dihuang Pill); DZ (Dizhen Granule); XS (Xiangshao Granule); KT (Kuntai Capsule); DK (Dingkun Pill); WJBF (Wuji Baifeng Pill); GHM (Guanhuangmu Granule); GNS (Gengnianshu Tablet); LLH (Linglianhua Granule); ZBDH (Zhibai Dihuang Pill)

## Table S5.4. Node-splitting of FSH. Inconsistency test between direct and indirect treatment comparisons in mixed treatment comparison.

| **Comparison** | **Direct** | | **Indirect** | | **Difference** | | | **τ^2^** |
| --- | --- | --- | --- | --- | --- | --- | --- | --- |
|  | Coef. | Std. Err. | Coef. | Std. Err. | Coef. | Std. Err. | P>\|z\| |  |
| SG +HT v.s. HT | 1.359999 | 11.98182 | 5.752525 | 12.73957 | -4.392526 | 17.48888 | 0.802 | 0.435077015 |
| DK +HT v.s. HT | 7.617658 | 3.790251 | 6.731538 | 56.02146 | 0.8861201 | 56.14967 | 0.987 | 0.422726514 |
| LWDH +HT v.s. HT | 7.624111 | 4.242664 | 6.788756 | 85.92106 | 0.8353559 | 86.02583 | 0.992 | 0.420520725 |
| DZ +HT v.s. HT | 8.208847 | 8.511843 | 6.819426 | 355.9943 | 1.389421 | 356.0958 | 0.997 | 0.41918616 |
| XS +HT v.s. KT +HT | 9.32 | 11.59133 | -15.2276 | 5.129529 | 24.5476 | 12.67561 | 0.053 | 0.342868428 |
| XS +HT v.s. HT | 1.585332 | 4.411396 | 25.56867 | 11.73067 | -23.98333 | 12.53277 | 0.056 | 0.342847569 |
| KT +HT v.s. LLH +HT | -2.010004 | 12.89556 | 13.5609 | 12.25564 | -15.5709 | 17.79033 | 0.381 | 0.42803934 |
| KT +HT v.s. HT | 17.2612 | 2.648413 | -4.383277 | 9.671572 | 21.64448 | 10.02616 | 0.061 | 0.320911177 |
| WJBF +HT v.s. HT | 6.41 | 11.93389 | 6.775411 | 250.4143 | -0.3654107 | 250.6984 | 0.999 | 0.419443491 |
| GHM +HT v.s. HT | 4.640054 | 5.98299 | 6.832875 | 143.667 | -2.19282 | 143.7907 | 0.988 | 0.419603144 |
| GNS +HT v.s. HT | 10.85203 | 6.071223 | 6.843383 | 360.1896 | 4.008643 | 360.2415 | 0.991 | 0.419145656 |
| LLH +HT v.s. HT | 2.589962 | 11.97345 | 18.13836 | 13.15287 | -15.5484 | 17.78659 | 0.382 | 0.42806324 |
| ZBDH +HT v.s. HT | 11.52 | 12.40278 | 6.820131 | 845.0755 | 4.699869 | 845.1651 | 0.996 | 0.419100388 |

Abbreviations: SG (Shugan Granule); LWDH (Liuwei Dihuang Pill); DZ (Dizhen Granule); XS (Xiangshao Granule); KT (Kuntai Capsule); DK (Dingkun Pill); WJBF (Wuji Baifeng Pill); GHM (Guanhuangmu Granule); GNS (Gengnianshu Tablet); LLH (Linglianhua Granule); ZBDH (Zhibai Dihuang Pill)

## Table S5.5. Node-splitting of the auxiliary outcome: Overall Effective Rate. Inconsistency test between direct and indirect treatment comparisons in mixed treatment comparison.

| **Comparison** | **Direct** | | **Indirect** | | **Difference** | | | **τ^2^** |
| --- | --- | --- | --- | --- | --- | --- | --- | --- |
|  | Coef. | Std. Err. | Coef. | Std. Err. | Coef. | Std. Err. | P>\|z\| |  |
| SG +HT v.s. HT | -0.1744328 | 0.0597835 | -0.0938924 | 1.583535 | -0.0805404 | 1.584662 | 0.959 | 0.002938 |
| DK +HT v.s. HT | -0.1842682 | 0.0295258 | -0.3400031 | 7.105799 | 0.1557348 | 7.105918 | 0.983 | 0.002938 |
| LWDH +HT v.s. HT | -0.1866293 | 0.0335274 | -0.3558423 | 8.981589 | 0.169213 | 8.981703 | 0.985 | 0.002938 |
| DZ +HT v.s. HT | -0.2789958 | 0.0658571 | -0.322136 | 16.72507 | 0.0431401 | 16.72534 | 0.998 | 0.002938 |
| XS +HT v.s. KT +HT | -0.2116124 | 0.0470225 | 0.0707759 | 0.0355702 | -0.2823883 | 0.0589607 | 0 | 0.000788 |
| XS +HT v.s. HT | -0.1129839 | 0.0294884 | -0.3953725 | 0.0514262 | 0.2823886 | 0.0589598 | 0 | 0.000788 |
| KT +HT v.s. LLH +HT | 0.1019649 | 0.0873073 | -0.004448 | 0.0990652 | 0.1064129 | 0.1320474 | 0.42 | 0.00302 |
| KT +HT v.s. HT | -0.1885279 | 0.0213398 | 0.0651092 | 0.0511278 | -0.2536371 | 0.0546173 | 0 | 0.000898 |
| WJBF +HT v.s. HT | -0.1789063 | 0.1100969 | -0.3384469 | 26.37594 | 0.1595406 | 26.37637 | 0.995 | 0.002938 |
| GHM +HT v.s. HT | -0.1635232 | 0.045306 | -0.3427533 | 11.64833 | 0.1792301 | 11.64849 | 0.988 | 0.002938 |
| GNS +HT v.s. HT | -0.1802791 | 0.0465448 | -0.3413489 | 11.2438 | 0.1610698 | 11.24399 | 0.989 | 0.002938 |
| LLH +HT v.s. HT | -0.1670544 | 0.0962457 | -0.2734703 | 0.0904066 | 0.1064159 | 0.132048 | 0.42 | 0.00302 |
| ZBDH +HT v.s. HT | -0.1091993 | 0.0959966 | -0.3456661 | 19.06763 | 0.2364668 | 19.06808 | 0.99 | 0.002938 |

Abbreviations: SG (Shugan Granule); LWDH (Liuwei Dihuang Pill); DZ (Dizhen Granule); XS (Xiangshao Granule); KT (Kuntai Capsule); DK (Dingkun Pill); WJBF (Wuji Baifeng Pill); GHM (Guanhuangmu Granule); GNS (Gengnianshu Tablet); LLH (Linglianhua Granule); ZBDH (Zhibai Dihuang Pill)

Note: Statistically significant local inconsistency was identified for selected comparisons in the overall-effective-rate network, including XS+HT versus KT+HT and KT+HT versus HT. Because the overall effective rate was not uniformly defined across trials and often incorporated composite or subjective criteria, node-splitting results for this outcome indicate limited compatibility between direct and indirect evidence. Therefore, consistency-model estimates for this auxiliary outcome should be interpreted cautiously and used only as supplementary descriptive evidence.

## Table S5.6. Node-splitting of adverse reactions. Inconsistency test between direct and indirect treatment comparisons in mixed treatment comparison.

| **Comparison** | **Direct** | | **Indirect** | | **Difference** | | | **τ^2^** |
| --- | --- | --- | --- | --- | --- | --- | --- | --- |
|  | Coef. | Std. Err. | Coef. | Std. Err. | Coef. | Std. Err. | P>\|z\| |  |
| SG +HT v.s. HT | 1.829526 | 0.9740504 | 0.1288561 | 16.62157 | 1.700669 | 16.65012 | 0.919 | 0.0733 |
| DK +HT v.s. HT | 0.1510135 | 0.3241302 | 3.717981 | 71.05694 | -3.566967 | 71.05796 | 0.96 | 0.0733 |
| LWDH +HT v.s. HT | 0.0055728 | 0.4280615 | 3.618387 | 133.037 | -3.612814 | 133.0377 | 0.978 | 0.0734 |
| DZ +HT v.s. HT | -0.2819848 | 0.5976946 | 3.65983 | 155.4901 | -3.941815 | 155.4914 | 0.98 | 0.0734 |
| XS +HT v.s. HT | -0.1127216 | 0.4281742 | 3.611893 | 126.348 | -3.724615 | 126.3488 | 0.976 | 0.0733 |
| KT +HT v.s. LLH +HT | -0.0175443 | 0.8703801 | -2.945479 | 399.8325 | 2.927935 | 399.8334 | 0.994 | 0.0733 |
| KT +HT v.s. HT | 0.3509265 | 0.1931863 | 3.457983 | 46.24806 | -3.107056 | 46.24841 | 0.946 | 0.0734 |
| WJBF +HT v.s. HT | 0.4372138 | 0.9687349 | 3.689059 | 330.0533 | -3.251845 | 330.0543 | 0.992 | 0.0734 |
| GHM +HT v.s. HT | 0.6068315 | 0.5196906 | 3.512921 | 134.4473 | -2.90609 | 134.4478 | 0.983 | 0.0734 |
| GNS +HT v.s. HT | -0.5828085 | 0.6609882 | 3.721135 | 188.408 | -4.303944 | 188.4097 | 0.982 | 0.0734 |
| ZBDH +HT v.s. HT | -8.04e-11 | 2.034434 | 3.647485 | 501.9936 | -3.647485 | 501.9977 | 0.994 | 0.0733 |

Abbreviations: SG (Shugan Granule); LWDH (Liuwei Dihuang Pill); DZ (Dizhen Granule); XS (Xiangshao Granule); KT (Kuntai Capsule); DK (Dingkun Pill); WJBF (Wuji Baifeng Pill); GHM (Guanhuangmu Granule); GNS (Gengnianshu Tablet); LLH (Linglianhua Granule); ZBDH (Zhibai Dihuang Pill)

## Table S5.7. Assessment of transitivity assumption: distribution of key clinical characteristics across intervention nodes

| **Intervention** | **No. of trials** | **Sample size (T/C)** | **Age (years)** | **Course of disease (years)** | **Treatment Cycles (months)** |
| --- | --- | --- | --- | --- | --- |
| DK +HT | 13 | 562 / 562 | 50.87 ± 5.56 | 1.76 ± 0.89 | 3.12 ± 0.85 |
| DZ +HT | 3 | 153 / 153 | 48.96 ± 4.55 | 1.80 ± 0.53 | 3.00 ± 0.00 |
| GHM +HT | 4 | 235 / 233 | 49.46 ± 3.83 | 1.91 ± 0.56 | 2.14 ± 0.34 |
| GNS +HT | 4 | 190 / 191 | 51.18 ± 4.47 | 2.28 ± 1.37 | 3.00 ± 0.00 |
| KT +HT | 22 | 1107 / 1105 | 49.74 ± 4.38 | 1.39 ± 0.64 | 2.87 ± 0.44 |
| LLH +HT | 2 | 174 / 172 | 50.55 ± 2.95 | 4.41 ± 4.37 | 3.00 ± 0.00 |
| LWDH +HT | 8 | 711 / 714 | 49.21 ± 4.49 | 2.78 ± 2.44 | 3.70 ± 1.27 |
| SG +HT | 4 | 178 / 167 | 47.54 ± 3.57 | 1.91 ± 0.60 | 2.42 ± 0.50 |
| WJBF +HT | 2 | 107 / 105 | 47.80 ± 3.17 | 1.01 ± 0.61 | 3.00 ± 0.00 |
| XS +HT | 8 | 577 / 575 | 49.78 ± 3.08 | 1.63 ± 1.32 | 2.91 ± 0.99 |
| ZBDH +HT | 3 | 172 / 171 | 52.33 ± 2.97 | 1.91 ± 0.99 | 5.19 ± 1.62 |

Abbreviations: T, treatment group; C, control group; HT, hormone therapy (including estrogen, progestogen, combined estrogen-progestogen, or tibolone); SG (Shugan Granule); LWDH (Liuwei Dihuang Pill); DZ (Dizhen Granule); XS (Xiangshao Granule); KT (Kuntai Capsule); DK (Dingkun Pill); WJBF (Wuji Baifeng Pill); GHM (Guanhuangmu Granule); GNS (Gengnianshu Tablet); LLH (Linglianhua Granule); ZBDH (Zhibai Dihuang Pill). Age, course of disease, and treatment duration are reported as the weighted mean ± pooled standard deviation of the treatment group values across contributing trials. For consistency, treatment duration was standardized to months (assuming 1 cycle = 4 weeks = 1 month). The transitivity assumption requires that the distribution of potential effect modifiers (age, course of disease, and treatment duration) is broadly similar across intervention nodes, such that indirect comparisons are clinically meaningful.

Appendix 6. Network meta-analysis of various CCPPs on secondary endpoints of perimenopausal symptoms

## Figure S6.1 Network map of the KI, and forest plot of network effect sizes compared with Hormone Therapy(HT).


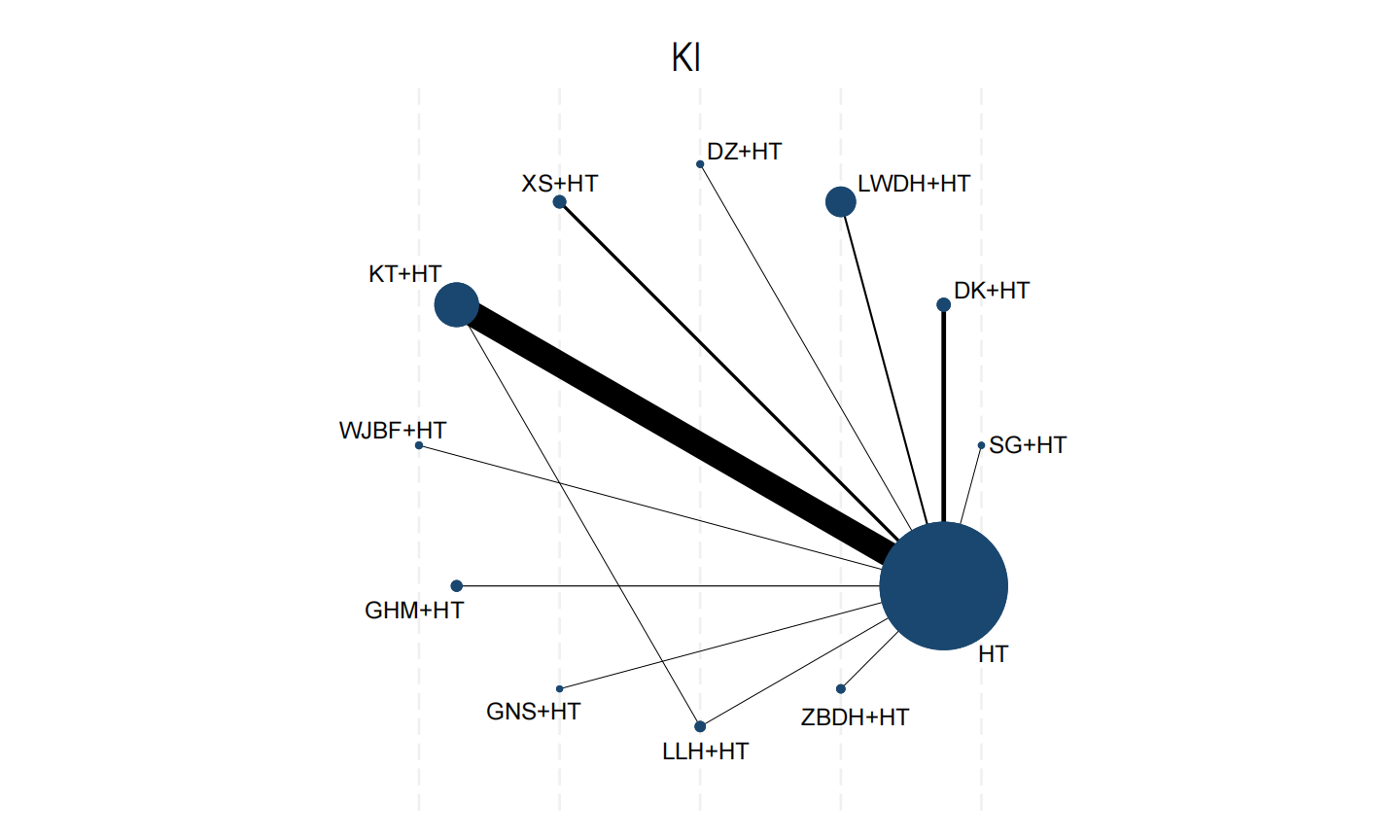


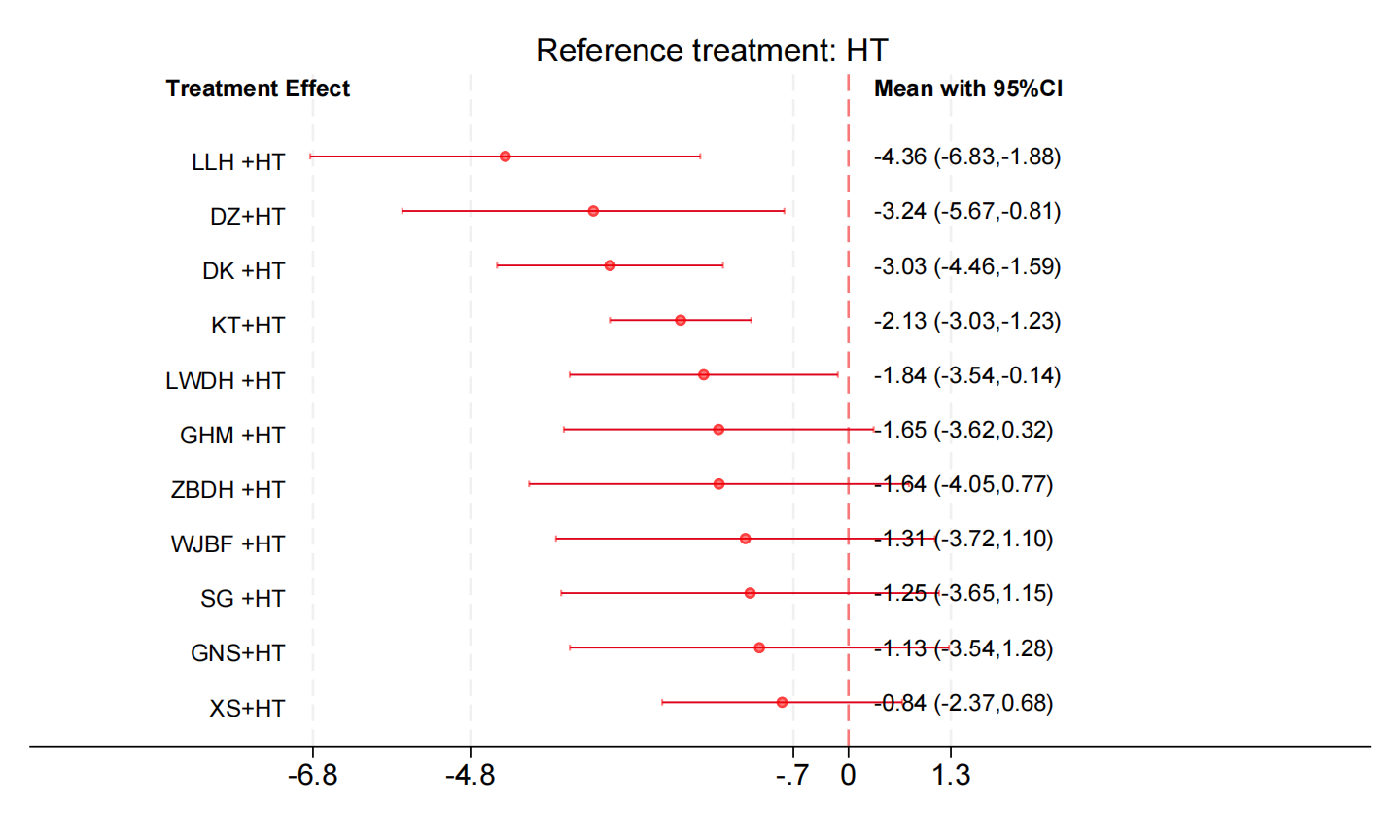


## Figure S6.2 Network map of the MENQOL, and forest plot of network effect sizes compared with HT.
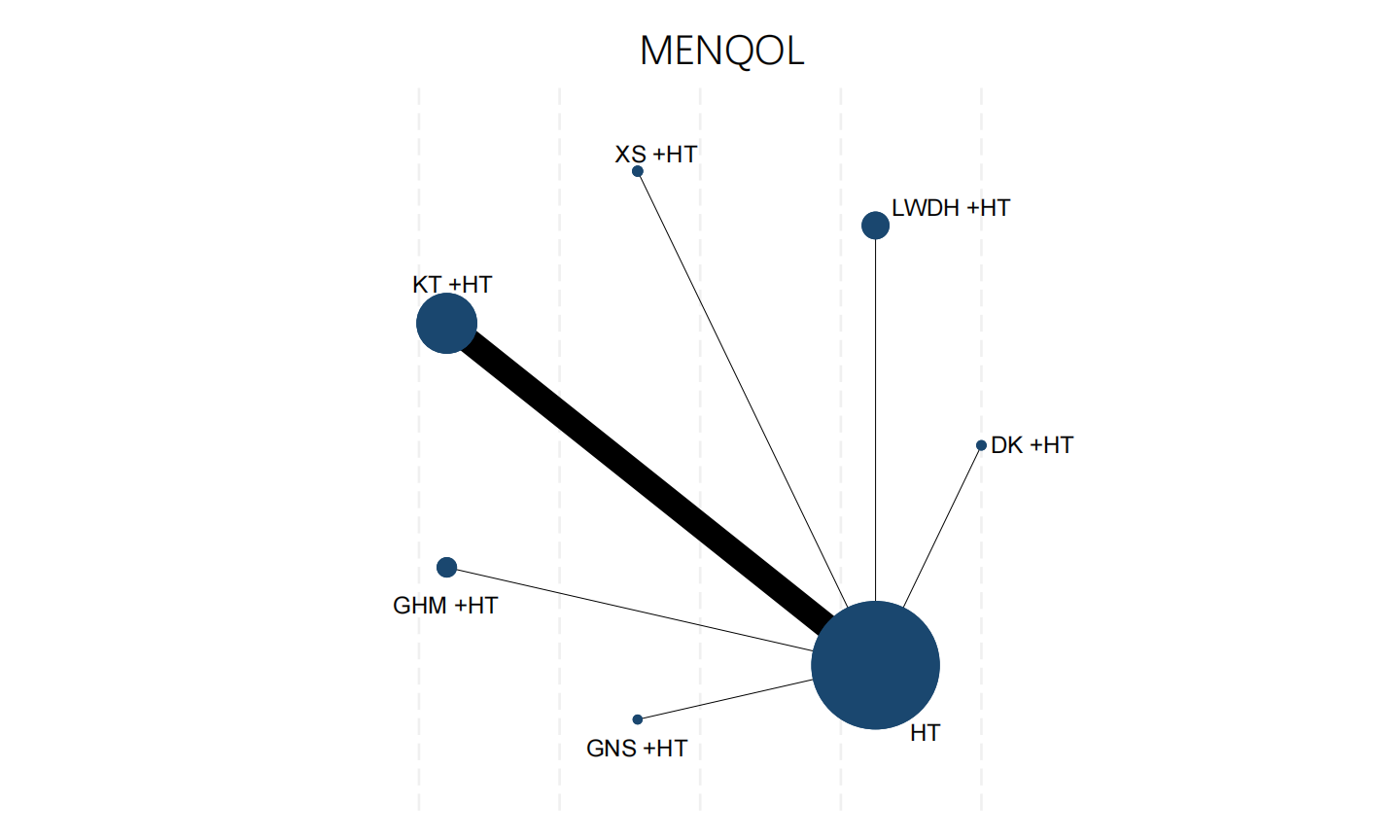


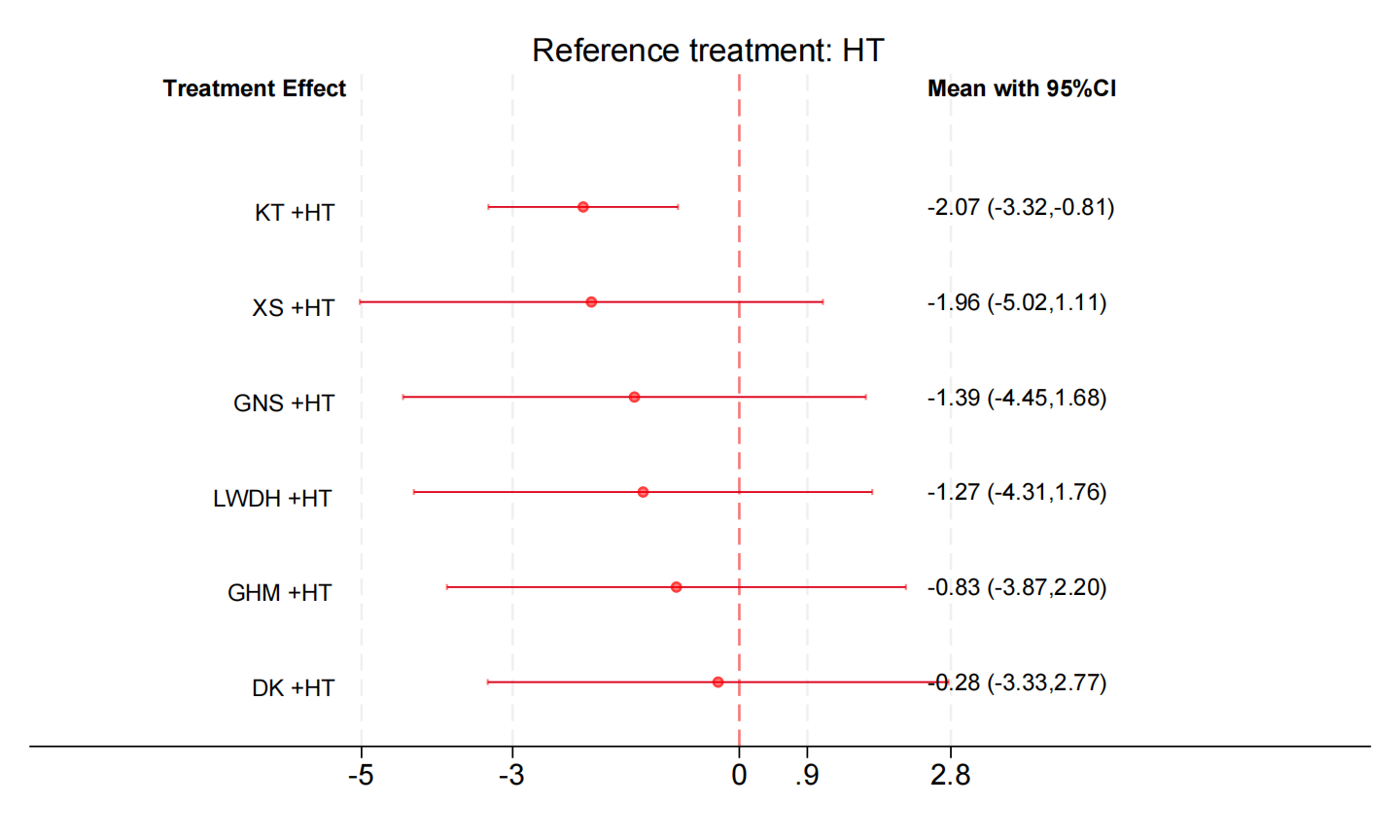


## Figure S6.3 Network map of the E2, and forest plot of network effect sizes compared with HT.
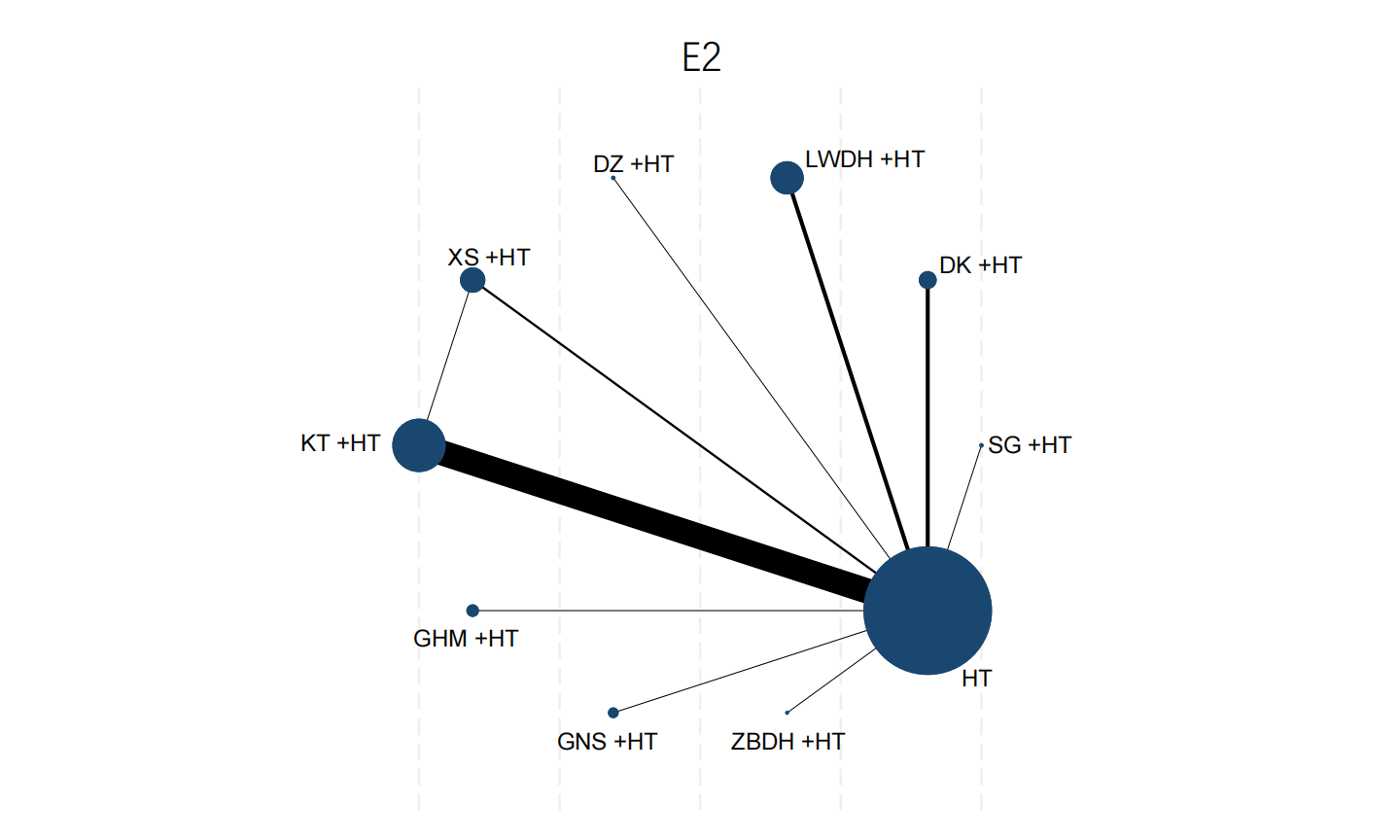


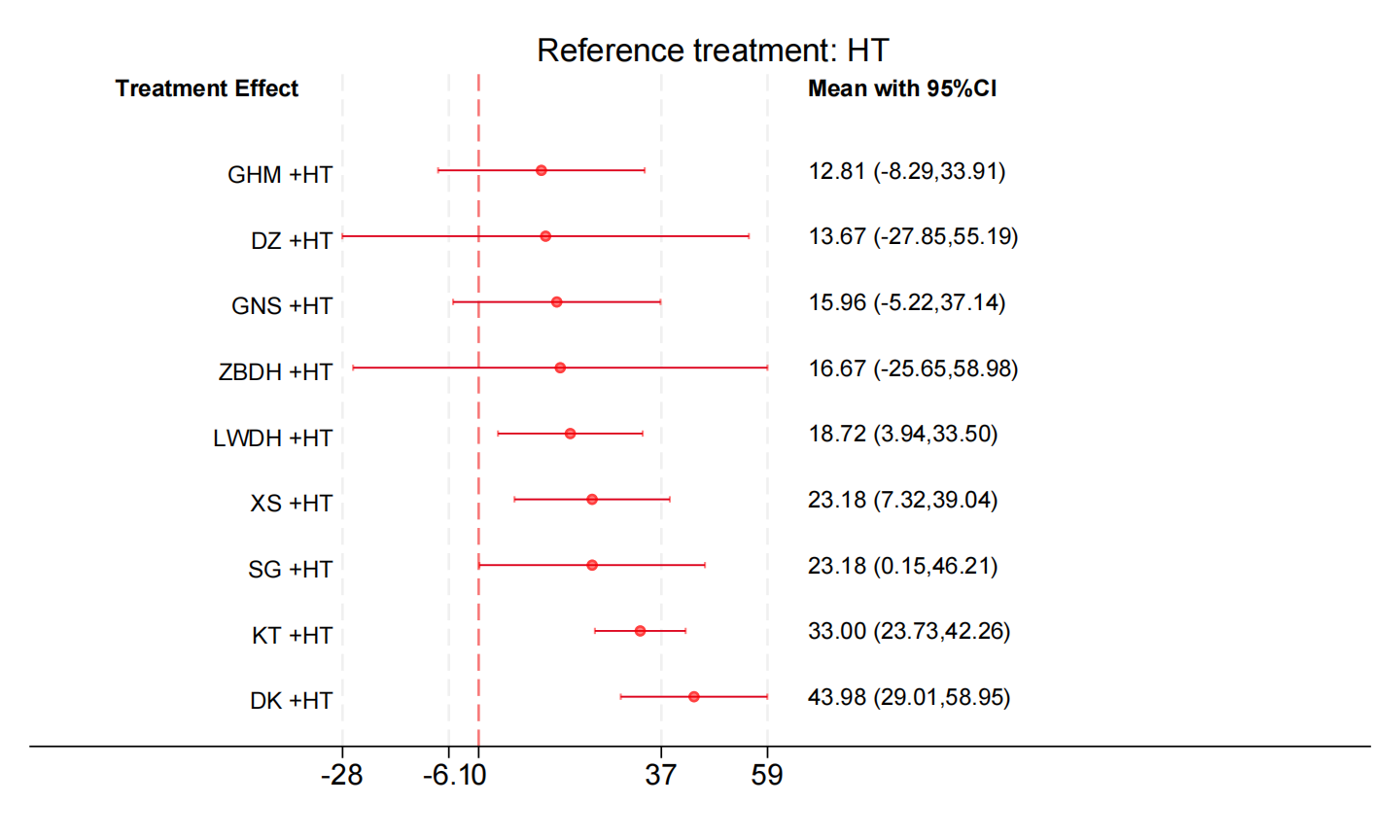


## Figure S6.4 Network map of the FSH, and forest plot of network effect sizes compared with HT.


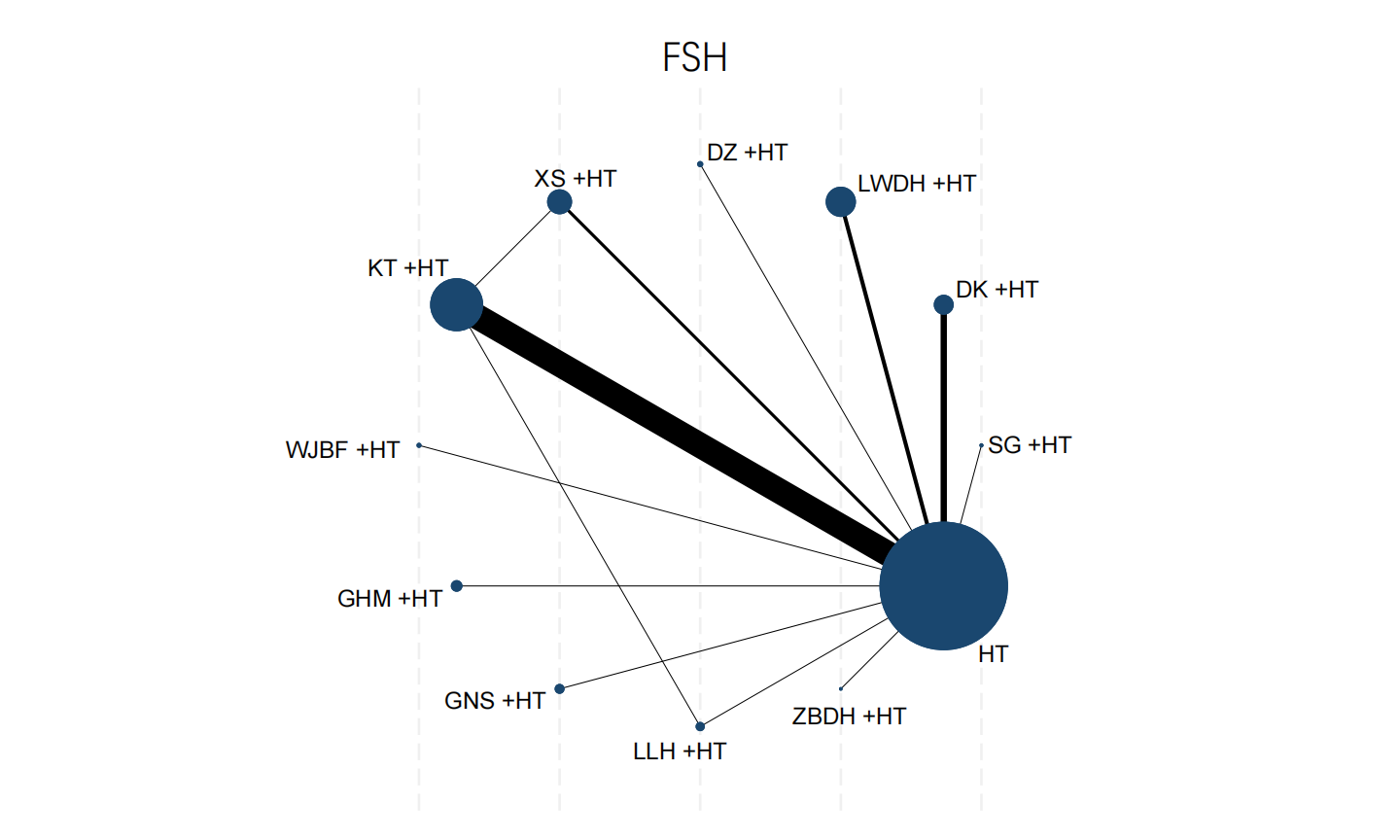


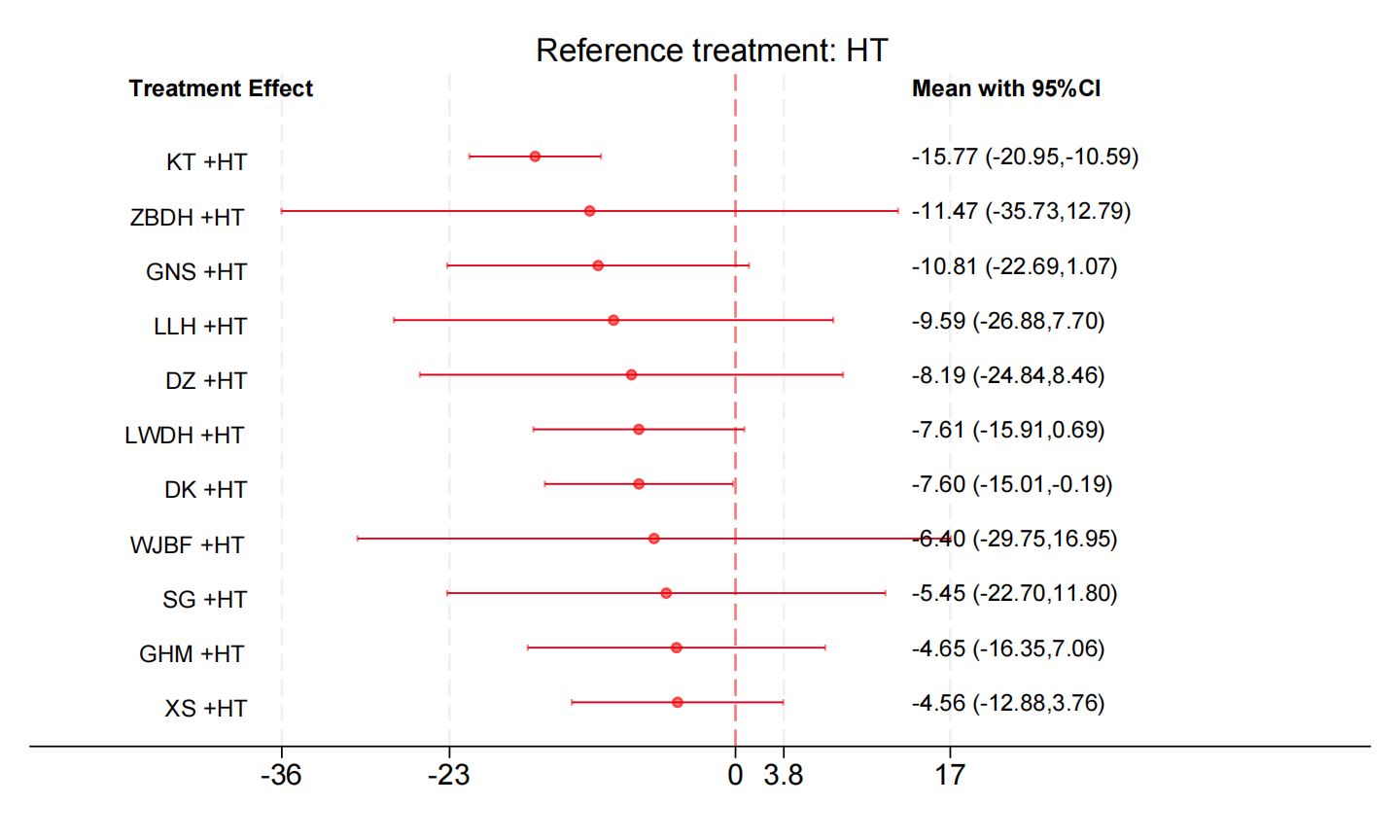


## Figure S6.5 Network map of the LH, and forest plot of network effect sizes compared with HT.


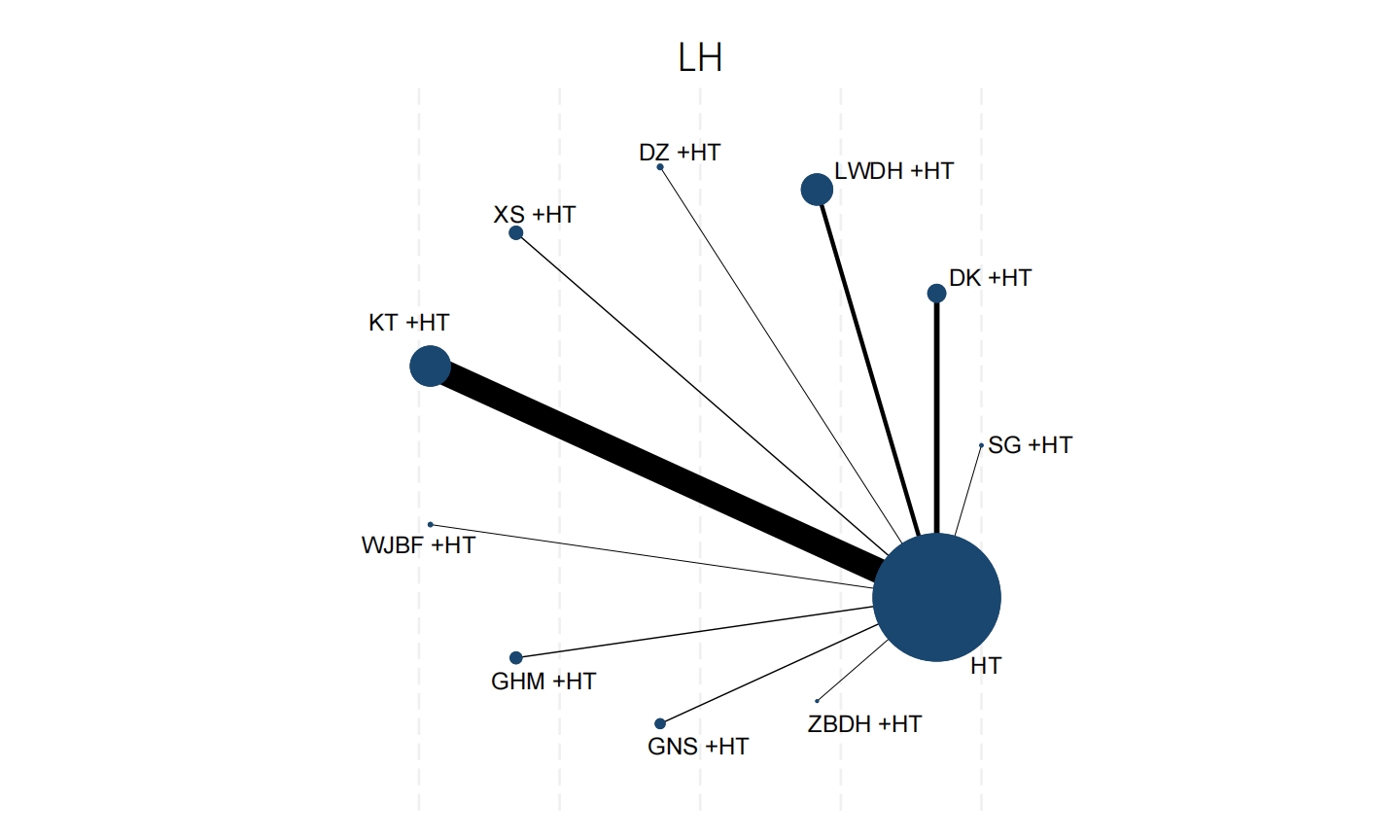

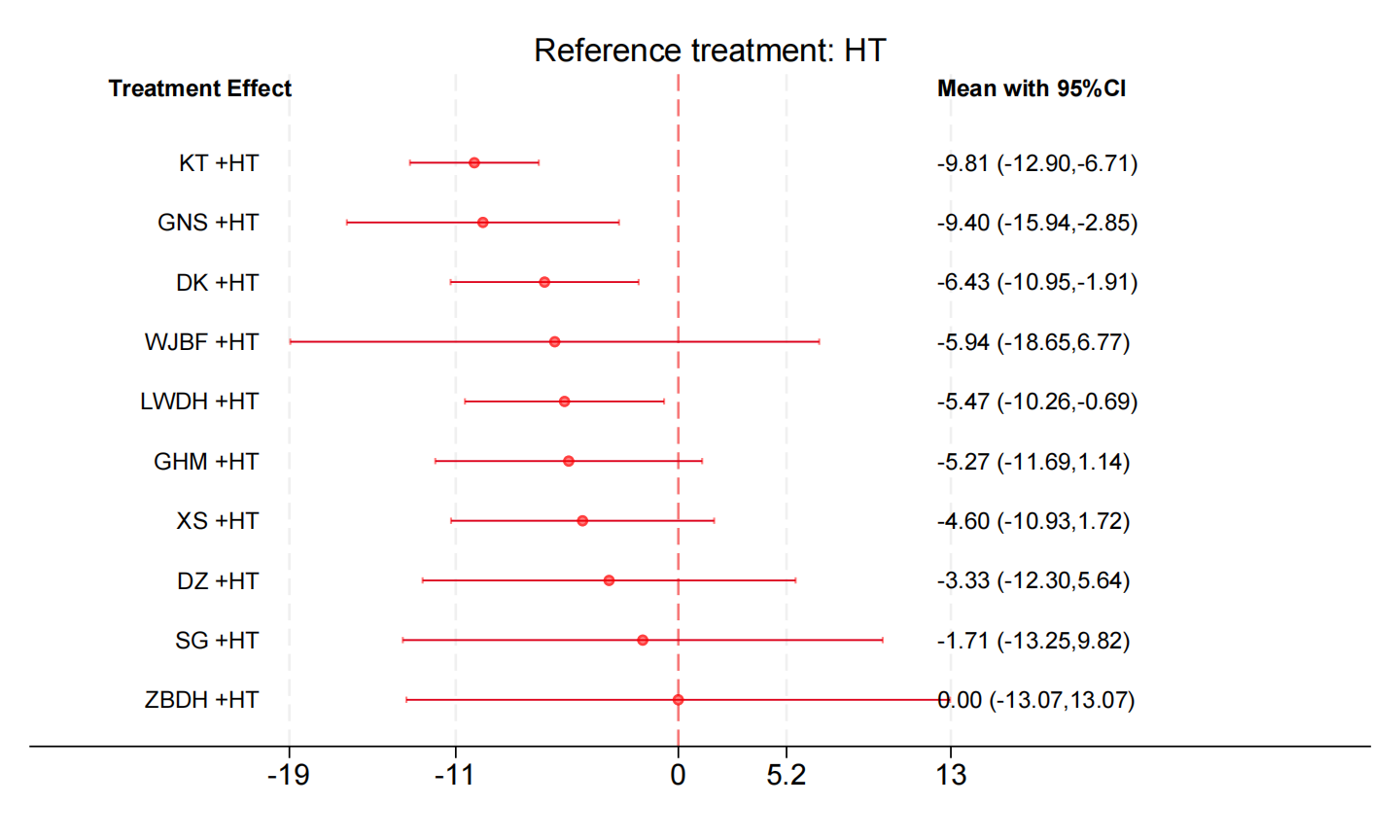


## Figure S6.6 Network map of the Overall effective rate, and forest plot of network effect sizes compared with HT.


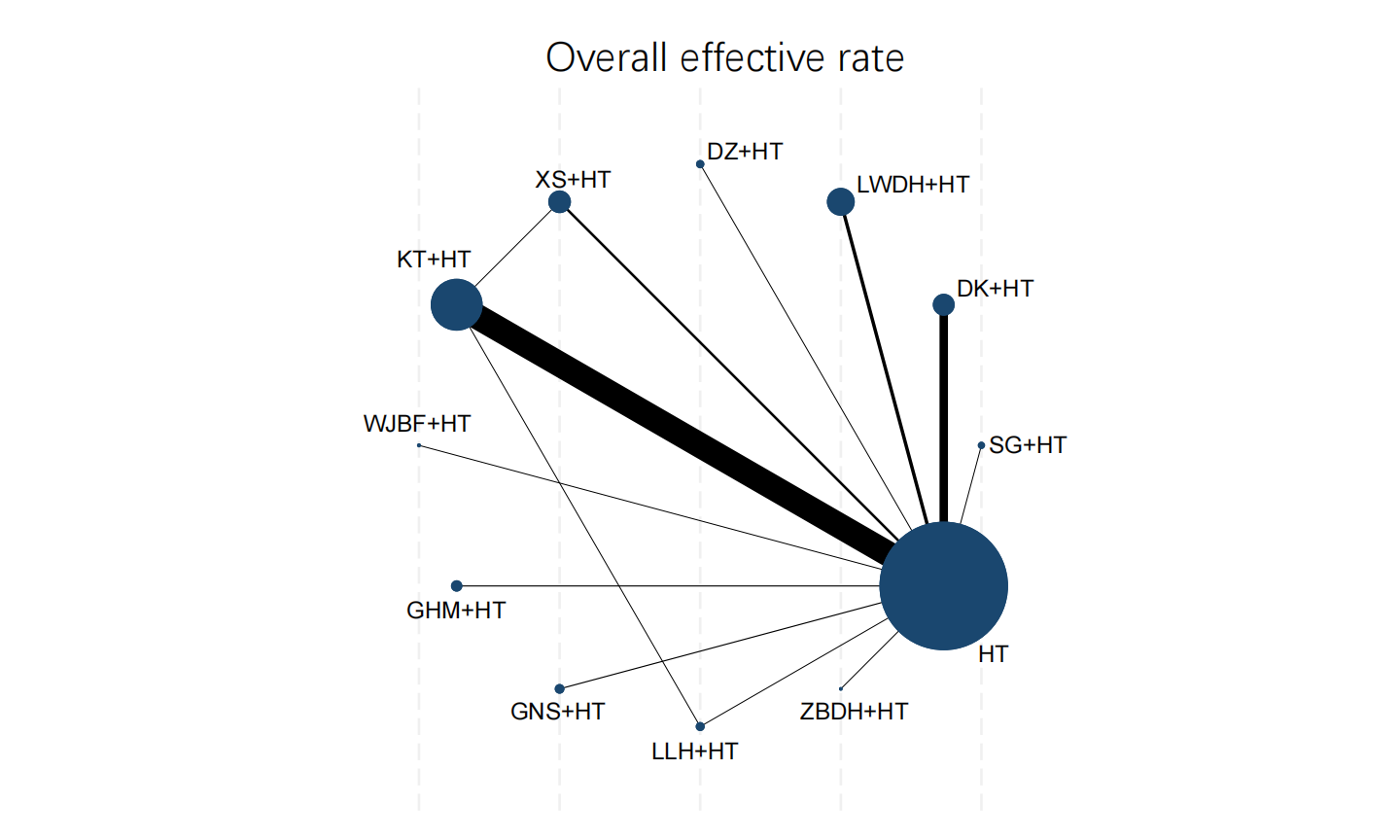


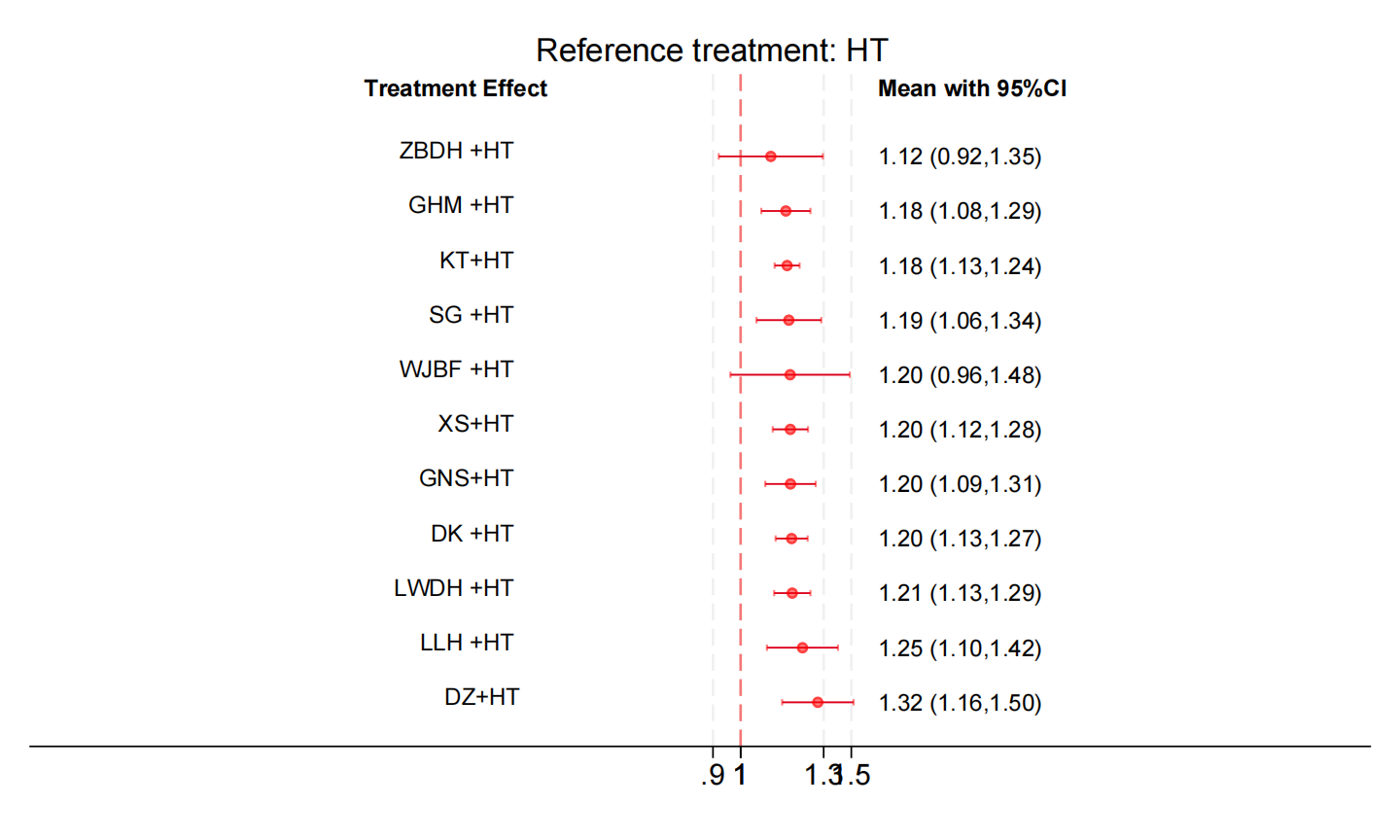


## Figure S6.7 Network map of the adverse reactions, and forest plot of network effect sizes compared with HT.


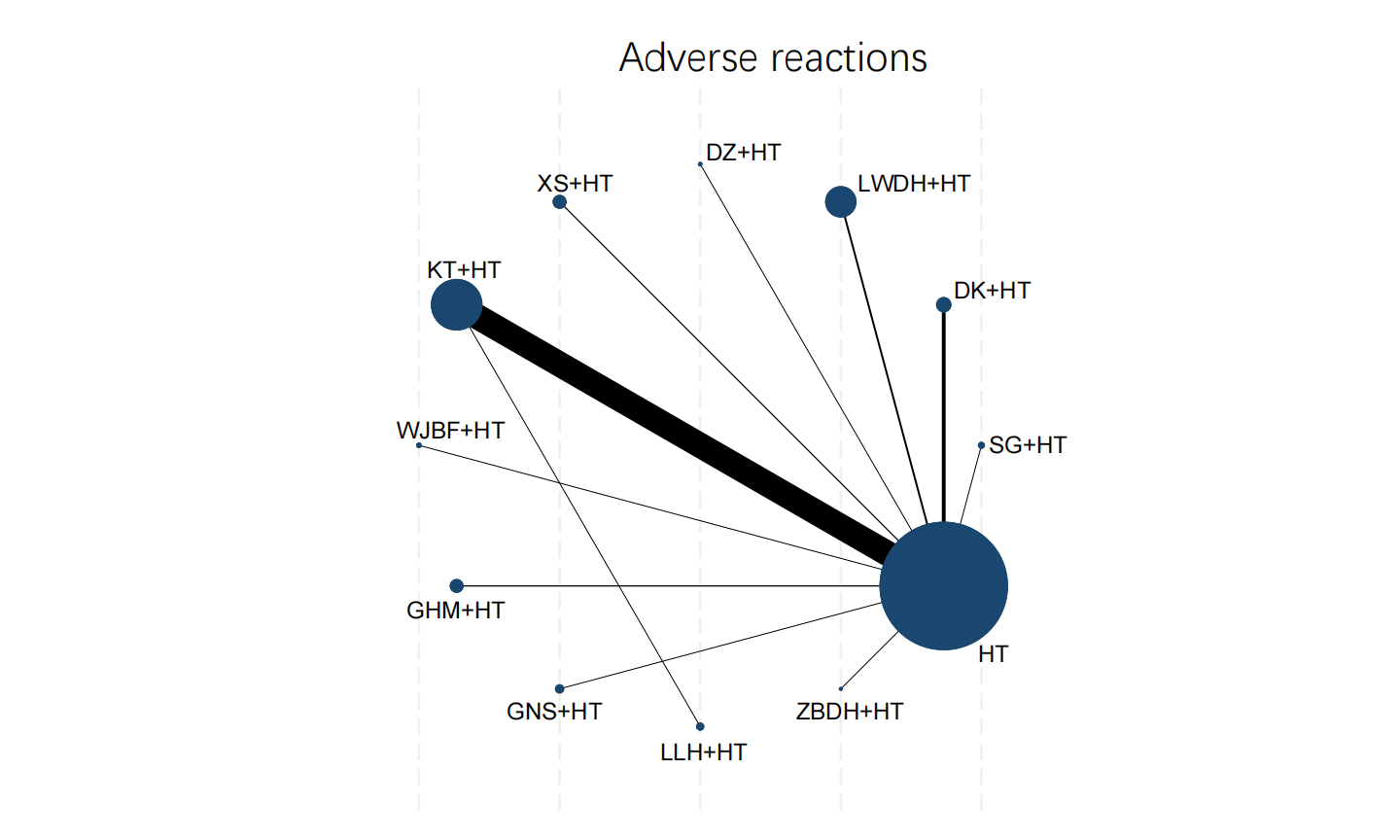


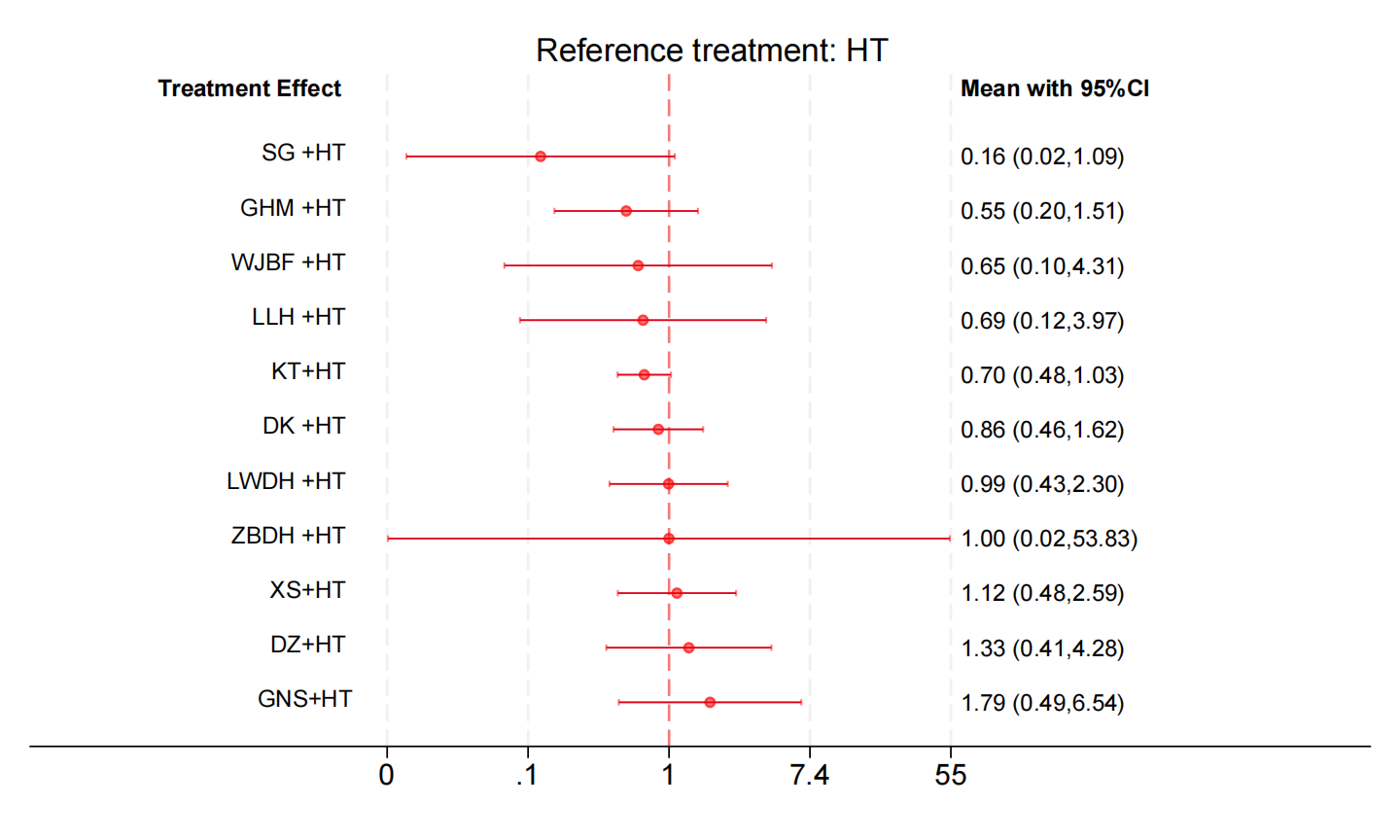


## Figure S6.8 Network map of the Endometrial Thickness, and forest plot of network effect sizes compared with HT.


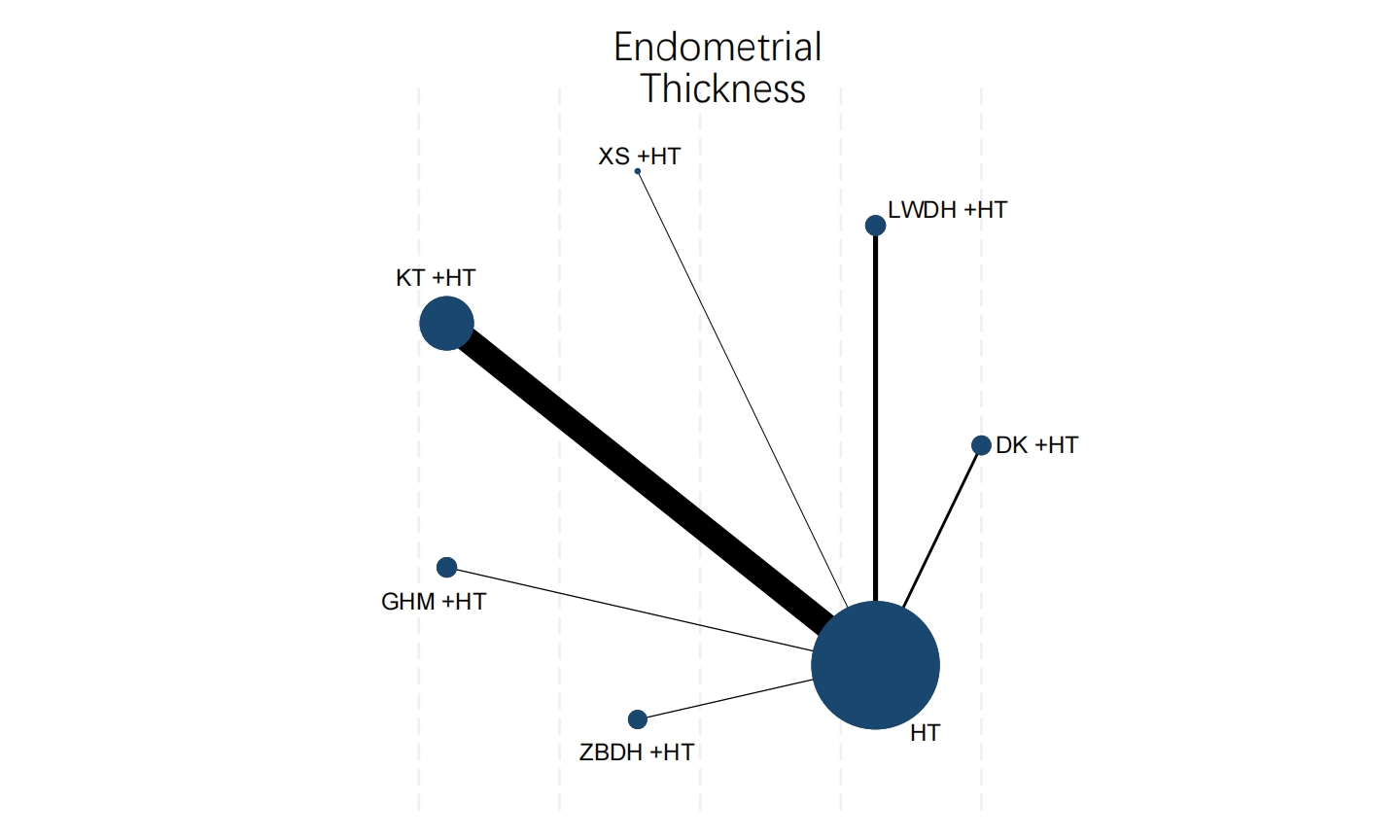


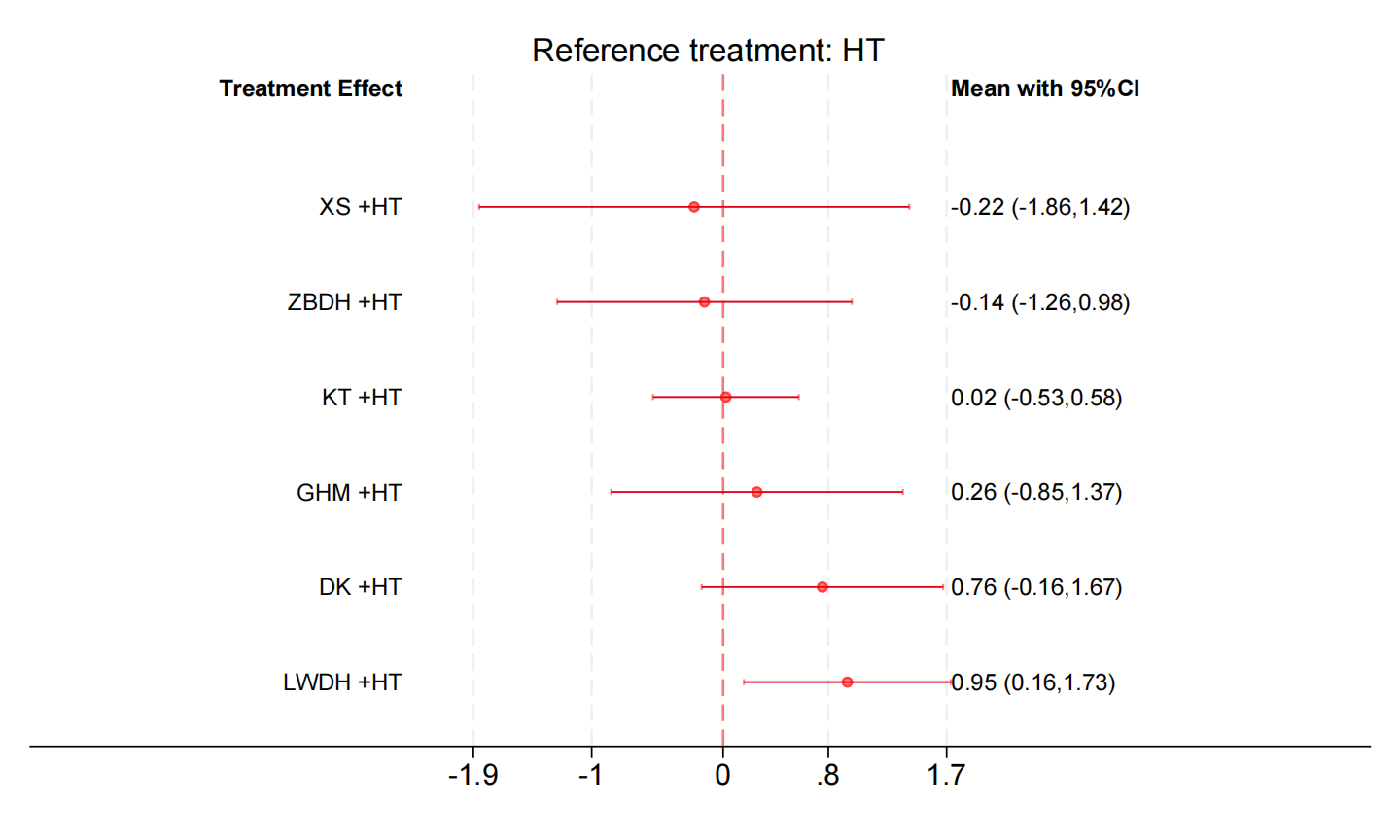


Appendix 7. SUCRA and cumulative probability plots

## Figure S7.1 SUCRA and cumulative ranking probability plot for KI


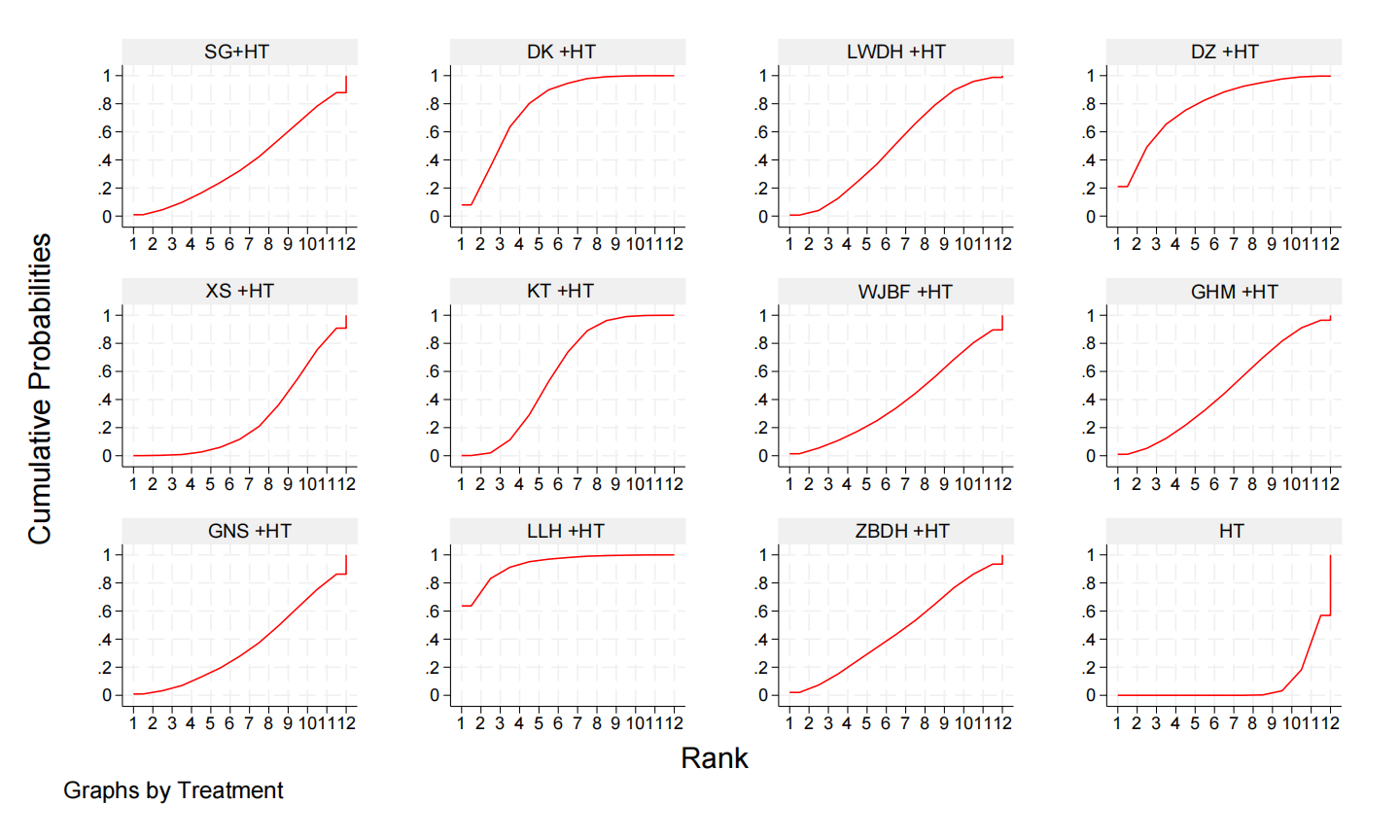


## Table S7.1 SUCRA values for KI

| **Treatment** | **SUCRA** | **PrBest** | **MeanRank** |
| --- | --- | --- | --- |
| SG +HT | 37.9 | 1 | 7.8 |
| DK +HT | 79 | 8 | 3.3 |
| LWDH +HT | 50.9 | 0.7 | 6.4 |
| DZ +HT | 78.7 | 21 | 3.3 |
| XS +HT | 27.3 | 0.1 | 9 |
| KT +HT | 59.4 | 0.1 | 5.5 |
| WJBF +HT | 39.4 | 1.4 | 7.7 |
| GHM +HT | 46.6 | 1 | 6.9 |
| GNS +HT | 34.8 | 0.9 | 8.2 |
| LLH +HT | 93.3 | 63.7 | 1.7 |
| ZBDH +HT | 45.6 | 2.1 | 7 |
| HT | 7.2 | 0 | 11.2 |

Sparse-evidence nodes were defined as intervention nodes supported by no more than two trials. Rankings involving LLH+HT and WJBF+HT should be interpreted cautiously because sparse evidence may lead to unstable rank estimates.

Warning: The SUCRA rankings for KI are exploratory statistical summaries only and are not suitable for clinical interpretation or treatment selection. KI is a subjective symptom score, most included trials were unblinded, the exact KI version was not consistently reported, several nodes were sparse, and the certainty of evidence was generally low to moderate. High ranking probabilities for sparse nodes should not be interpreted as clinical superiority.

## Figure S7.2 SUCRA and cumulative ranking probability plot for MENQOL
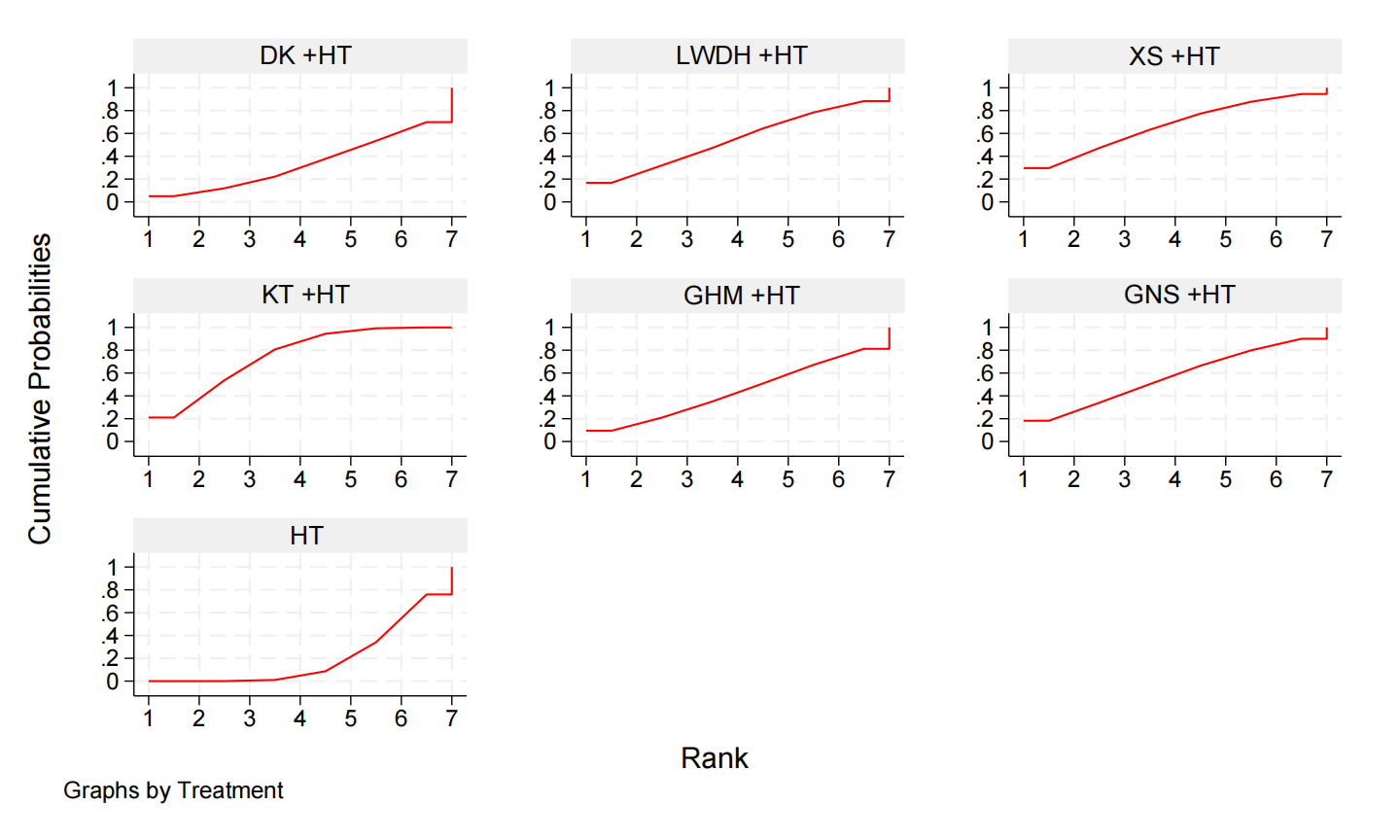


## Table S7.2 SUCRA values for MENQOL

| **Treatment** | **SUCRA** | **PrBest** | **MeanRank** |
| --- | --- | --- | --- |
| DK +HT | 33.4 | 5 | 5 |
| LWDH +HT | 54.5 | 16.7 | 3.7 |
| XS +HT | 66.7 | 29.6 | 3 |
| KT +HT | 74.9 | 21.1 | 2.5 |
| GHM +HT | 44.1 | 9.4 | 4.4 |
| GNS +HT | 56.5 | 18.2 | 3.6 |
| HT | 20 | 0 | 5.8 |

## Figure S7.3 SUCRA and cumulative ranking probability plot for the overall effective rate


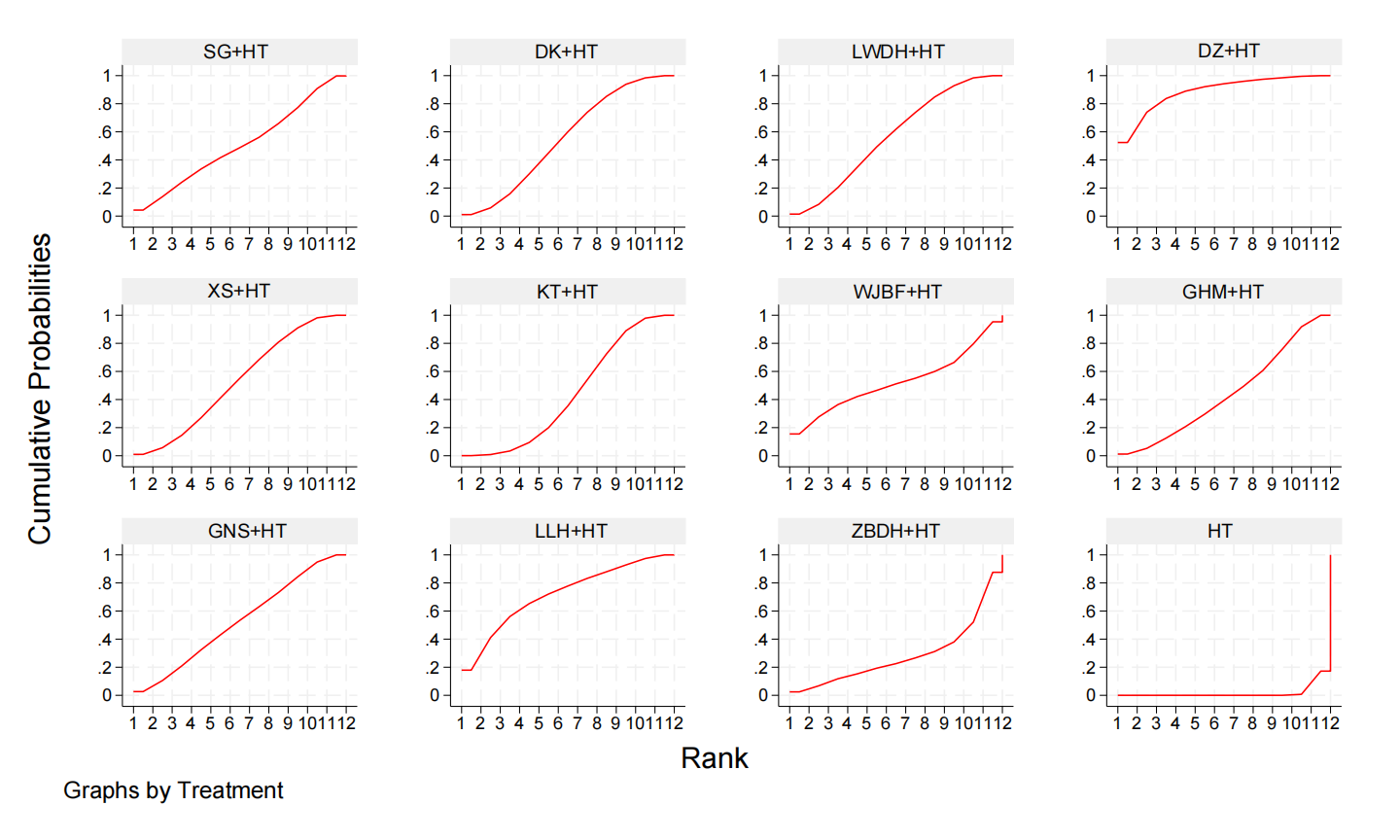


## Table S7.3 SUCRA values for overall effective rate

| **Treatment** | **SUCRA** | **PrBest** | **MeanRank** |
| --- | --- | --- | --- |
| SG +HT | 50.6 | 4.3 | 6.4 |
| DK +HT | 55.4 | 1.1 | 5.9 |
| LWDH +HT | 56.9 | 1.4 | 5.7 |
| DZ +HT | 88.8 | 52.4 | 2.2 |
| XS +HT | 53 | 1 | 6.2 |
| KT +HT | 43.9 | 0.1 | 7.2 |
| WJBF +HT | 52.4 | 15.5 | 6.2 |
| GHM +HT | 44.2 | 1.2 | 7.1 |
| GNS +HT | 52.6 | 2.7 | 6.2 |
| LLH +HT | 72 | 17.9 | 4.1 |
| ZBDH +HT | 28.5 | 2.5 | 8.9 |
| HT | 1.6 | 0 | 11.8 |

Note: The overall effective rate was treated as an auxiliary outcome because its definition varied across trials and often incorporated composite or subjective clinical judgment. Local inconsistency was identified for selected node-splitting comparisons in this network. Therefore, SUCRA rankings for this outcome should be interpreted as descriptive supplementary information and should not be used as a primary basis for comparative clinical recommendations.

## Figure S7.4 SUCRA and cumulative ranking probability plot for E2


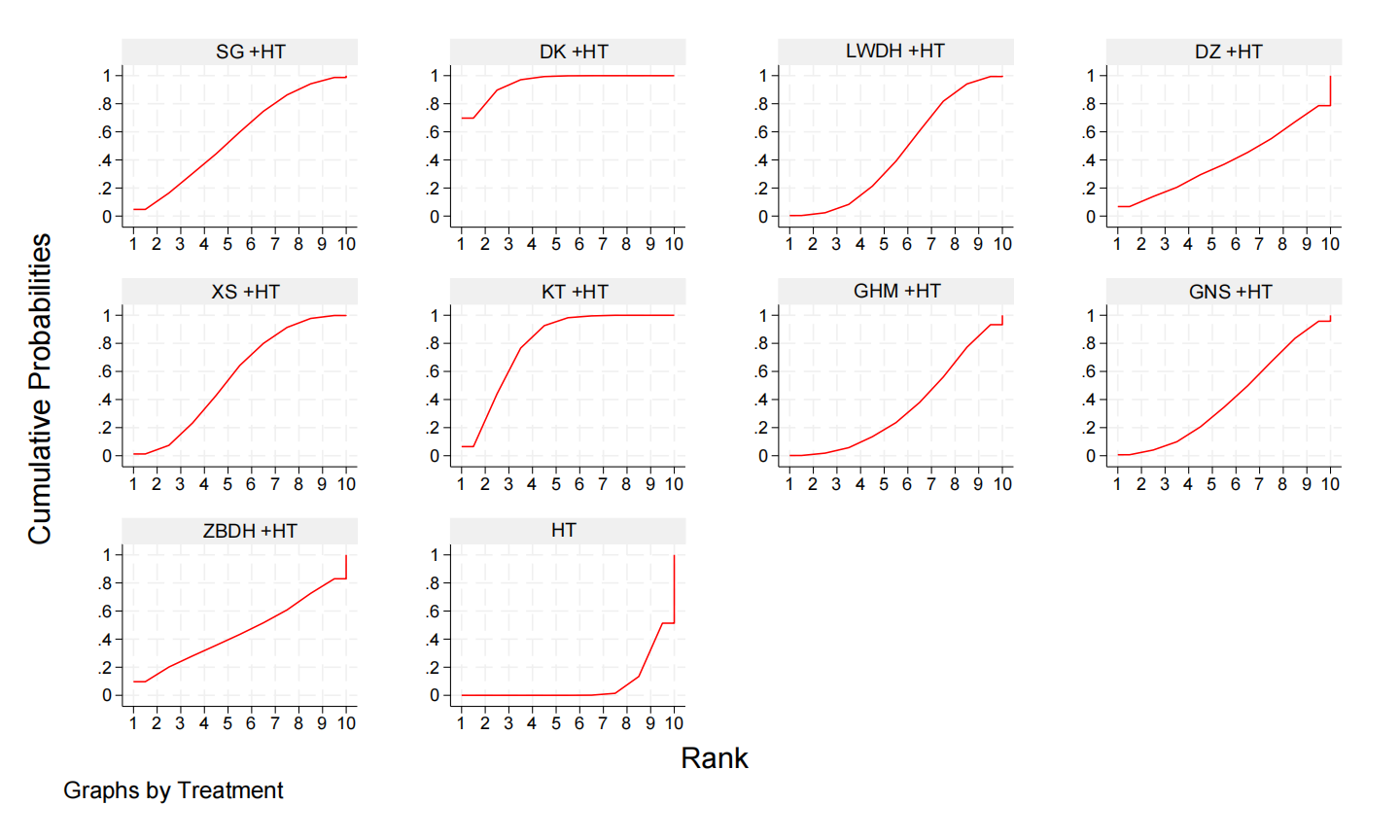


## Table S7.4 SUCRA values for E2

| **Treatment** | **SUCRA** | **PrBest** | **MeanRank** |
| --- | --- | --- | --- |
| SG +HT | 56.6 | 4.7 | 4.9 |
| DK +HT | 95.1 | 69.7 | 1.4 |
| LWDH +HT | 45.3 | 0.4 | 5.9 |
| DZ +HT | 39.3 | 6.8 | 6.5 |
| XS +HT | 56.5 | 1.2 | 4.9 |
| KT +HT | 79.7 | 6.5 | 2.8 |
| GHM +HT | 34.4 | 0.2 | 6.9 |
| GNS +HT | 40.7 | 0.7 | 6.3 |
| ZBDH +HT | 45 | 9.7 | 5.9 |
| HT | 7.4 | 0 | 9.3 |

## Figure S7.5 SUCRA and cumulative ranking probability plot for FSH


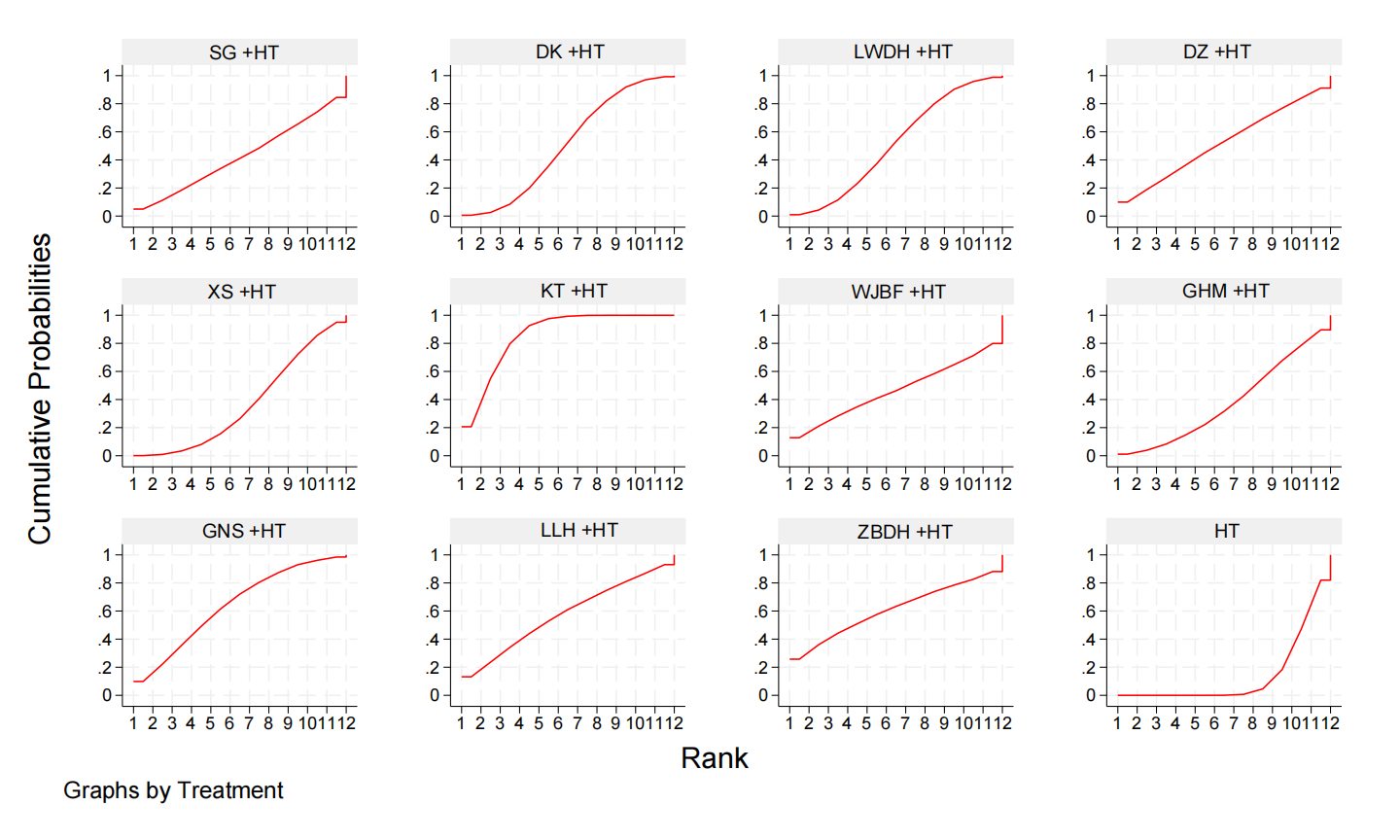


## Table S7.5 SUCRA values for FSH

| **Treatment** | **SUCRA** | **PrBest** | **MeanRank** |
| --- | --- | --- | --- |
| SG +HT | 42.4 | 5 | 7.3 |
| DK +HT | 50.9 | 0.6 | 6.4 |
| LWDH +HT | 51.2 | 1 | 6.4 |
| DZ +HT | 52.1 | 10 | 6.3 |
| XS +HT | 36.8 | 0.1 | 8 |
| KT +HT | 85.9 | 20.6 | 2.6 |
| WJBF +HT | 46.5 | 12.8 | 6.9 |
| GHM +HT | 37.8 | 1.1 | 7.8 |
| GNS +HT | 64.2 | 9.9 | 4.9 |
| LLH +HT | 57.5 | 13.2 | 5.7 |
| ZBDH +HT | 60.9 | 25.7 | 5.3 |
| HT | 13.9 | 0 | 10.5 |

## Figure S7.6 SUCRA and cumulative ranking probability plot for LH


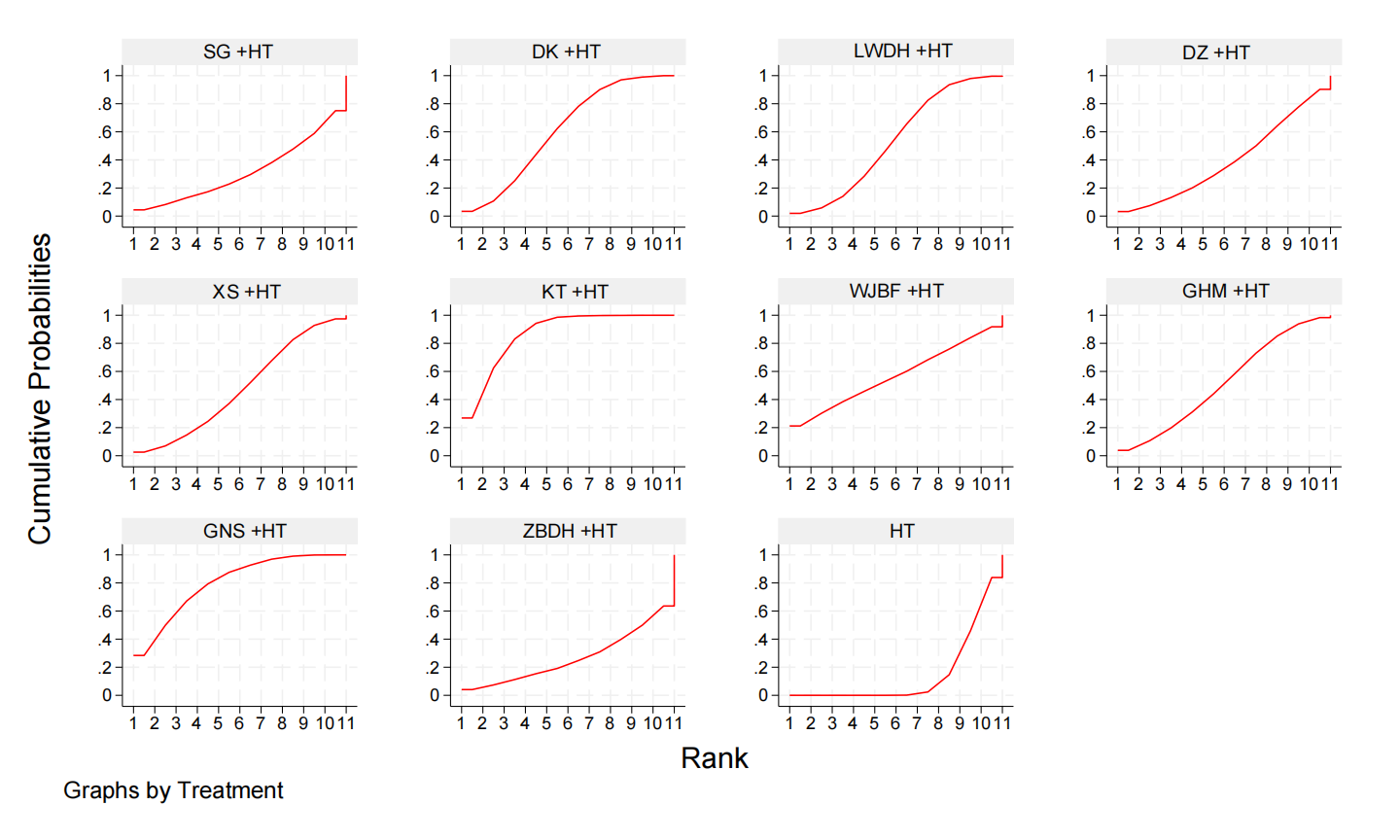


## Table S7.6 SUCRA values for LH

| **Treatment** | **SUCRA** | **PrBest** | **MeanRank** |
| --- | --- | --- | --- |
| SG +HT | 31.5 | 4.4 | 7.8 |
| DK +HT | 61 | 3.4 | 4.9 |
| LWDH +HT | 53.6 | 1.9 | 5.6 |
| DZ +HT | 39.4 | 3.2 | 7.1 |
| XS +HT | 47.9 | 2.5 | 6.2 |
| KT +HT | 86.4 | 26.8 | 2.4 |
| WJBF +HT | 56.9 | 21.2 | 5.3 |
| GHM +HT | 51.8 | 3.8 | 5.8 |
| GNS +HT | 80.1 | 28.4 | 3 |
| ZBDH +HT | 26.7 | 4.1 | 8.3 |
| HT | 14.7 | 0 | 9.5 |

## Figure S7.7 SUCRA and cumulative ranking probability plot for endometrial thickness


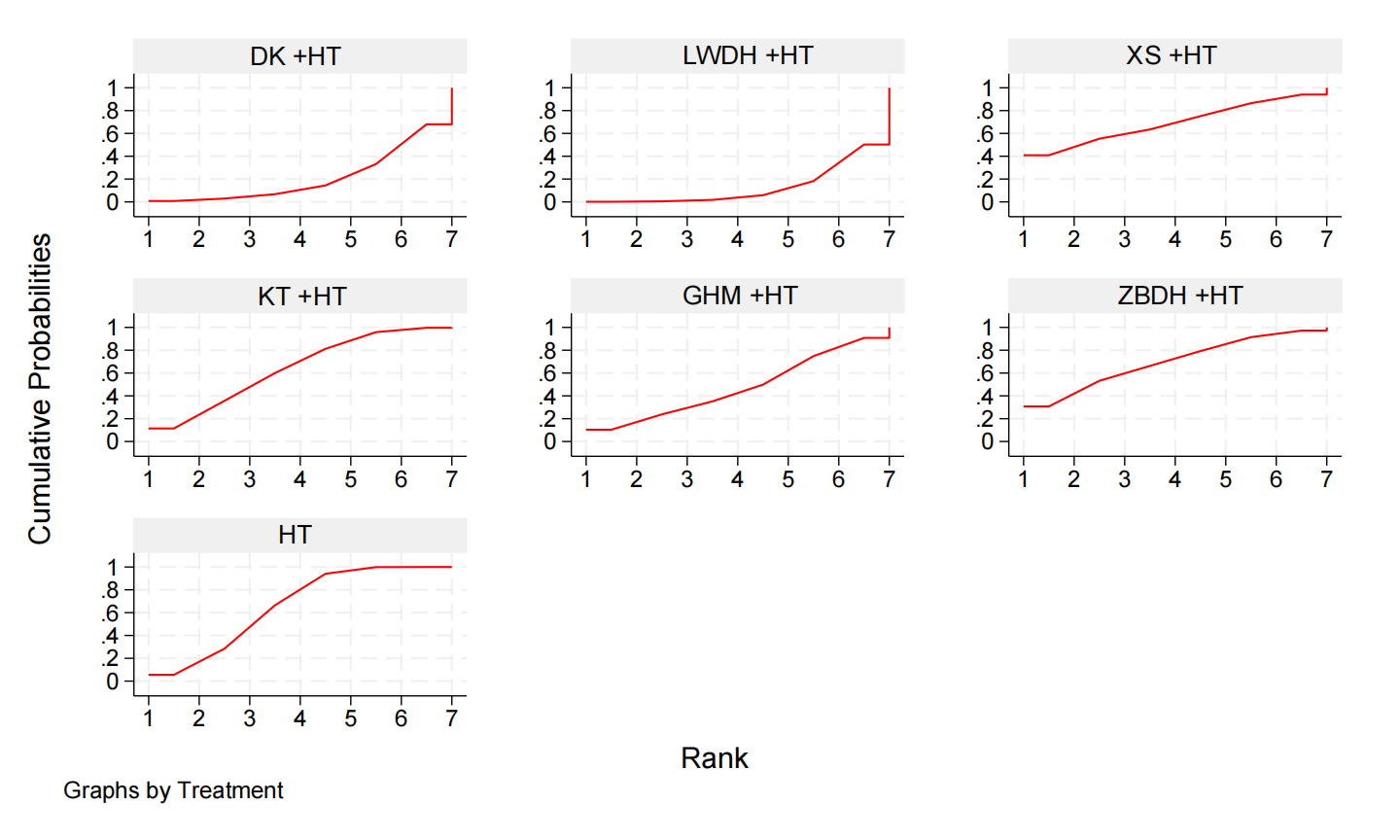


## Table S7.7 SUCRA values for endometrial thickness

| **Treatment** | **SUCRA** | **PrBest** | **MeanRank** |
| --- | --- | --- | --- |
| DK +HT | 21 | 0.8 | 5.7 |
| LWDH +HT | 12.8 | 0.2 | 6.2 |
| XS +HT | 69.3 | 40.9 | 2.8 |
| KT +HT | 64 | 11.5 | 3.2 |
| GHM +HT | 47.5 | 10.4 | 4.2 |
| ZBDH +HT | 69.7 | 30.7 | 2.8 |
| HT | 65.7 | 5.6 | 3.1 |

## Figure S7.8 SUCRA and cumulative ranking probability plot for adverse reactions


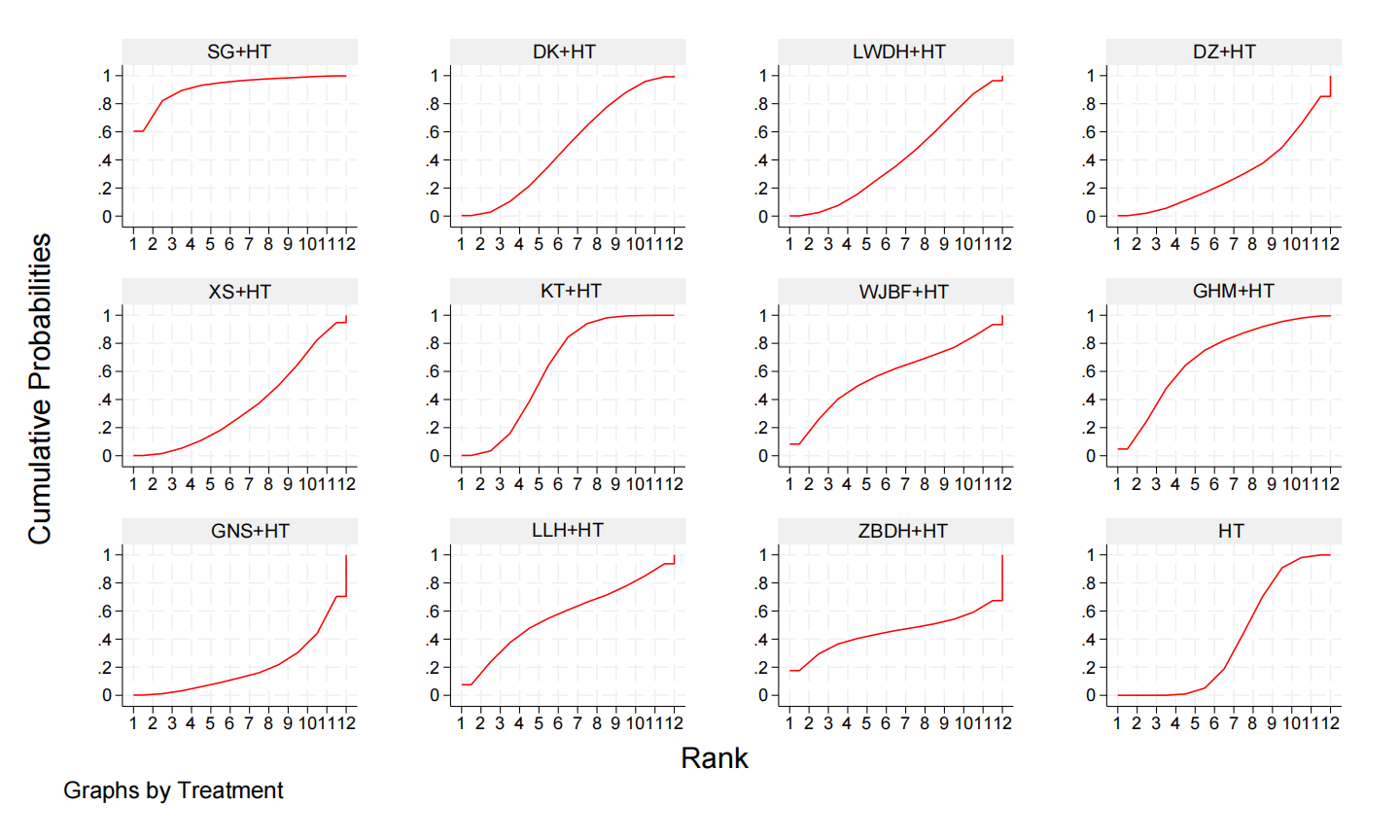


## Table S7.8 SUCRA values for adverse reactions

| **Treatment** | **SUCRA** | **PrBest** | **MeanRank** |
| --- | --- | --- | --- |
| SG +HT | 91.8 | 60.4 | 1.9 |
| DK +HT | 49.7 | 0.4 | 6.5 |
| LWDH +HT | 41 | 0.2 | 7.5 |
| DZ +HT | 29.7 | 0.3 | 8.7 |
| XS +HT | 35.8 | 0.1 | 8.1 |
| KT +HT | 63.5 | 0.2 | 5 |
| WJBF +HT | 57.9 | 8.3 | 5.6 |
| GHM +HT | 70.1 | 4.8 | 4.3 |
| GNS +HT | 19.6 | 0.2 | 9.8 |
| LLH +HT | 57 | 7.6 | 5.7 |
| ZBDH +HT | 44.9 | 17.6 | 7.1 |
| HT | 39 | 0 | 7.7 |

Sparse-evidence nodes were defined as intervention nodes supported by no more than two trials. Rankings involving LLH+HT and WJBF+HT should be interpreted cautiously because sparse evidence may lead to unstable rank estimates.

Appendix 8. League tables of pooled estimates for CCPPs combined with HT versus HT alone in perimenopausal symptoms, derived from network meta-analysis of 73 RCTs

## Table S8.1 League table for KI

| **SG +HT** |  |  |  |  |  |  |  |  |  |  |  |
| --- | --- | --- | --- | --- | --- | --- | --- | --- | --- | --- | --- |
| 1.78 (-1.02,4.57) | DK +HT |  |  |  |  |  |  |  |  |  |  |
| 0.59 (-2.35,3.53) | -1.19 (-3.42,1.03) | LWDH +HT |  |  |  |  |  |  |  |  |  |
| 1.99 (-1.42,5.40) | 0.21 (-2.61,3.03) | 1.40 (-1.56,4.37) | DZ +HT |  |  |  |  |  |  |  |  |
| -0.41 (-3.25,2.44) | **-2.19 (-4.28,-0.09)** | -1.00 (-3.28,1.29) | -2.40 (-5.26,0.47) | XS +HT |  |  |  |  |  |  |  |
| 0.88 (-1.68,3.44) | -0.90 (-2.59,0.79) | 0.29 (-1.63,2.22) | -1.11 (-3.69,1.48) | 1.29 (-0.48,3.06) | KT +HT |  |  |  |  |  |  |
| 0.06 (-3.34,3.46) | -1.72 (-4.52,1.08) | -0.53 (-3.48,2.42) | -1.93 (-5.35,1.49) | 0.47 (-2.38,3.31) | -0.82 (-3.39,1.75) | WJBF +HT |  |  |  |  |  |
| 0.40 (-2.70,3.50) | -1.38 (-3.81,1.05) | -0.19 (-2.79,2.41) | -1.59 (-4.71,1.53) | 0.81 (-1.68,3.29) | -0.48 (-2.65,1.68) | 0.34 (-2.77,3.45) | GHM +HT |  |  |  |  |
| -0.12 (-3.52,3.28) | -1.90 (-4.70,0.90) | -0.71 (-3.66,2.24) | -2.11 (-5.53,1.31) | 0.29 (-2.56,3.14) | -1.00 (-3.57,1.57) | -0.18 (-3.58,3.22) | -0.52 (-3.63,2.59) | GNS +HT |  |  |  |
| 3.11 (-0.34,6.56) | 1.33 (-1.53,4.19) | 2.52 (-0.49,5.53) | 1.12 (-2.35,4.58) | **3.52 (0.61,6.42)** | 2.23 (-0.24,4.70) | 3.05 (-0.40,6.50) | 2.71 (-0.45,5.87) | 3.23 (-0.23,6.68) | LLH +HT |  |  |
| 0.40 (-3.01,3.80) | -1.38 (-4.19,1.42) | -0.19 (-3.15,2.76) | -1.60 (-5.02,1.82) | 0.80 (-2.05,3.65) | -0.49 (-3.06,2.08) | 0.33 (-3.07,3.74) | -0.00 (-3.12,3.11) | 0.51 (-2.89,3.92) | -2.71 (-6.17,0.74) | ZBDH +HT |  |
| -1.25 (-3.65,1.15) | **-3.03 (-4.46,-1.59)** | **-1.84 (-3.54,-0.14)** | **-3.24 (-5.67,-0.81)** | -0.84 (-2.37,0.68) | **-2.13 (-3.03,-1.23)** | -1.31 (-3.72,1.10) | -1.65 (-3.62,0.32) | -1.13 (-3.54,1.28) | **-4.36 (-6.83,-1.88)** | -1.64 (-4.05,0.77) | HT |

Comparisons involving sparse-evidence nodes, particularly LLH+HT and WJBF+HT where applicable, should be interpreted cautiously because estimates may be unstable and may change with future evidence.

## Table S8.2 League table for MENQOL

| **DK +HT** |  |  |  |  |  |  |
| --- | --- | --- | --- | --- | --- | --- |
| 0.99 (-3.31,5.30) | LWDH +HT |  |  |  |  |  |
| 1.68 (-2.65,6.00) | 0.68 (-3.63,5.00) | XS +HT |  |  |  |  |
| 1.79 (-1.51,5.08) | 0.79 (-2.49,4.08) | 0.11 (-3.20,3.42) | KT +HT |  |  |  |
| 0.55 (-3.75,4.86) | -0.44 (-4.73,3.85) | -1.13 (-5.44,3.19) | -1.23 (-4.52,2.05) | GHM +HT |  |  |
| 1.11 (-3.22,5.43) | 0.11 (-4.20,4.43) | -0.57 (-4.90,3.76) | -0.68 (-3.99,2.63) | 0.56 (-3.76,4.87) | GNS +HT |  |
| -0.28 (-3.33,2.77) | -1.27 (-4.31,1.76) | -1.96 (-5.02,1.11) | **-2.07 (-3.32,-0.81)** | -0.83 (-3.87,2.20) | -1.39 (-4.45,1.68) | HT |

## Table S8.3 League table for overall effective rate

| **SG+HT** |  |  |  |  |  |  |  |  |  |  |  |
| --- | --- | --- | --- | --- | --- | --- | --- | --- | --- | --- | --- |
| 0.99 (0.87,1.13) | DK+HT |  |  |  |  |  |  |  |  |  |  |
| 0.99 (0.86,1.13) | 1.00 (0.91,1.09) | LWDH+HT |  |  |  |  |  |  |  |  |  |
| 0.90 (0.76,1.07) | 0.91 (0.79,1.05) | 0.91 (0.79,1.05) | DZ+HT |  |  |  |  |  |  |  |  |
| 0.99 (0.87,1.14) | 1.00 (0.92,1.10) | 1.01 (0.92,1.10) | 1.10 (0.96,1.28) | XS+HT |  |  |  |  |  |  |  |
| 1.01 (0.89,1.14) | 1.02 (0.94,1.09) | 1.02 (0.94,1.10) | 1.12 (0.98,1.28) | 1.01 (0.94,1.09) | KT+HT |  |  |  |  |  |  |
| 1.00 (0.78,1.27) | 1.01 (0.80,1.26) | 1.01 (0.80,1.26) | 1.11 (0.86,1.42) | 1.00 (0.80,1.25) | 0.99 (0.79,1.23) | WJBF+HT |  |  |  |  |  |
| 1.01 (0.87,1.17) | 1.02 (0.92,1.14) | 1.02 (0.92,1.14) | 1.12 (0.96,1.31) | 1.02 (0.91,1.13) | 1.00 (0.91,1.11) | 1.02 (0.80,1.28) | GHM+HT |  |  |  |  |
| 0.99 (0.86,1.15) | 1.00 (0.90,1.12) | 1.01 (0.90,1.13) | 1.10 (0.94,1.29) | 1.00 (0.89,1.12) | 0.99 (0.89,1.09) | 1.00 (0.79,1.26) | 0.98 (0.87,1.12) | GNS+HT |  |  |  |
| 0.95 (0.80,1.13) | 0.96 (0.84,1.11) | 0.96 (0.83,1.11) | 1.06 (0.88,1.27) | 0.96 (0.83,1.10) | 0.95 (0.83,1.07) | 0.96 (0.74,1.23) | 0.94 (0.81,1.10) | 0.96 (0.82,1.12) | LLH+HT |  |  |
| 1.07 (0.86,1.33) | 1.08 (0.89,1.31) | 1.08 (0.89,1.32) | 1.19 (0.94,1.49) | 1.07 (0.88,1.31) | 1.06 (0.87,1.29) | 1.07 (0.81,1.43) | 1.06 (0.86,1.30) | 1.07 (0.87,1.32) | 1.12 (0.89,1.41) | ZBDH+HT |  |
| **1.19 (1.06,1.34)** | **1.20 (1.13,1.27)** | **1.21 (1.13,1.29)** | **1.32 (1.16,1.50)** | **1.20 (1.12,1.28)** | **1.18 (1.13,1.24)** | 1.20 (0.96,1.48) | **1.18 (1.08,1.29)** | **1.20 (1.09,1.31)** | **1.25 (1.10,1.42)** | 1.12 (0.92,1.35) | HT |

Note: Because definitions of the overall effective rate were not standardized across trials and local inconsistency was identified in selected node-splitting comparisons, this outcome was analyzed as auxiliary evidence. Comparisons in this table should be interpreted cautiously and should not be considered equivalent to validated symptom-scale outcomes such as KI or MENQOL.

## Table S8.4 League table for E2

| **SG +HT** |  |  |  |  |  |  |  |  |  |
| --- | --- | --- | --- | --- | --- | --- | --- | --- | --- |
| -20.80 (-47.82,6.22) | DK +HT |  |  |  |  |  |  |  |  |
| 4.46 (-22.46,31.39) | **25.26 (4.24,46.28)** | LWDH +HT |  |  |  |  |  |  |  |
| 9.51 (-37.72,56.74) | 30.31 (-13.82,74.44) | 5.05 (-39.01,49.11) | DZ +HT |  |  |  |  |  |  |
| 0.00 (-27.46,27.47) | 20.80 (-0.99,42.60) | -4.46 (-26.12,17.20) | -9.51 (-53.95,34.93) | XS +HT |  |  |  |  |  |
| -9.82 (-34.11,14.48) | 10.98 (-6.60,28.57) | -14.28 (-31.70,3.15) | -19.33 (-61.86,23.21) | -9.82 (-27.49,7.86) | KT +HT |  |  |  |  |
| 10.37 (-20.46,41.21) | **31.17 (5.31,57.03)** | 5.91 (-19.84,31.66) | 0.86 (-45.71,47.43) | 10.37 (-16.01,36.75) | 20.19 (-2.84,43.22) | GHM +HT |  |  |  |
| 7.22 (-23.66,38.11) | **28.02 (2.10,53.95)** | 2.76 (-23.05,28.57) | -2.29 (-48.89,44.31) | 7.22 (-19.23,33.67) | 17.04 (-6.07,40.14) | -3.15 (-33.03,26.74) | GNS +HT |  |  |
| 6.52 (-41.40,54.43) | 27.32 (-17.56,72.19) | 2.05 (-42.76,46.87) | -2.99 (-62.27,56.28) | 6.51 (-38.67,51.69) | 16.33 (-26.98,59.64) | -3.86 (-51.13,43.42) | -0.71 (-48.02,46.60) | ZBDH +HT |  |
| **23.18 (0.15,46.21)** | **43.98 (29.01,58.95)** | **18.72 (3.94,33.50)** | 13.67 (-27.85,55.19) | **23.18 (7.32,39.04)** | **33.00 (23.73,42.26)** | 12.81 (-8.29,33.91) | 15.96 (-5.22,37.14) | 16.67 (-25.65,58.98) | HT |

## Table S8.5 League table for FSH

| **SG +HT** |  |  |  |  |  |  |  |  |  |  |  |
| --- | --- | --- | --- | --- | --- | --- | --- | --- | --- | --- | --- |
| 12.15 (-16.50,20.81) | DK +HT |  |  |  |  |  |  |  |  |  |  |
| 2.16 (-16.87,21.19) | 0.01 (-11.12,11.13) | LWDH +HT |  |  |  |  |  |  |  |  |  |
| 2.74 (-21.14,26.62) | 0.59 (-17.64,18.81) | 0.58 (-18.03,19.19) | DZ +HT |  |  |  |  |  |  |  |  |
| -0.89 (-19.91,18.13) | -3.04 (-14.18,8.09) | -3.05 (-14.79,8.70) | -3.63 (-22.24,14.98) | XS +HT |  |  |  |  |  |  |  |
| 10.32 (-7.54,28.17) | 8.16 (-0.87,17.20) | 8.16 (-1.62,17.94) | 7.58 (-9.86,25.02) | **11.21 (1.76,20.65)** | KT +HT |  |  |  |  |  |  |
| 0.95 (-28.00,29.91) | -1.20 (-25.69,23.30) | -1.21 (-25.98,23.57) | -1.79 (-30.46,26.89) | 1.84 (-22.94,26.63) | -9.36 (-33.28,14.55) | WJBF +HT |  |  |  |  |  |
| -0.80 (-21.54,19.94) | -2.96 (-16.81,10.89) | -2.96 (-17.31,11.38) | -3.54 (-23.90,16.81) | 0.09 (-14.27,14.44) | -11.12 (-23.92,1.68) | -1.76 (-27.88,24.36) | GHM +HT |  |  |  |  |
| 5.36 (-15.47,26.20) | 3.21 (-10.79,17.21) | 3.20 (-11.29,17.69) | 2.62 (-17.83,23.08) | 6.25 (-8.25,20.75) | -4.95 (-17.91,8.00) | 4.41 (-21.79,30.60) | 6.17 (-10.51,22.84) | GNS +HT |  |  |  |
| 4.14 (-20.13,28.42) | 1.99 (-16.82,20.80) | 1.98 (-17.19,21.16) | 1.40 (-22.60,25.41) | 5.03 (-14.07,24.14) | -6.17 (-23.52,11.17) | 3.19 (-25.86,32.24) | 4.95 (-15.93,25.82) | -1.22 (-22.19,19.76) | LLH +HT |  |  |
| 6.02 (-23.66,35.70) | 3.87 (-21.50,29.23) | 3.86 (-21.78,29.50) | 3.28 (-26.14,32.71) | 6.91 (-18.73,32.55) | -4.30 (-29.10,20.51) | 5.07 (-28.60,38.74) | 6.82 (-20.11,33.76) | 0.66 (-26.35,27.67) | 1.88 (-27.91,31.67) | ZBDH +HT |  |
| -5.45 (-22.70,11.80) | **-7.60 (-15.01,-0.19)** | -7.61 (-15.91,0.69) | -8.19 (-24.84,8.46) | -4.56 (-12.88,3.76) | **-15.77 (-20.95,-10.59)** | -6.40 (-29.75,16.95) | -4.65 (-16.35,7.06) | -10.81 (-22.69,1.07) | -9.59 (-26.88,7.70) | -11.47 (-35.73,12.79) | HT |

## Table S8.6 League table for LH

| **SG +HT** |  |  |  |  |  |  |  |  |  |  |
| --- | --- | --- | --- | --- | --- | --- | --- | --- | --- | --- |
| 4.72 (-7.65,17.08) | DK +HT |  |  |  |  |  |  |  |  |  |
| 3.76 (-8.70,16.23) | -0.96 (-7.54,5.62) | LWDH +HT |  |  |  |  |  |  |  |  |
| 1.62 (-12.97,16.21) | -3.10 (-13.14,6.94) | -2.14 (-12.31,8.02) | DZ +HT |  |  |  |  |  |  |  |
| 2.89 (-10.24,16.03) | -1.83 (-9.60,5.95) | -0.87 (-8.80,7.06) | 1.27 (-9.70,12.25) | XS +HT |  |  |  |  |  |  |
| 8.09 (-3.82,20.01) | 3.38 (-2.10,8.85) | 4.33 (-1.37,10.03) | 6.48 (-3.01,15.96) | 5.20 (-1.84,12.24) | KT +HT |  |  |  |  |  |
| 4.23 (-12.92,21.38) | -0.49 (-13.98,13.00) | 0.47 (-13.12,14.05) | 2.61 (-12.95,18.17) | 1.34 (-12.86,15.54) | -3.87 (-16.95,9.22) | WJBF +HT |  |  |  |  |
| 3.56 (-9.62,16.73) | -1.16 (-9.01,6.69) | -0.20 (-8.21,7.80) | 1.94 (-9.08,12.96) | 0.67 (-8.34,9.68) | -4.54 (-11.66,2.59) | -0.67 (-14.91,13.57) | GHM +HT |  |  |  |
| 7.68 (-5.55,20.92) | 2.97 (-4.98,10.92) | 3.92 (-4.18,12.03) | 6.07 (-5.03,17.16) | 4.79 (-4.31,13.89) | -0.41 (-7.65,6.83) | 3.45 (-10.84,17.75) | 4.12 (-5.04,13.29) | GNS +HT |  |  |
| -1.71 (-19.12,15.71) | -6.43 (-20.25,7.40) | -5.47 (-19.39,8.45) | -3.33 (-19.18,12.52) | -4.60 (-19.12,9.92) | -9.80 (-23.23,3.63) | -5.94 (-24.17,12.30) | -5.27 (-19.83,9.29) | -9.39 (-24.01,5.22) | ZBDH +HT |  |
| -1.71 (-13.25,9.82) | **-6.43 (-10.95,-1.91)** | **-5.47 (-10.26,-0.69)** | -3.33 (-12.30,5.64) | -4.60 (-10.93,1.72) | **-9.81 (-12.90,-6.71)** | -5.94 (-18.65,6.77) | -5.27 (-11.69,1.14) | **-9.40 (-15.94,-2.85)** | -0.00 (-13.07,13.07) | HT |

## Table S8.7 League table for endometrial thickness

| **DK +HT** |  |  |  |  |  |  |
| --- | --- | --- | --- | --- | --- | --- |
| -0.19 (-1.40,1.02) | LWDH +HT |  |  |  |  |  |
| 0.98 (-0.90,2.85) | 1.17 (-0.65,2.98) | XS +HT |  |  |  |  |
| 0.73 (-0.34,1.81) | 0.92 (-0.04,1.89) | -0.24 (-1.97,1.49) | KT +HT |  |  |  |
| 0.50 (-0.94,1.94) | 0.69 (-0.67,2.05) | -0.48 (-2.45,1.50) | -0.24 (-1.48,1.00) | GHM +HT |  |  |
| 0.90 (-0.55,2.35) | 1.09 (-0.28,2.46) | -0.08 (-2.06,1.91) | 0.16 (-1.09,1.41) | 0.40 (-1.18,1.98) | ZBDH +HT |  |
| 0.76 (-0.16,1.67) | **0.95 (0.16,1.73)** | -0.22 (-1.86,1.42) | 0.02 (-0.53,0.58) | 0.26 (-0.85,1.37) | -0.14 (-1.26,0.98) | HT |

## Table S8.8 League table for adverse reactions

| **SG +HT** |  |  |  |  |  |  |  |  |  |  |  |
| --- | --- | --- | --- | --- | --- | --- | --- | --- | --- | --- | --- |
| 0.19 (0.03,1.40) | DK +HT |  |  |  |  |  |  |  |  |  |  |
| 0.16 (0.02,1.30) | 0.87 (0.31,2.45) | LWDH +HT |  |  |  |  |  |  |  |  |  |
| 0.12 (0.01,1.13) | 0.65 (0.17,2.44) | 0.75 (0.18,3.15) | DZ +HT |  |  |  |  |  |  |  |  |
| 0.14 (0.02,1.16) | 0.77 (0.27,2.16) | 0.89 (0.27,2.88) | 1.18 (0.28,4.96) | XS +HT |  |  |  |  |  |  |  |
| 0.23 (0.03,1.60) | 1.22 (0.60,2.50) | 1.41 (0.57,3.50) | 1.88 (0.55,6.39) | 1.59 (0.65,3.90) | KT +HT |  |  |  |  |  |  |
| 0.25 (0.02,3.66) | 1.33 (0.18,9.82) | 1.54 (0.19,12.22) | 2.05 (0.22,18.97) | 1.74 (0.22,13.76) | 1.09 (0.16,7.53) | WJBF +HT |  |  |  |  |  |
| 0.30 (0.03,2.56) | 1.58 (0.48,5.19) | 1.82 (0.49,6.78) | 2.43 (0.52,11.40) | 2.06 (0.55,7.61) | 1.29 (0.44,3.79) | 1.18 (0.14,10.16) | GHM +HT |  |  |  |  |
| **0.09 (0.01,0.90)** | 0.48 (0.11,2.03) | 0.56 (0.12,2.59) | 0.74 (0.13,4.22) | 0.63 (0.13,2.92) | 0.39 (0.10,1.52) | 0.36 (0.04,3.57) | 0.30 (0.06,1.58) | GNS +HT |  |  |  |
| 0.23 (0.02,3.09) | 1.24 (0.20,7.86) | 1.44 (0.21,9.88) | 1.91 (0.24,15.56) | 1.62 (0.24,11.08) | 1.02 (0.19,5.58) | 0.93 (0.07,12.22) | 0.79 (0.11,5.89) | 2.58 (0.29,22.65) | LLH +HT |  |  |
| 0.16 (0.00,13.36) | 0.86 (0.02,48.69) | 1.00 (0.02,58.38) | 1.33 (0.02,84.26) | 1.12 (0.02,65.75) | 0.71 (0.01,38.61) | 0.65 (0.01,53.27) | 0.55 (0.01,33.32) | 1.79 (0.03,118.19) | 0.69 (0.01,53.65) | ZBDH +HT |  |
| 0.16 (0.02,1.08) | 0.86 (0.46,1.62) | 0.99 (0.43,2.30) | 1.33 (0.41,4.25) | 1.12 (0.49,2.58) | 0.71 (0.48,1.03) | 0.65 (0.10,4.29) | 0.55 (0.20,1.51) | 1.79 (0.49,6.52) | 0.69 (0.12,3.96) | 1.00 (0.02,53.72) | HT |

Comparisons involving sparse-evidence nodes, particularly LLH+HT and WJBF+HT where applicable, should be interpreted cautiously because estimates may be unstable and may change with future evidence.

Appendix 9. Funnel plots for publication bias

## Figure S9.1 Funnel plot for KI


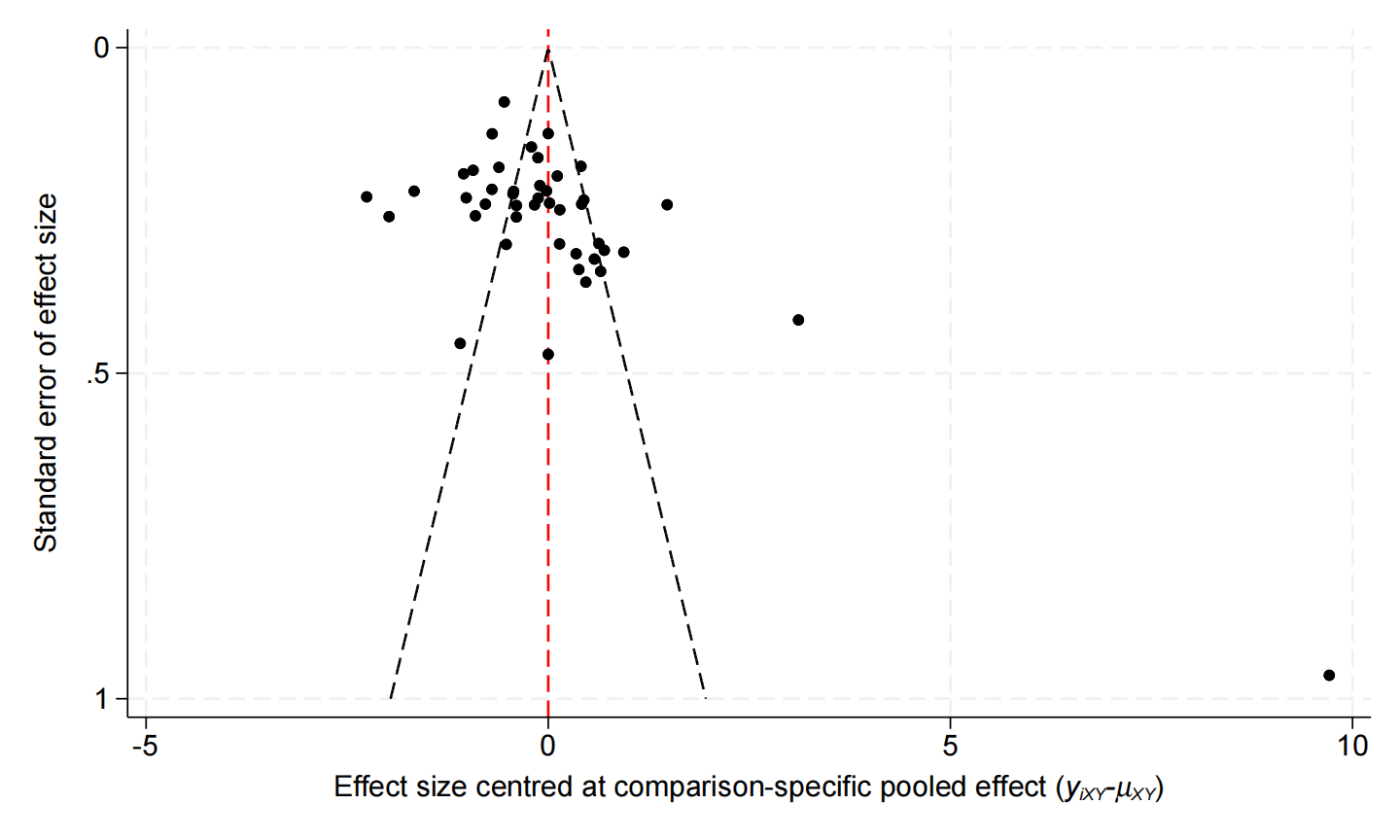


## Figure S9.2 Funnel plot for MENQOL


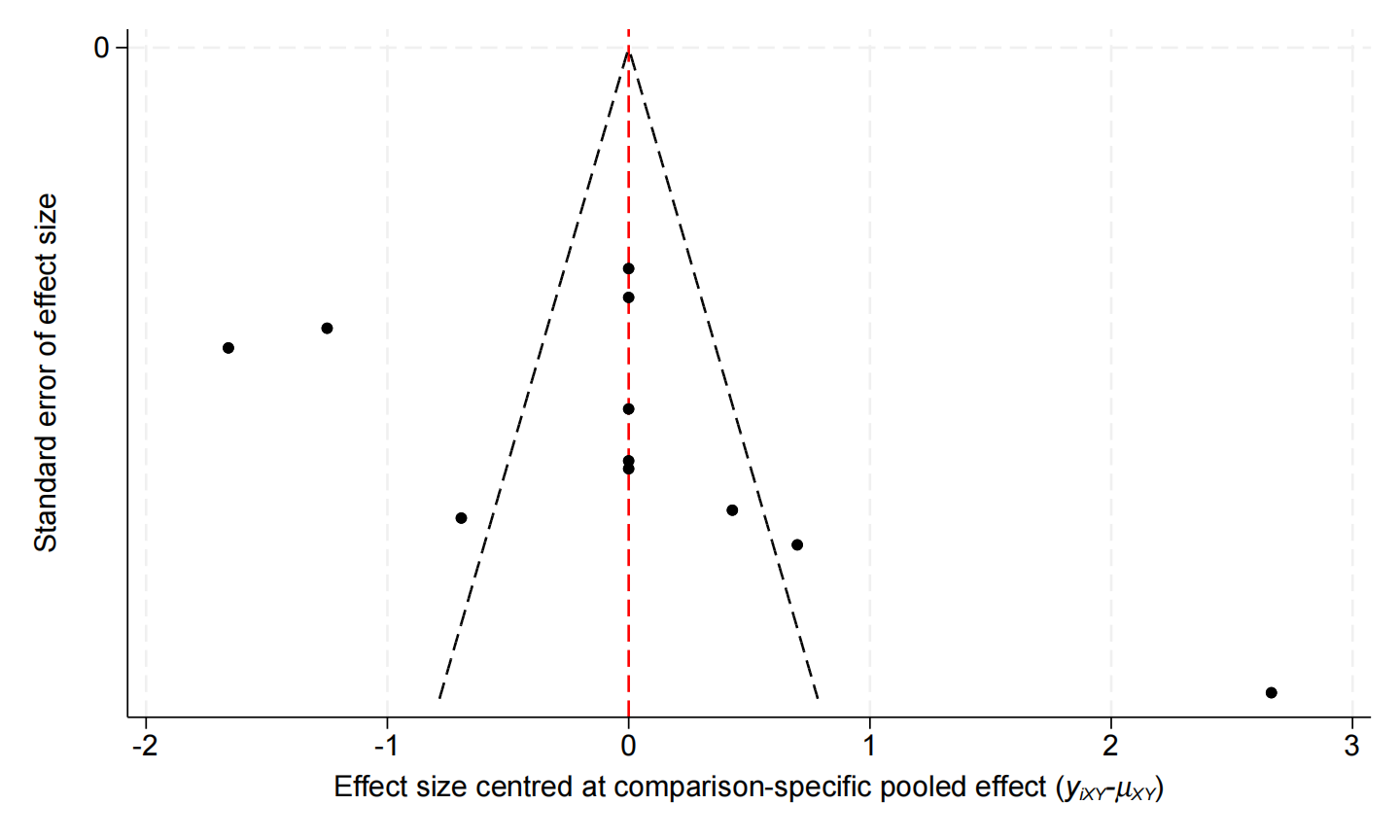


## Figure S9.3 Funnel plot for overall effective rate


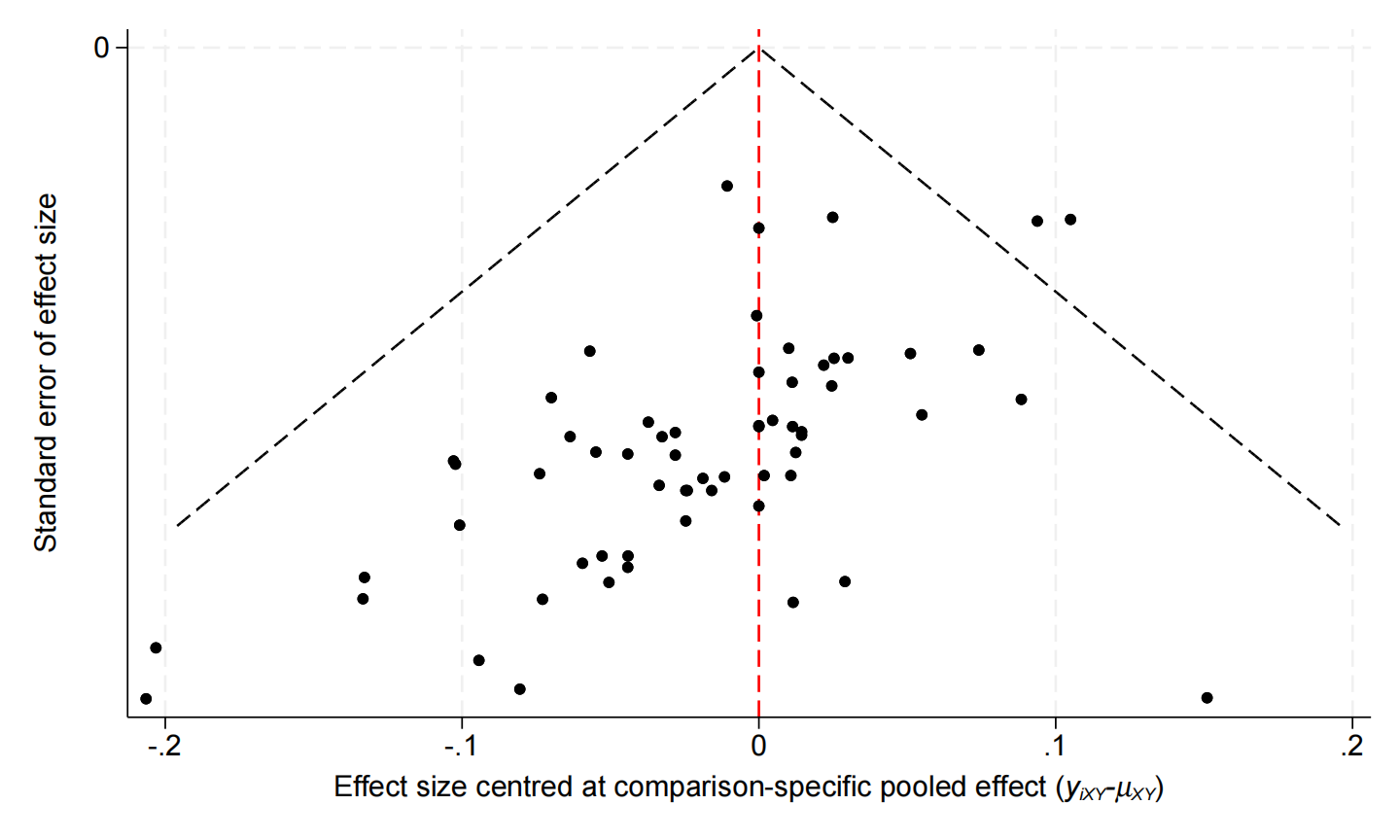


## Figure S9.4 Funnel plot for E2


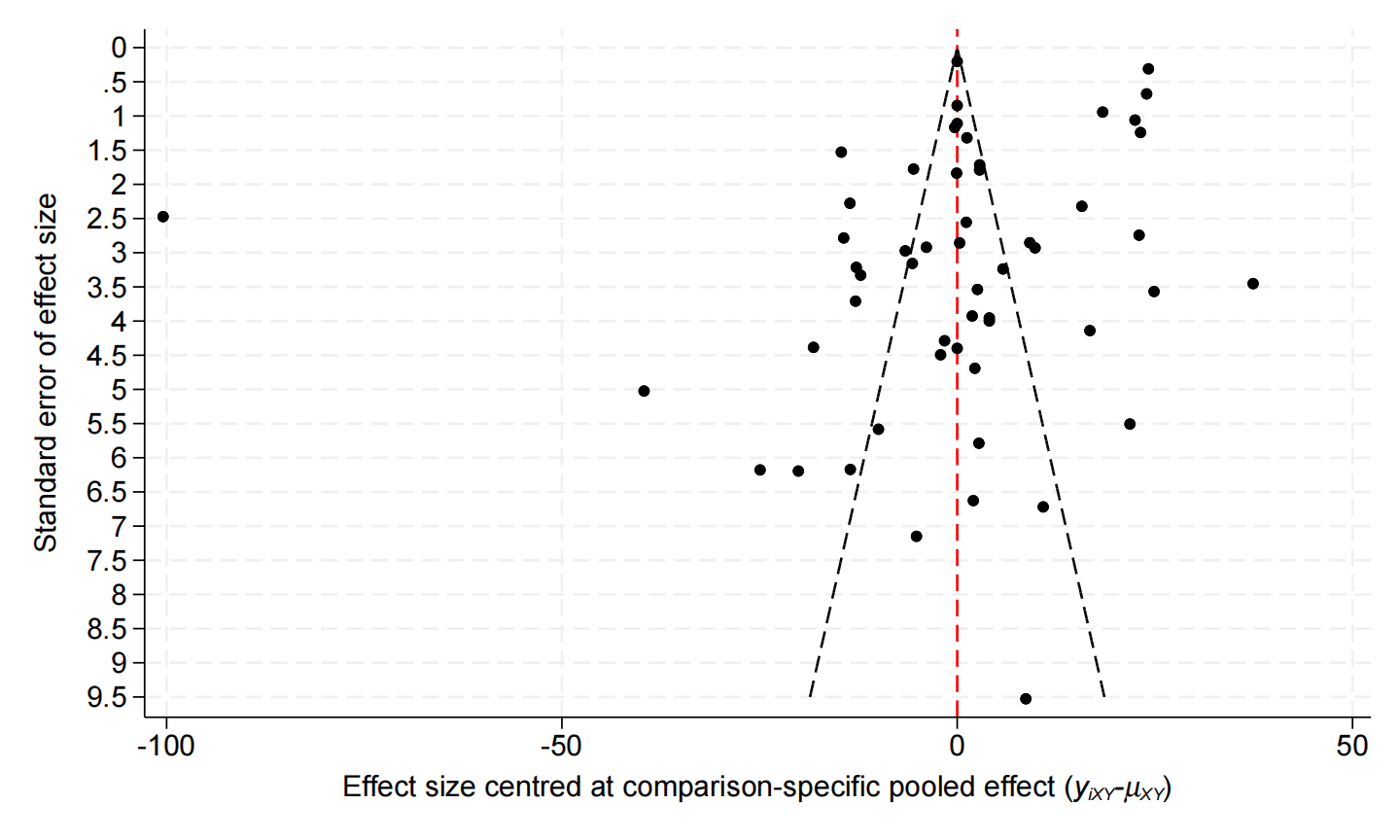


## Figure S9.5 Funnel plot for FSH


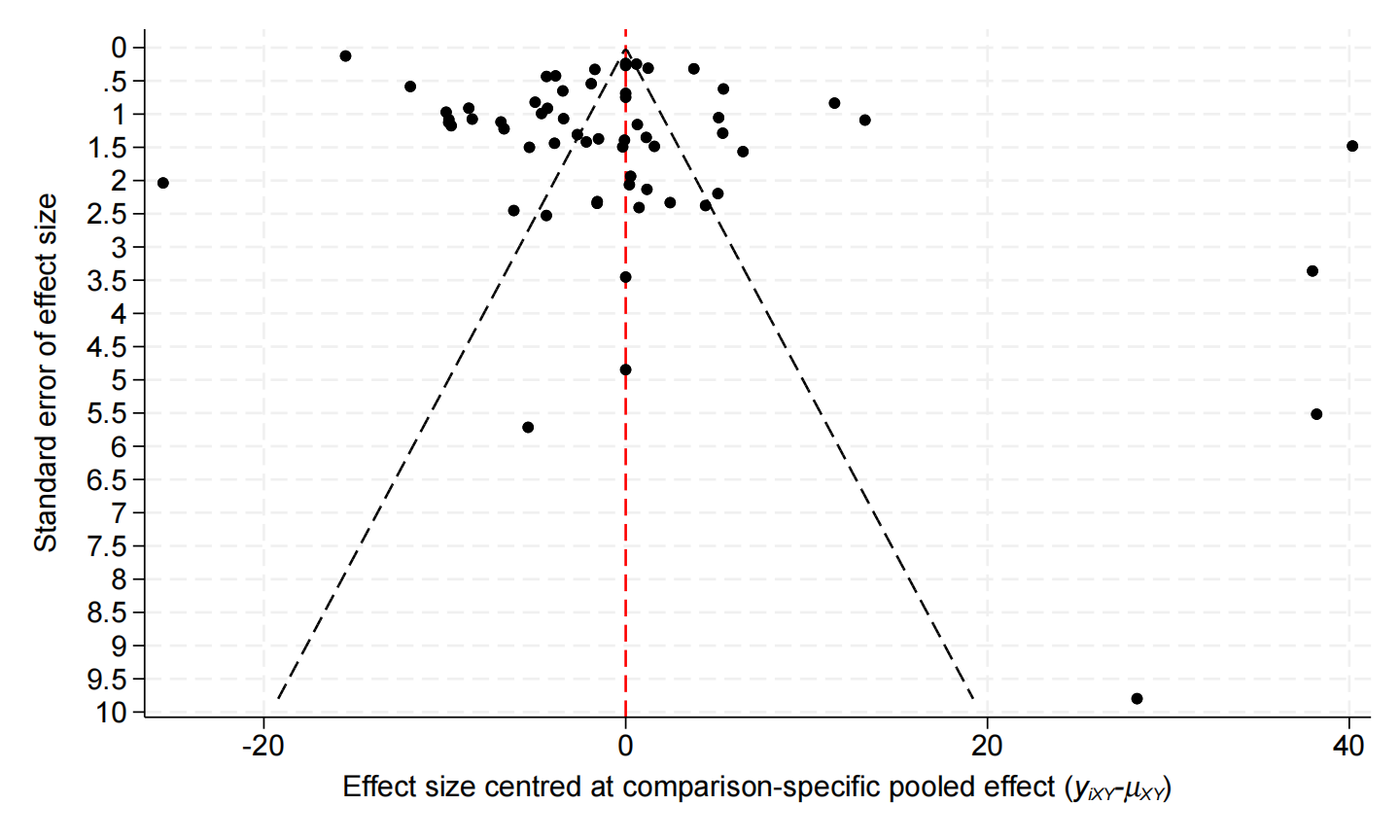


## Figure S9.6 Funnel plot for LH


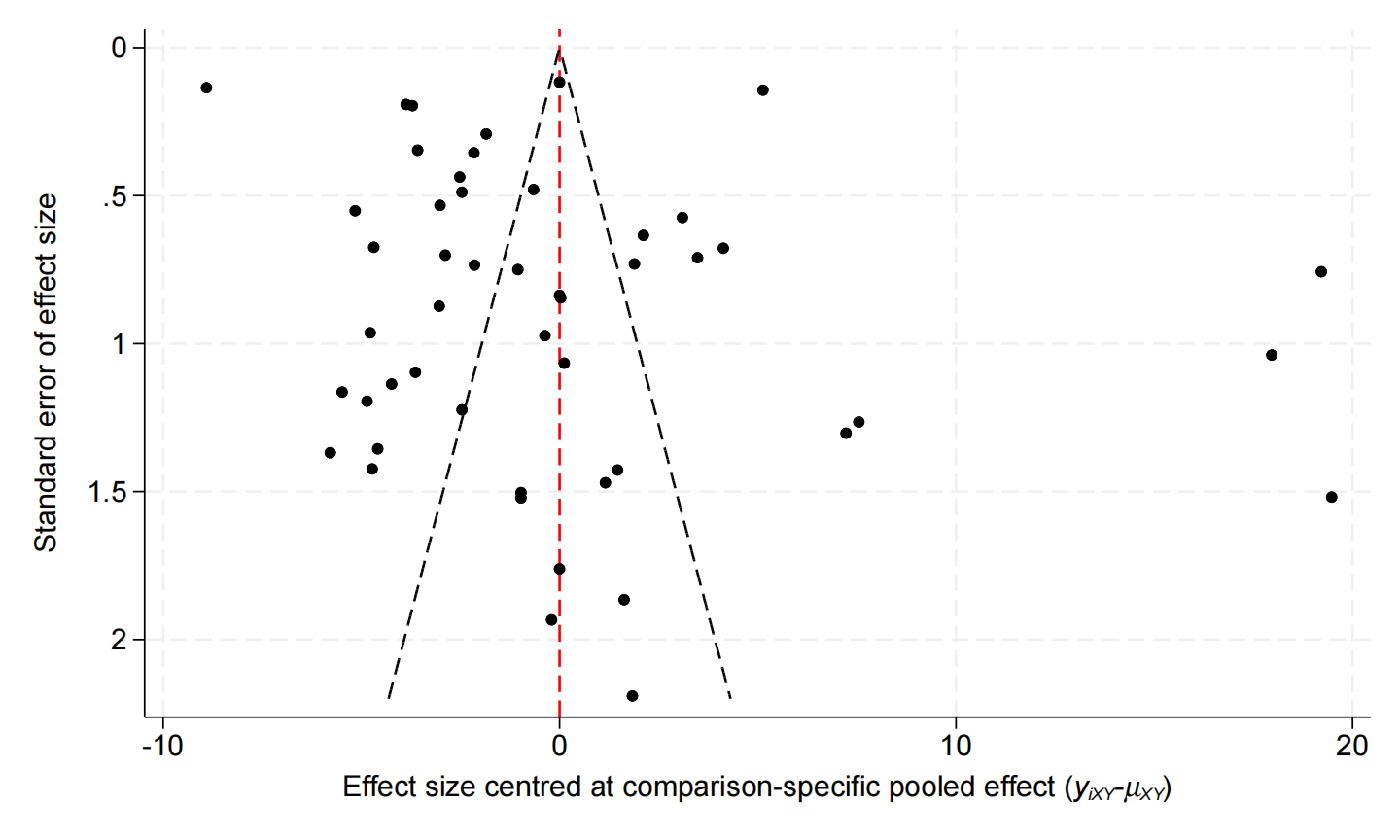


## Figure S9.7 Funnel plot for endometrial thickness


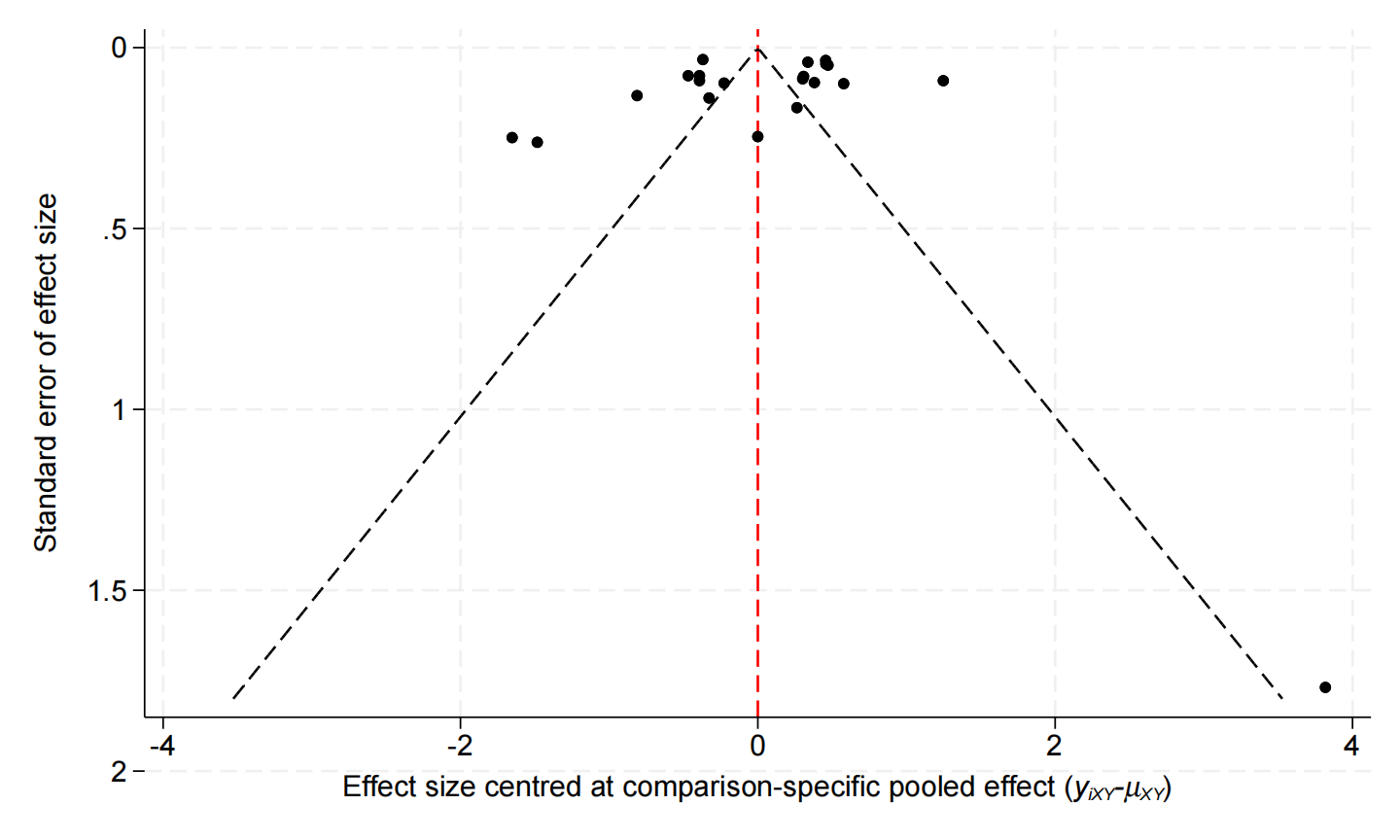


## Figure S9.8 Funnel plot for adverse reactions


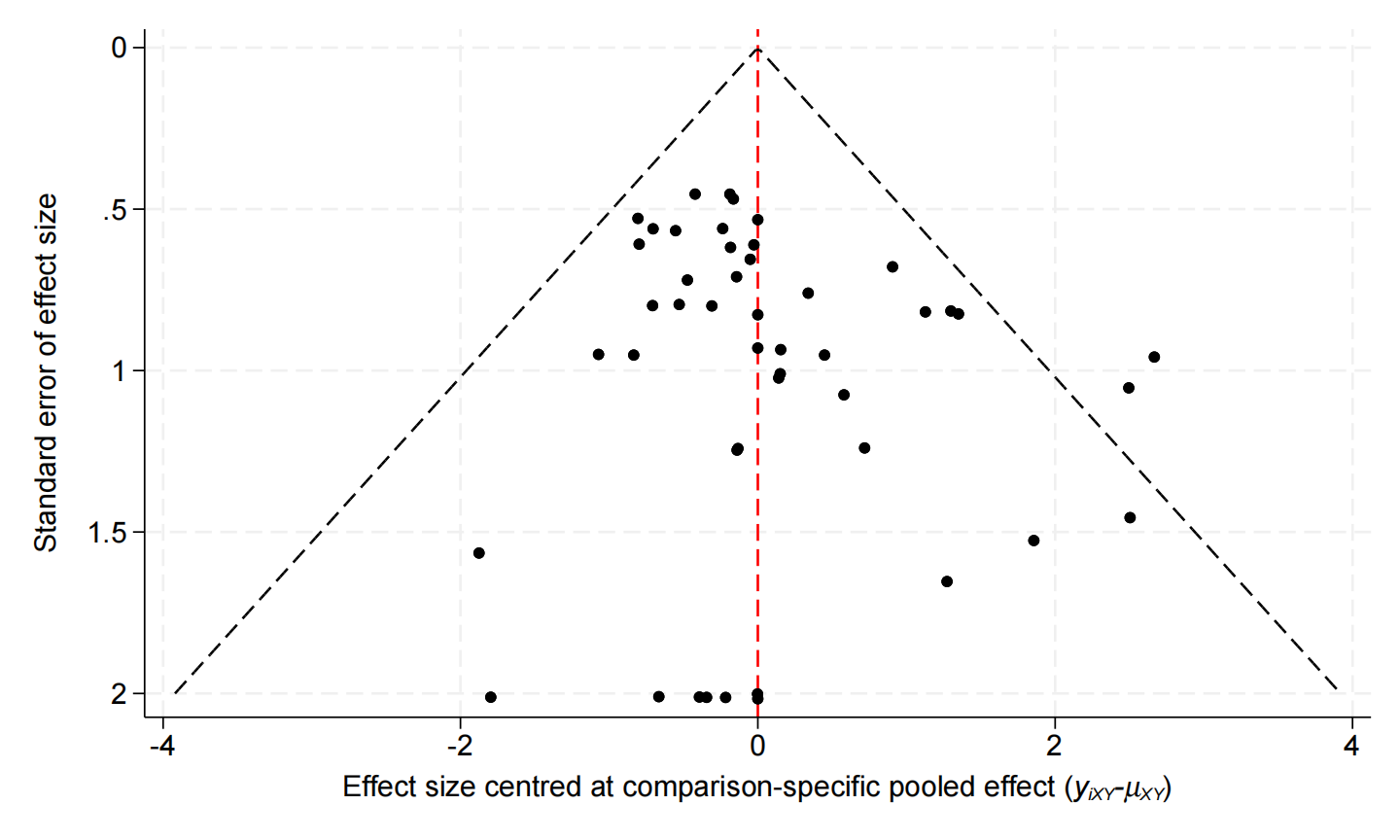


Appendix 10. Subgroup analyses for KI

## Figure S10.1 Forest plot of network meta-analysis results for KI: Short-course subgroup (<3 months)


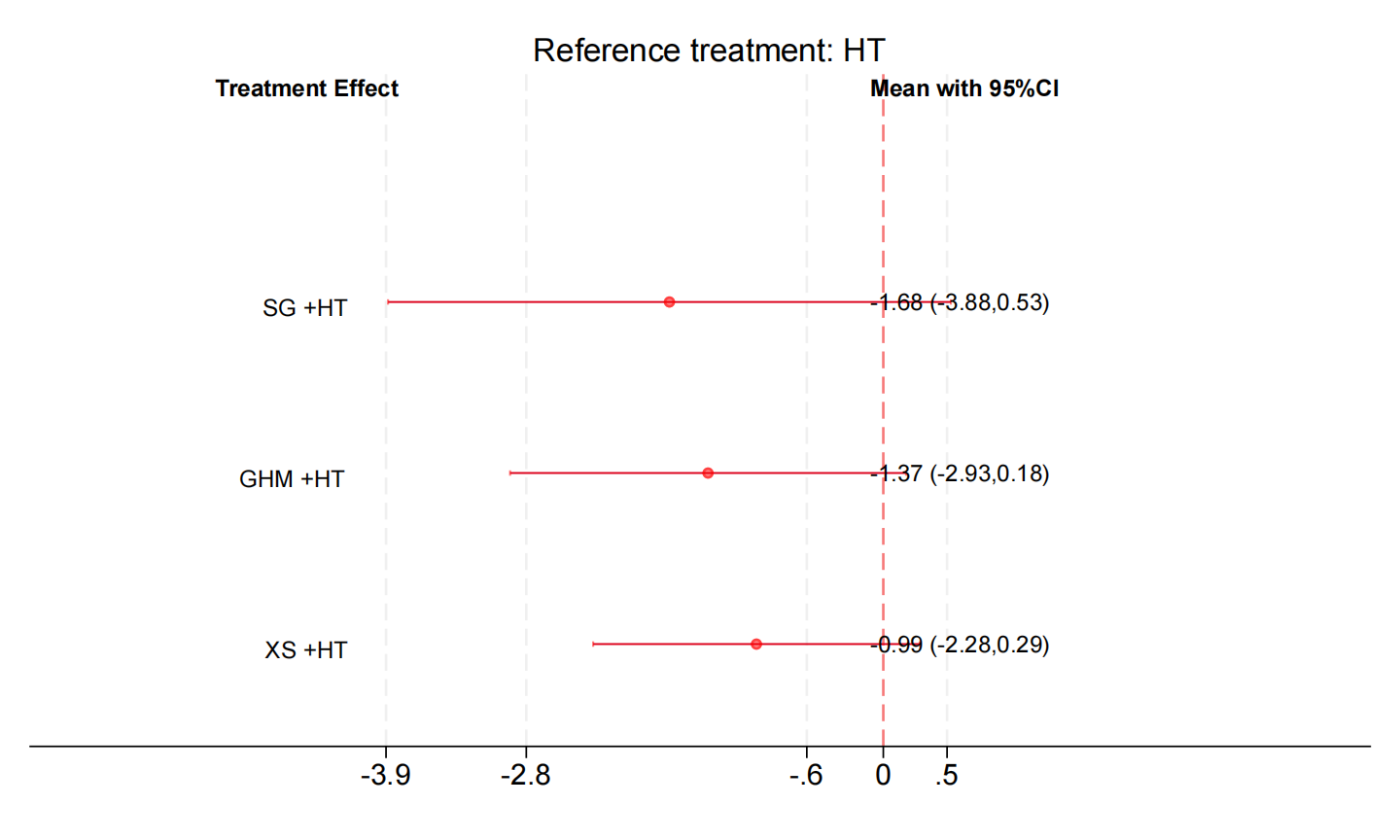


## Figure S10.2 Forest plot of network meta-analysis results for KI: Standard-course subgroup (3 months)


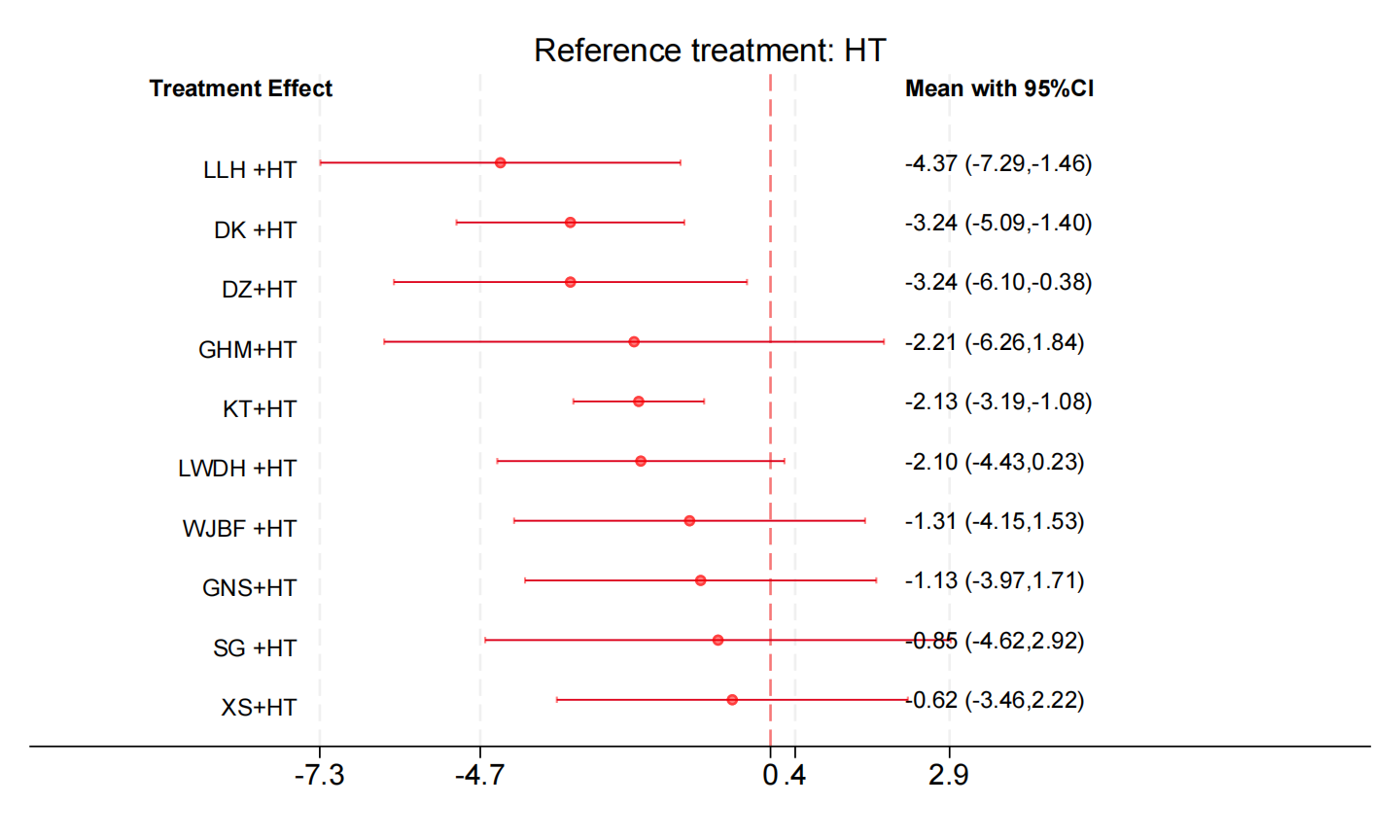


## Figure S10.3 Forest plot of network meta-analysis results for KI: Long-course subgroup (>3 months)


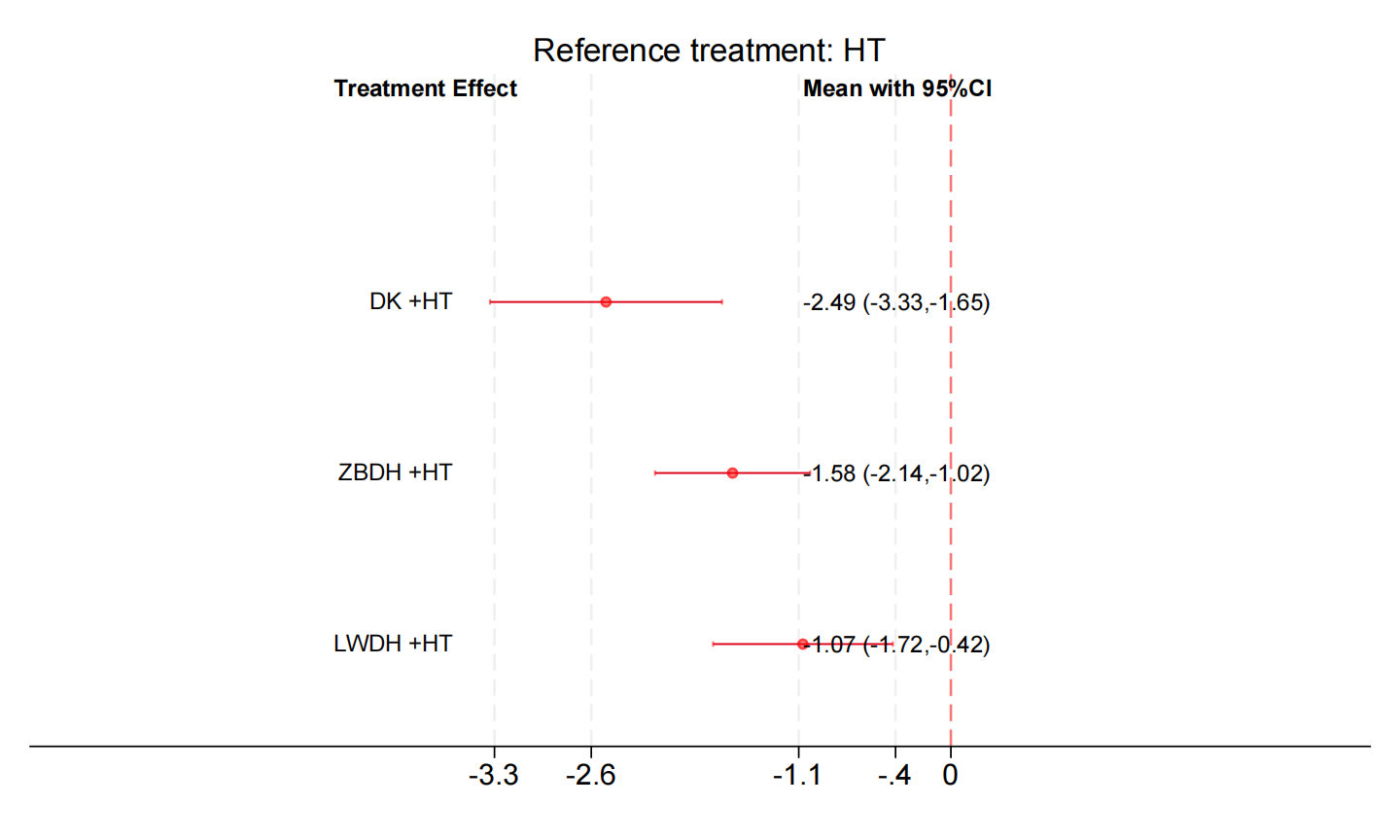


## Figure S10.4 Forest plot of network meta-analysis results for KI: E+P subgroup (estrogen + progestogen)


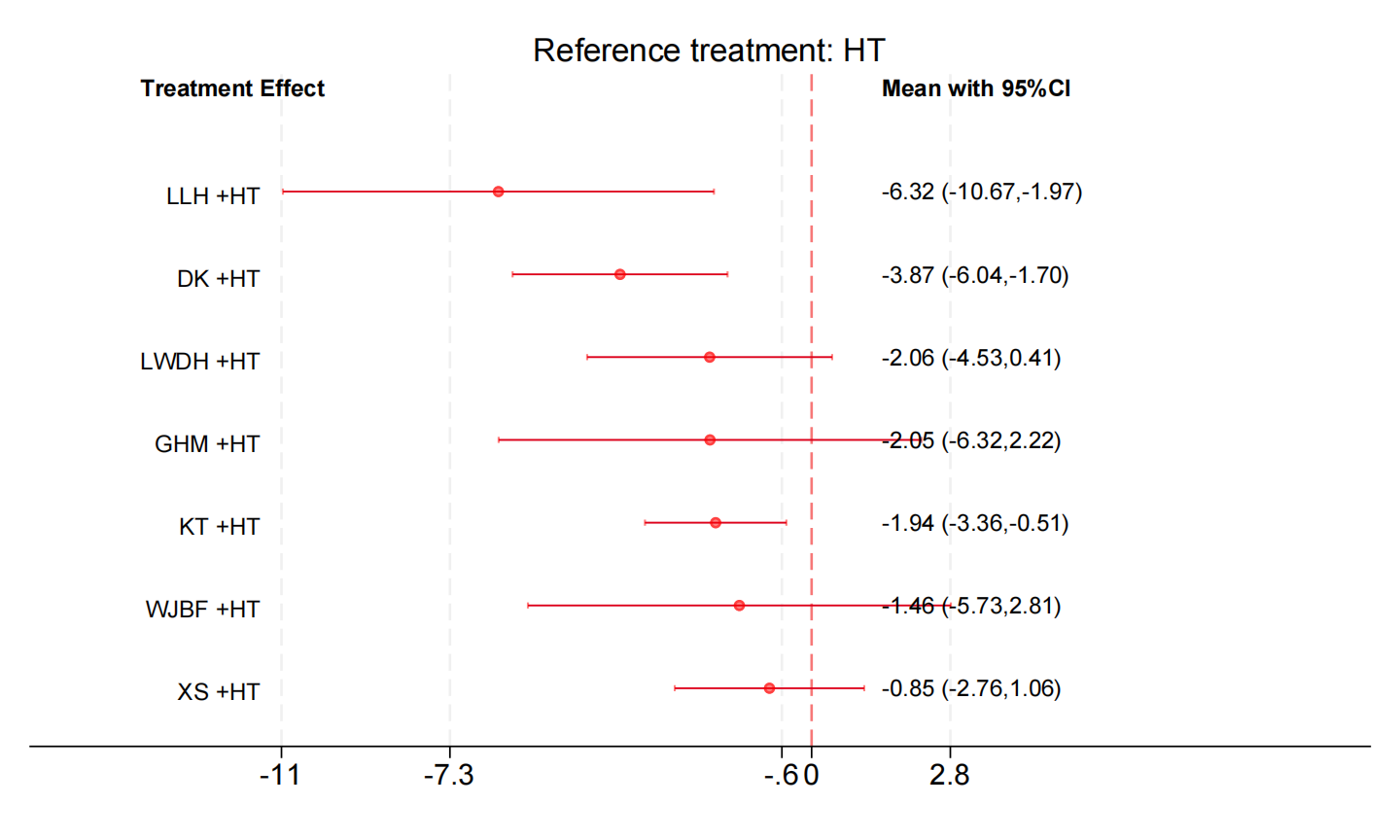


## Figure S10.5 Forest plot of network meta-analysis results for KI: Tibolone subgroup (tibolone)


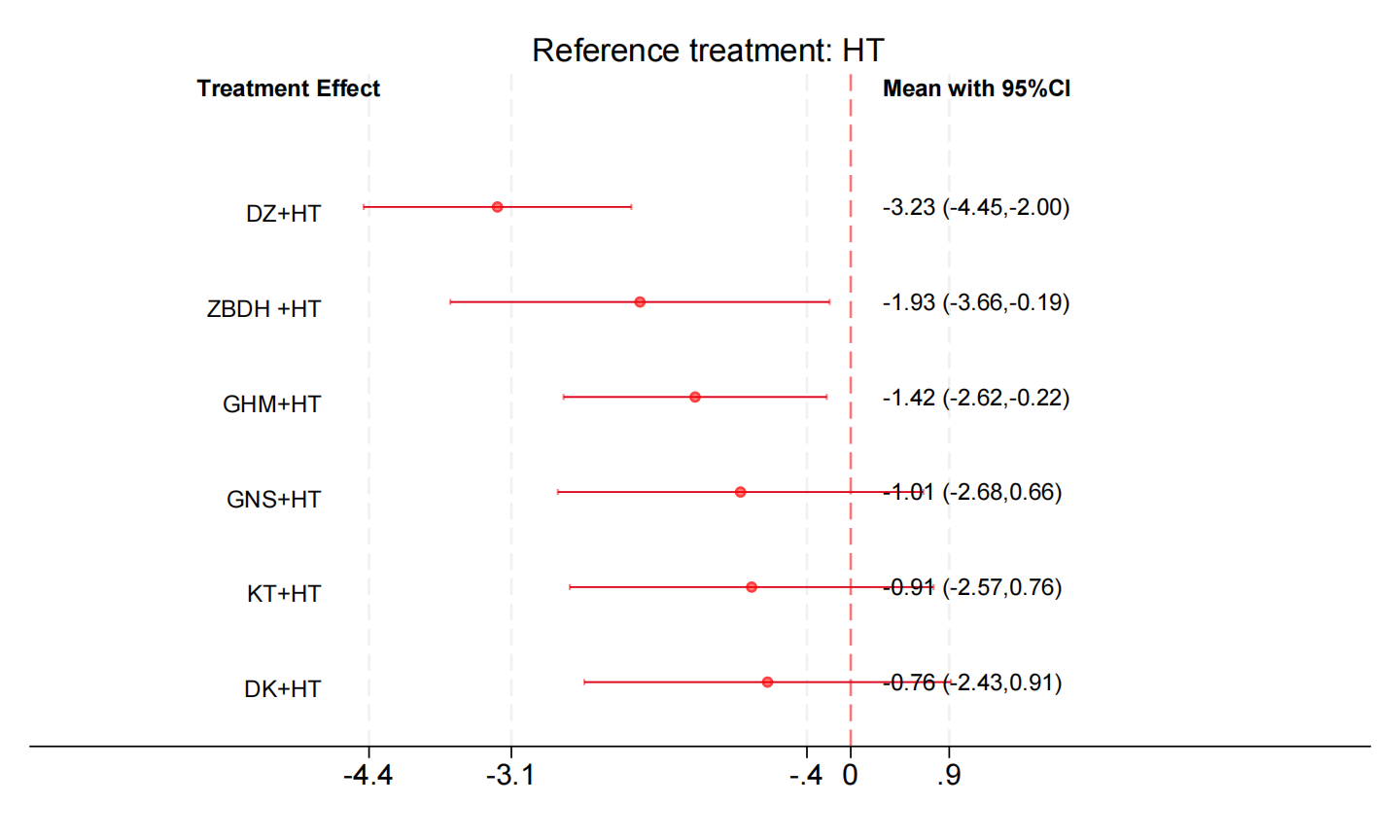


## Figure S10.6 Forest plot of network meta-analysis results for KI: E-only subgroup (estrogen)


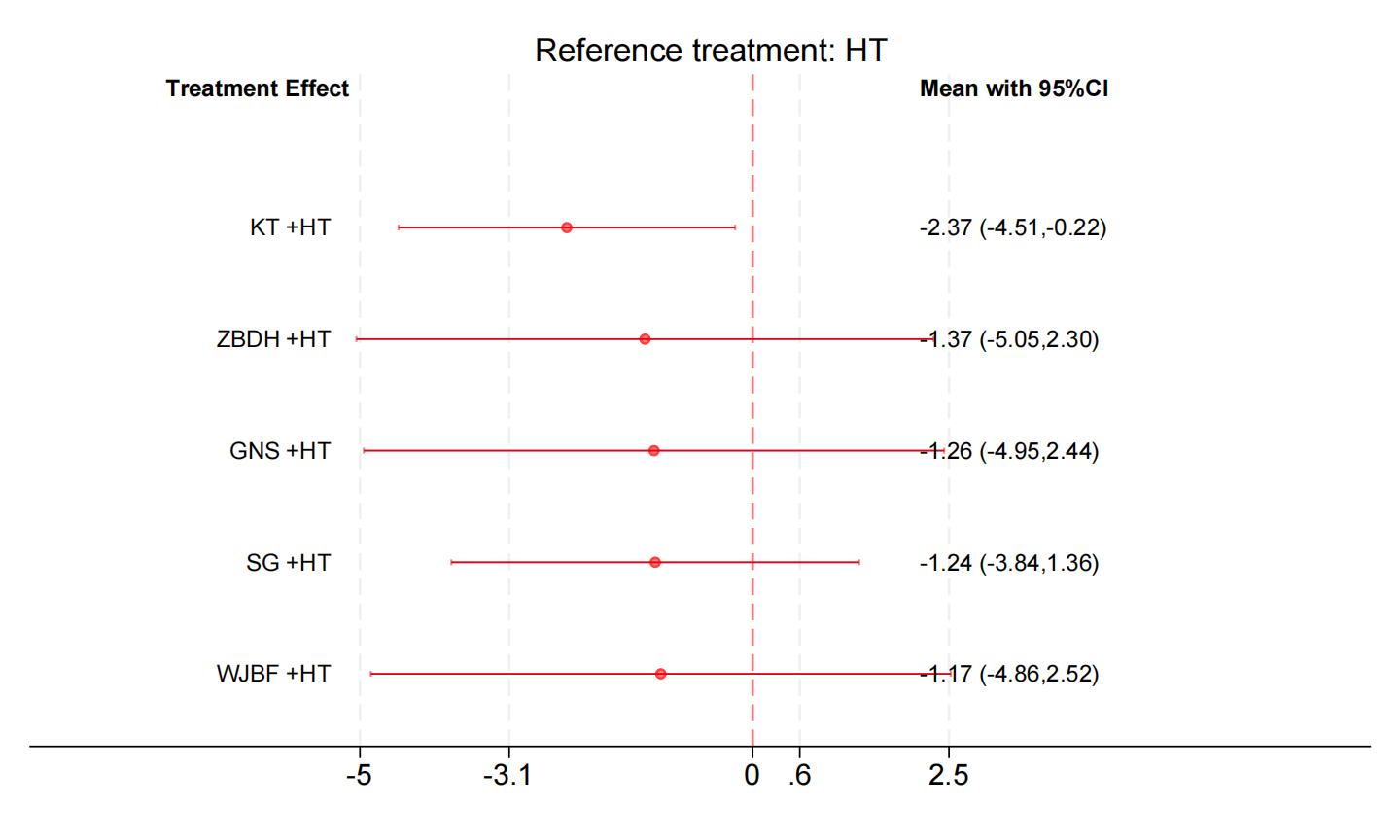


Appendix 11. Sensitivity analyses for KI: standardized mean differences (SMD) with 95% confidence intervals (CI)

| **Intervention** | **Primary analysis** | **Excluding high-RoB studies** | **Excluding small-sample studies** | **Excluding progestogen-only studies** |
| --- | --- | --- | --- | --- |
| SG +HT | -1.25 (-3.65,1.15) | -1.24 (-3.90,1.43) | -1.23 (-3.59,1.13) | -1.23 (-3.69,1.23) |
| DK +HT | -3.03 (-4.46,-1.59) | -3.10 (-4.72,-1.47) | -3.26 (-4.85,-1.67) | -3.18 (-4.84,-1.51) |
| LWDH +HT | -1.84 (-3.54,-0.14) | -1.84 (-3.79,0.10) | -1.84 (-3.56,-0.12) | -2.05 (-4.13,0.03) |
| DZ +HT | -3.24 (-5.67,-0.81) | -3.24 (-6.01,-0.47) | -3.24 (-5.69,-0.78) | -3.24 (-5.80,-0.68) |
| XS +HT | -0.84 (-2.37,0.68) | -0.39 (-2.64,1.85) | -0.84 (-2.39,0.70) | -0.84 (-2.45,0.77) |
| KT +HT | -2.13 (-3.03,-1.23) | -2.12 (-3.18,-1.06) | -2.13 (-3.04,-1.22) | -2.09 (-3.08,-1.11) |
| WJBF +HT | -1.31 (-3.72,1.10) | -1.17 (-5.06,2.72) | -1.31 (-3.75,1.13) | -1.31 (-3.85,1.23) |
| GHM +HT | -1.65 (-3.62,0.32) | -1.45 (-4.21,1.31) | -1.65 (-3.64,0.34) | -1.65 (-3.73,0.43) |
| GNS +HT | -1.13 (-3.54,1.28) | -1.26 (-5.15,2.64) | -1.13 (-3.57,1.31) | -1.13 (-3.68,1.41) |
| LLH +HT | -4.36 (-6.83,-1.88) | -4.36 (-7.19,-1.54) | -4.36 (-6.86,-1.85) | -4.34 (-6.96,-1.73) |
| ZBDH +HT | -1.64 (-4.05,0.77) | -1.93 (-5.84,1.99) | -1.64 (-4.08,0.80) | -1.64 (-4.19,0.90) |

Appendix 12. Certainty of evidence assessment (CINeMA)

## Table S12.1 CINeMA certainty of evidence for KI


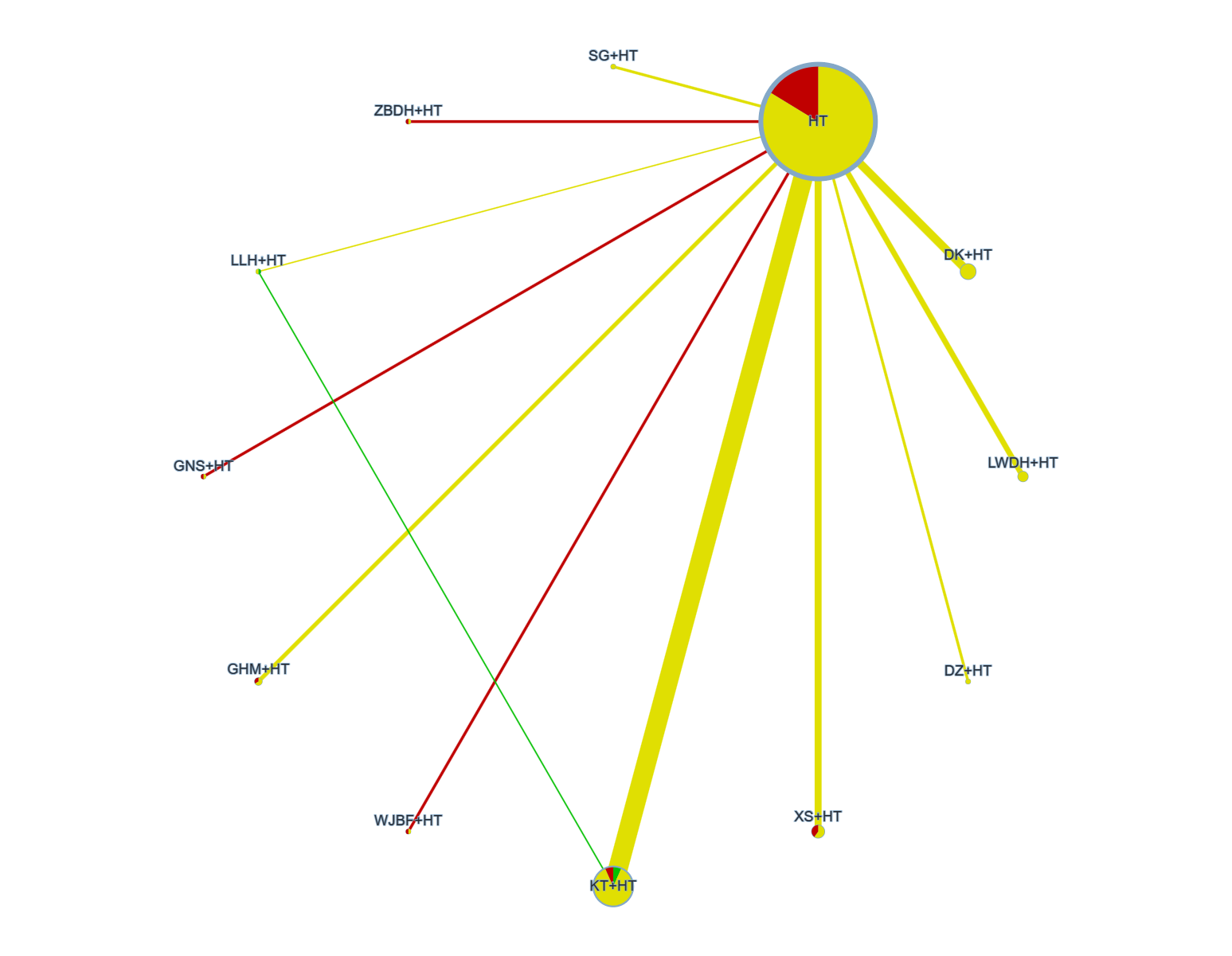


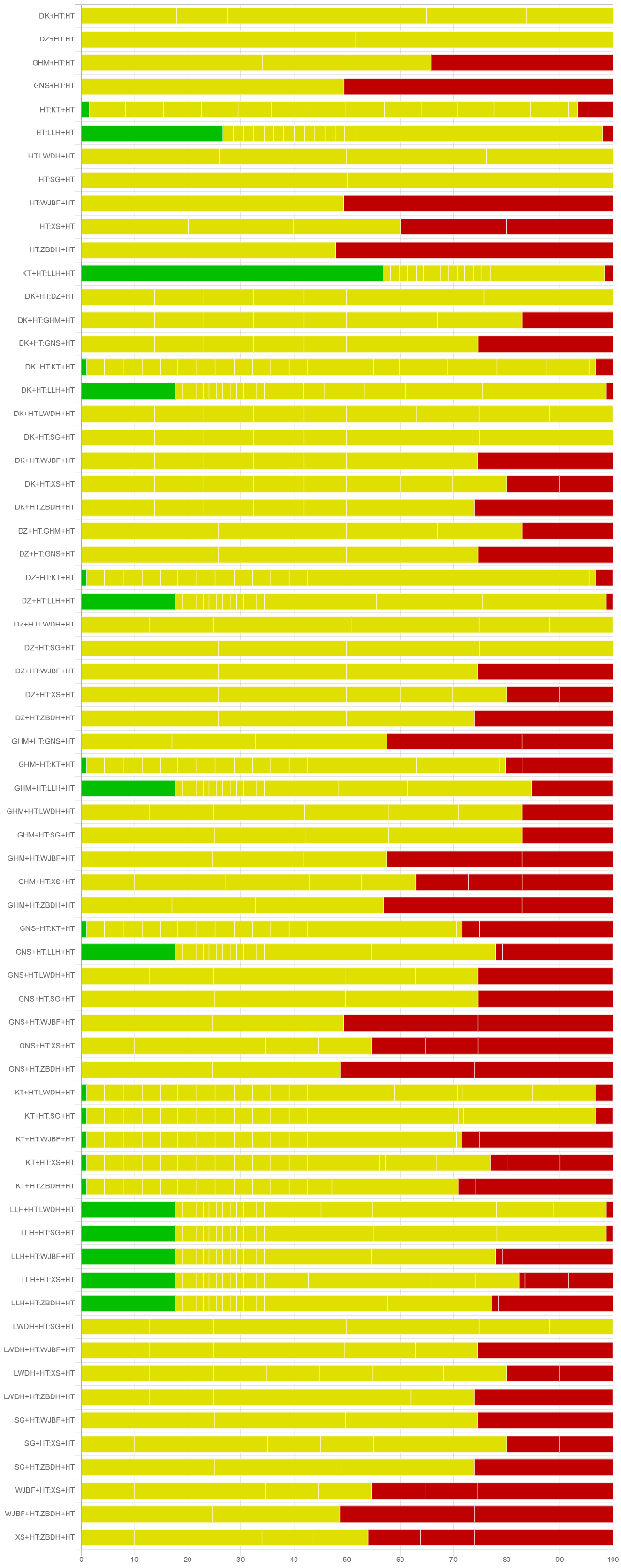


| **Comparison** | **Number of studies** | **Within-study bias** | **Reporting bias** | **Indirectness** | **Imprecision** | **Heterogeneity** | **Incoherence** | **Confidence rating** |
| --- | --- | --- | --- | --- | --- | --- | --- | --- |
| DK+HT:HT | 6 | Some concerns | Low risk | No concerns | No concerns | No concerns | Major concerns | Low |
| DZ+HT:HT | 2 | Some concerns | Low risk | No concerns | No concerns | No concerns | Major concerns | Low |
| GHM+HT:HT | 3 | Some concerns | Low risk | No concerns | No concerns | Major concerns | Major concerns | Low |
| GNS+HT:HT | 2 | Major concerns | Low risk | No concerns | Major concerns | No concerns | Major concerns | Low |
| HT: KT+HT | 14 | Some concerns | Low risk | No concerns | No concerns | No concerns | No concerns | Moderate |
| HT: LLH+HT | 1 | Some concerns | Low risk | No concerns | No concerns | No concerns | No concerns | Low |
| HT: LWDH+HT | 4 | Some concerns | Low risk | No concerns | No concerns | Major concerns | Major concerns | Low |
| HT: SG+HT | 2 | Some concerns | Low risk | No concerns | Major concerns | No concerns | Major concerns | Low |
| HT: WJBF+HT | 2 | Major concerns | Low risk | No concerns | No concerns | Major concerns | Major concerns | Low |
| HT: XS+HT | 5 | Some concerns | Low risk | No concerns | No concerns | Major concerns | Major concerns | Low |
| HT: ZBDH+HT | 2 | Major concerns | Low risk | No concerns | No concerns | Major concerns | Major concerns | Low |

Appendix 13. Reported adverse reactions and limitations of safety reporting

| **Study (ID)** | **Intervention (T vs C)** | **Breast tenderness (T/C)** | **Gastrointestinal reactions (T/C)** | **Unexpected vaginal bleeding (T/C)** | **Headache (T/C)** | **Dizziness (T/C)** | **Weight change (T/C)** | **Skin rash (T/C)** | **Abnormal liver function (T/C)** | **Other adverse reactions (Description: T/C)** |
| --- | --- | --- | --- | --- | --- | --- | --- | --- | --- | --- |
| An W 2024 | DK+HT vs HT | NR / NR | NR / NR | NR / NR | NR | NR | NR | NR | NR | NR |
| Zeng F 2023 | DK+HT vs HT | 3 / 2 | 1 / 2 | 1 / NR | NR | NR | NR | NR | NR | NR |
| Chen SM 2020 | DK+HT vs HT | 12 / 11 | 2 / 2 | 2 / 1 | NR | NR | 1 / 1 | NR | NR | NR |
| Ju LJ 2023 | DK+HT vs HT | NR / NR | NR / NR | NR / NR | NR | NR | NR | NR | NR | NR |
| Li LJ 2022 | DK+HT vs HT | 4 / 13 | NR / NR | NR / NR | NR | NR | NR | NR | NR | NR |
| Shi YR 2019 | DK+HT vs HT | NR / NR | 1 / 1 | NR / NR | NR | 1 / 0 | NR | NR | NR | Insomnia: 1 / 1 |
| Wang M 2017 | DK+HT vs HT | NR / NR | NR / NR | 2 / 0 | NR | NR | NR | NR | NR | NR |
| Wang Y 2024 | DK+HT vs HT | NR / NR | NR / NR | NR / NR | NR | NR | NR | NR | NR | NR |
| Wang YH 2022 | DK+HT vs HT | 0 / 0 | 0 / 0 | 0 / 0 | NR | NR | NR | NR | NR | NR |
| Wu J 2025 | DK+HT vs HT | NR / NR | NR / NR | NR / NR | NR | NR | NR | NR | NR | NR |
| Xi YN 2021 | DK+HT vs HT | NR / NR | NR / NR | NR / NR | NR | NR | NR | NR | NR | NR |
| Zhao HX 2024 | DK+HT vs HT | 1 / 1 | 1 / 2 | NR / NR | 1 / 2 | 1 / 2 | NR | NR | NR | NR |
| Zhu XQ 2023 | DK+HT vs HT | NR / NR | NR / NR | NR / NR | NR | NR | NR | NR | NR | NR |
| Wang Q 2020 | DZ+HT vs HT | 2 / 2 | 3 / 2 | 3 / 3 | NR | NR | NR | 2 / 1 | NR | NR |
| Wei CK 2020 | DZ+HT vs HT | NR / NR | NR / NR | NR / NR | NR | NR | NR | NR | NR | NR |
| Yuan Y 2015 | DZ+HT vs HT | NR / NR | NR / NR | NR / NR | NR | NR | NR | NR | NR | NR |
| Fei Y 2024 | GHM vs HT | 0 / 3 | 0 / 2 | 0 / 2 | NR | NR | 0 / 3 | NR | NR | NR |
| Chen JM 2023 | GHM+HT vs HT | 0 / 0 | 0 / 0 | 0 / 0 | NR | NR | NR | NR | NR | NR |
| Wang Y 2023 | GHM+HT vs HT | NR / NR | 2 / 7 | NR / NR | 2 / 0 | NR | NR | NR | NR | NR |
| Zhao J 2026 | GHM+HT vs HT | NR / NR | 1 / 1 | NR / NR | 1 / 1 | NR | NR | NR | NR | NR |
| Chen MM 2020 | GNS+HT vs HT | NR / NR | 2 / 1 | NR / NR | NR | NR | NR | NR | NR | NR |
| Liao Y 2021 | GNS+HT vs HT | NR / NR | 3 / 2 | NR / NR | NR | NR | NR | NR | NR | NR |
| Liu QH 2016 | GNS+HT vs HT | NR / NR | NR / NR | NR / NR | NR | NR | NR | NR | NR | NR |
| Yang QX 2019 | GNS+HT vs HT | NR / NR | 2 / 1 | NR / NR | NR | NR | NR | NR | NR | NR |
| Chen LM 2024 | KT+HT vs HT | 0 / 3 | 1 / 4 | 1 / 2 | NR | NR | NR | NR | NR | NR |
| Chen XF 2022 | KT+HT vs HT | 0 / 2 | 8 / 6 | NR / NR | 2 / 4 | NR | NR | NR | NR | NR |
| Cuan MR 2020 | KT+HT vs HT | 1 / 0 | 0 / 0 | 1 / 2 | NR | NR | 1 / 1 | 1 / 0 | NR | NR |
| Shan D 2019 | KT+HT vs HT | 1 / 2 | 1 / 3 | 0 / 2 | NR | 0 / 2 | NR | NR | NR | NR |
| Ding KQ 2018 | KT+HT vs HT | 1 / 4 | 2 / 5 | 0 / 1 | NR | NR | NR | NR | NR | NR |
| Ding YL 2025 | KT+HT vs HT | NR / NR | NR / NR | NR / NR | NR | NR | NR | NR | NR | NR |
| Gong CL 2016 | KT+HT vs HT | NR / NR | NR / NR | NR / NR | NR | NR | NR | NR | NR | NR |
| Hou JX 2017 | KT+HT vs HT | 0 / 0 | 0 / 0 | 0 / 0 | NR | NR | NR | NR | NR | NR |
| Ji Y 2016 | KT+HT vs HT | 3 / 2 | 1 / 2 | 0 / 1 | NR | NR | NR | NR | NR | NR |
| Li SS 2023 | KT+HT vs HT | 0 / 0 | 0 / 0 | 0 / 0 | NR | NR | NR | NR | NR | NR |
| Li XH 2021 | KT+HT vs HT | 1 / 2 | 4 / 5 | NR / NR | NR | NR | NR | NR | NR | NR |
| Liu CF 2020 | KT+HT vs HT | 1 / 3 | 1 / 3 | 0 / 2 | NR | NR | NR | NR | NR | NR |
| Liu WW 2025 | KT+HT vs HT | 3 / 3 | 3 / 2 | NR / NR | 2 / 1 | NR | NR | NR | NR | NR |
| OuYang H 2022 | KT+HT vs HT | 1 / 1 | 7 / 6 | NR / NR | NR | NR | NR | NR | NR | NR |
| Qi BB 2025 | KT+HT vs HT | 5 / 4 | 3 / 2 | 1 / 1 | NR | NR | NR | NR | NR | NR |
| Shi LX 2022 | KT+HT vs HT | 3 / 2 | 4 / 3 | 2 / 2 | 2 / 1 | NR | NR | NR | NR | NR |
| Wang HW 2025 | KT+HT vs HT | 1 / 7 | 0 / 4 | 0 / 3 | NR | NR | NR | NR | NR | NR |
| Yan XH 2019 | KT+HT vs HT | NR / NR | NR / NR | NR / NR | NR | NR | NR | NR | NR | NR |
| Yang B 2017 | KT+HT vs HT | NR / NR | NR / NR | NR / NR | NR | NR | NR | NR | NR | NR |
| Yin H 2025 | KT+HT vs HT | 2 / 2 | 4 / 2 | 1 / 3 | NR | NR | NR | NR | NR | Fatigue: 0 / 1 |
| Yu JX 2025 | KT+HT vs HT | 1 / 1 | NR / NR | NR / NR | NR | NR | NR | NR | NR | Endometrial thickening: 1 / 1 |
| Zhou YY 2023 | KT+HT vs HT | NR / NR | NR / NR | NR / NR | NR | NR | NR | NR | NR | NR |
| Lu Y 2018 | LLH vs KT | NR / NR | 2 / 2 | NR / NR | NR | NR | NR | NR | 1 / 1 | NR |
| Liu WY 2022 | LLH+HT vs HT | NR / NR | NR / NR | NR / NR | NR | NR | NR | NR | NR | NR |
| Chen L 2013 | LWDH vs HT | 0 / 0 | 0 / 0 | 0 / 0 | NR | NR | NR | NR | NR | NR |
| Chen Y 2022 | LWDH+HT vs HT | NR / NR | NR / NR | NR / NR | NR | NR | NR | NR | NR | NR |
| Chen Y 2024 | LWDH+HT vs HT | NR / NR | NR / NR | NR / NR | NR | NR | NR | NR | NR | NR |
| Fu XN 2021 | LWDH+HT vs HT | 1 / 0 | 2 / 3 | 1 / 0 | NR | NR | NR | NR | NR | NR |
| Ren W 2019 | LWDH+HT vs HT | 2 / 1 | 3 / 3 | 1 / 1 | NR | NR | NR | NR | NR | NR |
| Wang XH 2023 | LWDH+HT vs HT | 0 / 1 | 1 / 1 | NR / NR | NR | NR | NR | NR | NR | NR |
| Wu XJ 2022 | LWDH+HT vs HT | 0 / 1 | 2 / 2 | NR / NR | NR | NR | NR | NR | NR | NR |
| Wu YL 2023 | LWDH+HT vs HT | NR / NR | NR / NR | NR / NR | NR | NR | NR | NR | NR | NR |
| Zhou CQ 2015 | SG vs HT | NR / NR | NR / NR | NR / NR | NR | NR | NR | NR | NR | NR |
| Liu Y 2020 | SG+HT vs HT | NR / NR | NR / NR | NR / NR | NR | NR | NR | NR | NR | NR |
| Zhang Z 2019 | SG+HT vs HT | 0 / 0 | 0 / 0 | 0 / 0 | NR | NR | NR | NR | NR | NR |
| Zhao AM 2013 | SG+HT vs HT | 0 / NR | 0 / 3 | 0 / NR | NR | NR | NR | NR | NR | NR |
| Fan X 2018 | WJBF+HT vs HT | 0 / 0 | 0 / 0 | 0 / 0 | NR | NR | NR | NR | NR | NR |
| Li SM 2019 | WJBF+HT vs HT | 2 / 2 | 0 / 1 | NR / NR | NR | NR | NR | NR | NR | NR |
| Cai ZM 2016 | XS vs HT | NR / NR | NR / NR | NR / NR | NR | NR | NR | NR | NR | NR |
| Wu YQ 2014 | XS vs HT | NR / NR | NR / NR | NR / NR | NR | NR | NR | NR | 0 / 1 | NR |
| Li WL 2022 | XS vs KT | NR / NR | NR / NR | NR / NR | NR | NR | NR | NR | NR | NR |
| Chen YL 2019 | XS+HT vs HT | NR / NR | NR / NR | NR / NR | NR | NR | NR | NR | NR | NR |
| Fang Q 2024 | XS+HT vs HT | NR / NR | 1 / 0 | 1 / 0 | NR | NR | NR | NR | NR | Arthralgia: 0/1; Alopecia: 0/1 |
| Geng YN 2021 | XS+HT vs HT | 4 / 3 | NR / NR | 1 / 1 | 3 / 2 | NR | 5 / 4 | NR | NR | NR |
| Kong XJ 2025 | XS+HT vs HT | NR / NR | 1 / 2 | NR / NR | NR | 1 / 0 | NR | NR | NR | NR |
| Zhang Y 2025 | XS+HT vs HT | NR / NR | NR / NR | NR / NR | NR | NR | NR | NR | NR | NR |
| Tang JJ 2003 | ZBDH+HT vs HT | NR / NR | NR / NR | NR / NR | NR | NR | NR | NR | NR | NR |
| Xiang D 2025 | ZBDH+HT vs HT | 0 / 0 | 0 / 0 | 0 / 0 | NR | NR | NR | NR | NR | NR |
| Yan JH 2015 | ZBDH+HT vs HT | NR / NR | NR / NR | NR / NR | NR | NR | NR | NR | NR | NR |

This appendix summarizes adverse-event categories reported in the included RCTs. “NR” indicates that the specific adverse-event category was not reported and should not be interpreted as absence of that event. Many trials reported only total adverse-event counts or did not provide detailed information on event severity, causality assessment, withdrawals due to adverse events, follow-up after treatment, or potential botanical drug–hormone interactions. Because the level of detail in adverse-event reporting differed across studies and CCPP nodes, differential reporting may have introduced reporting bias into the safety analysis. Therefore, this appendix should be interpreted as a summary of reported short-term adverse reactions rather than as a comprehensive safety or interaction assessment.
